# Global, regional, and national burden of disorders affecting the nervous system, 1990–2021: a systematic analysis for the Global Burden of Disease Study 2021

**DOI:** 10.1016/S1474-4422(24)00038-3

**Published:** 2024-04

**Authors:** GBD 2021 Nervous System Disorders Collaborators, Jaimie D Steinmetz, Katrin Maria Seeher, Nicoline Schiess, Emma Nichols, Bochen Cao, Chiara Servili, Vanessa Cavallera, Ewerton Cousin, Hailey Hagins, Madeline E Moberg, Max L Mehlman, Yohannes Habtegiorgis Abate, Jaffar Abbas, Madineh Akram Abbasi, Mohammadreza Abbasian, Hedayat Abbastabar, Michael Abdelmasseh, Mohammad Abdollahi, Mozhan Abdollahi, Mohammad-Amin Abdollahifar, Rami Abd-Rabu, Deldar Morad Abdulah, Auwal Abdullahi, Aidin Abedi, Vida Abedi, Roberto Ariel Abeldaño Zuñiga, Hassan Abidi, Olumide Abiodun, Richard Gyan Aboagye, Hassan Abolhassani, Victor Aboyans, Woldu Aberhe Abrha, Ahmed Abualhasan, Eman Abu-Gharbieh, Salahdein Aburuz, Lawan Hassan Adamu, Isaac Yeboah Addo, Oladimeji M Adebayo, Victor Adekanmbi, Tayo Alex Adekiya, Wirawan Adikusuma, Qorinah Estiningtyas Sakilah Adnani, Saryia Adra, Tsion Afework, Aanuoluwapo Adeyimika Afolabi, Ali Afraz, Saira Afzal, Shahin Aghamiri, Antonella Agodi, Williams Agyemang-Duah, Bright Opoku Ahinkorah, Aqeel Ahmad, Danish Ahmad, Sajjad Ahmad, Amir Mahmoud Ahmadzade, Ali Ahmed, Ayman Ahmed, Haroon Ahmed, Jivan Qasim Ahmed, Luai A Ahmed, Muktar Beshir Ahmed, Syed Anees Ahmed, Marjan Ajami, Budi Aji, Olufemi Ajumobi, Seyed Esma'il Akade, Morteza Akbari, Hossein Akbarialiabad, Shiva Akhlaghi, Karolina Akinosoglou, Rufus Olusola Akinyemi, Maxwell Akonde, Syed Mahfuz Al Hasan, Fares Alahdab, Tareq Mohammed Ali AL-Ahdal, Rasmieh Mustafa Al-amer, Mohammed Albashtawy, Mohammad T AlBataineh, Khalifah A Aldawsari, Hediyeh Alemi, Sharifullah Alemi, Abdelazeem M Algammal, Adel Ali Saeed Al-Gheethi, Fadwa Alhalaiqa Naji Alhalaiqa, Robert Kaba Alhassan, Abid Ali, Endale Alemayehu Ali, Liaqat Ali, Mohammed Usman Ali, Musa Mohammed Ali, Rafat Ali, Shahid Ali, Syed Shujait Shujait Ali, Zahid Ali, Sheikh Mohammad Alif, Yousef Alimohamadi, Ahmednur Adem Aliyi, Mohamad Aljofan, Syed Mohamed Aljunid, Suvarna Alladi, Joseph Uy Almazan, Sami Almustanyir, Basem Al-Omari, Jaber S Alqahtani, Ibrahim Alqasmi, Ahmed Yaseen Alqutaibi, Rustam Al-Shahi Salman, Zaid Altaany, Jaffar A Al-Tawfiq, Khalid A Altirkawi, Nelson Alvis-Guzman, Yaser Mohammed Al-Worafi, Hany Aly, Safwat Aly, Karem H Alzoubi, Reza Amani, Alireza Amindarolzarbi, Sohrab Amiri, Mohammad Hosein Amirzade-Iranaq, Hubert Amu, Dickson A Amugsi, Ganiyu Adeniyi Amusa, Jimoh Amzat, Robert Ancuceanu, Deanna Anderlini, David B Anderson, Catalina Liliana Andrei, Sofia Androudi, Dhanalakshmi Angappan, Teklit W Angesom, Abhishek Anil, Alireza Ansari-Moghaddam, Razique Anwer, Mosab Arafat, Aleksandr Y Aravkin, Demelash Areda, Hany Ariffin, Hidayat Arifin, Mesay Arkew, Johan Ärnlöv, Mahwish Arooj, Anton A Artamonov, Kurnia Dwi Artanti, Raphael Taiwo Aruleba, Ali A Asadi-Pooya, Tilahun Ferede Asena, Mohammad Asghari-Jafarabadi, Muhammad Ashraf, Tahira Ashraf, Kendalem Asmare Atalell, Seyyed Shamsadin Athari, Bantalem Tilaye Tilaye Atinafu, Prince Atorkey, Maha Moh'd Wahbi Atout, Alok Atreya, Avinash Aujayeb, Abolfazl Avan, Beatriz Paulina Ayala Quintanilla, Haleh Ayatollahi, Olatunde O Ayinde, Seyed Mohammad Ayyoubzadeh, Sina Azadnajafabad, Zahra Azizi, Khalil Azizian, Ahmed Y Azzam, Mahsa Babaei, Muhammad Badar, Ashish D Badiye, Soroush Baghdadi, Sara Bagherieh, Ruhai Bai, Atif Amin Baig, Senthilkumar Balakrishnan, Shivanthi Balalla, Ovidiu Constantin Baltatu, Maciej Banach, Soham Bandyopadhyay, Indrajit Banerjee, Mehmet Firat Baran, Miguel A Barboza, Martina Barchitta, Mainak Bardhan, Suzanne Lyn Barker-Collo, Till Winfried Bärnighausen, Amadou Barrow, Davood Bashash, Hamideh Bashiri, Hameed Akande Bashiru, Afisu Basiru, João Diogo Basso, Sanjay Basu, Abdul-Monim Mohammad Batiha, Kavita Batra, Bernhard T Baune, Neeraj Bedi, Ahmet Begde, Tahmina Begum, Babak Behnam, Amir Hossein Behnoush, Maryam Beiranvand, Yannick Béjot, Alehegn Bekele, Melaku Ashagrie Belete, Uzma Iqbal Belgaumi, Maryam Bemanalizadeh, Rose G Bender, Bright Benfor, Derrick A Bennett, Isabela M Bensenor, Betyna Berice, Paulo J G Bettencourt, Kebede A Beyene, Abhishek Bhadra, Devidas S Bhagat, Kayleigh Bhangdia, Nikha Bhardwaj, Pankaj Bhardwaj, Ashish Bhargava, Sonu Bhaskar, Ajay Nagesh Bhat, Vivek Bhat, Gurjit Kaur Bhatti, Jasvinder Singh Bhatti, Rajbir Bhatti, Ali Bijani, Boris Bikbov, Mariah Malak Bilalaga, Atanu Biswas, Saeid Bitaraf, Veera R Bitra, Tone Bjørge, Virginia Bodolica, Aadam Olalekan Bodunrin, Archith Boloor, Dejana Braithwaite, Carol Brayne, Hermann Brenner, Andrey Briko, Maria L Bringas Vega, Julie Brown, Christine M Budke, Danilo Buonsenso, Katrin Burkart, Richard A Burns, Yasser Bustanji, Muhammad Hammad Butt, Nadeem Shafique Butt, Zahid A Butt, Lucas Scotta Cabral, Florentino Luciano Caetano dos Santos, Daniela Calina, Ismael R Campos-Nonato, Chao Cao, Hélène Carabin, Rosario Cárdenas, Giulia Carreras, Andre F Carvalho, Carlos A Castañeda-Orjuela, Adriano Casulli, Ferrán Catalá-López, Alberico L Catapano, Arthur Caye, Luca Cegolon, Muthia Cenderadewi, Ester Cerin, Pamela R Uscamaita Chacón-Uscamaita, Jeffrey Shi Kai Chan, Gashaw Sisay Chanie, Jaykaran Charan, Vijay Kumar Chattu, Endeshaw Chekol Abebe, Hui Chen, Jianqi Chen, Gerald Chi, Fatemeh Chichagi, Saravana Babu Chidambaram, Ritesh Chimoriya, Patrick R Ching, Abdulaal Chitheer, Yuen Yu Chong, Hitesh Chopra, Sonali Gajanan Choudhari, Enayet Karim Chowdhury, Rajiv Chowdhury, Hanne Christensen, Dinh-Toi Chu, Isaac Sunday Chukwu, Eric Chung, Kaleb Coberly, Alyssa Columbus, Josielli Comachio, Joao Conde, Paolo Angelo Cortesi, Vera Marisa Costa, Rosa A S Couto, Michael H Criqui, Natália Cruz-Martins, Mohammad Amin Dabbagh Ohadi, Sriharsha Dadana, Omid Dadras, Xiaochen Dai, Zhaoli Dai, Emanuele D'Amico, Hadi A Danawi, Lalit Dandona, Rakhi Dandona, Amira Hamed Darwish, Saswati Das, Subasish Das, Ana Maria Dascalu, Nihar Ranjan Dash, Mohsen Dashti, Fernando Pio De la Hoz, Alejandro de la Torre-Luque, Diego De Leo, Frances E Dean, Amin Dehghan, Azizallah Dehghan, Hiwot Dejene, Daniel Demant, Andreas K Demetriades, Solomon Demissie, Xinlei Deng, Hardik Dineshbhai Desai, Vinoth Gnana Chellaiyan Devanbu, Kuldeep Dhama, Samath Dhamminda Dharmaratne, Meghnath Dhimal, Diana Dias da Silva, Daniel Diaz, Mahmoud Dibas, Delaney D Ding, Monica Dinu, M Ashworth Dirac, Mengistie Diress, Thanh Chi Do, Thao Huynh Phuong Do, Khanh Duy Khanh Doan, Milad Dodangeh, Mohamed Fahmy Doheim, Klara Georgieva Dokova, Deepa Dongarwar, Haneil Larson Dsouza, John Dube, Senbagam Duraisamy, Oyewole Christopher Durojaiye, Sulagna Dutta, Arkadiusz Marian Dziedzic, Hisham Atan Edinur, Negin Eissazade, Michael Ekholuenetale, Temitope Cyrus Ekundayo, Nevine El Nahas, Iman El Sayed, Mohammad Amin Elahi Najafi, Iffat Elbarazi, Noha Mousaad Elemam, Frank J Elgar, Islam Y Elgendy, Hala Rashad Elhabashy, Muhammed Elhadi, Legesse Tesfaye Elilo, Richard G Ellenbogen, Omar Abdelsadek Abdou Elmeligy, Mohamed A Elmonem, Mohammed Elshaer, Ibrahim Elsohaby, Mehdi Emamverdi, Theophilus I Emeto, Matthias Endres, Christopher Imokhuede Esezobor, Sharareh Eskandarieh, Abdolmajid Fadaei, Adeniyi Francis Fagbamigbe, Ayesha Fahim, Ali Faramarzi, Jawad Fares, Mohsen Farjoud Kouhanjani, Andre Faro, Farshad Farzadfar, Ali Fatehizadeh, Mobina Fathi, Saeid Fathi, Syeda Anum Fatima Fatima, Alireza Feizkhah, Seyed-Mohammad Fereshtehnejad, Alize J Ferrari, Nuno Ferreira, Getahun Fetensa, Neda Firouraghi, Florian Fischer, Ana Catarina Fonseca, Lisa M Force, Arianna Fornari, Behzad Foroutan, Takeshi Fukumoto, Muktar A Gadanya, Abhay Motiramji Gaidhane, Yaseen Galali, Nasrin Galehdar, Quan Gan, Aravind P Gandhi, Balasankar Ganesan, William M Gardner, Naval Garg, Shuo-Yan Gau, Rupesh K Gautam, Teshome Gebre, Mesfin Gebrehiwot, Gebreamlak Gebremedhn Gebremeskel, Haftay Gebremedhin Gebreslassie, Lemma Getacher, Bardiya Ghaderi Yazdi, Fataneh Ghadirian, Fariborz Ghaffarpasand, Reza Ghanbari, MohammadReza Ghasemi, Ramy Mohamed Ghazy, Sailaja Ghimire, Ali Gholami, Ali Gholamrezanezhad, Elena Ghotbi, Sherief Ghozy, Alessandro Gialluisi, Paramjit Singh Gill, Logan M Glasstetter, Elena V Gnedovskaya, Ali Golchin, Mahaveer Golechha, Pouya Goleij, Davide Golinelli, Mansueto Gomes-Neto, Alessandra C Goulart, Anmol Goyal, Richard J Gray, Michal Grivna, Habtamu Alganeh Guadie, Bin Guan, Giovanni Guarducci, Stefano Guicciardi, Damitha Asanga Gunawardane, Hanbing Guo, Bhawna Gupta, Rajeev Gupta, Sapna Gupta, Veer Bala Gupta, Vivek Kumar Gupta, Reyna Alma Gutiérrez, Farrokh Habibzadeh, Vladimir Hachinski, Rasool Haddadi, Mostafa Hadei, Najah R Hadi, Nils Haep, Teklehaimanot Gereziher Haile, Arvin Haj-Mirzaian, Brian J Hall, Rabih Halwani, Sajid Hameed, Mohammad Hamiduzzaman, Ahmad Hammoud, Hannah Han, Nasrin Hanifi, Graeme J Hankey, Md. Abdul Hannan, Junwei Hao, Harapan Harapan, Habtamu Endashaw Hareru, Arief Hargono, Netanja I. Harlianto, Josep Maria Haro, Nicholas Nathaniel Hartman, Ahmed I. Hasaballah, Faizul Hasan, Hamidreza Hasani, Mohammad Hasanian, Amr Hassan, Shoaib Hassan, Soheil Hassanipour, Hadi Hassankhani, Mohammed Bheser Hassen, Johannes Haubold, Simon I Hay, Khezar Hayat, Mohamed I Hegazy, Golnaz Heidari, Mohammad Heidari, Reza Heidari-Soureshjani, Hamed Hesami, Kamal Hezam, Yuta Hiraike, Howard J Hoffman, Ramesh Holla, Kathleen Pillsbury Hopf, Nobuyuki Horita, Md Mahbub Hossain, Md. Belal Hossain, Sahadat Hossain, Hassan Hosseinzadeh, Mehdi Hosseinzadeh, Sorin Hostiuc, Chengxi Hu, Junjie Huang, Md. Nazmul Huda, Javid Hussain, Nawfal R Hussein, Hong-Han Huynh, Bing-Fang Hwang, Segun Emmanuel Ibitoye, Mehran Ilaghi, Olayinka Stephen Ilesanmi, Irena M Ilic, Milena D Ilic, Mustapha Immurana, Farideh Iravanpour, Sheikh Mohammed Shariful Islam, Faisal Ismail, Hiroyasu Iso, Gaetano Isola, Masao Iwagami, Chidozie C D Iwu, Mahalaxmi Iyer, Ali Jaan, Louis Jacob, Farhad Jadidi-Niaragh, Mahboobeh Jafari, Morteza Jafarinia, Abdollah Jafarzadeh, Kasra Jahankhani, Nader Jahanmehr, Haitham Jahrami, Abhishek Jaiswal, Mihajlo Jakovljevic, Roland Dominic G Jamora, Somnath Jana, Nilofer Javadi, Saad Javed, Saad Javeed, Sathish Kumar Jayapal, Shubha Jayaram, Heng Jiang, Catherine Owens Johnson, Walter D Johnson, Mohammad Jokar, Jost B Jonas, Abel Joseph, Nitin Joseph, Charity Ehimwenma Joshua, Mikk Jürisson, Ali Kabir, Zubair Kabir, Gebisa Guyasa Kabito, Vidya Kadashetti, Fatemeh Kafi, Rizwan Kalani, Farnaz Kalantar, Feroze Kaliyadan, Ashwin Kamath, Sagarika Kamath, Tanuj Kanchan, Amit Kandel, Himal Kandel, Kehinde Kazeem Kanmodi, Mehrdad Karajizadeh, Jafar Karami, Shama D Karanth, Ibraheem M Karaye, André Karch, Aliasghar Karimi, Hanie Karimi, Arman Karimi Behnagh, Hengameh Kasraei, Nicholas J Kassebaum, Joonas H Kauppila, Harkiran Kaur, Navjot Kaur, Gbenga A Kayode, Foad Kazemi, Leila Keikavoosi-Arani, Cathleen Keller, Mohammad Keykhaei, Mohammad Amin Khadembashiri, Yousef Saleh Khader, Morteza Abdullatif Khafaie, Himanshu Khajuria, Amirmohammad Khalaji, Faham Khamesipour, Mohammad Khammarnia, Maseer Khan, Moien AB Khan, Yusra H Khan, Mahammed Ziauddin Khan Suheb, Shaghayegh Khanmohammadi, Tripti Khanna, Khaled Khatab, Haitham Khatatbeh, Moawiah Mohammad Khatatbeh, Sorour Khateri, Mahalaqua Nazli Khatib, Hamid Reza Khayat Kashani, Mohammad Saeid Khonji, Fatemeh khorashadizadeh, Moein Khormali, Jagdish Khubchandani, Saeid Kian, Grace Kim, Jihee Kim, Min Seo Kim, Yun Jin Kim, Ruth W Kimokoti, Adnan Kisa, Sezer Kisa, Mika Kivimäki, Sonali Kochhar, Ali-Asghar Kolahi, Kamrun Nahar Koly, Farzad Kompani, Walter J Koroshetz, Soewarta Kosen, Masoumeh Kourosh Arami, Ai Koyanagi, Michael A Kravchenko, Kewal Krishan, Vijay Krishnamoorthy, Barthelemy Kuate Defo, Md Abdul Kuddus, Ashish Kumar, G Anil Kumar, Manasi Kumar, Nithin Kumar, Netsanet Bogale Kumsa, Satyajit Kundu, Maria Dyah Kurniasari, Dian Kusuma, Ambily Kuttikkattu, Hmwe Hmwe Kyu, Carlo La Vecchia, Muhammad Awwal Ladan, Chandrakant Lahariya, Tri Laksono, Dharmesh Kumar Lal, Tea Lallukka, Judit Lám, Faris Hasan Lami, Iván Landires, Berthold Langguth, Savita Lasrado, Kamaluddin Latief, Kaveh Latifinaibin, Kathryn Mei-Ming Lau, Matthew B Laurens, Basira Kankia Lawal, Long Khanh Dao Le, Thao Thi Thu Le, Caterina Ledda, Munjae Lee, Sang-woong Lee, Seung Won Lee, Wei-Chen Lee, Yo Han Lee, Matilde Leonardi, Temesgen L Lerango, Ming-Chieh Li, Wei Li, Virendra S Ligade, Stephen S Lim, Christine Linehan, Chaojie Liu, Jue Liu, Wei Liu, Chun-Han Lo, Warren David Lo, Stany W Lobo, Giancarlo Logroscino, Graciliana Lopes, Platon D Lopukhov, László Lorenzovici, Stefan Lorkowski, Joana A Loureiro, Jailos Lubinda, Giancarlo Lucchetti, Ricardo Lutzky Saute, Zheng Feei Ma, Mahmoud Mabrok, Monika Machoy, Farzan Madadizadeh, Mohammed Magdy Abd El Razek, Azzam A Maghazachi, Nastaran Maghbouli, Soleiman Mahjoub, Morteza Mahmoudi, Azeem Majeed, Jeadran N. Malagón-Rojas, Elaheh Malakan Rad, Kashish Malhotra, Ahmad Azam Malik, Iram Malik, Tauqeer Hussain Mallhi, Deborah Carvalho Malta, Aseer Manilal, Vahid Mansouri, Mohammad Ali Mansournia, Bishnu P Marasini, Hamid Reza Marateb, Seyed Farzad Maroufi, Jose Martinez-Raga, Santi Martini, Francisco Rogerlândio Martins-Melo, Miquel Martorell, Winfried März, Roy Rillera Marzo, João Massano, Yasith Mathangasinghe, Elezebeth Mathews, Richard James Maude, Andrea Maugeri, Pallab K Maulik, Mahsa Mayeli, Maryam Mazaheri, Colm McAlinden, John J McGrath, Jitendra Kumar Meena, Man Mohan Mehndiratta, Max Alberto Mendez Mendez-Lopez, Walter Mendoza, Oliver Mendoza-Cano, Ritesh G Menezes, Mohsen Merati, Atte Meretoja, Alexander Merkin, Abera M Mersha, Tomislav Mestrovic, Tianyue Mi, Tomasz Miazgowski, Irmina Maria Michalek, Ephrem Tesfaye Mihretie, Le Huu Nhat Minh, Reza Mirfakhraie, Andreea Mirica, Erkin M Mirrakhimov, Mehdi Mirzaei, Awoke Misganaw, Sanjeev Misra, Prasanna Mithra, Biru Abdissa Mizana, Ashraf Mohamadkhani, Nouh Saad Mohamed, Esmaeil Mohammadi, Hiwa Mohammadi, Shadieh Mohammadi, Soheil Mohammadi, Marita Mohammadshahi, Mustapha Mohammed, Salahuddin Mohammed, Shafiu Mohammed, Syam Mohan, Hoda Mojiri-forushani, Nagabhishek Moka, Ali H Mokdad, Sabrina Molinaro, Holger Möller, Lorenzo Monasta, Md Moniruzzaman, Fateme Montazeri, Maryam Moradi, Yousef Moradi, Maziar Moradi-Lakeh, Paula Moraga, Negar Morovatdar, Shane Douglas Morrison, Abbas Mosapour, Jonathan F Mosser, Elias Mossialos, Majid Motaghinejad, Parsa Mousavi, Seyed Ehsan Mousavi, Sumaira Mubarik, Lorenzo Muccioli, Faraz Mughal, George Duke Mukoro, Admir Mulita, Francesk Mulita, Fungai Musaigwa, Ahmad Mustafa, Ghulam Mustafa, Sathish Muthu, Ahamarshan Jayaraman Nagarajan, Pirouz Naghavi, Ganesh R Naik, Firzan Nainu, Tapas Sadasivan Nair, Hastyar Hama Rashid Najmuldeen, Noureddin Nakhostin Ansari, Gopal Nambi, Hossein Namdar Areshtanab, Shumaila Nargus, Bruno Ramos Nascimento, Abdallah Y Naser, Abdulqadir J J Nashwan, Hadis Nasoori, Ahmed Nasreldein, Zuhair S Natto, Javaid Nauman, Biswa Prakash Nayak, Athare Nazri-Panjaki, Mohammad Negaresh, Hadush Negash, Ionut Negoi, Ruxandra Irina Negoi, Serban Mircea Negru, Seyed Aria Nejadghaderi, Mohammad Hadi Nematollahi, Olivia D Nesbit, Charles Richard James Newton, Dang H Nguyen, Hau Thi Hien Nguyen, Hien Quang Nguyen, Ngoc-Trinh Thi Nguyen, Phat Tuan Nguyen, Van Thanh Nguyen, Robina Khan Niazi, Taxiarchis Konstantinos Nikolouzakis, Vikram Niranjan, Lawrence Achilles Nnyanzi, Efaq Ali Noman, Nafise Noroozi, Bo Norrving, Jean Jacques Noubiap, Chisom Adaobi Nri-Ezedi, George Ntaios, Virginia Nuñez-Samudio, Dieta Nurrika, Bogdan Oancea, Ismail A Odetokun, Martin James O'Donnell, Ropo Ebenezer Ogunsakin, James Odhiambo Oguta, In-Hwan Oh, Hassan Okati-Aliabad, Sylvester Reuben Okeke, Akinkunmi Paul Okekunle, Osaretin Christabel Okonji, Patrick Godwin Okwute, Andrew T Olagunju, Muideen Tunbosun Olaiya, Matifan Dereje Olana, Matthew Idowu Olatubi, Gláucia Maria Moraes Oliveira, Isaac Iyinoluwa Olufadewa, Bolajoko Olubukunola Olusanya, Ahmed Omar Bali, Sokking Ong, Obinna E Onwujekwe, Michal Ordak, Aislyn U Orji, Doris V Ortega-Altamirano, Uchechukwu Levi Osuagwu, Nikita Otstavnov, Stanislav S Otstavnov, Amel Ouyahia, Mayowa O Owolabi, Mahesh Padukudru P A, Kevin Pacheco-Barrios, Jagadish Rao Padubidri, Pramod Kumar Pal, Padmavali Nanaji Palange, Claudia Palladino, Raffaele Palladino, Raul Felipe Palma-Alvarez, Feng Pan, Demosthenes Panagiotakos, Songhomitra Panda-Jonas, Anamika Pandey, Ashok Pandey, Jeyaraj Durai Pandian, Helena Ullyartha Pangaribuan, Ioannis Pantazopoulos, Shahina Pardhan, Pragyan Paramita Parija, Romil R Parikh, Seoyeon Park, Ashwaghosha Parthasarathi, Ava Pashaei, Jay Patel, Shankargouda Patil, Dimitrios Patoulias, Shrikant Pawar, Paolo Pedersini, Umberto Pensato, David M Pereira, Jeevan Pereira, Maria Odete Pereira, Mario F P Peres, Norberto Perico, Simone Perna, Ionela-Roxana Petcu, Fanny Emily Petermann-Rocha, Hoang Tran Pham, Michael R Phillips, Gabriel D Pinilla-Monsalve, Michael A Piradov, Evgenii Plotnikov, Dimitri Poddighe, Burcu Polat, Ramesh Poluru, Constance Dimity Pond, Govinda Raj Poudel, Alireza Pouramini, Ali Mohammad Pourbagher-Shahri, Mohammad Pourfridoni, Naeimeh Pourtaheri, Peralam Yegneswaran Prakash, Sanjay Prakash, V Prakash, Elton Junio Sady Prates, Natalie Pritchett, Hery Purnobasuki, Nameer Hashim Qasim, Ibrahim Qattea, Gangzhen Qian, Venkatraman Radhakrishnan, Pourya Raee, Hadi Raeisi Shahraki, Ibrar Rafique, Alberto Raggi, Pankaja Raghav Raghav, Meghdad M Rahati, Fakher Rahim, Zahra Rahimi, Mahban Rahimifard, Md Obaidur Rahman, Mohammad Hifz Ur Rahman, Mosiur Rahman, Muhammad Aziz Rahman, Amir Masoud Rahmani, Shayan Rahmani, Hamed Rahmani Youshanlouei, Masoud Rahmati, Sheetal Raj Moolambally, Ali Rajabpour-Sanati, Hazem Ramadan, Shakthi Kumaran Ramasamy, Premkumar Ramasubramani, Sheena Ramazanu, Nemanja Rancic, Indu Ramachandra Rao, Sowmya J Rao, Deepthi Rapaka, Vahid Rashedi, Ahmed Mustafa Rashid, Mohammad-Mahdi Rashidi, Mehran Rashidi Alavijeh, Ashkan Rasouli-Saravani, Salman Rawaf, Christian Razo, Elrashdy Moustafa Mohamed Redwan, Atefe Rekabi Bana, Giuseppe Remuzzi, Nazila Rezaei, Negar Rezaei, Nima Rezaei, Mohsen Rezaeian, Taeho Gregory Rhee, Abanoub Riad, Stephen R Robinson, Mónica Rodrigues, Jefferson Antonio Buendia Rodriguez, Leonardo Roever, Emma L B Rogowski, Michele Romoli, Luca Ronfani, Priyanka Roy, Koushik Roy Pramanik, Enrico Rubagotti, Milagros A Ruiz, Tom C Russ, Katharina S Sunnerhagen, Aly M A Saad, Zahra Saadatian, Korosh Saber, Morteza SaberiKamarposhti, Simona Sacco, Basema Saddik, Erfan Sadeghi, Saeid Sadeghian, Umar Saeed, Usman Saeed, Mahdi Safdarian, Sher Zaman Safi, Rajesh Sagar, Dominic Sagoe, Fatemeh Saheb Sharif-Askari, Narjes Saheb Sharif-Askari, Amirhossein Sahebkar, Soumya Swaroop Sahoo, Mohammad Ali Sahraian, Seyed Aidin Sajedi, Joseph W Sakshaug, Mohamed A Saleh, Hossein Salehi Omran, Marwa Rashad Salem, Sohrab Salimi, Hossein Samadi Kafil, Sara Samadzadeh, Saad Samargandy, Yoseph Leonardo Samodra, Vijaya Paul Samuel, Abdallah M Samy, Nima Sanadgol, Rama Krishna Sanjeev, Francesco Sanmarchi, Damian Francesco Santomauro, Ichtiarini Nurullita Santri, Milena M Santric-Milicevic, Aswini Saravanan, Arash Sarveazad, Maheswar Satpathy, Mete Saylan, Mehdi Sayyah, Nikolaos Scarmeas, Markus P Schlaich, Art Schuermans, Michaël Schwarzinger, David C Schwebel, Siddharthan Selvaraj, Ashenafi Kibret Sendekie, Pallav Sengupta, Subramanian Senthilkumaran, Dragos Serban, Mihretu Tagesse Sergindo, Yashendra Sethi, SeyedAhmad SeyedAlinaghi, Allen Seylani, Mohammad Shabani, Maryam Shabany, Mahan Shafie, Saeed Shahabi, Ataollah Shahbandi, Samiah Shahid, Fariba Shahraki-Sanavi, Hamid R Shahsavari, Moyad Jamal Shahwan, Masood Ali Shaikh, KS Shaji, Sunder Sham, Adisu Tafari T Shama, Muhammad Aaqib Shamim, Mehran Shams-Beyranvand, Mohammad Anas Shamsi, Mohd Shanawaz, Medha Sharath, Sadaf Sharfaei, Amin Sharifan, Manoj Sharma, Rajesh Sharma, Bereket Beyene Shashamo, Maryam Shayan, Rahim Ali Sheikhi, Shashank Shekhar, Jiabin Shen, Suchitra M Shenoy, Pavanchand H Shetty, Desalegn Shiferaw Shiferaw, Mika Shigematsu, Rahman Shiri, Aminu Shittu, K M Shivakumar, Fereshteh Shokri, Sina Shool, Seyed Afshin Shorofi, Sunil Shrestha, Akhenaten Benjamin Siankam Tankwanchi, Emmanuel Edwar Siddig, Inga Dora Sigfusdottir, João Pedro Silva, Luís Manuel Lopes Rodrigues Silva, Ehsan Sinaei, Balbir Bagicha Singh, Garima Singh, Paramdeep Singh, Surjit Singh, Sarah Brooke Sirota, Shravan Sivakumar, Abdullah Al Mamun Sohag, Ranjan Solanki, Hamidreza Soleimani, Solikhah Solikhah, Yerukneh Solomon, Yonatan Solomon, Suhang Song, Yimeng Song, Houman Sotoudeh, Michael Spartalis, Benjamin A Stark, Joseph R Starnes, Antonina V Starodubova, Dan J Stein, Timothy J Steiner, Lars Jacob Stovner, Muhammad Suleman, Rizwan Suliankatchi Abdulkader, Abida Sultana, Jing Sun, David Sunkersing, Angel Sunny, Hani Susianti, Chandan Kumar Swain, Mindy D Szeto, Rafael Tabarés-Seisdedos, Seyyed Mohammad Tabatabaei, Shima Tabatabai, Mohammad Tabish, Majid Taheri, Azin Tahvildari, Ardeshir Tajbakhsh, Mircea Tampa, Jacques JL Lukenze Tamuzi, Ker-Kan Tan, Haosu Tang, Minale Tareke, Ingan Ukur Tarigan, Nathan Y Tat, Vivian Y Tat, Razieh Tavakoli Oliaee, Seyed Mohammad Tavangar, Arian Tavasol, Yibekal Manaye Tefera, Arash Tehrani-Banihashemi, Worku Animaw Temesgen, Mohamad-Hani Temsah, Masayuki Teramoto, Amensisa Hailu Tesfaye, Edosa Geta Tesfaye, Riki Tesler, Ocean Thakali, Pugazhenthan Thangaraju, Rajshree Thapa, Rekha Thapar, Nikhil Kenny Thomas, Amanda G Thrift, Jansje Henny Vera Ticoalu, Tala Tillawi, Razie Toghroli, Marcello Tonelli, Marcos Roberto Tovani-Palone, Eugenio Traini, Nghia Minh Tran, Ngoc-Ha Tran, Phu Van Tran, Samuel Joseph Tromans, Thomas Clement Truelsen, Thien Tan Tri Tai Truyen, Aristidis Tsatsakis, Guesh Mebrahtom Tsegay, Evangelia Eirini Tsermpini, Abdul Rohim Tualeka, Derara Girma Tufa, Chukwudi S Ubah, Aniefiok John Udoakang, Inam Ulhaq, Muhammad Umair, Srikanth Umakanthan, Krishna Kishore Umapathi, Brigid Unim, Bhaskaran Unnikrishnan, Asokan Govindaraj Vaithinathan, Alireza Vakilian, Sahel Valadan Tahbaz, Rohollah Valizadeh, Jef Van den Eynde, Priya Vart, Shoban Babu Varthya, Tommi Juhani Vasankari, Siavash Vaziri, Balachandar Vellingiri, Narayanaswamy Venketasubramanian, Georgios-Ioannis Verras, Dominique Vervoort, Jorge Hugo Villafañe, Leonardo Villani, Andres Fernando Vinueza Veloz, Maria Viskadourou, Sergey Konstantinovitch Vladimirov, Vasily Vlassov, Simona Ruxandra Volovat, Loc Tri Vu, Isidora S Vujcic, Birhanu Wagaye, Yasir Waheed, Waseem Wahood, Mandaras Tariku Walde, Fang Wang, Shu Wang, Yanzhong Wang, Yuan-Pang Wang, Muhammad Waqas, Abdul Waris, Kosala Gayan Weerakoon, Robert G Weintraub, Abrha Hailay Weldemariam, Ronny Westerman, Joanna L Whisnant, Dakshitha Praneeth Wickramasinghe, Nuwan Darshana Wickramasinghe, Barbara Willekens, Lauren B Wilner, Andrea Sylvia Winkler, Charles D A Wolfe, Ai-Min Wu, Sarah Wulf Hanson, Suowen Xu, Xiaoyue Xu, Ali Yadollahpour, Sajad Yaghoubi, Galal Yahya, Kazumasa Yamagishi, Lin Yang, Yuichiro Yano, Yao Yao, Sisay Shewasinad Yehualashet, Alex Yeshaneh, Metin Yesiltepe, Siyan Yi, Arzu Yiğit, Vahit Yiğit, Dong Keon Yon, Naohiro Yonemoto, Yuyi You, Mustafa Z Younis, Chuanhua Yu, Hadiza Yusuf, Siddhesh Zadey, Mohammad Zahedi, Fathiah Zakham, Nazar Zaki, Alireza Zali, Giulia Zamagni, Ramin Zand, Ghazal G Z Zandieh, Moein Zangiabadian, Amin Zarghami, Mikhail Sergeevich Zastrozhin, Mohammed G M Zeariya, Zelalem Banjaw Zegeye, Francis Zeukeng, Chunxia Zhai, Chen Zhang, Haijun Zhang, Yunquan Zhang, Zhi-Jiang Zhang, Hanqing Zhao, Yang Zhao, Peng Zheng, Hengxing Zhou, Bin Zhu, Abzal Zhumagaliuly, Magdalena Zielińska, Yossef Teshome Zikarg, Mohammad Zoladl, Christopher J L Murray, Kanyin Liane Ong, Valery L Feigin, Theo Vos, Tarun Dua

## Abstract

**Background:**

Disorders affecting the nervous system are diverse and include neurodevelopmental disorders, late-life neurodegeneration, and newly emergent conditions, such as cognitive impairment following COVID-19. Previous publications from the Global Burden of Disease, Injuries, and Risk Factor Study estimated the burden of 15 neurological conditions in 2015 and 2016, but these analyses did not include neurodevelopmental disorders, as defined by the International Classification of Diseases (ICD)-11, or a subset of cases of congenital, neonatal, and infectious conditions that cause neurological damage. Here, we estimate nervous system health loss caused by 37 unique conditions and their associated risk factors globally, regionally, and nationally from 1990 to 2021.

**Methods:**

We estimated mortality, prevalence, years lived with disability (YLDs), years of life lost (YLLs), and disability-adjusted life-years (DALYs), with corresponding 95% uncertainty intervals (UIs), by age and sex in 204 countries and territories, from 1990 to 2021. We included morbidity and deaths due to neurological conditions, for which health loss is directly due to damage to the CNS or peripheral nervous system. We also isolated neurological health loss from conditions for which nervous system morbidity is a consequence, but not the primary feature, including a subset of congenital conditions (ie, chromosomal anomalies and congenital birth defects), neonatal conditions (ie, jaundice, preterm birth, and sepsis), infectious diseases (ie, COVID-19, cystic echinococcosis, malaria, syphilis, and Zika virus disease), and diabetic neuropathy. By conducting a sequela-level analysis of the health outcomes for these conditions, only cases where nervous system damage occurred were included, and YLDs were recalculated to isolate the non-fatal burden directly attributable to nervous system health loss. A comorbidity correction was used to calculate total prevalence of all conditions that affect the nervous system combined.

**Findings:**

Globally, the 37 conditions affecting the nervous system were collectively ranked as the leading group cause of DALYs in 2021 (443 million, 95% UI 378–521), affecting 3·40 billion (3·20–3·62) individuals (43·1%, 40·5–45·9 of the global population); global DALY counts attributed to these conditions increased by 18·2% (8·7–26·7) between 1990 and 2021. Age-standardised rates of deaths per 100 000 people attributed to these conditions decreased from 1990 to 2021 by 33·6% (27·6–38·8), and age-standardised rates of DALYs attributed to these conditions decreased by 27·0% (21·5–32·4). Age-standardised prevalence was almost stable, with a change of 1·5% (0·7–2·4). The ten conditions with the highest age-standardised DALYs in 2021 were stroke, neonatal encephalopathy, migraine, Alzheimer's disease and other dementias, diabetic neuropathy, meningitis, epilepsy, neurological complications due to preterm birth, autism spectrum disorder, and nervous system cancer.

**Interpretation:**

As the leading cause of overall disease burden in the world, with increasing global DALY counts, effective prevention, treatment, and rehabilitation strategies for disorders affecting the nervous system are needed.

**Funding:**

Bill & Melinda Gates Foundation.

## Introduction

Conditions can affect the nervous system throughout life, for example by disrupting brain growth; damaging the brain, spinal cord, or peripheral nerves; and impairing cognitive, sensory, socioemotional, and motor function and behaviour. This diverse group of conditions includes congenital and neurodevelopmental disorders, cerebrovascular and neurodegenerative diseases, neurological infections, neurological–immunological disorders, neuromuscular or peripheral nervous system disorders, traumatic injuries, and cancers of the nervous system, for brevity summarised as neurological disorders or nervous system conditions. These disorders vary in cause, symptoms, and course. Some nervous system conditions cause lifelong disability, whereas others are associated with high fatality rates; some are treatable or preventable, whereas for others there is no cure.

**Research in context**

**Evidence before this study**

We searched PubMed between Jan 1, 1980, and Oct 22, 2023, to identify studies that assessed trends in nervous system health loss globally, with the search string (“nervous system”[Title] OR “neurological”[Title]) AND (“prevalen*”[Title/Abstract] OR “inciden*”[Title/Abstract] OR “death*”[Title/Abstract] OR “burden”[Title/Abstract]) AND (“global”[Title] OR “international”[Title]). Results yielded studies that either looked at a small subset of conditions, such as only cancers or COVID-19, looked at single geographies, or used Global Burden of Disease, Injuries, and Risk Factors Study (GBD) results. Previous GBD reports on the global, regional, and national burden of neurological disorders covered a period from 1990 to 2016 and were limited to 15 conditions. In 2016, neurological disorders ranked as the leading cause of DALYs and the second-leading cause of death. The largest contributors to DALYs were stroke, migraine, and Alzheimer's disease and other dementias. Other research groups used GBD 2019 results to quantify 18 neurological disorders or smaller geographies. Previous analyses excluded neurodevelopmental conditions that frequently cause lifelong disability, and neurological complications from conditions that affect multiple body systems, such as diabetes, syphilis, malaria, or more recently COVID-19 and Zika virus disease.

**Added value of this study**

This study extends previous evidence by including important neurological and neurodevelopmental disorders that were previously not considered and by adding consequences of non-neurological conditions that affect the nervous system, bringing the total number of included conditions to 37. This study estimated the proportion of nervous system burden that was potentially avertible by eliminating known risk factors for stroke, Alzheimer's disease and other dementias, multiple sclerosis, Parkinson's disease, encephalitis, meningitis, and idiopathic intellectual disability but also emphasises the scarcity of knowledge about risk factors for nervous system conditions.

**Implications of all available evidence**

Until recently, the nervous system has not been a focus of global public health discourse. Quantifying the global burden associated with nervous system health loss aids policy making and helps to lift brain health onto the public health agenda. With the adoption of the *Intersectoral Global Action Plan on Epilepsy and Other Neurological Disorders 2022–2031* by the World Health Assembly, the prevention, early identification, diagnosis, treatment, and rehabilitation of disorders that affect the nervous system have been brought into focus. This study provides the latest evidence to guide ongoing advocacy and awareness efforts. Additional research on modifiable risks, and support for adequate facilities and workforces in managing nervous system conditions, is necessary for equity and access to quality care. As the leading cause of DALYs, affecting more than 40% of the global population, nervous system health loss should be a public health priority.

Increased life expectancy is arguably one of the greatest achievements of health systems around the world. However, this increase has also led to increases in age-related neurological disorders, such as Alzheimer's disease and other dementias, stroke, and Parkinson's disease, necessitating global health policies not only to focus on survival but also to minimise health loss due to disability by promoting function and independence. Not all neurological burden is associated with population ageing,^1^ rendering it important to quantify the overall health loss associated with nervous system conditions throughout the lifespan.^2^

In response to the growing burden of nervous system disorders and conditions worldwide, the World Health Assembly adopted the *Intersectoral Global Action Plan on Epilepsy and Other Neurological Disorders 2022–2031* (IGAP) in May, 2022.^3^ The action plan aims to “reduce the stigma, impact and burden of neurological disorders, including their associated mortality, morbidity and disability, and to improve the quality of life of people with neurological disorders, their carers and families”.^3^

In the Global Burden of Disease, Injuries, and Risk Factors Study (GBD), nervous system disorders and conditions are spread across many disease groupings. The basic GBD grouping of neurological disorders includes Alzheimer's disease and other dementias, headaches, idiopathic epilepsy, motor neuron disease, multiple sclerosis, Parkinson's disease, and a residual group of other neurological disorders that includes, for instance, muscular dystrophy and Huntington's disease. Using GBD 2015 and 2016 estimates, Feigin and colleagues previously provided more comprehensive estimates of neurological health loss than the basic GBD grouping by also including stroke, meningitis, encephalitis, tetanus, traumatic brain injury, spinal cord injury, and brain and CNS cancers.^1^, ^4^ Other researchers used GBD 2019 estimates to look at 18 neurological disorders or specific geographies.^5^, ^6^ However, a proportion of global neurological burden stems from neurodevelopmental disorders, which traditionally have been classified in GBD under mental health or neonatal conditions, and from childhood infections. Both infections and neurodevelopmental disorders are often associated with lifelong disability.^7^ Furthermore, some peripheral neuropathies were not previously captured within the neurological burden.

Additionally, as shown by the COVID-19 pandemic,^8^, ^9^ emerging and re-emerging infectious diseases are becoming an increasing global concern. For example, vector-borne viruses such as Zika virus,^10^ Japanese encephalitis virus,^11^ and West Nile virus^12^ are increasing their geographical spread owing to climate change and constitute a notable global public health threat. These infections, often targeting the nervous system directly, cause both mortality and neurological morbidity, with an especially high burden in low-income and middle-income countries (LMICs).^13^

This Article aims to create an estimate of the burden of disorders and conditions that affect the nervous system using an expanded group of GBD conditions, cause categories, and disease consequences compared with previous analyses. This manuscript was produced as part of the GBD Collaborator Network and in accordance with the GBD Protocol.

## Methods

### Overview

GBD 2021 quantifies health loss for 371 diseases in 204 countries and territories, including measures of prevalence, disease severity, and death that together constitute a comprehensive assessment of disease burden. The analysis presented here groups conditions that affect the CNS and peripheral nervous system for which neurological consequences can be isolated in GBD (table 1). The conditions included here are not an exhaustive list; we were unable to include neurological consequences from some conditions, for example HIV or adrenoleukodystrophy, because they could not be explicitly estimated in GBD at this stage. However, this analysis is the most comprehensive attempt to capture neurological health loss to date. Broadly, and as outlined in the IGAP, these conditions include: neurodevelopmental disorders;^14^ neurological disorders (eg, stroke or Alzheimer's disease and other dementias); and neurological consequences of other congenital, neonatal, metabolic, or infectious diseases. Neurological consequences captured in this analysis include intellectual disability, cognitive impairment, motor impairment, epilepsy, microcephaly, neuropathy, and sensory deficits resulting from neonatal insults (described by condition in appendix p 5). For example, persistent cognitive symptoms and Guillain–Barré syndrome after COVID-19 were included in the analysis, but ongoing fatigue or respiratory symptoms due to COVID-19 were not included. Methods that were used to isolate nervous system health loss for conditions with neurological and non-neurological outcomes are described later in this section. Other neurological disorders is a residual category (appendix pp 5–6) that encompasses conditions that affect the CNS and peripheral nervous system but are not explicitly modelled as isolated diseases in GBD because of resource constraints. The category broadly includes unspecified disorders of the nervous system, some degenerative or demyelinating diseases, disorders of the autonomic nervous system, some movement disorders, spinocerebellar diseases, nerve root and plexus disorders, peripheral nerve disorders, some neuromuscular disorders, and muscle diseases, such as myopathies (appendix pp 5–6, 63–64). Epilepsy estimates exclude epilepsy cases that are captured under other conditions to avoid double counting. A Guidelines for Accurate and Transparent Health Estimates Reporting checklist is included in the appendix (pp 2–5).

**Table 1:** Conditions included in our analysis of nervous system health loss

	**Included in GBD 2016 analysis by Feigin and colleagues**^1^	**Mortality included in this analysis**
Alzheimer's disease and other dementias	Yes	Yes
Attention deficit hyperactivity disorder	No	No
Autism spectrum disorder	No	No
Cerebral malaria	No	No
Cognitive impairment or Guillain–Barré syndrome due to COVID-19	No	No
Congenital and adult neurosyphilis	No	No
Diabetic neuropathy	No	No
Encephalitis	Yes	Yes
Epilepsy	Yes (idiopathic)	Yes
Epilepsy due to cystic echinococcosis	No	No
Fetal alcohol syndrome	No	No
Guillain–Barré syndrome	No	Yes*
Idiopathic intellectual disability	No	No
Meningitis	Yes	Yes
Migraine	Yes	No
Motor neuron disease	Yes	Yes
Multiple sclerosis	Yes	Yes
Neonatal encephalopathy	No	Yes
Nervous system cancer (ie, CNS cancers, neuroblastoma, and other peripheral nervous cell tumours; includes paediatric and adult primary cases, and excludes metastases)	Yes (CNS)	Yes
Neural tube defects	No	Yes
Neurocysticercosis	No	Yes
Neurological complications due to congenital birth defects	No	No
Neurological complications due to congenital Zika syndrome	No	No
Neurological complications due to Down syndrome	No	No
Neurological complications due to Klinefelter syndrome	No	No
Neurological complications due to neonatal jaundice	No	No
Neurological complications due to neonatal sepsis	No	No
Neurological complications due to other chromosomal anomalies (excluding Down syndrome, Klinefelter syndrome, and Turner syndrome)	No	No
Neurological complications due to preterm birth	No	No
Other neurological disorders (including degenerative diseases, disorders of the autonomic nervous system, some movement disorders, spinocerebellar diseases, nerve root and plexus disorders, peripheral nerve disorders, neuromuscular disorders, and some muscle diseases such as myopathies; appendix pp 5–6)	Yes	Yes
Parkinson's disease	Yes	Yes
Rabies	No	Yes
Spinal cord injury	Yes	No
Stroke (ie, ischaemic stroke, subarachnoid haemorrhage, and intracerebral haemorrhage)	Yes	Yes
Tension-type headache	Yes	No
Tetanus	Yes	Yes
Traumatic brain injury	Yes	No

* Guillain-Barré syndrome deaths not ascribed to an underlying cause are captured in the category of other neurological disorders.

### Prevalence estimates

GBD attempts to acquire all available population-representative studies, large-scale surveys, censuses, insurance claims, and hospital records that catalogue incidence and prevalence for each condition. The number of data sources that inform non-fatal estimates for each condition are summarised in the appendix (pp 6–7). For each condition, a reference case definition and data collection method was set. Data collected with non-reference methods were adjusted to the reference case definition using regression analyses assessing systematic bias. Case definitions and International Classification of Disease (ICD) codes used in non-fatal analyses are described in the appendix (pp 8–16).

For most conditions, Bayesian models were used to estimate incidence and prevalence across time, geography, age, and sex, on the basis of data and relevant predictive covariates. Modelling details vary by condition but generally use a Bayesian meta-regression tool called Disease Modelling Meta-Regression (DisMod-MR) 2.1.^15^ Details of Dismod-MR 2.1 are in the GBD 2019 capstone appendix 1, section 4.5 of reference 9,^15^ and described in the appendix (p 16). Predictive covariates included in models for individual conditions and details on geographical categories are also described in the appendix (pp 16–18). By including remission rates in the GBD modelling approach, we counted cases as being prevalent only if they were still a case, and we further accounted for transient versus progressive conditions in the case definition (appendix pp 8–14) and assessment of burden. Details of the modelling approach of three conditions with episodic occurrence (ie, migraine, tension-type headaches, and epilepsy) are in the appendix (p 20).

This Article describes prevalence, which reflects incidence and duration. Duration in turn is determined by mortality and remission rates and reflects disease severity and access to care. Total prevalence for each condition was split into more granular categories, termed sequelae, to capture different levels of severities and different possible health outcomes (appendix pp 21–48). For example, Parkinson's disease prevalence was proportionally split between three sequelae (ie, mild, moderate, and severe).^16^ For conditions that are not purely neurological, such as COVID-19, only relevant neurological sequelae were included. Further details on non-fatal methods for these conditions and diseases are shown in the appendix (pp 5–62).

### Case aggregation and comorbidity corrections

The total number of prevalent cases for each individual condition was calculated by aggregating cases for all underlying sequelae. For wholly neurological conditions, the aggregate of sequela-level cases equalled the total number of cases for the overall condition. For conditions with both non-neurological and neurological health loss, the aggregate number of cases for sequelae with nervous system health loss was a subset of the total number of cases. For example, only a subset of all individuals with long-term consequences of COVID-19 in 2021 had neurological sequelae captured in GBD, such as cognitive impairment or Guillain–Barré syndrome (details of analysis linking Guillain–Barré syndrome as an outcome from COVID-19 infection are shown in appendix p 62). The total cases of cognitive impairment due to COVID-19 included in this analysis equals the total number of people who had cognitive impairment or Guillain–Barré syndrome attributed to COVID-19, but calculation of years lived with disability (YLDs) excludes any additional health loss in these individuals due to acute COVID-19 symptoms, fatigue, or ongoing respiratory problems.

A comorbidity correction was used to calculate total prevalence of all nervous system conditions and disorders combined, assuming independent comorbidity. Without a comorbidity correction, prevalence would be overestimated because individual conditions or disorders would be assumed to be non-overlapping (ie, one condition or disorder per individual). The total number of people with any nervous system health loss was then calculated by multiplying by the number of people in the population: prevalence_total_= 1 – [(1 – prevalence_condition1_) * (1 – prevalence_condition2_) *… (1 – prevalence_condition37_)].

### YLDs due to nervous system health loss

The YLDs measure of non-fatal burden allows for comparison of relative health effects between disparate diseases. Disability refers to the relative health loss from a condition, as reflected in disability weights that grade severity of health loss from none (ie, disability weight of 0) to severe (ie, disability weight of 1, which is equivalent to death).^17^ YLDs account for both prevalence and severity of health loss by multiplying sequela prevalence by a sequela-specific disability weight. For example, although tension-type headaches are highly prevalent, the associated disability weight is relatively low compared with those of many other neurological conditions, which is reflected in the final YLD values. As another example, the disability weight for mild multiple sclerosis is 0·183, and that for severe multiple sclerosis is 0·719 (a description of multiple sclerosis disability weights is shown in appendix pp 55–56). These weights are derived from population and internet surveys where respondents were asked to indicate the person that they believed to be healthier between random pairs of hypothetical people, each with a brief lay description of health states included in GBD. Disability weights and lay descriptions for all health states included in this analysis are described in the appendix (pp 49–61). To account for the fact that individuals can have more than one condition, we did a simulation to produce adjusted disability weights on the basis of the observed combinations of comorbidities generated by the simulation.

For sequelae that include both neurological and non-neurological health loss, YLDs were recalculated to isolate the non-fatal burden attributable to nervous system health loss. For example, sequelae of Down syndrome include severe intellectual disability with congenital heart disease due to Down syndrome (sequela 1) and severe intellectual disability due to Down syndrome (sequela 2). To isolate the neurological component of sequela 1, the adjusted disability weight generated from the comorbidity simulation for sequela 2 was used to recalculate YLDs by multiplying the prevalence of sequela 1 by the adjusted disability weight for sequela 2. This method was used to calculate YLDs for all sequelae with combined neurological and non-neurological health loss to isolate the burden due to nervous system health loss.

### Calculating deaths and years of life lost

Deaths were estimated for 15 neurological conditions (table 1). An overview of data and modelling methods is shown in the appendix (pp 62–66). Total sources included for each individual condition are in the appendix (pp 62–63); ICD-9 and ICD-10 mapping for each condition and predictive covariates included in models are in the appendix (pp 63–66).

Causes of death in GBD are mutually exclusive and collectively exhaustive, meaning a given individual is assigned only one underlying cause of death and the sum of all disease-specific deaths adds up to total deaths for a given year. Years of life lost (YLLs) were calculated for each condition by multiplying deaths and remaining standard life expectancy.^15^ This measure captures premature death, meaning that a death at a young age, for example from neonatal encephalopathy, leads to more YLLs than a death at older ages, such as from Alzheimer's disease and other dementias. Disability-adjusted life-years (DALYs) were calculated as the sum of YLDs and YLLs by year, age, sex, and location, and represent the combined non-fatal and fatal burden of each condition. For conditions where we did not ascribe deaths (eg, headaches or conditions such as preterm birth where we included only long-term neurological outcomes), DALYs are equal to YLDs. Percentage of deaths and DALYs that occurred in LMICs were calculated using World Bank income levels, which are based on gross national income per capita.

### Risk factors

GBD assesses the degree to which risk factors contribute to disease burden by identifying how disease DALYs would change given a theoretical minimum risk exposure level.^17^ The contribution of preventable risk factors to DALYs was quantified for eight conditions: stroke, Alzheimer's disease and other dementias, multiple sclerosis, Parkinson's disease, idiopathic epilepsy, meningitis, encephalitis, and idiopathic intellectual disability. We did not include conditions that are not wholly neurological, for examples diabetes, as we could not ascribe risk contribution specific to neurological health states. The number of risk factors assessed in GBD 2021 varies by condition on the basis of evidence of association and available data. Exposure to a given risk was estimated for each location, age, sex, and year using regression modelling, and relative risk curves were computed by pooling data from studies assessing a given risk–outcome pair, such as intervention, cohort, and case–control studies. For each risk–outcome pair, risk exposure and relative risk analyses were used to calculate population attributable fraction. Attributable DALYs were then calculated, defined as the expected decrease in disease burden if risk exposure had equalled the theoretical minimum risk exposure level. Total attributable burden across all risks for a given outcome accounted for mediation effects but not potential synergistic effects.

### Mean estimate, uncertainty, and percentage change calculation

Estimates were calculated 500 times by sampling the posterior distribution of the estimate (termed draws) at each step in the modelling process, and all calculations were performed by draw. Mean estimates for each year, age, sex, and location were taken from the distribution of 100 estimates, and 95% uncertainty intervals (UI) were taken as the 12·5th and 487·5th ordered draws. Age-standardised estimates were calculated using standard GBD population age weights.^15^ Percentage change between 1990 and 2021 was calculated by subtracting 1990 estimates from 2021 estimates and dividing the difference by 1990 estimates.

### Role of the funding source

The funder of the study had no role in the study design, data collection, data analysis, data interpretation, or writing of the report.

## Results

### Overview

An estimated 3·40 billion (95% UI 3·20–3·62) individuals had a condition affecting the nervous system in 2021, corresponding to 43·1% (40·5–45·9) of the world population (table 2). These conditions caused 11·1 million (9·75–13·8) deaths and contributed to 168 million (114–243) YLDs and 275 million (247–316) YLLs. With a total of 443 million (378–521) DALYs, this expanded nervous system category was the top-ranked contributor to global DALYs and YLLs in GBD 2021, followed by cardiovascular diseases (excluding stroke; unpublished estimates, GBD 2021 Diseases and Injuries Collaborators). The grouping of 15 conditions or disorders published in previous analyses^1^ contributed 313 million (260–379) DALYs, or 70·5% (66·9–74·6) of the total neurological DALYs reported here for 2021. Newly added neurological conditions contributed 2·14 million (1·53–2·82) DALYs, or 0·5% (0·4–0·6) of total neurological DALYs; neurodevelopmental and paediatric conditions contributed 80·3 million (69·6–92·5) DALYs, or 18·2% (15·3–21·2); and other conditions that include neurological health loss contributed 48·1 million (34·1–64·8) DALYs, or 10·8% (8·1–13·6) of total neurological DALYs. The percentage contribution of original and newly added groups of conditions to total DALYs is shown in the appendix (p 67). The original category of 15 conditions would have still been the top-ranked contributor to global DALYs in GBD 2021 even without the addition of new conditions. The expanded category was also the first-ranked contributor to global YLDs, followed by musculoskeletal disorders (unpublished estimates, GBD 2021 Diseases and Injuries Collaborators).

**Table 2:** Global DALYs, YLDs, YLLs, prevalence, and deaths per 100 000 people and age-standardised rates by neurological disorder category, 1990–2021

	**Counts (thousands)**		**Age-standardised rate (per 100 000 people)**				
	2021	Percentage change, 1990–2021	2021	Percentage change, 1990–2021	Females	Males	Female-to-male ratio
**All neurological conditions**							
DALYs	443 000 (378 000 to 521 000)	18·2% (8·7 to 26·7)	5637·6 (4829·7 to 6587·9)	−27·0% (−32·4 to −21·5)	5185·8 (4281·2 to 6262·9)	6101·0 (5320·2 to 6982·7)	0·85 (0·78 to 0·93)
YLDs	168 000 (114 000 to 243 000)	85·6% (75·8 to 98·0)	2064·1 (1390·0 to 2983·1)	11·2% (7·2 to 16·3)	2078·2 (1301·3 to 3133·1)	2043·6 (1439·2 to 2832·7)	1·01 (0·88 to 1·14)
YLLs	275 000 (247 000 to 316 000)	−3·1% (−11·8 to 7·7)	3573·3 (3190·9 to 4134·3)	−39·0% (−44·3 to −33·2)	3107·4 (2755·1 to 3604·8)	4057·2 (3574·4 to 4576·6)	0·77 (0·71 to 0·84)
Prevalence	3 400 000 (3 200 000 to 3 620 000)	58·8% (56·3 to 61·5)	41 204·1 (38 654·3 to 43 869·9)	1·5% (0·7 to 2·4)	43 458·5 (40 796·2 to 46 078·7)	38 949·0 (36 503·4 to 41 604·7)	1·12 (1·10 to 1·14)
Deaths	11 100 (9750 to 13 800)	41·2% (28·1 to 58·8)	139·0 (121·3 to 173·3)	−33·6% (−38·8 to −27·6)	125·0 (105·1 to 161·2)	154·6 (136·0 to 186·7)	0·81 (0·73 to 0·90)
**Alzheimer's disease and other dementias**							
DALYs	36 300 (17 200 to 77 400)	168·7% (156·3 to 179·9)	450·9 (212·9 to 956·8)	1·7% (−2·8 to 5·1)	504·8 (241·5 to 1062·7)	372·4 (170·8 to 806·4)	1·37 (1·26 to 1·46)
YLDs	11 600 (7960 to 15 300)	162·7% (157·0 to 168·0)	141·9 (97·7 to 187·2)	2·6% (1·1 to 3·6)	161·9 (110·7 to 214·8)	114·4 (78·5 to 151·2)	1·42 (1·33 to 1·49)
YLLs	24 700 (6370 to 65 700)	173·4% (153·2 to 192·5)	308·9 (78·9 to 789·0)	1·4% (−4·1 to 7·4)	342·9 (89·2 to 872·0)	258·0 (62·8 to 674·5)	1·36 (1·22 to 1·47)
Prevalence	56 900 (49 400 to 65 000)	160·8% (156·1 to 165·9)	694·0 (602·9 to 794·1)	3·2% (1·7 to 4·2)	769·9 (670·7 to 877·6)	589·5 (507·5 to 678·8)	1·31 (1·28 to 1·35)
Deaths	1950 (503 to 5080)	198·3% (175·2 to 221·8)	25·2 (6·7 to 64·4)	1·5% (−4·0 to 7·1)	27·9 (7·5 to 70·1)	20·7 (5·2 to 55·1)	1·38 (1·24 to 1·49)
**Attention deficit hyperactivity disorder**							
YLDs	1030 (572 to 1670)	18·7% (14·8 to 22·8)	13·5 (7·4 to 21·9)	−9·6% (−11·9 to −7·3)	7·7 (4·2 to 12·7)	19·0 (10·4 to 30·7)	0·40 (0·38 to 0·43)
Prevalence	84 800 (63 400 to 117 000)	18·8% (14·9 to 23·2)	1108·9 (828·7 to 1536·2)	−9·7% (−11·8 to −7·4)	636·0 (467·9 to 879·0)	1561·7 (1172·5 to 2151·7)	0·41 (0·39 to 0·43)
**Autism spectrum disorder**							
YLDs	11 500 (7840 to 16 300)	46·7% (44·5 to 48·5)	147·6 (100·2 to 208·1)	2·1% (0·6 to 3·4)	94·5 (64·6 to 133·1)	199·8 (136·3 to 281·7)	0·47 (0·46 to 0·49)
Prevalence	61 800 (52 100 to 72 700)	47·5% (45·1 to 49·4)	788·3 (663·8 to 927·2)	2·0% (0·4 to 3·1)	508·1 (424·6 to 604·3)	1064·7 (898·5 to 1245·7)	0·48 (0·46 to 0·50)
**Cerebral malaria**							
YLDs	407 (296 to 514)	109·1% (98·6 to 120·1)	5·3 (3·9 to 6·7)	53·6% (45·6 to 61·5)	5·6 (4·1 to 7·1)	5·0 (3·6 to 6·3)	1·12 (1·08 to 1·16)
Prevalence	996 (923 to 1070)	122·2% (116·4 to 128·1)	12·9 (12·0 to 13·9)	63·0% (58·7 to 67·4)	13·8 (12·7 to 14·9)	12·1 (11·2 to 13·1)	1·14 (1·13 to 1·15)
**Congenital birth defects**†							
YLDs	1450 (676 to 2750)	53·4% (48·3 to 58·4)	18·7 (8·8 to 35·4)	8·9% (5·5 to 12·4)	15·9 (7·4 to 30·4)	21·4 (10·2 to 39·9)	0·74 (0·69 to 0·80)
Prevalence	13 900 (7980 to 21 600)	56·0% (51·6 to 60·4)	180·2 (104·5 to 278·7)	11·4% (8·6 to 14·5)	157·0 (90·4 to 243·6)	203·0 (118·0 to 315·0)	0·77 (0·73 to 0·81)
**Congenital Zika syndrome**†							
YLDs	0·100 (0·100 to 0·100)	100·0% (100·0 to 100·0)	0·0 (0·0 to 0·0)	100·0% (100·0 to 100·0)	0·0 (0·0 to 0·0)	0·0 (0·0 to 0·0)	0·97 (0·93 to 1·02)
Prevalence	0·100 (0·100 to 0·200)	100·0% (100·0 to 100·0)	0·0 (0·0 to 0·0)	100·0% (100·0 to 100·0)	0·0 (0·0 to 0·0)	0·0 (0·0 to 0·0)	0·97 (0·97 to 0·97)
**COVID-19**†							
YLDs	2480 (87·2 to 7990)	100% (100 to 100)	29·4 (1·1 to 101·1)	100·0% (100·0 to 100·0)	36·9 (1·0 to 122·8)	19·7 (0·9 to 75·0)	1·84 (0·97 to 3·35)
Prevalence	23 400 (4140 to 72 800)	100% (100 to 100)	288·5 (50·5 to 899·2)	100·0% (100·0 to 100·0)	370·9 (64·3 to 1175·0)	205·7 (35·8 to 608·9)	1·87 (1·32 to 2·61)
**Cystic echinococcosis**†							
YLDs	4·60 (2·90 to 7·00)	21·1% (−0·3 to 48·3)	0·1 (0·0 to 0·1)	−24·7% (−37·7 to −9·2)	0·1 (0·0 to 0·1)	0·1 (0·0 to 0·1)	1·21 (1·14 to 1·27)
Prevalence	15·1 (11·5 to 19·3)	45·2% (27·9 to 60·2)	0·2 (0·1 to 0·2)	−10·7% (−19 to −2·0)	0·2 (0·2 to 0·3)	0·2 (0·1 to 0·2)	1·21 (1·14 to 1·27)
**Diabetic neuropathy**							
YLDs	26 300 (18 000 to 37 400)	309·1% (296·2 to 320·4)	694·0 (602·9 to 794·1)	91·9% (86·3 to 97·3)	769·9 (670·7 to 877·6)	589·5 (507·5 to 678·8)	0·92 (0·89 to 0·94)
Prevalence	206 000 (171 000 to 249 000)	310·5% (297·4 to 322·3)	301·9 (207·0 to 429·4)	92·2% (86·4 to 97·7)	289·7 (198·4 to 410·9)	315·9 (216·4 to 449·9)	0·95 (0·92 to 0·97)
**Down syndrome**†							
YLDs	135 (89·8 to 194)	−4·5% (−11·8 to 3·0)	1·8 (1·2 to 2·6)	−24·2% (−29·7 to −18·3)	1·7 (1·2 to 2·5)	1·9 (1·2 to 2·7)	0·93 (0·92 to 0·95)
Prevalence	1490 (1240 to 1780)	−5·4% (−12·6 to 2·3)	20·0 (16·7 to 24·0)	−24·6% (−30·2 to −18·6)	19·3 (16·1 to 22·9)	20·7 (17·2 to 25·0)	0·93 (0·91 to 0·94)
**Encephalitis**							
DALYs	4950 (4150 to 5700)	−17·8% (−33·4 to 4·0)	67·4 (55·7 to 78·3)	−35·7% (−47·8 to −19·0)	67·2 (58·4 to 78·2)	67·7 (50·6 to 83·5)	1·01 (0·76 to 1·26)
YLDs	497 (354 to 658)	5·0% (0·0 to 10·5)	6·2 (4·4 to 8·2)	−29·9% (−33·2 to −26·6)	6·1 (4·3 to 8·1)	6·3 (4·5 to 8·4)	0·96 (0·93 to 1·00)
YLLs	4460 (3690 to 5280)	−19·7% (−36·4 to 4·6)	61·2 (49·9 to 72·9)	−36·2% (−49·1 to −17·9)	61·2 (51·9 to 71·1)	61·4 (44·2 to 76·2)	1·01 (0·74 to 1·31)
Prevalence	4640 (3250 to 6000)	0·7% (−3·0 to 5·3)	57·3 (40·2 to 73·9)	−35·6% (−38·2 to −3·02)	54·7 (38·7 to 70·3)	59·9 (41·7 to 77·7)	0·91 (0·90 to 0·93)
Deaths	92·0 (79·0 to 108)	9·9% (−8·9 to 36·4)	1·2 (1·0 to 1·4)	−26·1% (−38·0 to −8·3)	1·2 (1·0 to 1·4)	1·2 (0·9 to 1·5)	0·98 (0·69 to 1·27)
**Epilepsy**							
DALYs	14 400 (11 000 to 18 500)	22·5% (7·7 to 38·7)	183·9 (141·0 to 237·2)	−14·6% (−24·6 to −3·9)	160·4 (118·3 to 212·2)	207·2 (161·9 to 262·2)	0·77 (0·67 to 0·86)
YLDs	7760 (4660 to 11 800)	35·6% (7·1 to 67·6)	98·9 (59·8 to 149·8)	−7·1% (−25·8 to 13·7)	94·4 (57·0 to 142·9)	103·6 (62·4 to 157·5)	0·91 (0·89 to 0·94)
YLLs	6610 (5450 to 7340)	10·7% (−0·6 to 24·4)	85·0 (70·7 to 95·0)	−21·6% (−29·6 to −12·5)	66·0 (47·6 to 76·6)	103·6 (84·2 to 117·2)	0·64 (0·45 to 0·78)
Prevalence	24 400 (18 600 to 30 800)	57·4% (30·6 to 85·6)	308·9 (236·2 to 390·1)	6·2% (−10·5 to 24·1)	294·6 (224·6 to 373·7)	323·8 (249·0 to 406·4)	0·91 (0·89 to 0·93)
Deaths	140 (116 to 153)	34·2% (23·3 to 45·8)	1·7 (1·5 to 1·9)	−15·7% (−22·5 to −8·8)	1·4 (1·0 to 1·5)	2·1 (1·8 to 2·4)	0·64 (0·46 to 0·76)
**Fetal alcohol syndrome**							
YLDs	23·6 (13·8 to 37·0)	24·0% (14·8 to 32·1)	0·3 (0·2 to 0·5)	−11·1% (−17·4 to −5·4)	0·3 (0·2 to 0·5)	0·3 (0·2 to 0·5)	0·91 (0·84 to 0·99)
Prevalence	517 (374 to 677)	33·7% (28·4 to 37·9)	6·7 (4·9 to 8·8)	−4·7% (−8·2 to −1·8)	6·2 (4·7 to 8·0)	7·1 (5·1 to 9·5)	0·88 (0·83 to 0·99)
**Guillain–Barré syndrome**							
YLDs	49·0 (31·6 to 72·5)	71·3% (61·4 to 81·9)	0·6 (0·4 to 0·9)	5·7% (3·3 to 8·2)	0·6 (0·4 to 0·8)	0·7 (0·4 to 1·0)	0·86 (0·84 to 0·89)
Prevalence	166 (134 to 201)	71·3% (61·4 to 81·9)	2·0 (1·7 to 2·5)	5·7% (3·3 to 8·2)	1·9 (1·5 to 2·3)	2·2 (1·8 to 2·7)	0·86 (0·84 to 0·89)
**Idiopathic intellectual disability**							
YLDs	3810 (1760 to 6520)	16·3% (10·5 to 22·3)	49·9 (23·2 to 85·4)	−13·6% (−18·0 to −8·4)	51·5 (25·5 to 85·6)	48·3 (20·8 to 84·7)	1·08 (1·00 to 1·20)
Prevalence	88 300 (47 100 to 129 000)	10·2% (5·7 to 13·7)	1157·2 (620·9 to 1688·1)	−18·0% (−21·1 to −15·7)	1204·4 (688·7 to 1708·4)	1110·5 (554·5 to 1667·2)	1·10 (1·02 to 1·24)
**Klinefelter syndrome**†							
YLDs	3·00 (1·50 to 5·80)	30·5% (24·1 to 37·3)	0·0 (0·0 to 0·0)	2·7% (−1·7 to 6·7)	NA	0·0 (0·0 to 0·1)	NA
Prevalence	230 (158 to 320)	30·2% (25·7 to 34·6)	3·1 (2·1 to 4·3)	2·7% (1·3 to 4·2)	NA	6·1 (4·2 to 8·4)	NA
**Meningitis**							
DALYs	14 500 (11 500 to 18 700)	−56·0% (−63·6 to −45·5)	208·5 (163·6 to 270·8)	−62·1% (−68·9 to −52·8)	193·3 (155·1 to 245·0)	223·0 (167·7 to 302·2)	0·87 (0·69 to 1·03)
YLDs	603 (425 to 791)	−31·3% (−34·3 to −28·0)	7·8 (5·5 to 10·2)	−51·7% (−53·8 to −49·4)	7·6 (5·4 to 10·0)	7·9 (5·6 to 10·4)	0·97 (0·95 to 1·00)
YLLs	13 900 (11 000 to 18 000)	−56·7% (−64·2 to −45·8)	200·7 (156·5 to 262·5)	−62·5% (−69·3 to −52·9)	185·7 (146·9 to 236·1)	215·1 (160·0 to 294·1)	0·87 (0·68 to 1·03)
Prevalence	7270 (5930 to 9070)	−35·5% (−38·2 to −32·7)	92·3 (75·2 to 114·8)	−56·8% (−58·6 to −55·1)	88·5 (72·5 to 109·7)	96·1 (78·2 to 120·0)	0·92 (0·91 to 0·93)
Deaths	214 (177 to 266)	−49·0% (−56·6 to −38·2)	2·9 (2·4 to 3·7)	−60·2% (−66·3 to −51·7)	2·7 (2·2 to 3·4)	3·2 (2·5 to 4·1)	0·87 (0·70 to 0·99)
**Migraine**							
YLDs	43 400 (6740 to 95 100)	58·9% (53·7 to 66·0)	532·7 (80·7 to 1167·8)	0·6% (−4·0 to 2·6)	662·8 (93·8 to 1450·9)	403·9 (67·5 to 872·6)	1·62 (1·39 to 1·79)
Prevalence	1 160 000 (996 000 to 1 330 000)	58·2% (54·4 to 62·6)	14 246·5 (12 194·1 to 16 378·7)	1·6% (0·3 to 2·6)	17 902·6 (15 446·0 to 20 487·0)	10 624·2 (9039·5 to 12 297·3)	1·69 (1·65 to 1·73)
**Motor neuron disease**							
DALYs	1040 (962 to 1120)	105·8% (87·1 to 125·9)	12·2 (11·2 to 13·2)	8·5% (−0·6 to 18·6)	10·1 (9·3 to 11·1)	14·4 (13·0 to 15·7)	0·70 (0·62 to 0·77)
YLDs	57·9 (40·7 to 78·0)	68·4% (61·6 to 75·9)	0·7 (0·5 to 1·0)	−1·4% (−3·4 to 0·7)	0·6 (0·4 to 0·9)	0·8 (0·5 to 1·0)	0·83 (0·81 to 0·86)
YLLs	983 (903 to 1070)	108·6% (88·7 to 130·2)	11·5 (10·5 to 12·4)	9·2% (−0·5 to 20·0)	9·4 (8·7 to 10·5)	13·7 (12·2 to 15·0)	0·69 (0·61 to 0·77)
Prevalence	273 (236 to 314)	68·6% (61·8 to 76·2)	3·3 (2·9 to 3·8)	−1·3% (−3·3 to 0·9)	3·0 (2·6 to 3·5)	3·6 (3·2 to 4·2)	0·83 (0·81 to 0·85)
Deaths	39·1 (35·6 to 42·4)	156·2% (136·0 to 178·6)	0·5 (0·4 to 0·5)	19·9% (10·9 to 30·1)	0·4 (0·3 to 0·4)	0·5 (0·5 to 0·6)	0·69 (0·62 to 0·77)
**Multiple sclerosis**							
DALYs	973 (836 to 1130)	69·5% (63·5 to 75·6)	11·4 (9·7 to 13·2)	−11·0% (−14 to −8·0)	14·5 (12·4 to 16·9)	8·1 (7·0 to 9·5)	1·80 (1·72 to 1·87)
YLDs	484 (344 to 631)	86·5% (78·9 to 94·5)	5·7 (4·0 to 7·4)	−0·4% (−3·9 to 3·1)	7·5 (5·3 to 9·7)	3·9 (2·7 to 5·1)	1·93 (1·86 to 2·00)
YLLs	490 (465 to 513)	55·7% (47·3 to 63·9)	5·7 (5·4 to 6·0)	−19·5% (−23·5 to −15·1)	7·1 (6·7 to 7·6)	4·2 (4·0 to 4·5)	1·68 (1·54 to 1·80)
Prevalence	1890 (1690 to 2110)	88·1% (80·8 to 95·5)	22·2 (19·8 to 24·8)	−0·3% (−3·6 to 3·3)	29·3 (26·2 to 32·7)	14·7 (13·0 to 16·6)	1·99 (1·94 to 2·05)
Deaths	16·3 (15·3 to 17·0)	79·1% (69·2 to 88·6)	0·2 (0·2 to 0·2)	−12·7% (−17·4 to −8·3)	0·2 (0·2 to 0·2)	0·1 (0·1 to 0·2)	1·59 (1·46 to 1·70)
**Neonatal encephalopathy**							
DALYs	58 600 (50 100 to 69 000)	−27·5% (−39·5 to −14·5)	932·1 (797·5 to 1101·7)	−26·5% (−38·9 to −13·0)	795·2 (675·1 to 942·3)	1060·2 (889·7 to 1259·1)	0·75 (0·67 to 0·87)
YLDs	4280 (3100 to 5590)	189·1% (61·5 to 348·9)	55·2 (39·9 to 72·1)	107·0% (16·2 to 220·4)	45·3 (32·5 to 59·5)	64·7 (46·7 to 84·3)	0·70 (0·68 to 0·72)
YLLs	54 300 (46 000 to 64 900)	−31·5% (−43·9 to −17·9)	876·9 (743·6 to 1056·6)	−29·2% (−42·0 to −15·3)	749·9 (627·7 to 895·0)	995·6 (824·7 to 1193·6)	0·76 (0·67 to 0·88)
Prevalence	18 600 (16 100 to 21 100)	175·2% (94·9 to 246·8)	238·1 (206·3 to 269·7)	91·2% (37·2 to 139·8)	188·4 (163·5 to 213·3)	286·6 (248·7 to 325·4)	0·66 (0·65 to 0·67)
Deaths	604 (511 to 722)	−31·5% (−44·0 to −17·9)	9·7 (8·3 to 11·7)	−29·2% (−42·0 to −15·3)	8·3 (7·0 to 10·0)	11·1 (9·2 to 13·3)	0·76 (0·67 to 0·88)
**Neonatal jaundice**†							
YLDs	718 (517 to 917)	80·0% (69·8 to 90·3)	9·3 (6·7 to 11·9)	31·5% (24·3 to 39·0)	9·5 (6·8 to 12·1)	9·2 (6·6 to 11·8)	1·03 (1·01 to 1·05)
Prevalence	1960 (1800 to 2160)	102·7% (95·8 to 109·0)	25·5 (23·4 to 28·2)	48·8% (43·8 to 53·5)	25·6 (23·5 to 28·2)	25·4 (23·3 to 28·1)	1·01 (1·00 to 1·02)
**Neonatal sepsis**†							
YLDs	2490 (1570 to 3590)	136·5% (32·3 to 322·0)	32·3 (20·3 to 46·5)	70·4% (−4·5 to 203·1)	27·0 (17·1 to 38·7)	37·3 (23·5 to 54·0)	0·72 (0·71 to 0·74)
Prevalence	7100 (5140 to 9260)	153·8% (51·5 to 332·6)	92·1 (66·8 to 120·1)	83·3% (9·5 to 212·0)	75·2 (54·7 to 97·7)	108·3 (78·3 to 141·4)	0·69 (0·69 to 0·70)
**Nervous system cancer**							
DALYs	9200 (7890 to 10 600)	50·2% (29·1 to 69·0)	111·8 (95·5 to 129·3)	−8·9% (−20·8 to 1·5)	96·8 (86·4 to 107·3)	127·5 (97·7 to 162·0)	0·77 (0·60 to 1·00)
YLDs	132 (93·8 to 174)	111·8% (89·2 to 133·2)	1·6 (1·1 to 2·1)	22·5% (9·0 to 34·4)	1·5 (1·0 to 2·0)	1·7 (1·2 to 2·3)	0·86 (0·70 to 1·08)
YLLs	9070 (7750 to 10 500)	49·6% (28·4 to 67·8)	110·3 (93·9 to 127·4)	−9·2% (−21·2 to 1·0)	95·3 (85·1 to 105·7)	125·8 (96·3 to 159·7)	0·77 (0·60 to 1·00)
Prevalence	1030 (907 to 1140)	117·1% (92·0 to 140·4)	12·8 (11·2 to 14·2)	37·7% (22·5 to 51·3)	12·5 (11·1 to 13·9)	13·2 (10·4 to 15·9)	0·95 (0·78 to 1·21)
Deaths	264 (226 to 302)	90·3% (67·8 to 110·7)	3·1 (2·7 to 3·6)	1·3% (−9·8 to 11·9)	2·7 (2·4 to 3·0)	3·6 (2·8 to 4·4)	0·75 (0·59 to 0·96)
**Neural tube defects**							
DALYs	5300 (4310 to 6510)	−47·3% (−59·0 to −24·7)	83·2 (67·8 to 102·5)	−47·7% (−59·5 to −25·1)	88·2 (64·3 to 118·1)	78·5 (61·6 to 104·5)	1·14 (0·72 to 1·61)
YLDs	333 (229 to 456)	11·8% (5·2 to 18·1)	4·5 (3·1 to 6·2)	−11·0% (−16·3 to −6·2)	4·6 (3·1 to 6·2)	4·4 (3·1 to 6·1)	1·03 (1·00 to 1·06)
YLLs	4970 (3980 to 6430)	−49·1% (−61·3 to −26·5)	78·7 (62·8 to 98·6)	−49·0% (−61·1 to −26·2)	83·7 (60·7 to 114·3)	74·1 (56·9 to 100·2)	1·15 (0·71 to 1·65)
Prevalence	1130 (965 to 1310)	11·2% (4·4 to 17·7)	15·2 (13·0 to 17·6)	−12·2% (−17·1 to −7·6)	15·4 (13·2 to 17·8)	15·0 (12·8 to 17·3)	1·03 (1·00 to 1·06)
Deaths	56·4 (45·3 to 72·8)	−48·6% (−60·9 to −25·7)	0·9 (0·7 to 1·1)	−48·8% (−61·0 to −26·0)	0·9 (0·7 to 1·3)	0·8 (0·6 to 1·1)	1·15 (0·71 to 1·66)
**Neurocysticercosis**							
DALYs	1240 (788 to 1810)	14·2% (−2·4 to 34·5)	14·6 (9·3 to 21·3)	−38·4% (−46·7 to −28·5)	15·6 (9·9 to 22·5)	13·6 (8·8 to 20·0)	1·15 (1·06 to 1·22)
YLDs	1150 (714 to 1730)	18·5% (0·6 to 41·3)	13·6 (8·4 to 20·2)	−37·3% (−46·2 to −27·0)	14·6 (9·1 to 21·5)	12·6 (7·8 to 18·9)	1·17 (1·09 to 1·24)
YLLs	81·3 (54·1 to 119)	−22·0% (−46·6 to 11·3)	1·0 (0·7 to 1·5)	−48·4% (−65·1 to −25·3)	1·0 (0·5 to 1·5)	1·1 (0·6 to 1·7)	0·94 (0·44 to 1·61)
Prevalence	4360 (3150 to 5720)	43·7% (31·2 to 59·2)	51·3 (37·2 to 67·3)	−25·1% (−29·0 to −19·7)	55·5 (40·3 to 71·9)	46·9 (33·7 to 63·1)	1·19 (1·11 to 1·25)
Deaths	1·60 (1·10 to 2·30)	−16·7% (−43·8 to 17·7)	0·0 (0·0 to 0·0)	−50·0% (−66·2 to −28·5)	0·0 (0·0 to 0·0)	0·0 (0·0 to 0·0)	0·93 (0·48 to 1·53)
**Neurosyphilis**							
YLDs	64·9 (42·2 to 93·4)	17·3% (4·8 to 31·0)	0·8 (0·5 to 1·2)	−18·5% (−27·0 to −9·4)	0·8 (0·5 to 1·1)	0·9 (0·6 to 1·3)	0·89 (0·83 to 0·94)
Prevalence	696 (563 to 857)	38·5% (25·0 to 53·3)	8·8 (7·1 to 10·8)	−6·1% (−15·5 to 4·0)	8·6 (6·9 to 10·6)	9·0 (7·3 to 11·0)	0·95 (0·93 to 0·97)
**Other chromosomal anomalies**†							
YLDs	295 (196 to 417)	7·3% (2·3 to 12·4)	4·1 (2·7 to 5·7)	−12·8% (−16·8 to −8·7)	3·3 (2·2 to 4·7)	4·7 (3·1 to 6·9)	0·71 (0·59 to 0·86)
Prevalence	3280 (2880 to 3760)	6·2% (0·9 to 11·2)	45·2 (39·5 to 52·0)	−13·0% (−17·2 to −9·0)	37·0 (32·4 to 43·0)	52·8 (44·2 to 62·9)	0·71 (0·59 to 0·85)
**Other neurological disorders**							
DALYs	4360 (3630 to 5270)	131·0% (107·3 to 155·5)	54·8 (45·3 to 66·6)	50·7% (35·4 to 65·5)	51·8 (42·7 to 63·2)	57·8 (48·0 to 69·4)	0·90 (0·84 to 0·96)
YLDs	2010 (1330 to 2870)	153·8% (110·9 to 197·8)	25·5 (16·8 to 36·6)	70·6% (43·8 to 98·8)	25·3 (16·6 to 36·2)	25·6 (16·8 to 36·9)	0·99 (0·96 to 1·02)
YLLs	2330 (2080 to 2510)	116·0% (83·3 to 140·8)	29·0 (25·9 to 31·4)	37·6% (18·3 to 52·7)	26·3 (22·7 to 28·9)	32·0 (29·0 to 34·8)	0·82 (0·74 to 0·93)
Deaths	71·5 (65·0 to 76·4)	189·8% (157·8 to 214·6)	0·9 (0·8 to 0·9)	50·1% (35·1 to 61·2)	0·8 (0·7 to 0·8)	1·0 (0·9 to 1·1)	0·78 (0·71 to 0·85)
**Parkinson's disease**							
DALYs	7470 (6730 to 8140)	161·8% (145·8 to 177·0)	89·6 (80·7 to 97·5)	10·0% (3·7 to 16·1)	68·6 (60·7 to 75·8)	117·5 (106·2 to 128·5)	0·58 (0·53 to 0·64)
YLDs	1670 (1170 to 2210)	271·2% (256·9 to 284·4)	19·6 (13·9 to 25·9)	60·6% (54·3 to 65·9)	16·1 (11·4 to 21·2)	23·9 (17·0 to 31·5)	0·67 (0·65 to 0·69)
YLLs	5800 (5250 to 6260)	141·4% (124·0 to 158·5)	70·0 (63·0 to 75·2)	1·0% (−5·9 to 7·5)	52·5 (45·4 to 58·0)	93·5 (84·6 to 101·7)	0·56 (0·50 to 0·63)
Prevalence	11 800 (10 400 to 13 400)	273·9% (260·2 to 287·3)	138·6 (123·1 to 157·6)	60·7% (54·9 to 66·2)	114·5 (102·1 to 129·8)	168·2 (148·4 to 191·7)	0·68 (0·67 to 0·69)
Deaths	388 (345 to 419)	162·2% (143·6 to 179·4)	4·8 (4·3 to 5·2)	4·1% (−2·7 to 10·3)	3·6 (3·0 to 4·0)	6·6 (5·9 to 7·1)	0·55 (0·49 to 0·61)
**Preterm birth**†							
YLDs	13 800 (9950 to 17 900)	74·0% (61·9 to 85·0)	180·7 (130·1 to 233·4)	29·2% (20·3 to 37·7)	169·3 (122·6 to 218·2)	191·4 (137·6 to 247·7)	0·89 (0·87 to 0·90)
Prevalence	97 500 (83 000 to 112 000)	64·1% (53·7 to 74·3)	1256·8 (1073·2 to 1445·9)	16·1% (8·7 to 23·7)	1146·3 (978·2 to 1315·6)	1363·9 (1162·4 to 1572·4)	0·84 (0·83 to 0·85)
**Rabies**							
DALYs	571 (325 to 826)	−59·0% (−71·8 to −44·1)	7·5 (4·2 to 11·0)	−69·9% (−79·3 to −58·8)	5·6 (3·0 to 9·9)	9·4 (5·0 to 13·6)	0·62 (0·26 to 0·93)
YLDs	0·100 (0·00 to 0·100)	−54·3% (−66·5 to −40·4)	0·0 (0·0 to 0·0)	−69·8% (−77·6 to −61·0)	0·0 (0·0 to 0·0)	0·0 (0·0 to 0·0)	0·63 (0·28 to 0·91)
YLLs	572 (324 to 832)	−59·0% (−71·9 to −44·0)	7·5 (4·2 to 11·0)	−69·9% (−79·2 to −58·8)	5·6 (3·0 to 9·9)	9·4 (5·0 to 13·6)	0·62 (0·26 to 0·93)
Prevalence	0·400 (0·200 to 0·500)	−54·3% (−66·5 to −40·4)	0·0 (0·0 to 0·0)	−69·8% (−77·6 to −61·0)	0·0 (0·0 to 0·0)	0·0 (0·0 to 0·0)	0·63 (0·28 to 0·91)
Deaths	10·1 (6·00 to 14·4)	−54·2% (−66·3 to −39·9)	0·1 (0·1 to 0·2)	−69·7% (−77·4 to −60·7)	0·1 (0·1 to 0·2)	0·2 (0·1 to 0·2)	0·62 (0·28 to 0·91)
**Spinal cord injury**							
YLDs	4570 (3200 to 6020)	28·9% (24·0 to 33·9)	54·7 (38·3 to 72·1)	−24·2% (−26·9 to −20·9)	37·9 (26·7 to 49·7)	71·6 (50·5 to 93·6)	0·53 (0·50 to 0·57)
Prevalence	15 400 (14 000 to 17 100)	42·0% (37·7 to 46·5)	183·9 (167·3 to 204·2)	−17·8% (−20·4 to −14·9)	131·5 (118·6 to 146·8)	236·6 (215·0 to 262·3)	0·56 (0·52 to 0·59)
**Stroke**							
DALYs	160 000 (148 000 to 172 000)	32·2% (21·7 to 42·6)	1886·0 (1740·1 to 2017·3)	−38·7% (−43·4 to −34·0)	1578·2 (1431·4 to 1710·6)	2232·3 (2028·8 to 2449·0)	0·71 (0·64 to 0·78)
YLDs	15 200 (11 000 to 19 400)	89·9% (85·9 to 94·0)	178·7 (128·9 to 227·6)	−7·4% (−9·0 to −5·9)	175·6 (126·7 to 223·1)	183·4 (132·7 to 234·5)	0·96 (0·94 to 0·98)
YLLs	145 000 (134 000 to 157 000)	28·2% (16·9 to 38·9)	1707·4 (1572·6 to 1838·0)	−40·8% (−45·8 to −35·8)	1402·6 (1263·6 to 1527·5)	2048·9 (1866·9 to 2253·8)	0·69 (0·61 to 0·77)
Prevalence	93 800 (89 000 to 99 300)	86·1% (83·0 to 89·4)	1099·3 (1044·2 to 1162·1)	−8·5% (−9·7 to −7·3)	1027·7 (974·4 to 1088·1)	1184·4 (1124·2 to 1252·1)	0·87 (0·85 to 0·88)
Deaths	7250 (6610 to 7820)	44·1% (32·6 to 56·2)	87·4 (78·6 to 94·2)	−39·4% (−44·0 to −34·7)	74·5 (66·0 to 81·7)	103·1 (93·1 to 112·8)	0·72 (0·64 to 0·81)
**Tension-type headache**							
YLDs	4600 (1350 to 15 000)	63·4% (49·6 to 69·5)	55·7 (16·1 to 185·1)	−3·1% (−4·8 to 0·9)	62·0 (18·8 to 189·0)	49·3 (13·5 to 172·4)	1·34 (0·94 to 1·43)
Prevalence	2 010 000 (1 780 000 to 2 270 000)	56·4% (52·7 to 60·2)	24 764·8 (21 863·6 to 27 954·7)	−0·6% (−1·3 to 0·2)	25 634·4 (22 631·5 to 28 974·3)	23 880·8 (21 046·2 to 26 935·1)	1·07 (1·06 to 1·09)
**Tetanus**							
DALYs	1340 (633 to 2130)	−91·7% (−94·4 to −86·8)	19·6 (9·1 to 31·9)	−92·6% (−94·9 to −88·0)	18·4 (8·6 to 31·6)	20·8 (8·3 to 36·8)	0·95 (0·49 to 1·69)
YLDs	3·00 (2·20 to 4·00)	−56·1% (−62·3 to −47·8)	0·0 (0·0 to 0·1)	−67·7% (−72·1 to −61·9)	0·0 (0·0 to 0·1)	0·0 (0·0 to 0·1)	0·91 (0·83 to 0·98)
YLLs	1340 (644 to 2180)	−91·8% (−94·4 to −86·3)	19·6 (9·1 to 32·2)	−92·6% (−95·0 to −88·1)	18·4 (8·5 to 31·6)	20·7 (8·3 to 36·8)	0·95 (0·49 to 1·69)
Prevalence	37·7 (28·5 to 49·2)	−62·2% (−66·3 to −58·1)	0·5 (0·4 to 0·6)	−72·8% (−75·5 to −70·1)	0·4 (0·3 to 0·6)	0·5 (0·4 to 0·7)	0·83 (0·79 to 0·87)
Deaths	21·3 (9·90 to 33·2)	−89·7% (−92·8 to −83·8)	0·3 (0·1 to 0·5)	−91·8% (−94·2 to −87·2)	0·3 (0·1 to 0·4)	0·3 (0·1 to 0·6)	0·85 (0·44 to 1·52)
**Traumatic brain injury**							
YLDs	5490 (3880 to 7340)	52·2% (49·5 to 55·2)	64·8 (45·8 to 86·8)	−16·4% (−17·7 to −15·0)	40·1 (28·5 to 53·7)	90·1 (63·5 to 120·8)	0·45 (0·43 to 0·46)
Prevalence	38 000 (36 400 to 39 800)	52·7% (50·4 to 55·4)	448·6 (429·9 to 470·3)	−16·9% (−18·0 to −15·6)	283·7 (270·2 to 299·2)	617·4 (592·2 to 646·0)	0·46 (0·45 to 0·47)

Data are mean (95% uncertainty interval). Counts are provided to three significant figures, rates and percentages are provided to one decimal place, and ratios are provided to two decimal places. Due to rounding, age-standardised rates for some rare conditions (eg, tetanus, rabies, neurocysticercosis, and congenital Zika syndrome) are shown as 0·0, but the actual values are higher than 0·0. To avoid double-counting cases, epilepsy includes all epilepsy not due to other causes explicitly analysed here (eg, preterm birth), and Guillain–Barré syndrome excludes Guillain–Barré syndrome due to COVID-19, as these estimates are included under COVID-19 estimates. DALYs=disability-adjusted life-years. NA=not applicable. YLDs=years lived with disability. YLLs=years of life lost.

*Values higher than 1 indicate higher levels in females.

† Neurological complications related to this condition.

For total nervous system health loss, global DALY counts increased by 18·2% (95% UI 8·7 to 26·7), from 375 million (339–419) DALYs in 1990 to 443 million (378–521) DALYs in 2021 (table 2). By contrast, age-standardised DALY rates decreased by 27·0% (21·5 to 32·4), from 7712·5 (6965·3 to 8626·0) per 100 000 people in 1990 to 5637·6 (4829·7 to 6587·9) per 100 000 people in 2021. Global YLLs remained almost constant, with a change of –3·1% (–11·8 to 7·7) from 284 million (268 to 302) YLLs in 1990 to 275 million (247 to 316) in 2021. By contrast, age-standardised YLL rates decreased 39·0% (33·2 to 44·3), from 5853·3 (5525·9 to 6272·7) per 100 000 people in 1990 to 3573·3 (3190·9 to 4134·3) per 100 000 people in 2021. Global YLDs increased 85·6% (75·8 to 98·0), from 91·0 million (58·6 to 134) YLDs in 1990 to 168 million (114 to 243) in 2021. Age-standardised YLDs rates increased by 11·2% (7·2 to 16·3), from 1859·4 (1217·7 to 2701·4) per 100 000 people in 1990 to 2064·1 (1390·0 to 2983·1) per 100 000 people in 2021 (table 2; appendix pp 68–69).

Regionally, age-standardised DALY rates were highest in western sub-Saharan Africa (8190·6 [95% UI 6986·0–9548·9] per 100 000 people) and central sub-Saharan Africa (7967·5 [6665·8–9546·6] per 100 000 people) and lowest in Australasia (2882·6 [2253·6–3717·3] per 100 000 people) and high-income Asia Pacific (2984·6 [2359·4–3768·2] per 100 000 people; appendix pp 70–78), with large differences observed for children younger than 5 years. For example, DALY rates for children younger than 5 years were approximately 18-fold higher in western sub-Saharan Africa (29 334·5 [23 721·8–35 170·5]) than in Australasia (1604·1 [1405·5–1846·4]), driven by conditions including neonatal encephalopathy, meningitis, and encephalitis. Age-standardised YLDs were similar between regions, ranging from a minimum in east Asia of 1698·6 (1133·9–2446·0) per 100 000 people to a maximum in the Caribbean of 2327·5 (1571·2–3295·6) per 100 000 people. Age-standardised YLLs had greater regional variation, ranging from a minimum in Australasia of 1098·9 (910·3–1456·4) to a maximum in western sub-Saharan Africa of 6163·8 (5131·0–7314·6; appendix pp 70–79). Using World Bank income levels, 81·9% (77·5–84·9; 9·10 million of 11·1 million) of deaths and 84·7% (83·1–86·3; 375 million of 443 million) of DALYs attributable to neurological conditions were in LMICs.

**Figure 1:**
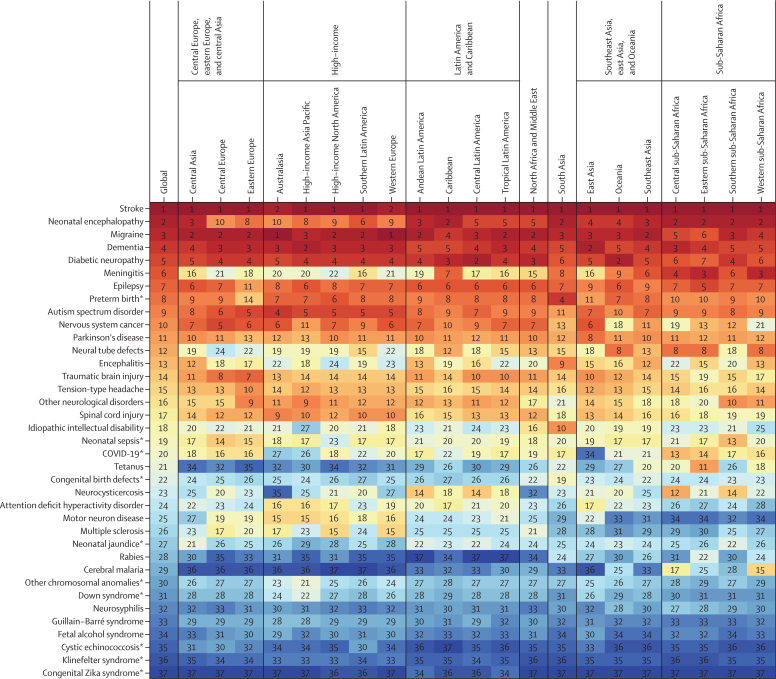
Ranking of age-standardised DALY rates for all conditions with neurological health loss by GBD region in 2021 Regions are grouped by GBD super-region and alphabetically ordered. Individual conditions are ordered by global ranking from highest age-standardised DALY rates to lowest age-standardised DALY rates. Dementia represents Alzheimer's disease and other dementias. DALYs=disability-adjusted life-years. GBD=Global Burden of Diseases, Injuries and Risk Factors Study. *Rankings are isolated to disease DALYs due to neurological complications, as opposed to DALYs attributed to the entire condition.

The ten conditions that accounted for the greatest nervous system DALYs in 2021 were stroke, neonatal encephalopathy, migraine, Alzheimer's disease and other dementias, diabetic neuropathy, meningitis, epilepsy, neurological complications due to preterm birth, autism spectrum disorder, and nervous system cancer, with stroke being the greatest contributor globally and in 19 of 21 GBD regions (table 2; figure 1). Notably, four of the top ten conditions were not included in our previous analyses of neurological burden: neonatal encephalopathy, diabetic neuropathy, neurological complications due to preterm birth, and autism spectrum disorder, which emphasises the effects of early life and childhood conditions on total nervous system health loss and the under-recognised effects on the peripheral nervous system.

Regional rankings across conditions were mostly consistent (figure 1). Exceptions included regional variation for meningitis (regional ranking range: third to 22nd), tetanus (range: 11th to 35th), multiple sclerosis (range: 15th to 31st), and motor neuron disease (range: 15th to 34th). Some infectious disease rankings reflected their regional pathogen endemicity (eg, cerebral malaria, neurocysticercosis, and neurological complications due to congenital Zika syndrome).

For children younger than 5 years, the three leading causes of DALYs globally were neonatal encephalopathy (8316·7 [95% UI 7072·6–9991·8] per 100 000 people), meningitis (1234·9 [869·5–1741·7] per 100 000 people), and neural tube defects (722·6 [580·8–899·4] per 100 000 people). For older children and adolescents aged 5–19 years, the three leading causes of DALYs were migraine (380·0 [24·7–946·6] per 100 000 people), neurological complications due to preterm birth (234·3 [168·4–305·2] per 100 000 people), and epilepsy (185·1 [137·0–261·7] per 100 000 people). For adults aged 20–59 years, the leading causes were stroke (1126·1 [1035·5–1218·4] per 100 000 people), migraine (750·8 [117·3–1617·8] per 100 000 people), and diabetic neuropathy (260·5 [171·9–387·6] per 100 000 people), and for adults aged 60–79 years, the leading causes were stroke (8490·9 [7827·5–9108·6] per 100 000 people), Alzheimer's disease and other dementias (1504·2 [746·6–3345·6] per 100 000 people), and diabetic neuropathy (1397·3 [911·8–1930·8] per 100 000 people). For adults aged 80 years and older, the leading causes were stroke (20 336·1 [17 762·6–22 137·4] per 100 000 people), Alzheimer's disease and other dementias (13 047·1 [5903·4–27 898·8] per 100 000 people), and Parkinson's disease (1773·2 [1550·8–1925·0] per 100 000 people; table 3).

**Table 3:** Global disability-adjusted life-years per 100 000 people by five broad age categories for all conditions with neurological health loss

	**Aged <5 years**	**Rank**	**Aged 5–19 years**	**Rank**	**Aged 20–59 years**	**Rank**	**Aged 60–79 years**	**Rank**	**Aged ≥80 years**	**Rank**
All neurological conditions	11 806·6 (10 144·1–13 813·9)	NA	1705·4 (1227·4–2372·8)	NA	3443·1 (2639·2–4531·4)	NA	13 742·8 (12 096·7–15 569·1)	NA	38 329·3 (31 687·1–50 519·4)	NA
Alzheimer's disease and other dementias	0·0 (0·0–0·0)	NA	0·0 (0·0–0·0)	NA	44·2 (21·2–101·5)	13	1504·2 (746·6–3345·6)	2	13 047·1 (5903·4–27 898·8)	2
Attention deficit hyperactivity disorder	2·4 (1·2–4·2)	24	28·3 (14·5–48)	14	10·7 (5·8–17·1)	23	1·0 (0·5–1·9)	29	0·0 (0·0–0·0)	33
Autism spectrum disorder	169·1 (114·9–237·3)	7	161·4 (109·2–227·2)	4	146·2 (99·4–206·3)	6	112·9 (77·4–157·1)	9	59·5 (40·5–83·6)	14
Cerebral malaria	2·8 (2·0–3·6)	23	8·8 (6·4–11·2)	22	5·0 (3·6–6·3)	27	0·8 (0·6–1·0)	30	0·0 (0·0–0·0)	32
Congenital birth defects*	27·2 (15·4–46·4)	14	20·5 (9·4–39·1)	15	17·4 (7·9–33·5)	19	13·1 (5·8–25·5)	23	10·0 (4·3–19·5)	20
Congenital Zika syndrome*	0·0 (0·0–0·0)	30	0·0 (0·0–0·0)	NA	0·0 (0·0–0·0)	NA	0·0 (0·0–0·0)	NA	0·0 (0·0–0·0)	NA
COVID-19*	8·6 (0·5–35·7)	18	15·4 (1·0–65·3)	18	40·1 (1·0–133·9)	16	32·6 (0·9–97·5)	18	30·0 (0·9–79·2)	17
Cystic echinococcosis*	0·0 (0·0–0·0)	31	0·0 (0·0–0·1)	34	0·1 (0·0–0·1)	35	0·1 (0·1–0·1)	35	0·0 (0·0–0·1)	31
Diabetic neuropathy	0·0 (0·0–0·0)	32	1·5 (0·8–2·4)	27	260·5 (171·9–387·6)	3	1397·3 (911·8–1930·8)	3	1493·5 (1026·5–2052·6)	4
Down syndrome*	3·2 (2·1–4·7)	22	3·0 (2·0–4·3)	25	1·3 (0·8–1·8)	31	0·1 (0·1–0·2)	34	0·0 (0·0–0·0)	NA
Encephalitis	270·6 (196·3–342·6)	4	49·6 (41·3–59·1)	11	34·9 (30·5–41·0)	17	63·8 (56–73·9)	14	89·6 (74·4–107·0)	10
Epilepsy	211·5 (163·7–268·9)	6	185·1 (137·0–261·7)	3	174·0 (134·1–219·5)	4	176·9 (125·9–247·7)	7	268·5 (186·4–379·7)	5
Fetal alcohol syndrome	0·4 (0·2–0·7)	27	0·4 (0·2–0·6)	32	0·3 (0·2–0·4)	34	0·1 (0·1–0·2)	33	0·1 (0·1–0·2)	30
Guillain–Barré syndrome	0·4 (0·2–0·7)	28	0·5 (0·2–0·8)	31	0·6 (0·3–0·9)	33	1·2 (0·7–2·0)	28	1·4 (0·8–2·2)	25
Idiopathic intellectual disability	68·3 (32·4–118·0)	11	70·4 (33·7–118·7)	7	43·1 (19·4–74·0)	14	17·1 (7·2–30·4)	21	9·8 (4·9–16·7)	21
Klinefelter syndrome*	0·1 (0·1–0·2)	29	0·0 (0·0–0·1)	33	0·0 (0·0–0·0)	36	0·0 (0·0–0·0)	36	0·0 (0·0–0·0)	34
Meningitis	1234·9 (869·5–1741·7)	2	130·4 (111·0–161·3)	5	72·4 (65·0–83·4)	11	69·8 (64·2–77·9)	13	84·1 (75·1–92·4)	11
Migraine	0·0 (0·0–0·0)	NA	380·0 (24·7–946·6)	1	750·8 (117·3–1617·8)	2	451·6 (112·3–953·6)	4	238·3 (65·8–499·5)	6
Motor neuron disease	6·2 (4·9–7·7)	19	1·3 (1·0–1·5)	28	8·7 (8·0–9·6)	24	57·3 (53·2–61·9)	15	48·2 (39·8–54·1)	16
Multiple sclerosis	0·0 (0·0–0·0)	NA	0·5 (0·4–0·7)	30	15·1 (12·7–17·8)	22	31·8 (28·1–35·8)	19	24·4 (20·7–28·3)	18
Neonatal encephalopathy	8316·7 (7072·6–9991·8)	1	64·9 (46·3–85·6)	8	55·4 (40·1–72·0)	12	25·8 (18·8–33·0)	20	2·1 (1·3–3·0)	23
Neonatal jaundice*	13·2 (9·5–16·8)	15	11·6 (8·4–14·8)	21	8·7 (6·3–11·2)	25	3·9 (2·8–5·0)	26	0·4 (0·3–0·6)	28
Neonatal sepsis*	42·3 (27·1–61·1)	13	39·5 (24·7–57·1)	12	31·3 (19·6–45·2)	18	13·5 (8·3–19·3)	22	0·6 (0·3–0·9)	27
Nervous system cancer	94·9 (71·4–121·5)	10	60·2 (50·3–71·7)	10	106·6 (91·2–125·6)	7	280·0 (245·7–316·1)	6	208·8 (174·3–232·0)	8
Neural tube defects	722·6 (580·8–899·4)	3	17·0 (12·5–23·6)	17	4·5 (3·6–5·9)	29	1·6 (1·2–2·1)	27	1·3 (0·9–1·8)	26
Neurocysticercosis	0·8 (0·1–1·9)	25	1·6 (0·8–2·5)	26	16·1 (9·1–23·8)	21	45·7 (24·8–76·2)	17	65·4 (36·1–105·0)	13
Neurosyphilis	0·7 (0·4–1·0)	26	1·1 (0·7–1·6)	29	0·8 (0·5–1·1)	32	0·6 (0·4–0·8)	31	0·4 (0·3–0·6)	29
Chromosomal anomalies*	12·2 (8·1–17·3)	17	5·8 (3·9–8·2)	24	2·3 (1·5–3·3)	30	0·4 (0·2–0·5)	32	0·0 (0·0–0·0)	36
Other neurological disorders	44·6 (36·2–54·4)	12	60·2 (44·2–84·0)	9	41·8 (34·6–49·6)	15	94·7 (84·0–106·8)	11	157·8 (133·9–176·9)	9
Parkinson's disease	0·0 (0·0–0·0)	NA	0·0 (0·0–0·0)	NA	16·2 (13·6–19·1)	20	430·7 (389·5–472·1)	5	1773·2 (1550·8–1925·0)	3
Preterm birth*	264·9 (190·4–344·9)	5	234·3 (168·4–305·2)	2	165·7 (120·5–212·9)	5	56·0 (41·9–71·4)	16	14·1 (9·6–20·9)	19
Rabies	12·8 (4·5–24·6)	16	12·3 (6·6–17·9)	19	4·9 (3·1–6·8)	28	3·9 (2·5–5·4)	25	1·6 (1·1–2·2)	24
Spinal cord injury	3·2 (2·3–4·2)	21	17·6 (11·9–23·5)	16	74·7 (52·3–99·2)	10	103·2 (74·5–132·7)	10	83·0 (57·6–113·0)	12
Stroke	147·5 (109·0–196·8)	8	72·9 (65·0–80·2)	6	1126·1 (1035·5–1218·4)	1	8490·9 (7827·5–9108·6)	1	20 336·1 (17 762·6–22 137·4)	1
Tension-type headache	0·0 (0·0–0·0)	NA	29·7 (4·8–148·1)	13	77·5 (23·3–238·7)	8	75·4 (23·3–224·1)	12	49·4 (12·2–165·8)	15
Tetanus	120·5 (51·4–218·6)	9	7·8 (3·5–13·8)	23	7·5 (3·3–12·2)	26	8·2 (3·7–12·8)	24	4·2 (1·5–7·2)	22
Traumatic brain injury	3·9 (2·6–5·3)	20	11·8 (8·3–16·0)	20	77·5 (54·2–103·7)	9	176·5 (126·5–236·6)	8	226·3 (160·7–298·5)	7

Rates are provided to one decimal place. Due to rounding, values for some rare conditions (eg, tetanus, rabies, neurocysticercosis, and congenital Zika syndrome) are shown as 0·0, but the actual values are higher than 0·0.

* Neurological complications related to this condition.

### Temporal and sex patterns

**Figure 2:**
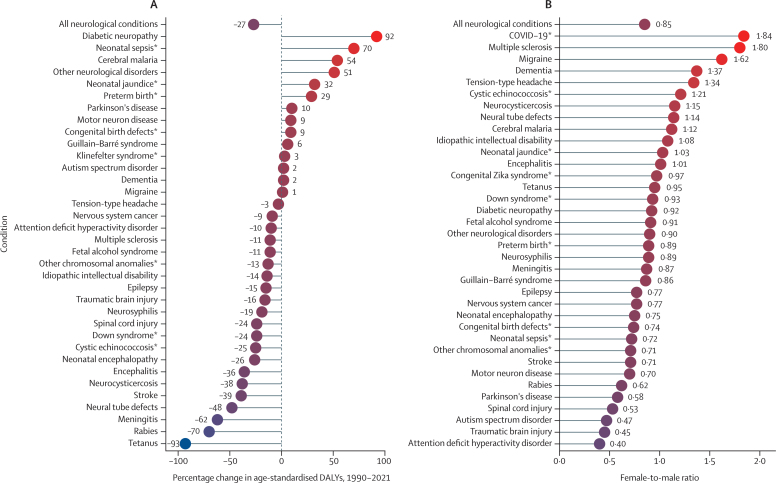
Change in age-standardised DALYs, 1990–2021, and female-to-male ratios in 2021 for each condition (A) Percentage change from 1990 to 2021 overall and for individual conditions. Colours range from dark blue (largest decrease in age-standardised DALYs) to bright red (largest increases). Cognitive impairment due to COVID-19 and congenital Zika virus disease are not included because they were not documented until after 1990. (B) Female-to-male ratio in 2021, where values higher than 1 indicate higher levels in females (log scale). Colours range from blue (smaller female-to-male ratios) to red (larger female-to-male ratios). Throughout the figure, dementia represents Alzheimer's disease and other dementias. DALYs=disability-adjusted life-years. *Percentage change and female-to-male ratios are isolated to disease DALYs due to neurological complications as opposed to DALYs attributed to the entire condition.

Temporal trends between 1990 and 2021 in age-standardised DALYs for individual conditions varied from a maximum increase of 91·9% (95% UI 86·3–97·3) for diabetic neuropathy to a maximum decrease for tetanus of 92·6% (88·0–94·9l figure 2). Six conditions had a 25·0% or larger increase in age-standardised DALYs from 1990 to 2021: diabetic neuropathy, neurological complications due to neonatal sepsis, cerebral malaria, other neurological disorders, neurological complications due to neonatal jaundice, and neurological complications due to preterm birth. Eight conditions had a 25·0% or larger decrease in DALYs in this time period: tetanus, rabies, meningitis, neural tube defects, stroke, neurocysticercosis, encephalitis, and neonatal encephalopathy. Congenital Zika syndrome and COVID-19 did not exist in 1990. In 2021, neurological complications due to COVID-19 was the 20th ranked contributor to age-standardised global neurological DALYs (figure 1). Of the 41·5 million (22·1–89·4) cases of neurological health loss with infectious causes, 23·4 million (4·14–72·8) were cases of COVID-19 with long-term cognitive symptoms or Guillain–Barré syndrome. These COVID-19 cases contributed 2·48 million (0·0872–7·99) DALYs.

**Figure 3:**
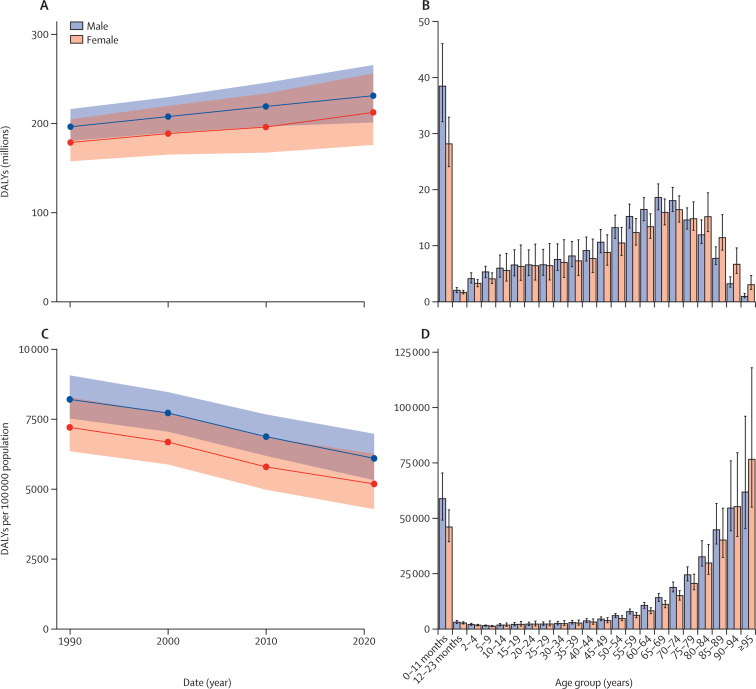
Temporal and age patterns in global DALYs for all neurological conditions combined DALY counts are shown over time (A) and by age group in 2021 (B). Age-standardised DALYs per 100 000 population are shown over time (C) and age-specific rates are shown in 2021 (D). Shading in panels A and C and black bars in panels B and D depict 95% uncertainty intervals. DALYs=disability-adjusted life-years.

In 2021, rates of age-standardised DALYs for the total neurological category were lower in females (5185·8 [95% UI 4281·2–6262·9] per 100 000 people) than in males (6101·0 [5320·2–6982·7] per 100 000 people; figure 3; table 2), with a female-to-male ratio of 0·85 (0·78–0·93; figure 2). Age-specific rates show similar or higher DALY burden in males than in females in most age groups, except for ages 90–94 years and 95 years and older (figure 3). The three conditions with the largest female-to-male ratios were cognitive impairment or Guillain–Barré syndrome due to COVID-19 (1·84, 0·97–3·35), multiple sclerosis (1·80, 1·72–1·87), and migraine (1·62, 1·39–1·79). Conditions with the smallest female-to-male ratios were attention deficit hyperactivity disorder (0·40, 0·38–0·43), traumatic brain injury (0·45, 0·43–0·46), and autism spectrum disorder (0·47, 0·46–0·49).

### Risk factor contribution to neurological condition burden

18 risk factors were quantified for stroke, four for encephalitis and meningitis, three for Alzheimer's disease and other dementias, and one each for multiple sclerosis, Parkinson's disease, idiopathic epilepsy, and idiopathic intellectual disability (appendix p 80). Stroke DALYs had the largest attributable burden globally (84·2%, 95% UI 78·2–88·8). The risk factor with the largest all-age population attributable fraction for stroke was high systolic blood pressure (57·3%, 42·7–68·4), and that for Alzheimer's disease and other dementias was high fasting plasma glucose (14·6%, 1·2–29·4). Smoking was associated with increased DALYs from stroke, Alzheimer's disease and other dementias, and multiple sclerosis. The proportion of DALYs associated with idiopathic epilepsy that was attributable to high alcohol use was almost four times higher in males (10·8%, 7·8–13·8) than in females (2·9%, 1·8–4·0). Ambient particulate matter pollution accounted for 16·7% (11·6–21·0) stroke risk, and household air pollution accounted for 11·3% (6·5–19·5) of stroke risk. The largest attributable fraction was for lead exposure, which accounted for 63·1% (33·3–81·1) of DALYs associated with idiopathic intellectual disability burden.

## Discussion

In 2021, 3·40 billion individuals had nervous system health loss and 11·1 million individuals died from a nervous system condition. This nervous system category was the leading cause of DALYs and YLLs, emphasising the enormous public health impact of these conditions.

This study builds on previous efforts^1^, ^4^ and provides the most comprehensive estimate of neurological burden globally. The original grouping of conditions published for GBD 2015^4^ and GBD 2016^1^ contributed 313 million DALYs, or 70·5% of the total neurological DALYs reported here for 2021. We added conditions affecting the nervous system that were previously not captured, such as Guillain–Barré syndrome, neurocysticercosis, rabies, and neuroblastoma and other peripheral nervous cell tumours (captured under the nervous system cancer category), which together contributed 2·14 million DALYs, or 0·5% of the total. To improve reflection of the life course in line with the new IGAP,^3^ we included neurodevelopmental disorders and paediatric neurological conditions, which represent a large proportion of global disease burden due to premature death and long-lasting disability,^7^ and together these conditions contributed 80·3 million DALYs or 18·2% of the total DALYs attributable to neurological health loss. Cerebral palsy was not listed separately among the 37 included conditions because the relevant GBD sequelae are captured under their causes, for example preterm birth and neonatal encephalopathy. We also quantified the disease burden of conditions that include neurological complications by extracting and accounting for neurological sequelae from a heterogeneous group of conditions with nervous system health loss, together contributing approximately 48·1 million DALYs or 10·8% of the total DALYs attributable to neurological health loss.

Nervous system health loss disproportionately affected people in LMICs, partly due to higher prevalence of conditions affecting neonates and children younger than 5 years (especially birth-related complications and infections). Increased survival in neonates is unfortunately accompanied by an increase in the long-term disability from neurological complications of these conditions.^18^, ^19^, ^20^ Access to treatment and rehabilitation services for nervous system conditions is limited by little availability or existence of specialised services and workforces, contributing to poor health outcomes and high mortality. For instance, the high proportion of deaths in LMICs compared with high-income countries is probably related to poorer access to high-quality services and nervous system specialists in LMICs.^21^ We showed that YLDs are more consistent between geographical regions than YLLs (appendix p 79). However, this similarity could partly be because sources directly reporting disease severity for highly disabling outcomes are sparse, and therefore we were unable to take into account potential effects of treatment on severity.

Nervous system conditions contribute to more DALYs in males but higher prevalence in females (mostly attributable to migraine and tension-type headache), and disease burden generally increases with age. Different age patterns emerged for different conditions, including differences in the relative contribution of YLLs and YLDs to total burden, emphasising the need for tailored interventions and prevention strategies across the lifespan.

In line with previous GBD findings,^1^ the absolute number of people living with, or dying from, neurological conditions increased over the past three decades. This trend mirrors global demographic and ageing trends and increased exposure to environmental, metabolic, and lifestyle risk factors that are especially relevant for non-communicable neurological conditions, such as stroke and dementia.^22^, ^23^ At the same time, the age-standardised death, DALY, and YLL rates of total neurological conditions decreased. Improved public awareness of stroke, use of statins,^24^ and blood pressure lowering medications^25^ have probably contributed to the decreased DALYs associated with stroke, as well as, primarily in high-income countries, the approval of intravenous thrombolytics in the mid-1990s, the increasing availability of endovascular thrombectomy for acute stroke, and growth of comprehensive stroke units.^26^, ^27^, ^28^ Observed reductions in DALYs were also probably driven by global vaccination and disease-prevention efforts, especially for tetanus, rabies, meningitis, neurocysticercosis, and encephalitis,^29^ and improved access to prevention and treatment. For instance, we estimate an 17·8% reduction in encephalitis DALYs, which could partly reflect Japanese encephalitis vaccination and improved access to health care.^30^ The substantial decrease in global deaths from tetanus because of vaccination strategies emphasises the profound value of vaccination prevention campaigns. Lastly, the promotion and enforcement of folic acid supplementation and fortification of grain products contributed to the decreases in incidence of neural tube defects in countries that have instituted this basic public health initiative.^31^, ^32^, ^33^

The estimate of Guillain–Barré syndrome due to COVID-19 was generated through an analysis of US data from a large sample of approximately 2 million individuals, a finding supported by some other studies^34^ but requiring further research, because other studies showed no effect of COVID-19 on Guillain–Barré syndrome.^35^, ^36^ A more detailed description of methods used in this analysis to estimate Guillain–Barré syndrome following COVID-19, including strengths and limitations, is in the appendix (p 2). Parkinson's disease was previously identified as the fastest growing neurological condition,^4^, ^37^ but through the inclusion of additional cause categories and sequelae, other conditions—ie, primarily diabetic neuropathy, neonatal conditions with neurological consequences, and cerebral malaria—are now ranking higher in terms of percentage growth of age-standardised DALYs since 1990 (table 1). Diabetic neuropathy is now the fifth-ranked cause of global age-standardised DALYs due to neurological conditions and its fast-increasing burden is in line with the observed increase in the global prevalence of diabetes,^38^ particularly type 2 diabetes, which constitutes 96% of all diabetic cases and is a preventable condition. The fast-increasing burden from diabetes, neonatal conditions, and cerebral malaria, which are all largely preventable, indicates poor implementation of effective risk reduction strategies for these health conditions.

Because cures do not exist for many neurological conditions, and because access to quality medical care can be limited by workforce constraints in some places,^39^ a comprehensive understanding of modifiable risk factors and the potentially avoidable burden of the neurological conditions they affect is key. This study extends previous estimates of risk factors for neurological disorders that focused on stroke, Alzheimer's disease and other dementias, and idiopathic epilepsy by additionally quantifying risk for multiple sclerosis, Parkinson's disease, encephalitis, meningitis, and idiopathic intellectual disability. Risk factors for stroke are the most comprehensively studied of all neurological conditions in GBD, and in 2021, 84·2% of stroke DALYs were potentially preventable through the reduction of exposure to 18 identified risk factors. Controlling lead exposure could reduce the burden of idiopathic intellectual disability by 63·1%, and reducing high fasting plasma glucose to typical concentrations (ie, 4·88–5·30 mmol/L^38^) could reduce the burden of Alzheimer's disease and other dementias by 14·6%. Smoking was identified as a risk factor for stroke,^22^, ^40^ Alzheimer's disease and other dementias,^41^ and multiple sclerosis. However, some established risk–outcome pairs (eg, hearing loss and Alzheimer's disease and other dementias;^41^ exposure to pesticides and Parkinson's disease) were not assessed in our model. Psychosocial factors, such as sleep, stress, and social isolation, are increasingly recognised as contributors to neurological conditions and should be quantified in future analyses.

This study has limitations. Although we attempted to capture all nervous system health loss modelled in GBD explicitly or in the broad category of other neurological disorders, some conditions that cause nervous system damage were left out because we could not isolate the neurological component (eg, genetic conditions such as adrenoleukodystrophy or infections such as HIV, which have large effects in many parts of the world and should be explicitly estimated in the future; appendix p 49) or because they are not quantified in GBD (eg, poliomyelitis). Diabetic neuropathy has now been included within the neurological conditions category and its high ranking indicates a considerable burden within the category that has not previously been conveyed or emphasised. The inclusion of diabetic neuropathy calls attention to the scarcity of data for other peripheral neuropathies, such as those associated with alcohol, HIV, and vitamin deficiencies. Thus, our singular representation of diabetic neuropathy in this study is probably a gross underestimation of the total peripheral neuropathy burden.

Neurodevelopmental disorders that are captured in GBD under mental health, such as attention deficit hyperactivity disorder, autism spectrum disorder, and idiopathic intellectual disability, have been added because of their strong links to, and effects on, the nervous system, and because of their ICD-11 classification as neurodevelopmental disorders. Other mental health conditions that were not classified as neurodevelopmental or neurocognitive were excluded. Neurological complications of non-neurological diseases have been added as far as possible. We also assumed independence of disabling sequelae when making comorbidity corrections, which would lead to some overestimation of the non-fatal burden. Future analyses should include an improved evaluation of comorbidities that does not assume independency of co-occurrence. Further, we do not assess comorbidity with other chronic conditions, such as heart disease, that can compound effects on quality of life.

The residual category for other neurological disorders ranked 16th for age-standardised DALYs, emphasising the need to explicitly estimate DALYS for the conditions within this category, including specific movement disorders, myopathies, and non-traumatic spinal cord diseases. For non-fatal estimates of the residual category, we approximated YLDs by assuming the same ratio of YLDs and YLLs that we estimated for the main fatal neurological disorders. This approach, although a reasonable approximation for residual neurological conditions that lead to death, does not capture the burden of any residual neurological conditions that are not a cause of death, such as peripheral neuropathies and neuropathic pain.^42^, ^43^ Some neurological pain quantified in GBD is captured within musculoskeletal conditions—eg, low back pain (including sciatica) or neck pain—and such pain was not included in our current analysis. In GBD 2021, these conditions are defined as pain lasting at least 1 day, making it impossible to distinguish between chronic neurological pain and shorter-term musculoskeletal pain. Our analysis also did not account for deaths associated with traumatic brain injury and spinal cord injury. Within GBD and the ICD, the underlying cause of death is assigned to the cause of injury (eg, violence or road traffic accident) rather than the nature of the injury.

When quantifying the neurological burden of post-COVID-19 condition, we accounted only for cognitive impairment, using the same disability weights as for mild or moderate dementia, which might not be accurate considering that cognitive impairment following COVID-19 appears to improve with time. As we did not have the resources to conduct disability weight surveys, such an approximation was necessary. We were also unable to analyse non-cognitive long-term neurological outcomes of COVID-19 (with the exception of Guillain–Barré syndrome) due to a dearth of such data for most countries. Some studies have shown that the prevalence of other neurological complications (eg, persistent headache, neuralgia, daytime sleepiness, and stroke) associated with COVID-19 varies widely.^44^, ^45^, ^46^ We also acknowledge that this analysis was conducted in the first 2 years after the emergence of COVID-19, and longer-term consequences on cognition or other aspects of nervous system health are not fully quantified.

Our study also has general limitations shared by all GBD studies, such as the scarcity of reliable epidemiological data in many countries, especially in LMICs, meaning that country estimates are informed by predictive covariates and data from surrounding countries, and our results are also affected by diagnostic and other biases in the original research. Efforts were made to correct for non-reference case definitions or measurement methods used in GBD source data. Adjusting for measurement bias is a strength of GBD because it allows us to leverage as many data sources as possible across time and geographies. This process is constantly being refined to enhance quality, transparency, and standardisation. However, some standard GBD reference definitions differ from other disease definitions, such as those used in the new ICD-11. Finally, because this is a global analysis, we could not capture the nuances of how these conditions affect specific geographies.

In summary, acute and chronic conditions affecting the nervous system are diverse. These conditions include infectious or vector-borne diseases, non-communicable diseases, and injuries, necessitating different public health strategies for prevention and treatment across the life course. These distinctions emphasise the complexity that health-care systems are facing and the need to balance acute and long-term disease management. For some conditions and disorders, effective treatments are available, whereas for others there is no cure, underscoring the importance of prevention and research into novel interventions.

Resources for nervous system conditions should therefore span the entire care continuum, including prevention, diagnosis, treatment, rehabilitation, long-term care, and palliation. Improved population-level preventive strategies for neurological infections and birth-related neurological sequelae can affect many lives; such strategies include appropriate sanitation, vaccination, folic acid fortification, improved access to prenatal and perinatal care and education, and early detection and treatment of birth defects. Additionally, zoonotic diseases, such as COVID-19 or Zika virus disease, have increasingly posed challenges to brain health,^8^, ^47^, ^48^ emphasising the importance of close collaboration across sectors and integration of human, animal, social, political, and environmental sciences in joint efforts to optimise brain health.^2^, ^47^, ^49^

Most nervous system burden occurs in LMICs, where health-care resources are scarce, access to services is limited by availability and workforce constraints, and many countries are insufficiently prepared to address the increasing number of cases or DALYs.^39^ In 2017, only 46 (24%) of 194 countries had policies with a separate budget dedicated to neurological disorders, and only 69 (55%) of 125 countries who responded to a questionnaire had clinical guidelines for neurological disorders (ie, systematically developed statements or recommendations designed to assist practitioners and patients in making decisions about appropriate health care for neurological disorders). The available neurological workforce is unevenly distributed across the world, with high-income countries having 70 times more neurological professionals per 100 000 individuals in the population compared with LMICs.^39^ The adoption of IGAP provides a guideline for a decade of concerted multisectoral actions to respond to the growing absolute burden of neurological conditions.^2^, ^3^

The findings in this study have important health service and policy implications and serve as evidence that global neurological health loss has been under-recognised and is increasing and unevenly distributed geographically and socioeconomically. Elucidating the individual contributions of conditions that affect nervous system health will inform targeted interventions and policy options that can increase nervous system health at individual and population levels.

**This online publication has been corrected. The corrected version first appeared at thelancet.com/neurology on March 18, 2024**

## Data sharing

The findings of this study are supported by data available in public online repositories, data publicly available on request of the data provider, and data not publicly available due to restrictions by the data provider. Non-publicly available data were used under license for the current study but might be available from the authors of this study on reasonable request to the corresponding author and with permission of the data provider.

## Declaration of interests

V Aboyans reports consulting fees from Bayer Healthcare, Amarin, Boehringer Ingelheim, and NovoNordisk; payment or honoraria for lectures, presentations, speakers bureaus, manuscript writing, or educational events from NovoNordisk and Amarin; and unpaid leadership or fiduciary roles in board, society, committee, or advocacy groups with the European Society of Cardiology and the French Society of Cardiology, all outside the submitted work. S Afzal reports payment or honoraria from educational events and webinars with King Edward Medical University and collaborative partners, including University of Johns Hopkins, University of California, and University of Massachusetts; participation on a data safety monitoring board or advisory board with National Bioethics Committee Pakistan, King Edward Medical University Institutional Ethical Review Board, Ethical Review Board Fatima Jinnah Medical University, and Sir Ganga Ram Hospital; leadership or fiduciary roles in board, society, committee, or advocacy groups, paid or unpaid with Pakistan Association of Medical Editors, the Faculty of Public Health Royal Colleges UK (Fellowship of Faculty of Public Health) as a fellow, the Society of Prevention, Advocacy And Research, King Edward Medical University as a member, and with the Pakistan Society of Infectious Diseases, outside the submitted work. K Akinosoglou reports payment or honoraria for lectures, presentations, speakers bureaus, manuscript writing, or educational events paid to the University of Patras from Pfizer Hellas, MSD, Gilead, ViiV/GSK, 3M, and Sobi and support for meeting and travel registration and accommodation costs from Pfizer Hellas, MSD, Gilead, Normal Hellas, and LEO Pharmaceuticals Hellas, outside the submitted work. R Ancuceanu reports consulting fees from Abbvie and payment or honoraria for lectures, presentations, speakers bureaus, manuscript writing, or educational events from Abbvie, Sandoz, B Braun, and Laropharm, outside the submitted work. P Atorkey reports support for the present manuscript from the Australian College of Applied Professions, Discipline of Psychological Sciences and The University of Newcastle, School of Medicine and Public Health. J Ärnlöv reports payment for lectures, presentations, speakers bureaus, manuscript writing, or educational events from AstraZeneca and Novartis and participation on an advisory board with AstraZeneca, Boerhinger Ingelheim, and Astella, outside the submitted work. R Bai reports support for the present manuscript from the National Natural Science Foundation of China (grant number 72204112), the Social Science Fund of Jiangsu Province (grant number 21GLD008), and the Fundamental Research Funds for the Central Universities (grant number 30923011101). M A Barboza reports payment or honoraria for lectures, presentations, speakers bureaus, manuscript writing, or educational events from Pfizer and Roche Pharmaceuticals and support for attending meetings and travel from Boehringer Ingelheim, outside the submitted work. T W Bärnighausen reports grants from the EU (Horizon 2020 and European Institute of Innovation and Technology Health), German Research Foundation, US National Institutes of Health, German Ministry of Education and Research, Alexander von Humboldt Foundation, Else-Kröner-Fresenius-Foundation, Wellcome Trust, Gates Foundation, KfW Development Bank, UNAIDS, and WHO; consulting fees for KfW on the OSCAR Initiative in Viet Nam; participation on a data safety monitoring board or advisory board with NIH-funded study Healthy Options as chair of the data safety and monitoring board, participation on a data safety monitoring board with the German National Committee on the Future of Public Health Research and Education, participation as chair of the scientific advisory board to the European and Developing Countries Clinical Trials Partnership Evaluation, and participation as a member of the UNAIDS Evaluation Expert Advisory Committee, National Institutes of Health Study Section Member on Population and Public Health Approaches to HIV/AIDS, US National Academies of Sciences, Engineering, and Medicine's Committee for the Evaluation of Human Resources for Health in the Republic of Rwanda under the President's Emergency Plan for AIDS Relief, and University of Pennsylvania (UPenn) Population Aging Research Center (PARC) External Advisory Board; and leadership or fiduciary roles in board, society, committee or advocacy groups, paid or unpaid with the Global Health Hub Germany as co-chair (which was initiated by the German Ministry of Health), outside the submitted work. S Bhaskar reports grants or contracts from Japan Society for Promotion Science; leadership or fiduciary roles in board, society, committee, or advocacy groups, paid or unpaid with Rotary District 9675 Diversity, Equity and Inclusion as a chair and with Global Health and Migration, Global Health Hub Germany as a founding member, manager, and chair. H Carabin reports grants or contracts from WHO. M Endres reports grants from Bayer (unrestricted grant to Charité for MonDAFIS study and Berlin AFib registry, with no personal fees); consulting fees paid to the institution from Bayer; payment (paid to institution) for honoraria for lectures, presentations, speakers bureaus, manuscript writing, or educational events from Bayer, Pfizer, Amgen, GSK, and Novartis; participation on a data safety monitoring board or advisory board (no personal fees) with BMS (country principal investigator for Axiomatic-SSP), Bayer (country principal investigator for NAVIGATE-ESUS), and Daiichi Sankyo; leadership or fiduciary roles in board, society, committee, or advocacy groups, paid or unpaid with the European Academy of Neurology as a member at large on the board of directors and as an unpaid fellow, the German Center for Neurodegenerative Diseases as an unpaid member, the International Society for Cerebral Blood Flow and Metabolism as an unpaid member, the American Health Association and American Stroke Association as an unpaid member, the European Stroke Organisation as an unpaid fellow, the World Stroke Organization as an unpaid member, German Centre of Cardiovascular Research as an unpaid principal investigator, and German Center of Neurodegenerative Diseases as a paid principal investigator under a personal contract; and receipt of equipment, materials, drugs, medical writing, gifts, or other services from Amgen, outside the submitted work. L M Force reports support for the present manuscript from the Gates Foundation; grants or contracts from Conquer Cancer Foundation, St Jude Children's Research Hospital, St Baldrick's Foundation, and National Institutes of Health Loan Repayment Program; and leadership or fiduciary roles in board, society, committee, or advocacy groups, unpaid with the Lancet Oncology International Advisory Board. Q Gan reports other financial or non-financial interests from the International Agency for Research on Cancer as the beneficiary of the International Agency for Research on Cancer Research and Training Programme. J F Mosser reports grant funding support for the present manuscript for Global Burden of Disease estimation from the Gates Foundation; grants from Gavi, the Vaccine Alliance; and support for attending meetings and travel from the Gates Foundation. S Muthu support for attending meetings and travel from ON Foundation (International Cartilage Regeneration and Joint Preservation Society 2023) and leadership or fiduciary roles in board, society, committee, or advocacy groups, paid or unpaid with the International Society of Orthopaedic Surgery and Traumatology on the Research Grants Committee and the International Cartilage Regeneration and Joint Preservation Society on the NextGEN Committee, outside the submitted work. L Ronfani reports support for the present manuscript from the Italian Ministry of Health (Ricerca Corrente 34/2017), payments made to the Institute for Maternal and Child Health Istituto di Ricovero e Cura a Carattere Scientifico Burlo Garofolo. S Zadey reports honoraria for lectures, presentations, speakers bureaus, manuscript writing, or educational events from Think Global Health, Harvard Public Health Magazine, and The Wire Science and leadership or fiduciary roles in board, society, committee, or advocacy groups, paid or unpaid with the Association for Socially Applicable Research as a co-founding director, The G4 Alliance as a permanent council member, Surgical, Obstetric, Trauma and Anesthesia Care in South Asia Working Group as a chair, and Maharashtra State Mental Health Policy as a drafting committee member, outside the submitted work. The authors alone are responsible for the views expressed in this Article, and they do not necessarily represent the views, decisions, or policies of the institutions or funders with which they are affiliated.
