# Correction to *Lancet Neurol* 2024; 23: 344–81

**DOI:** 10.1016/S1474-4422(24)00114-5

**Published:** 2024-05

*GBD 2021 Nervous System Disorders Collaborators. Global, regional, and national burden of disorders affecting the nervous system, 1990–2021: a systematic analysis for the Global Burden of Disease Study 2021.* Lancet Neurol *2024; **23:** 344–81*—In this Article, the dates on the x axis for figure 3A and 3C have been corrected. This correction has been made as of March 18, 2024.

