# Supplementary material for: Global, regional, and national burden of disorders affecting the nervous system, 1990–2021: a systematic analysis for the Global Burden of Disease Study 2021
Source: Lancet Neurol. 2024 Apr;23(4):344–81. doi: 10.1016/S1474-4422(24)00038-3 (PMC10949203; doi:10.1016/S1474-4422(24)00038-3)
Supplement: Supplementary appendix [file mmc1.pdf]

THE LANCET
Neurology

Supplementary appendix

This appendix formed part of the original submission and has been peer reviewed. We post it as supplied by the authors.

Supplement to: GBD 2021 Nervous System Disorders Collaborators. Global, regional, and national burden of disorders affecting the nervous system, 1990–2021: a systematic analysis for the Global Burden of Disease Study 2021. *Lancet Neurol* 2024; published online Mar 14. https://doi.org/10.1016/S1474-4422(24)00038-3.

Supplemental materials

Global, regional, and national burden of disorders affecting the nervous system, 1990–2021: a systematic analysis of the Global Burden of Disease Study 2021

Contents

Supplemental methods 2

Statement of GATHER compliance 2

Supplemental methods table 1. GATHER checklist........ 2

Abbreviations 5

Supplemental methods table 2. Neurological features of conditions that impact multiple systems. 5

Supplemental methods table 3. Conditions in other neurological disorders category 5

Supplemental methods table 4. Input data to non-fatal models and most recent dates for data seeking 6

Case definitions and adjustments........ 8

Supplemental methods table 5. Summary of reference and alternative case definitions 8

Supplemental methods table 6. ICD-9 and ICD-10 non-fatal coding for each condition* 14

Summary of Bayesian meta-regression methods 16

Supplemental methods table 7. Country-level predictive covariates in non-fatal models 16

Supplemental methods table 8. Global Burden of Disease geographical hierarchy 18

Accounting for transiency of some neurological conditions: two examples 20

Supplemental methods table 9. Sequelae included in analysis 21

Neurological outcomes of HIV 49

Supplemental methods table 10. Disability weights used to calculate YLDs........ 49

Analysis of Guillain-Barré Syndrome due to COVID-19........ 62

Data types and modelling methods to estimate deaths........ 62

Supplemental methods table 11. Source counts in fatal models 62

Supplemental methods table 12. ICD-9 and ICD-10 fatal coding for each condition 63

Common ICD-10 vital registration codes for the residual “other neurological disorders” category globally in 2021 64

Supplemental methods table 13. Predictive covariates included in fatal models........ 65

Supplemental results 67

Supplemental results figure 1. Proportion of total DALYs attributable to different categories of conditions........ 67

Supplemental results figure 2. Age and sex trends in YLDs in 2021 due to neurological conditions 68

Supplemental results figure 3. Age and sex trends in YLLs in 2021 due to neurological conditions...... 69
Supplemental results table 1. Global, regional, and national age-standardised prevalence, years lived with disability, deaths, years of life lost, and disability-adjusted life-years (per 100 000) for all neurological conditions........ 70
Supplemental results figure 4. Age-standardised rate of YLDs and YLLs for all neurological health loss by region in 2021. 79
Supplemental results table 2. Global, all-age population attributable fractions for risk and neurological outcome pairs 80
References 81
Global Burden of Disease 2021 Nervous System Disorders Collaborators' contributions 81

Supplemental methods

Statement of GATHER compliance

This study complies with the Guidelines for Accurate and Transparent Health Estimates Reporting (GATHER) recommendations. Below is the GATHER checklist.[2]

Supplemental methods table 1. GATHER checklist

#	GATHER checklist item	Description of compliance	Reference
**Objectives and funding**			
**1**	Define the indicator(s), populations (including age, sex, and geographic entities), and time period(s) for which estimates were made.	Narrative provided in paper and appendix describing indicators, definitions, populations, and time periods	Main text (Methods) and Appendix (Supplemental Methods)
**2**	List the funding sources for the work.	Funding sources listed in paper	Abstract (Funding)
**Data Inputs**			
For all data inputs from multiple sources that are synthesized as part of the study:			
**3**	Describe how the data were identified and how the data were accessed.	Narrative description of data seeking methods provided	Main text (Methods) and Appendix (Supplemental Methods)
**4**	Specify the inclusion and exclusion criteria. Identify all ad-hoc exclusions.	Narrative about inclusion and exclusion criteria provided; ad hoc exclusions	Main text (Methods and Discussion) and Appendix (Supplemental Methods)

		in appendix supplementary methods	
**5**	Provide information on all included data sources and their main characteristics. For each data source used, report reference information or contact name/institution, population represented, data collection method, year(s) of data collection, sex and age range, diagnostic criteria or measurement method, and sample size, as relevant.	An interactive, online data source tool that provides metadata for data sources by component, geography, cause, risk, or impairment has been developed, and data source citations provided	Appendix (Supplemental Methods) with additional information about these sources available at https://ghdx.healthdata.org/
**6**	Identify and describe any categories of input data that have potentially important biases (e.g., based on characteristics listed in item 5).	Summary of known biases provided	Main Text (Discussion)
For data inputs that contribute to the analysis but were not synthesized as part of the study:			
**7**	Describe and give sources for any other data inputs.	Included in online data source tool	Global Health Data Exchange (https://ghdx.healthdata.org/)
For all data inputs:			
**8**	Provide all data inputs in a file format from which data can be efficiently extracted (e.g., a spreadsheet rather than a PDF), including all relevant meta-data listed in item 5. For any data inputs that cannot be shared because of ethical or legal reasons, such as third-party ownership, provide a contact name or the name of the institution that retains the right to the data.	Downloads of input data available through online data tools; input data not available in tools will be made available upon request	Global Health Data Exchange (https://ghdx.healthdata.org/)
**Data analysis**			
**9**	Provide a conceptual overview of the data analysis method. A diagram may be helpful.	Summary of modelling process provided	Main text (Methods) and Appendix (Supplemental Methods)

**10**	Provide a detailed description of all steps of the analysis, including mathematical formulae. This description should cover, as relevant, data cleaning, data pre-processing, data adjustments and weighting of data sources, and mathematical or statistical model(s).	Summary of data extraction, processing, and modelling processes provided	Main text (Methods) and Appendix (Supplemental Methods)
**11**	Describe how candidate models were evaluated and how the final model(s) were selected.	Summary of model evaluation provided	Main text (Methods) and Appendix (Supplemental Methods)
**12**	Provide the results of an evaluation of model performance, if done, as well as the results of any relevant sensitivity analysis.	N/A	N/A
**13**	Describe methods for calculating uncertainty of the estimates. State which sources of uncertainty were, and were not, accounted for in the uncertainty analysis.	Provided in main text methods narrative description and appendix methods	Main text (Methods) and Appendix (Supplemental Methods)
**14**	State how analytic or statistical source code used to generate estimates can be accessed.	Included in online data code tool	Global Health Data Exchange (https://ghdx.healthdata.org/)
**Results and Discussion**			
**15**	Provide published estimates in a file format from which data can be efficiently extracted.	Published estimates not available in main text or appendix will be made available upon request.	Main text (Methods, Results and Discussion) and Appendix (Supplemental Methods and Results)
**16**	Report a quantitative measure of the uncertainty of the estimates (e.g. uncertainty intervals).	Uncertainty provided with all results	Main text (Methods) and Appendix (Supplemental Results)
**17**	Interpret results in light of existing evidence. If updating a previous set of estimates, describe the reasons for changes in estimates.	Discussion of results and methodological changes between GBD rounds provided in manuscript narrative and appendix	Main text (Methods, Results and Discussion) and Appendix (Supplemental Methods and. Results)

**18**	Discuss limitations of the estimates. Include a discussion of any modelling assumptions or data limitations that affect interpretation of the estimates.	Discussion of limitations, including modelling assumptions and data limitations, included in manuscript narrative and appendix	Main text (Methods and Discussion) and Appendix (Supplemental Methods)

Abbreviations

Haemolytic disease and other neonatal jaundice = neonatal jaundice
Neonatal encephalopathy due to birth asphyxia and trauma = neonatal encephalopathy
Neonatal sepsis and other neonatal infections = neonatal sepsis

Supplemental methods table 2. Neurological features of conditions that impact multiple systems.

**Condition**	**Neurological features**
Congenital birth defects	Cognitive impairment, motor impairment, intellectual disability, hearing loss
COVID-19	Cognitive impairment, Guillain-Barré syndrome (due to COVID)
Diabetes	Diabetic neuropathy (including diabetic foot and amputation)
Down syndrome	Intellectual disability, dementia
Echinococcosis	Epilepsy
Klinefelter syndrome	Intellectual disability
Malaria	Long-term consequences of cerebral malaria: motor impairment, cognitive impairment, epilepsy, blindness
Neonatal jaundice	Long-term consequences: motor impairment, cognitive impairment, epilepsy, blindness
Neonatal sepsis	Long-term consequences: motor impairment, cognitive impairment, epilepsy, blindness
Neurocysticercosis	Epilepsy
Other chromosomal abnormalities	Intellectual disability, dementia
Preterm birth	Long-term consequences: motor impairment, cognitive impairment, epilepsy, blindness, retinopathy of prematurity
Syphilis	Adult and congenital neurosyphilis
Zika virus syndrome	Congenital (microcephaly)

Supplemental methods table 3. Conditions in other neurological disorders category

Broad category	Conditions*
Nervous system	ICD 9: other and unspecified disorders of the nervous system;
Brain	ICD-9: cerebral degenerations usually manifest in childhood; idiopathic normal pressure hydrocephalus; corticobasal degeneration; cerebral degeneration in diseases classified elsewhere; other cerebral degeneration or unspecified; other demyelinating diseases of central nervous system

	ICD-10: other demyelinating diseases of central nervous system; dementia in other diseases classified elsewhere; other degenerative diseases of basal ganglia; systemic atrophies primarily affecting central nervous system in diseases classified elsewhere
Movement disorders	ICD-10: Huntington's disease; other extrapyramidal and movement disorders; extrapyramidal and movement disorders in diseases classified elsewhere; other extrapyramidal disease and abnormal movement disorders; dystonia;
Spinocerebellar disease	ICD-9: spinocerebellar disease<br>ICD-10: hereditary ataxia
Diseases of the spinal cord	ICD-9: other diseases of spinal cord<br>ICD-10: other and unspecified diseases of spinal cord; spinal muscular atrophy and related syndromes
Autonomic nervous system	ICD-9: disorders of the autonomic nervous system<br>ICD-10: disorders of the autonomic nervous system
Nerve root/plexus disorders	ICD-9: other or unspecified nerve root and plexus disorders
Peripheral nerve disorders**	ICD-9: acute infective polyneuritis; polyneuropathy; hereditary and idiopathic peripheral neuropathy<br>ICD-10: inflammatory polyneuropathy
Neuromuscular disorders	ICD-9: myoneural disorders (eg, myasthenia gravis, Lambert-Eaton syndrome); neonatal myasthenia gravis<br>ICD-10: myasthenia gravis and other myoneural disorders; disorders of myoneural junction and muscle in diseases classified elsewhere
Muscle diseases	ICD-9: muscular dystrophies and other myopathies<br>ICD-10: primary disorders of muscles, dermatomyositis, other and unspecified myopathies

*Listed conditions correspond to three-digit ICD 9 and 10 coding unless four-digit is required to code to an included neurological category; see ICD table (supplemental methods table 11) for a granular list of codes included in the 'other neurological disorders' category; **includes fatal outcomes of Guillain-Barré syndrome

Supplemental methods table 4. Input data to non-fatal models and most recent dates for data seeking

Note that source count numbers are comprehensive for the condition, not just neurological sequelae unless otherwise specified.

Condition*	Prevalence	Incidence	Other	Countries with data	Most recent GBD round with new data added (clinical or literature)^
AD/HD	172	2	17	49	GBD 2019
Alzheimer's disease and other dementias	254	92	225	58	GBD 2021
Autism spectrum disorder	105	0	6	31	GBD 2021
Congenital birth defects	1746	0	188	105	GBD 2021
COVID-19 (long-term)	0	0	26	14	GBD 2021
Cystic echinococcosis	0	358	0	62	GBD 2021
Diabetes	920	214	85	172	GBD 2021

Down syndrome	1510	0	40	78	GBD 2021
Encephalitis	0	392	1	57	GBD 2021
Epilepsy	384	89	188	94	GBD 2021
Fetal alcohol syndrome	169	0	8	32	GBD 2015
Guillain-Barré syndrome	0	357	44	47	GBD 2021
Idiopathic intellectual disability	64	0	0	31	GBD 2016
Klinefelter syndrome	834	0	3	45	GBD 2021
Malaria	1846	4957	7530	121	GBD 2021
Meningitis	0	556	345	96	GBD 2021
Migraine	148	4	7	51	GBD 2017
Motor neuron disease	24	48	1	18	GBD 2019
Multiple sclerosis	211	86	29	53	GBD 2021
Neonatal encephalopathy	331	0	747	65	GBD 2021
Neonatal jaundice	62	1	1240	191	GBD 2021
Neonatal sepsis	0	0	349	57	GBD 2021
Nervous system cancers**	6	6230	641	101	GBD 2021
Neural tube defects	1566	0	13	90	GBD 2021
Neurocysticercosis	30	0	0	16	GBD 2015
Other chromosomal abnormalities	1330	0	23	71	GBD 2021
Other neurological disorders	N/A (death data only for this group)				GBD 2021 (death data)
Parkinson's disease	127	45	42	45	GBD 2021
Preterm birth	0	0	2187	176	GBD 2021
Rabies	N/A (death data only)				GBD 2021 (death data)
Spinal cord injury	N/A (nature of injury is calculated from causes of injury)				GBD 2021 (cause of injury data)
Stroke (ischaemic)	145	351	173	78	GBD 2021
Stroke (intracerebral haemorrhage)	143	359	144	76	GBD 2021
Stroke (subarachnoid haemorrhage)	131	288	102	63	GBD 2021
Syphilis	996	667	24	177	GBD 2021
Tension-type headache	94	0	6	39	GBD 2017
Tetanus	0	0	258	50	
Traumatic brain injury	N/A (nature of injury is calculated from causes of injury)				GBD 2021 (cause of injury data)
Zika virus disease	0	247	7	60	GBD 2021

*Source counts are for the entire condition and not just the neurological component unless otherwise specified; **nervous system cancers refers to the combination of "Brain and central nervous system cancer" and "Neuroblastoma and other peripheral nervous cell tumours"; ^conditions are updated on a rotating basis.

Case definitions and adjustments

Non-reference case definition data were adjusted to reference. Input data using different case definitions were matched by age, sex, location, and year; where possible, within-study comparisons or validation studies were used. Matched pairs were logit-transformed, and the logit difference was calculated and used as input data into a meta-regression tool called MR-BRT (meta-regression—Bayesian, regularised, trimmed; additional information can be found in appendix 1, section 4.4.1 of citation).[Vos et al.] Meta-regression results were used to systematically adjust non-reference data up or down to account for under- or over-counting, respectively.

Supplemental methods table 5. Summary of reference and alternative case definitions

**Condition**		**Definition**
AD/HD	Reference	An externalising disorder characterised by persistent inattention and/or hyperactivity-impulsivity. As per criteria set by the Diagnostic and Statistical Manual of Mental Disorders (DSM) Fourth Edition, Text Revision, diagnosis requires 6+ symptoms of inattention or hyperactivity-impulsivity to have persisted for at least six months in two or more settings causing significant impairment to functioning, with at least some impairing symptoms present prior to 7 years of age (12 years of age in DSM-5).
Alzheimer's disease and other dementias	Reference	A progressive, degenerative, and chronic neurological disorder typified by memory impairment and other neurological dysfunctions. Defined based on DSM III, IV or V, or ICD case criteria, including cognitive deficits that must include memory impairment, functional impairment, and gradual onset and continued decline.
	Alternatives	Diagnosis from clinical records, algorithm criteria, National Institute on Aging Alzheimer's disease criteria, 10/66 algorithm criteria, general practitioner records.
Autism spectrum disorder	Reference	A group of neurodevelopmental disorders with onset occurring in early childhood, characterised by pervasive impairment in several areas of development, including social interaction and communication skills, along with restricted and repetitive patterns of behaviours and/or interests, as defined by DSM-5 criteria or equivalent in International Classification of Diseases (ICD) or Chinese Classification of Mental Disorders (CCMD), and estimated from general population surveys with additional case-finding or total population screening.
	Alternatives	Study captures autism instead of autism spectrum disorder, study is a general population survey without additional case finding.
Congenital birth defects	Reference	For this analysis, this category includes congenital heart defects, other congenital anomalies, Edwards syndrome and Patau syndrome, and other chromosomal abnormalities that have neurological outcomes. The case definition of congenital

		anomalies includes any condition present at birth that is a result of abnormalities of embryonic development, excluding those directly the result of infections or substance abuse, and excludes minor anomalies as defined by European Surveillance of Congenital Anomalies (EUROCAT). Registries with the most complete list of reported case definitions (highest case ascertainment) were used as reference registries for each condition in the congenital birth defect category.
	Alternatives	Registries that did not have the highest level of case ascertainment.
Congenital Zika virus syndrome	Reference	Zika virus infection during pregnancy leading to fetal neurological symptoms such as microcephaly or decreased brain volume, and other congenital malformations. Cases identified from official reports primarily from the Pan American Health Organization (PAHO), which further describes criteria for maternal exposure and child phenotypes.[1]
COVID-19 (long)	Reference	A SARS-CoV-19 infection leading to new and persistent cognitive impairment that impacts everyday functioning and lasts at least three months after acute infection symptom onset.
	Alternatives	Outcome definition of cognitive impairment based on "memory problems".
Cystic echinococcosis	Reference	A parasitic disease caused by infection with the *Echinococcus granulosis* tapeworm that can spread to humans through ingestion of soil, water, or food contaminated with the faecal matter of an infected dog containing infective eggs. Diagnosis comes from clinical findings, imaging, serology, and tissue pathology.
Diabetic neuropathy	Reference	Diabetes: A chronic condition where either the pancreas does not produce enough insulin or the body is not able to metabolise insulin properly. This is defined as a fasting plasma glucose concentration ≥ to 7mmol/L, or use of insulin or diabetes medication. Neuropathy: People with diabetes mellitus who have diabetic neuropathy determined by microfilament test. Diabetic foot: People with diabetes mellitus who have diabetes foot, which is a poorly healing ulcer. Amputation: People with diabetes mellitus who have a lower limb amputation.
	Alternatives	Diabetes: Blood glucose tests other than reference (includes fasting plasma glucose threshold other than 7mmol/L, post-prandial glucose test, oral glucose tolerance test, glycated haemoglobin, and various cutoffs for each test) or USA and Taiwan insurance claims data. Neuropathy: Diagnosis determined with test other than microfilament. Amputation: Amputation of specific part of lower limb (e.g., toes only, feet only, below ankle only).
Down syndrome	Reference	Also known as Trisomy 21, is the presence of a third copy of chromosome 21, typically caused by nondisjunction during the production of gametes. Down syndrome is associated with

		several specific physical characteristics, including decreased muscle tone, flat facial features, an upward slant to the eyes, abnormally shaped ears, a single deep crease across the centre of the palm, folded skin on the inner corners of the eyes, and ability to extend joints beyond the usual, among others.
Encephalitis	Reference	A disease caused by acute inflammation of the brain, which can cause flu-like symptoms such as headaches, fever, drowsiness, fatigue, and at times, seizures, hallucinations, or stroke. Reference definition is based on ICD-10 criteria from inpatient data.
	Alternatives	Diagnosis from USA private claims data or epidemiological surveillance.
Epilepsy	Reference	A condition characterised by recurrent (two or more) epileptic seizures, unprovoked by any immediate identified cause. Active epilepsy is at least one epileptic seizure in the past five years, regardless of antiepileptic drug treatment. Diagnosis criteria based on "Guidelines for Epidemiological Studies on Epilepsy" (commissioned by the International League Against Epilepsy).
	Alternatives	Lifetime recall of epilepsy.
Fetal alcohol syndrome	Reference	Caused by maternal drinking during pregnancy and the most severe form of fetal alcohol spectrum disorder (FASD); other forms of FASD including partial fetal alcohol syndrome, alcohol-related neurodevelopmental disorder, and alcohol-related birth defects are not included. The syndrome is characterised by certain patterns of facial anomalies, growth retardation, and central nervous system neurodevelopmental abnormalities. Diagnostic criteria for active case finding comes from the USA Institute of Medicine, the British Paediatric Association, and other recognised bodies in the area.
	Alternatives	Passive case finding
Guillain-Barré syndrome	Reference	An immune-mediated nerve dysfunction that usually occurs as a complication of respiratory or gastrointestinal infection and leads to rapid onset of weakness in the feet and legs, and sometimes the arms, which then progresses toward the trunk. Cases are identified by doctor diagnosis or other record, including ICD-coded claims or hospital data.
Idiopathic intellectual disability	Reference	A condition of below-average mental ability originating before age 18, as defined by the American Association on Intellectual and Developmental Disabilities. A prevalent case is defined as an IQ score <70.
Klinefelter syndrome	Reference	Also known as 47 XXY, is a condition in which a male is born with an extra X chromosome in all or some of his cells; here the definition also includes other genotypes with supernumerary X chromosomes, eg, XXXY, XXXXY, etc. The primary feature is sterility, but it can cause a variety of other conditions, including weaker muscles, increased height, poor coordination abilities,

		smaller genitals, breast growth, and reduced sexual drive as a result of lower testosterone levels.
Malaria	Reference	An acute parasitic mosquito-borne disease – detectable *P. falciparum* or *P. vivax* parasites through microscopy and/or rapid diagnostic tests and clinical symptoms for malaria (fever, diarrhoea, and/or vomiting). For purposes of this analysis, the proportion of children under 5 with cerebral malaria who go on to have long-term disability (motor impairment, intellectual disability, seizures, blindness).
	Alternatives	People in malaria-endemic locations with clinical symptoms (fever, diarrhoea, and/or vomiting) for whom diagnostic testing was inconclusive or unavailable.
Meningitis	Reference	A disease caused by inflammation of the meninges, the protective membrane surrounding the brain and spinal cord, that is typically caused by an infection in the cerebrospinal fluid. Symptoms include headache, fever, stiff neck, and sometimes seizure. Gold-standard diagnosis in inpatient hospital clinical data or literature via antigen test, blood test, cerebrospinal fluid test, or latex agglutination test.
	Alternatives	Diagnosis from private insurance claims data or via epidemiological surveillance.
Migraine		A disabling primary headache disorder, typically characterised by recurrent moderate or severe unilateral pulsatile headaches, either without aura or with aura (transient neurological symptoms). Diagnosis based on International Classification of Headaches (ICD-3) criteria of 5+ attacks that (1) last 4–72 hours, (2) causes nausea and/or vomiting or photophobia and phonophobia, (3) has at least two of the following – unilateral location, pulsating quality, moderate or severe pain, aggravation by or causing avoidance of routine physical activity; (4) not due to other diagnosis. Definite migraines meet all of the above criteria and probable meet all but one of the above criteria.
	Alternatives	Other than one-year recall, not representative study population, low-quality methods (sampling, survey, diagnostic instrument, diagnostic criteria), poor response rate, headache type assumed
Motor neuron disease	Reference	A set of chronic, degenerative, and progressive neurological conditions typified by the destruction of motor neurons and the subsequent deterioration of voluntary muscle activity. The most common type is amyotrophic lateral sclerosis (ALS). Gold-standard diagnosis uses the El Escorial Criteria with clinical examination, as well as imaging and electrophysiology.
	Alternatives	Surveys limited to ALS case finding only.
Multiple sclerosis	Reference	A chronic, degenerative, and progressive condition typified by damage to the myelin sheaths around neurons. Accepted diagnostic criteria include McDonald's, Poser, Schumacher, and McAllen), or diagnosis via a clinical neurological exam.

Neonatal encephalopathy	Reference	Clinical diagnosis of disturbed neurological function in the earliest days of life in an infant born at or beyond 35 weeks' gestation, manifested by reduced level of consciousness or seizures, often accompanied by difficulty initiating and maintaining respiration, and by depression of tone and reflexes.
Neonatal jaundice	Reference	Clinical diagnosis of pathological neonatal jaundice or total serum bilirubin greater than expected for postnatal age and prematurity (hyperbilirubinaemia).
Neonatal preterm birth	Reference	Newborn born alive and less than 36 completed weeks of gestation; ie, birth between [0, 37.0) weeks of gestation; no adjustment is currently made between different forms of gestational age dating (obstetric estimate, last menstrual period, or other).
Neonatal sepsis	Reference	Includes infections during the neonatal period that advance to a systemic bloodstream infection (sepsis) and infections that occur during the neonatal period that are not already modelled separately in the GBD.
Nervous system cancers	Reference	Malignant neoplasm of the brain or central nervous system, or peripheral nervous system. Note: This combined nervous system cancer category is composed of two cancer groupings explicitly modelled in the GBD: "Brain and central nervous system cancer" and "Neuroblastoma and other peripheral nervous cell tumours". This category encompasses paediatric and adult cases, which includes primary malignancies but not metastases. Although rare, PNS tumours are included in the category.
Neural tube defects	Reference	Includes anencephaly, which is the absence of a major portion of the brain, skull, and scalp, encephalocele, which is characterised by sac-like protrusions of the brain and meninges through openings in the skull, and spina bifida, which is when part of the spinal cord and/or meninges are uncovered by skin. The reference definitions refer to livebirths including those with chromosomal anomalies.
	Alternatives	Livebirths excluding chromosomal diagnoses, livebirths and stillbirths
Neurocysticercosis	Reference	A parasitic disease caused by the pig tapeworm *Taenia solium* that leads to cysts in the brain and epilepsy. Diagnosis is made in epilepsy patients with either *T. Solium* identified in excised cysticerci from tissues by microscopic examination or identification of cysticerci using magnetic resonance imaging (MRI), computerised tomography (CT), or X-ray brain scans to identify cysts and a positive result on CDC immunoblot assay.
	Alternatives	An epilepsy patient with calcified cystic lesions in the brain identified by CT scan, MRI, or X-ray, or a positive result on CDC immunoblot assay ("probable" case).
Other chromosomal abnormalities	Reference	Unbalanced chromosomal rearrangements, which are genetic anomalies that typically occur due to meiotic non-disjunction, when homologous chromosomes do not separate normally in

		nuclear division during gamete formation. Other chromosomal arrangements included here are Triple X syndrome, other meiotic non-disjunction events, other female sex chromosome abnormalities, and other unspecified chromosomal abnormalities.
Other neurological disorders	Reference	A residual category which groups together neurological conditions that are not directly estimated in the GBD, for example muscular dystrophy, Huntington disease, and myasthenia gravis. A full list of included causes included in this category are listed in supplemental methods table 4.
Parkinson's disease	Reference	A chronic, degenerative, and progressive neurological condition typified by loss of motor mobility and control, most notably causing tremors. Requires the presence of two of the four primary symptoms and is consistent with the Gelb criteria: (1) tremors/trembling, (2) bradykinesia, (3) stiffness of limbs and torso, and (4) posture instability.
	Alternatives	Not population-representative, study excludes nursing homes, study uses the Movement Disorder Society (MDS) criteria, United Kingdom Parkinson's Disease Society (UKPD) Brain Bank criteria, or does not use explicit criteria.
Rabies	Reference	A viral infection transmitted by animal bite that is almost universally fatal without prophylactic vaccination. Clinical diagnosis of rabies excludes cases where intervention prevented disease following animal bite.
Spinal cord injury	Reference	A spinal cord lesion at or below the cervical level that leads to partial or total paralysis depending on the level and degree of injury.
Stroke (intracerebral haemorrhage)	Reference	A focal collection of blood within the brain parenchyma or ventricular system that is not caused by trauma. Stroke cases are considered acute from the day of incidence of a first-ever stroke through day 28 following the event. Stroke cases are considered chronic beginning 28 days following the occurrence of an event; includes all recurrent stroke events.
	Alternatives	Sources including first and recurrent strokes, estimates reported for all subtypes combined, estimates reported only for cases which survived to hospital admission.
Stroke (ischaemic)	Reference	Neurological dysfunction caused by focal cerebral, spinal, or retinal infarction. Stroke cases are considered acute from the day of incidence of a first-ever stroke through day 28 following the event. Stroke cases are considered chronic beginning 28 days following the occurrence of an event, and include all recurrent stroke events.
	Alternatives	Sources including first and recurrent strokes, estimates reported for all subtypes combined, estimates reported only for cases which survived to hospital admission.
	Reference	Bleeding into the subarachnoid space (the space between the arachnoid membrane and the pia mater of the brain or spinal

Stroke (subarachnoid haemorrhage)		cord). Stroke cases are considered acute from the day of incidence of a first-ever stroke through day 28 following the event. Stroke cases are considered chronic beginning 28 days following the occurrence of an event, and include all recurrent stroke events.
	Alternatives	Sources including first and recurrent strokes, estimates reported only for aneurysmal subarachnoid haemorrhage, estimates reported only for cases which survived to hospital admission.
Syphilis	Reference	Infection with the *Treponema pallidum* bacterium usually spread by sexual contact or from a pregnant person to offspring; we account here for acute and chronic infection, with or without symptoms, and sequelae of congenital cases that persist after treatment.
	Alternatives	Private insurance claims.
Tension-type headache		Characterised by a dull, non-pulsatile, diffuse, band- or vice-like pain of mild to moderate intensity in the head or neck. Diagnosed based on ICD-3 criteria of 10+ attacks that (1) last 30 minutes to 7 days, (2) no nausea or vomiting and no more than one of photophobia or phonophobia, (3) has at least two of the following – bilateral location, pressing or tightening quality, mild or moderate pain, not aggravated by routine physical activity, (4) not due to other diagnosis. Definite migraines meet all of the above criteria, and probable meet all but one of the above criteria.
	Alternatives	Other than one-year recall, not representative study population, low-quality methods (sampling, survey, diagnostic instrument, diagnostic criteria), poor response rate, headache type assumed
Tetanus	Reference	A life-threatening disease caused by infection with the toxin-producing bacterium *Clostridium tetani* and acquired via contamination of wounds. Tetanus is typically characterised by generalised, painful muscular spasms, with complications including mechanical respiratory failure, autonomic dysfunction, and death. Neonatal tetanus is often caused by contamination of the umbilical stump; initial symptoms include failure to feed and excessive crying, progressing to the typical clinical presentation of tetanus.
Traumatic brain injury	Reference	Injury to the head that causes short-term and in some cases long-term damage to the brain, manifesting in loss of concentration, headaches, memory problems, nausea, dizziness, and/or mood changes.

Supplemental methods table 6. ICD-9 and ICD-10 non-fatal coding for each condition*

**Condition**	**ICD-9**	**ICD-10**
AD/HD	314.0, 314.01	F90
Alzheimer's disease and other dementias	290, 291.2, 291.8, 294, 331	F00, F01, F02, F03, G30, G31

Autism spectrum disorder	299.00, 299.80, 299.8, 299.10	F84.0, F84.1, F84.2, F84.3, F84.4, F84.5, F84.8, F84.9
Congenital birth defects	4745, 746	Q20.0, Q20.3, Q21.3, Q21.4, Q22.0, Q22.1, Q22.2, Q22.3, Q22.5, Q22.8, Q22.9, Q23.0, Q26.2
COVID-19	N/A	N/A
Cystic echinococcosis	122.0-122.9	B67-B67.9
Diabetes	249, 250, 362	E08, E10-14
Down syndrome		Q90.0, Q90.1, Q90.2, Q90.9
Encephalitis	062, 063, 064, 323	A83-A86.4, B94.1, F07.1, G04-G05.8
Epilepsy	345	G40, G41
Fetal alcohol syndrome		Q86.0
Guillain-Barré syndrome	357.0	G61.0
Idiopathic intellectual disability	N/A	N/A
Klinefelter syndrome	758.7	Q98
Malaria		B50-54
Meningitis	036, 047-049.9, 320.0-320.3, 320.5-320.9, 321.5-321.7	A39, A87, G00-G03.0
Migraine	N/A	N/A
Motor neuron disease	335	G12
Multiple sclerosis	340	G35
Neonatal encephalopathy	768, 768.5, 768.6, 768.7, 768.71, 768.72, 768.73, 768.9, 779.1, 779.2	P21, P24, P91
Neonatal jaundice	N/A	N/A
Neonatal preterm birth	Find	Find
Neonatal sepsis	771	P36, P38, P39
Nervous system cancers**	191, 191.0, 191.1, 191.2, 191.3, 191.4, 191.5, 191.6, 191.7, 191.8, 191.9, 192, 192.0, 192.1, 192.2 192.3, 192.4, 192.8, 192.9, 194.3, 194.4, 194‡, 194.0‡, and 194.9‡	C47, C47.0, C47.1, C47.10. C47.11, C47.12, C47.2, C47.20, C47.21, C47.22, C47.3, C47.4, C47.5, C47.6, C47.8, C47.9, C47.90, C70, C70.0, C70.1, C70.5, C70.6, C70.9, C71, C71.0, C71.1, C71.2, C71.3, C71.4, C71.5, C71.6, C71.7, C71.8, C71.9, C72, C72.0, C72.1, C72.2, C72.20, C72.21, C72.22, C72.3, C72.30, C72.31, C72.32, C72.4, C72.40, C72.41, C72.42, C72.5, C72.50, C72.59, C72.8, C72.9, C74‡, C75.1-C75.3
Neural tube defects	740.0, 740.2, 741.0, 741.9, 742.0	Anencephaly: Q00.0, Q00.2; Encephalocele: Q01.2, Q01.8, Q01.9; Spina bifida: Q05.0, Q05.4, Q05.6, Q05.7, Q05.8, Q05.9
Neurocysticercosis	123.1	B69-B69.9
Other chromosomal abnormalities		Q92.0, Q97.0, Q97.8, Q99.9

Other neurological disorders^	330, 331.8, 331.9, 333, 334, 335.3 336, 337, 341, 349, 349.2, 349.3, 349.8, 353.8, 353.9, 356, 357.0, 357.1, 357.3, 357.4, 357.7, 358, 359, 728.88, 775.2	F02.2, G10, G11, G12, G12.0, G12.1, G13, G23, G24, G25, G26, G26.0, G36, G37, G61, G70, G71, G72, G73, G90, G95, M33
Parkinson's disease	332	G20
Rabies	071	A82
Spinal cord injury	N33, N34	N33, N34
Stroke (ischaemic)	433-435.9, 437.0-437.1, 437.5-437.8	G45-G46.8, I63-I63.9, I65-I66.9, I67.2-I67.3, I67.5-I67.6, I69.3
Stroke (intracerebral haemorrhage)	431-432.9, 437.2	I61-I62, I62.1-I62.9, I68.1-I68.2, I69.1-I69.2
Stroke (subarachnoid haemorrhage)	430-430.9	I60-I60.9, I62.0, I67.0-I67.1, I69.0
Syphilis	094	A52
Tension-type headache	N/A	N/A
Tetanus	037-037.9, 771.3, V03.7	A33-A35.0, Z23.5
Traumatic brain injury	N27, N28	N27, N28
Zika virus disease	N/A	N/A

*ICD codes are comprehensive for the condition, not just neurological sequelae unless otherwise specified; **denotes ICD codes used for non-fatal analysis only; ‡ Cases and deaths are redistributed by age group between the two GBD causes 'neuroblastoma and other peripheral nervous cell tumours' and 'other malignant neoplasms'; ^for other neurological conditions, ICD codes are used for fatal analysis only, the results of which are used to calculate years lived with disability.

Summary of Bayesian meta-regression methods

Modelling details vary by condition but generally use a Bayesian meta-regression tool called DisMod-MR 2.1. DisMod fits an initial model integrated across ages with all available input data regardless of geography or collection year and calculates predictive covariate coefficients. Then, seven super-region models are estimated using all relevant data from each respective super-region and the global model as a prior after adjustment by super-region random effects and covariate coefficients. This process is repeated for 21 region models, and then 204 country models. Estimates at the most granular level are summed to get final models at higher levels of the geographical hierarchy.

Supplemental methods table 7. Country-level predictive covariates in non-fatal models

Predictive covariates were used in non-fatal modelling to help inform estimates in data-poor locations and were selected based on evidence of a relationship between the covariate and respective condition. These predictive covariates are comprehensive for the condition, not just neurological sequelae.

**Condition**	**Predictive covariates**
AD/HD	None
Alzheimer's disease and other dementias	Education (years per capita), smoking prevalence
Autism spectrum disorder	None

Congenital birth defects	Legality of abortion
COVID-19	See relevant publication: Hanson et al. 2022[2]
Cystic echinococcosis	Echinococcosis endemicity, urbanicity, proportion of population involved in agricultural activities
Diabetes	Prevalence of obesity, year, Healthcare Access and Quality Index; Type 1 diabetes only: proportion of live births in women 35+ years, maternal education (years per capita)
Down syndrome	Legality of abortion, proportion of livebirths to women age 35+, Healthcare Access and Quality Index
Encephalitis	Japanese encephalitis endemic area, lag-distributed income (per capita)
Epilepsy	Lag-distributed income (per capita), SEV for idiopathic epilepsy
Fetal alcohol syndrome	Maternal alcohol consumption during pregnancy (proportion)
Guillain-Barré syndrome	None
Idiopathic intellectual disability	Lag-distributed income (per capita), SEV for child underweight
Klinefelter syndrome	Legality of abortion, proportion of livebirths to women age 35+, Healthcare Access and Quality Index
Malaria	See publications for details: Battle et al. 2019[3], Weiss et al. 2019[4]
Meningitis	Hib3 vaccine coverage (proportion), PCV3 coverage (proportion), meningitis belt (proportion), proportion of total population covered by MenAfriVac initiative (meningitis meningococcal type A vaccine), Healthcare Access and Quality Index
Migraine	None
Motor neuron disease	Average latitude (absolute value), lag-distributed income (per capita)
Multiple sclerosis	Average latitude (absolute value), Healthcare Access and Quality Index
Neonatal encephalopathy	Antenatal care coverage (1+ visits), in-facility delivery, lag-distributed income per capita, livebirths among women aged 35+ years, total fertility rate, maternal care and immunisation, Socio-demographic Index, HAQ Index, maternal education (6+ years and 12+ years), ambient particulate matter SEV, household air pollution SEV, SEV for low birthweight, SEV for short gestation, and SEV for smoking
Neonatal jaundice	Total fertility rate, Healthcare Access and Quality Index
Neonatal sepsis	Lag-distributed income per capita, Socio-demographic Index, HAQ Index, unsafe water SEV, unsafe sanitation SEV, maternal care and immunisation index, livebirths among women aged 35+ years, preterm birth SEV, low birthweight SEV, short gestation SEV, smoking SEV, mortality due to war and conflict, and neonatal CSMR
Neural tube defects	Folic acid availability, folic acid fortification
Nervous system cancers*†	Cumulative cigarettes (10 years), education (years per capita), lag-distributed income (per capita), cholesterol (total, mean per capita), smoking prevalence, Socio-demographic Index, Healthcare Access and Quality Index, summary exposure value (SEV) for low fruit, SEV for low vegetables, SEV for high red meat, litres of alcohol consumed per capita, systolic blood pressure (mmHg), universal health coverage, health worker density, maternal care and immunisation

Neurocysticercosis	Proportion of population that is Muslim, religion (binary, >50% Muslim), Socio-demographic Index, pigs raised in extensive agricultural systems (per capita)
Other chromosomal abnormalities	Legality of abortion, proportion of livebirths to women age 35+, Healthcare Access and Quality Index
Other neurological disorders†	Cumulative cigarettes (5 years, 10 years), education (years per capita), lag-distributed income (per capita), mean body-mass index, mean cholesterol, mean systolic blood pressure, pigs per capita, population density over 1000 per square km (percentage), smoking prevalence, Socio-demographic Index, Healthcare Access and Quality Index, SEV for underweight, SEV for low fruit, SEV for diet high in red meat
Parkinson's disease	Smoking prevalence, Healthcare Access and Quality Index
Rabies†	ANC6 coverage proportion, IFD coverage proportion, population density between 500 and 1000 people per square km (percentage), population density under 150 people per square kilometre (percentage), SBA coverage proportion, maternal care and immunisation, Socio-demographic Index, Healthcare Access and Quality Index
Spinal cord injury	None
Stroke (ischaemic)	SEV for ischaemic stroke, lag-distributed income (per capita)
Stroke (intracerebral haemorrhage)	SEV for intracerebral stroke, lag-distributed income (per capita)
Stroke (subarachnoid haemorrhage)	Lag-distributed income (per capita), systolic blood pressure (mmHg)
Syphilis	Healthcare Access and Quality Index
Tension-type headache	None
Tetanus	Healthcare Access and Quality Index
Traumatic brain injury	None
Zika virus disease	Healthcare Access and Quality Index, enhanced vegetation index, mean temperature, rainfall, sanitation, solar radiation

*Nervous system cancers refers to the combination of "Brain and central nervous system cancer" and "Neuroblastoma and other peripheral nervous cell tumours"; †predictive covariates come from Cause of Death modelling, which is used to inform non-fatal estimates. Abbreviations: SEV=summary exposure value.

Supplemental methods table 8. Global Burden of Disease geographical hierarchy

The below table is organised by seven super-regions (headers), their corresponding regions (left column), and each region's corresponding countries (right column). Countries where subnational estimates were analysed are noted.

**Central Europe, eastern Europe, and central Asia**	
Central Asia	Armenia, Azerbaijan, Georgia, Kazakhstan, Kyrgyzstan, Mongolia, Tajikistan, Turkmenistan, Uzbekistan
Central Europe	Albania, Bosnia and Herzegovina, Bulgaria, Croatia, Czech Republic, Hungary, Montenegro, North Macedonia, Poland (subnational), Romania, Serbia, Slovakia, Slovenia
Eastern Europe	Belarus, Estonia, Latvia, Lithuania, Moldova, Russia (subnational), Ukraine
**High-income**	

Australasia	Australia, New Zealand (subnational Māori + non-Māori)
High-income Asia Pacific	Brunei, Japan (subnational), Singapore, South Korea
High-income North America	Canada, Greenland, USA (subnational)
Southern Latin America	Argentina, Chile, Uruguay
Western Europe	Andorra, Austria, Belgium, Cyprus, Denmark, Finland, France, Germany, Greece, Iceland, Ireland, Israel, Italy (subnational), Luxembourg, Malta, Monaco, Netherlands, Norway (subnational), Portugal, San Marino, Spain, Sweden (subnational), Switzerland, UK (subnational)
**Latin America and Caribbean**	
Andean Latin America	Bolivia, Ecuador, Peru
Caribbean	Antigua and Barbuda, Bahamas, Barbados, Belize, Bermuda, Cuba, Dominica, Dominican Republic, Grenada, Guyana, Haiti, Jamaica, Puerto Rico, Saint Kitts and Nevis, Saint Lucia, Saint Vincent and the Grenadines, Suriname, Trinidad and Tobago, Virgin Islands
Central Latin America	Colombia, Costa Rica, El Salvador, Guatemala, Honduras, Mexico (subnational), Nicaragua, Panama, Venezuela
Tropical Latin America	Brazil (subnational), Paraguay
**North Africa and Middle East**	
North Africa and Middle East	Afghanistan, Algeria, Bahrain, Egypt, Iran (subnational), Iraq, Jordan, Kuwait, Lebanon, Libya, Morocco, Oman, Palestine, Qatar, Saudi Arabia, Sudan, Syria, Tunisia, Türkiye, United Arab Emirates, Yemen
**South Asia**	
South Asia	Bangladesh, Bhutan, India (subnational), Nepal, Pakistan (subnational)
**Southeast Asia, east Asia, Oceania**	
East Asia	China, North Korea, Taiwan (province of China)
Oceania	American Samoa, Cook Islands, Federated States of Micronesia, Fiji, Guam, Kiribati, Marshall Islands, Nauru, Niue, Northern Mariana Islands, Palau, Papua New Guinea, Samoa, Solomon Islands, Tokelau, Tonga, Tuvalu, Vanuatu
Southeast Asia	Cambodia, Indonesia (subnational), Laos, Malaysia, Maldives, Mauritius, Myanmar, Philippines (subnational), Seychelles, Sri Lanka, Thailand, Timor-Leste, Viet Nam
**Sub-Saharan Africa**	
Central sub-Saharan Africa	Angola, Central African Republic, Congo (Brazzaville), Democratic Republic of the Congo, Equatorial Guinea, Gabon
Eastern sub-Saharan Africa	Burundi, Comoros, Djibouti, Eritrea, Ethiopia (subnational), Kenya (subnational), Madagascar, Malawi, Mozambique, Rwanda, Somalia, South Sudan, Tanzania, Uganda, Zambia
Southern sub-Saharan Africa	Botswana, Eswatini, Lesotho, Namibia, South Africa (subnational), Zimbabwe
Western sub-Saharan Africa	Benin, Burkina Faso, Cabo Verde, Cameroon, Chad, Côte d'Ivoire, Gambia, Ghana, Guinea, Guinea-Bissau, Liberia, Mali, Mauritania, Niger, Nigeria (subnational), São Tomé and Príncipe, Senegal, Sierra Leone, Togo

Accounting for transiency of some neurological conditions: two examples

Disability can either be transient or permanent/progressive depending on the disease, and we account for this in our analysis. For example, to calculate years lived with disability for headaches, we use a meta-analysis of 19 studies from “Lifting the Burden” (a collaboration with WHO aimed at headache data collection using the validated HARDSHIP questionnaire: https://www.l-t-b.org/) to assess both headache frequency and duration and use this to proportionally split headaches (tension-type and migraine) into symptomatic and asymptomatic. We have separate health states and disability weights for each of these categories to reflect the differing impact on health. For epilepsy, we have differing health states for severe (>= one seizure per month on average) versus less severe (< one seizure per month) epilepsy, and our case definition specifies “active” epilepsy, defined as having at least one seizure in the preceding five years, regardless of antiepileptic treatment. We also assess treatment gap (proportion of those with epilepsy adherent to antiepileptic drugs) and treatment success (proportion with successfully treated epilepsy), where the corresponding health state for treated epilepsy has a much lower disability weight than other epilepsy disability weights (0.049 for treated vs 0.263 for less severe epilepsy, vs 0.552 for severe epilepsy).

Supplemental methods table 9. Sequelae included in analysis

Condition	Sequela
Alzheimer's disease and other dementias	Mild Alzheimer's disease and other dementias
Alzheimer's disease and other dementias	Moderate Alzheimer's disease and other dementias
Alzheimer's disease and other dementias	Severe Alzheimer's disease and other dementias
Attention-deficit/hyperactivity disorder	Symptomatic attention-deficit/hyperactivity disorder
Attention-deficit/hyperactivity disorder	Attention-deficit/hyperactivity disorder, currently without symptoms
Autism spectrum disorder	Autism spectrum disorders without intellectual disability
Autism spectrum disorder	Autism spectrum disorders with borderline intellectual disability
Autism spectrum disorder	Autism spectrum disorders with mild intellectual disability
Autism spectrum disorder	Autism spectrum disorders with moderate intellectual disability
Autism spectrum disorder	Autism spectrum disorders with severe intellectual disability
Autism spectrum disorder	Autism spectrum disorders with profound intellectual disability
Autism spectrum disorder	Asperger syndrome and other autistic spectrum disorders
Brain and central nervous system cancers	Diagnosis and primary therapy phase of brain and central nervous system cancers
Brain and central nervous system cancers	Controlled phase of brain and central nervous system cancers
Brain and central nervous system cancers	Metastatic phase of brain and central nervous system cancers
Brain and central nervous system cancers	Terminal phase of brain and central nervous system cancers
Congenital birth defects	Mild hearing loss due to other congenital anomalies
Congenital birth defects	Mild hearing loss with ringing due to other congenital anomalies
Congenital birth defects	Moderate hearing loss due to other congenital anomalies
Congenital birth defects	Moderate hearing loss with ringing due to other congenital anomalies
Congenital birth defects	Severe hearing loss with ringing due to other congenital anomalies
Congenital birth defects	Severe hearing loss due to other congenital anomalies
Congenital birth defects	Complete hearing loss due to other congenital anomalies
Congenital birth defects	Complete hearing loss with ringing due to other congenital anomalies
Congenital birth defects	Moderately severe hearing loss due to other congenital anomalies
Congenital birth defects	Moderately severe hearing loss with ringing due to other congenital anomalies
Congenital birth defects	Profound hearing loss due to other congenital anomalies
Congenital birth defects	Profound hearing loss with ringing due to other congenital anomalies
Congenital birth defects	Developmental delay or mild intellectual disability due to congenital diaphragmatic hernia

Congenital birth defects	Severe motor and cognitive impairment due to Edward syndrome or Patau syndrome
Congenital birth defects	Severe motor and cognitive impairment with congenital heart disease due to Edward syndrome or Patau syndrome
Congenital birth defects	Congenital heart disease and borderline intellectual disability without heart failure due to other congenital cardiovascular anomalies
Congenital birth defects	Congenital heart disease and mild intellectual disability without heart failure due to other congenital cardiovascular anomalies
Congenital birth defects	Congenital heart disease and moderate intellectual disability without heart failure due to other congenital cardiovascular anomalies
Congenital birth defects	Congenital heart disease and severe intellectual disability without heart failure due to other congenital cardiovascular anomalies
Congenital birth defects	Congenital heart disease and profound intellectual disability without heart failure due to other congenital cardiovascular anomalies
Congenital birth defects	Congenital heart disease and borderline intellectual disability without heart failure due to single ventricle and single ventricle pathway heart defects
Congenital birth defects	Congenital heart disease and mild intellectual disability without heart failure due to single ventricle and single ventricle pathway heart defects
Congenital birth defects	Congenital heart disease and moderate intellectual disability without heart failure due to single ventricle and single ventricle pathway heart defects
Congenital birth defects	Congenital heart disease and severe intellectual disability without heart failure due to single ventricle and single ventricle pathway heart defects
Congenital birth defects	Congenital heart disease and profound intellectual disability without heart failure due to single ventricle and single ventricle pathway heart defects
Congenital birth defects	Congenital heart disease, borderline intellectual disability, and mild heart failure due to single ventricle and single ventricle pathway heart defects
Congenital birth defects	Congenital heart disease, mild intellectual disability, and mild heart failure due to single ventricle and single ventricle pathway heart defects
Congenital birth defects	Congenital heart disease, moderate intellectual disability, and mild heart failure due to single ventricle and single ventricle pathway heart defects
Congenital birth defects	Congenital heart disease, severe intellectual disability, and mild heart failure due to single ventricle and single ventricle pathway heart defects
Congenital birth defects	Congenital heart disease, profound intellectual disability, and mild heart failure due to single ventricle and single ventricle pathway heart defects
Congenital birth defects	Congenital heart disease, borderline intellectual disability, and moderate heart failure due to single ventricle and single ventricle pathway heart defects
Congenital birth defects	Congenital heart disease, mild intellectual disability, and moderate heart failure due to single ventricle and single ventricle pathway heart defects
Congenital birth defects	Congenital heart disease, moderate intellectual disability, and moderate heart failure due to single ventricle and single ventricle pathway heart defects
Congenital birth defects	Congenital heart disease, severe intellectual disability, and moderate heart failure due to single ventricle and single ventricle pathway heart defects
Congenital birth defects	Congenital heart disease, profound intellectual disability, and moderate heart failure due to single ventricle and single ventricle pathway heart defects
Congenital birth defects	Congenital heart disease, borderline intellectual disability, and severe heart failure due to single ventricle and single ventricle pathway heart defects

Congenital birth defects	Congenital heart disease, mild intellectual disability, and severe heart failure due to single ventricle and single ventricle pathway heart defects
Congenital birth defects	Congenital heart disease, moderate intellectual disability, and severe heart failure due to single ventricle and single ventricle pathway heart defects
Congenital birth defects	Congenital heart disease, severe intellectual disability, and severe heart failure due to single ventricle and single ventricle pathway heart defects
Congenital birth defects	Congenital heart disease, profound intellectual disability, and severe heart failure due to single ventricle and single ventricle pathway heart defects
Congenital birth defects	Congenital heart disease and mild intellectual disability without heart failure due to severe congenital heart anomalies excluding single ventricle heart defects
Congenital birth defects	Congenital heart disease and moderate intellectual disability without heart failure due to severe congenital heart anomalies excluding single ventricle heart defects
Congenital birth defects	Congenital heart disease and severe intellectual disability without heart failure due to severe congenital heart anomalies excluding single ventricle heart defects
Congenital birth defects	Congenital heart disease and profound intellectual disability without heart failure due to severe congenital heart anomalies excluding single ventricle heart defects
Congenital birth defects	Congenital heart disease, borderline intellectual disability, and mild heart failure due to severe congenital heart anomalies excluding single ventricle heart defects
Congenital birth defects	Congenital heart disease, mild intellectual disability, and mild heart failure due to severe congenital heart anomalies excluding single ventricle heart defects
Congenital birth defects	Congenital heart disease, moderate intellectual disability, and mild heart failure due to severe congenital heart anomalies excluding single ventricle heart defects
Congenital birth defects	Congenital heart disease, severe intellectual disability, and mild heart failure due to severe congenital heart anomalies excluding single ventricle heart defects
Congenital birth defects	Congenital heart disease, profound intellectual disability, and mild heart failure due to severe congenital heart anomalies excluding single ventricle heart defects
Congenital birth defects	Congenital heart disease, borderline intellectual disability, and moderate heart failure due to severe congenital heart anomalies excluding single ventricle heart defects
Congenital birth defects	Congenital heart disease, mild intellectual disability, and moderate heart failure due to severe congenital heart anomalies excluding single ventricle heart defects
Congenital birth defects	Congenital heart disease, moderate intellectual disability, and moderate heart failure due to severe congenital heart anomalies excluding single ventricle heart defects
Congenital birth defects	Congenital heart disease, severe intellectual disability, and moderate heart failure due to severe congenital heart anomalies excluding single ventricle heart defects
Congenital birth defects	Congenital heart disease, profound intellectual disability, and moderate heart failure due to severe congenital heart anomalies excluding single ventricle heart defects
Congenital birth defects	Congenital heart disease, borderline intellectual disability, and severe heart failure due to severe congenital heart anomalies excluding single ventricle heart defects
Congenital birth defects	Congenital heart disease, mild intellectual disability, and severe heart failure due to severe congenital heart anomalies excluding single ventricle heart defects

Congenital birth defects	Congenital heart disease, moderate intellectual disability, and severe heart failure due to severe congenital heart anomalies excluding single ventricle heart defects
Congenital birth defects	Congenital heart disease, severe intellectual disability, and severe heart failure due to severe congenital heart anomalies excluding single ventricle heart defects
Congenital birth defects	Congenital heart disease, profound intellectual disability, and severe heart failure due to severe congenital heart anomalies excluding single ventricle heart defects
Congenital birth defects	Congenital heart disease and borderline intellectual disability without heart failure due to critical malformations of great vessels, congenital valvular heart disease and patent ductus arteriosus
Congenital birth defects	Congenital heart disease and mild intellectual disability without heart failure due to critical malformations of great vessels, congenital valvular heart disease and patent ductus arteriosus
Congenital birth defects	Congenital heart disease and moderate intellectual disability without heart failure due to critical malformations of great vessels, congenital valvular heart disease and patent ductus arteriosus
Congenital birth defects	Congenital heart disease and severe intellectual disability without heart failure due to critical malformations of great vessels, congenital valvular heart disease and patent ductus arteriosus
Congenital birth defects	Congenital heart disease and profound intellectual disability without heart failure due to critical malformations of great vessels, congenital valvular heart disease and patent ductus arteriosus
Congenital birth defects	Congenital heart disease, borderline intellectual disability, and mild heart failure due to critical malformations of great vessels, congenital valvular heart disease and patent ductus arteriosus
Congenital birth defects	Congenital heart disease, mild intellectual disability, and mild heart failure due to critical malformations of great vessels, congenital valvular heart disease and patent ductus arteriosus
Congenital birth defects	Congenital heart disease, moderate intellectual disability, and mild heart failure due to critical malformations of great vessels, congenital valvular heart disease and patent ductus arteriosus
Congenital birth defects	Congenital heart disease, severe intellectual disability, and mild heart failure due to critical malformations of great vessels, congenital valvular heart disease and patent ductus arteriosus
Congenital birth defects	Congenital heart disease, profound intellectual disability, and mild heart failure due to critical malformations of great vessels, congenital valvular heart disease and patent ductus arteriosus
Congenital birth defects	Congenital heart disease, borderline intellectual disability, and moderate heart failure due to critical malformations of great vessels, congenital valvular heart disease and patent ductus arteriosus
Congenital birth defects	Congenital heart disease, mild intellectual disability, and moderate heart failure due to critical malformations of great vessels, congenital valvular heart disease and patent ductus arteriosus
Congenital birth defects	Congenital heart disease, moderate intellectual disability, and moderate heart failure due to critical malformations of great vessels, congenital valvular heart disease and patent ductus arteriosus
Congenital birth defects	Congenital heart disease, severe intellectual disability, and moderate heart failure due to critical malformations of great vessels, congenital valvular heart disease and patent ductus arteriosus
Congenital birth defects	Congenital heart disease, profound intellectual disability, and moderate heart failure due to critical malformations of great vessels, congenital valvular heart disease and patent ductus arteriosus
Congenital birth defects	Congenital heart disease, borderline intellectual disability, and severe heart failure due to critical malformations of great vessels, congenital valvular heart disease and patent ductus arteriosus
Congenital birth defects	Congenital heart disease, mild intellectual disability, and severe heart failure due to critical malformations of great vessels, congenital valvular heart disease and patent ductus arteriosus

Congenital birth defects	Congenital heart disease, moderate intellectual disability, and severe heart failure due to critical malformations of great vessels, congenital valvular heart disease and patent ductus arteriosus
Congenital birth defects	Congenital heart disease, severe intellectual disability, and severe heart failure due to critical malformations of great vessels, congenital valvular heart disease and patent ductus arteriosus
Congenital birth defects	Congenital heart disease, profound intellectual disability, and severe heart failure due to critical malformations of great vessels, congenital valvular heart disease and patent ductus arteriosus
Congenital birth defects	Congenital heart disease and borderline intellectual disability without heart failure due to ventricular septal defect and atrial septal defect
Congenital birth defects	Congenital heart disease and mild intellectual disability without heart failure due to ventricular septal defect and atrial septal defect
Congenital birth defects	Congenital heart disease and moderate intellectual disability without heart failure due to ventricular septal defect and atrial septal defect
Congenital birth defects	Congenital heart disease and severe intellectual disability without heart failure due to ventricular septal defect and atrial septal defect
Congenital birth defects	Congenital heart disease and profound intellectual disability without heart failure due to ventricular septal defect and atrial septal defect
Congenital birth defects	Congenital heart disease, borderline intellectual disability, and mild heart failure due to ventricular septal defect and atrial septal defect
Congenital birth defects	Congenital heart disease, mild intellectual disability, and mild heart failure due to ventricular septal defect and atrial septal defect
Congenital birth defects	Congenital heart disease, moderate intellectual disability, and mild heart failure due to ventricular septal defect and atrial septal defect
Congenital birth defects	Congenital heart disease, severe intellectual disability, and mild heart failure due to ventricular septal defect and atrial septal defect
Congenital birth defects	Congenital heart disease, profound intellectual disability, and mild heart failure due to ventricular septal defect and atrial septal defect
Congenital birth defects	Congenital heart disease, borderline intellectual disability, and moderate heart failure due to ventricular septal defect and atrial septal defect
Congenital birth defects	Congenital heart disease, mild intellectual disability, and moderate heart failure due to ventricular septal defect and atrial septal defect
Congenital birth defects	Congenital heart disease, moderate intellectual disability, and moderate heart failure due to ventricular septal defect and atrial septal defect
Congenital birth defects	Congenital heart disease, severe intellectual disability, and moderate heart failure due to ventricular septal defect and atrial septal defect
Congenital birth defects	Congenital heart disease, profound intellectual disability, and moderate heart failure due to ventricular septal defect and atrial septal defect
Congenital birth defects	Congenital heart disease, borderline intellectual disability, and severe heart failure due to ventricular septal defect and atrial septal defect
Congenital birth defects	Congenital heart disease, mild intellectual disability, and severe heart failure due to ventricular septal defect and atrial septal defect
Congenital birth defects	Congenital heart disease, moderate intellectual disability, and severe heart failure due to ventricular septal defect and atrial septal defect
Congenital birth defects	Congenital heart disease, severe intellectual disability, and severe heart failure due to ventricular septal defect and atrial septal defect
Congenital birth defects	Congenital heart disease, profound intellectual disability, and severe heart failure due to ventricular septal defect and atrial septal defect
Congenital birth defects	Congenital heart disease, borderline intellectual disability and controlled, medically managed heart failure due to critical malformations of great vessels, congenital valvular heart disease and patent ductus arteriosus

Congenital birth defects	Congenital heart disease, borderline intellectual disability and controlled, medically managed heart failure due to severe congenital heart anomalies excluding single ventricle heart defects
Congenital birth defects	Congenital heart disease, borderline intellectual disability and controlled, medically managed heart failure due to single ventricle and single ventricle pathway heart defects
Congenital birth defects	Congenital heart disease, borderline intellectual disability and controlled, medically managed heart failure due to ventricular septal defect and atrial septal defect
Congenital birth defects	Congenital heart disease, mild intellectual disability and controlled, medically managed heart failure due to critical malformations of great vessels, congenital valvular heart disease and patent ductus arteriosus
Congenital birth defects	Congenital heart disease, mild intellectual disability and controlled, medically managed heart failure due to severe congenital heart anomalies excluding single ventricle heart defects
Congenital birth defects	Congenital heart disease, mild intellectual disability and controlled, medically managed heart failure due to single ventricle and single ventricle pathway heart defects
Congenital birth defects	Congenital heart disease, mild intellectual disability and controlled, medically managed heart failure due to ventricular septal defect and atrial septal defect
Congenital birth defects	Congenital heart disease, moderate intellectual disability and controlled, medically managed heart failure due to critical malformations of great vessels, congenital valvular heart disease and patent ductus arteriosus
Congenital birth defects	Congenital heart disease, moderate intellectual disability and controlled, medically managed heart failure due to severe congenital heart anomalies excluding single ventricle heart defects
Congenital birth defects	Congenital heart disease, moderate intellectual disability and controlled, medically managed heart failure due to single ventricle and single ventricle pathway heart defects
Congenital birth defects	Congenital heart disease, moderate intellectual disability and controlled, medically managed heart failure due to ventricular septal defect and atrial septal defect
Congenital birth defects	Congenital heart disease, profound intellectual disability and controlled, medically managed heart failure due to severe congenital heart anomalies excluding single ventricle heart defects
Congenital birth defects	Congenital heart disease, profound intellectual disability and controlled, medically managed heart failure due to single ventricle and single ventricle pathway heart defects
Congenital birth defects	Congenital heart disease, profound intellectual disability and controlled, medically managed heart failure due to ventricular septal defect and atrial septal defect
Congenital birth defects	Congenital heart disease, profound intellectual disability and controlled, medically managed heart failure due to critical malformations of great vessels, congenital valvular heart disease and patent ductus arteriosus
Congenital birth defects	Congenital heart disease, severe intellectual disability and controlled, medically managed heart failure due to critical malformations of great vessels, congenital valvular heart disease and patent ductus arteriosus
Congenital birth defects	Congenital heart disease, severe intellectual disability and controlled, medically managed heart failure due to severe congenital heart anomalies excluding single ventricle heart defects
Congenital birth defects	Congenital heart disease, severe intellectual disability and controlled, medically managed heart failure due to single ventricle and single ventricle pathway heart defects
Congenital birth defects	Congenital heart disease, severe intellectual disability and controlled, medically managed heart failure due to ventricular septal defect and atrial septal defect
COVID-19	Post-acute mild cognitive symptoms due to COVID-19
COVID-19	Post-acute severe cognitive symptoms due to COVID-19

COVID-19	Post-acute fatigue syndrome and mild cognitive symptoms due to COVID-19
COVID-19	Post-acute fatigue syndrome and severe cognitive symptoms due to COVID-19
COVID-19	Post-acute mild respiratory and mild cognitive symptoms due to COVID-19
COVID-19	Post-acute mild respiratory and severe cognitive symptoms due to COVID-19
COVID-19	Post-acute moderate respiratory and mild cognitive symptoms due to COVID-19
COVID-19	Post-acute moderate respiratory and severe cognitive symptoms due to COVID-19
COVID-19	Post-acute severe respiratory and mild cognitive symptoms due to COVID-19
COVID-19	Post-acute severe respiratory and severe cognitive symptoms due to COVID-19
COVID-19	Post-acute fatigue syndrome and mild respiratory and mild cognitive symptoms due to COVID-19
COVID-19	Post-acute fatigue syndrome and mild respiratory and severe cognitive symptoms due to COVID-19
COVID-19	Post-acute fatigue syndrome and moderate respiratory and mild cognitive symptoms due to COVID-19
COVID-19	Post-acute fatigue syndrome and moderate respiratory and severe cognitive symptoms due to COVID-19
COVID-19	Post-acute fatigue syndrome and severe respiratory and mild cognitive symptoms due to COVID-19
COVID-19	Post-acute fatigue syndrome and severe respiratory and severe cognitive symptoms due to COVID-19
COVID-19	Guillain-Barré syndrome due to COVID-19
Diabetes	Diabetic foot due to neuropathy due to diabetes mellitus type 1
Diabetes	Diabetic neuropathy due to diabetes mellitus type 1, without diabetic foot or amputation
Diabetes	Diabetic neuropathy and amputation with treatment due to diabetes mellitus type 1
Diabetes	Diabetic neuropathy and amputation without treatment due to diabetes mellitus type 1
Diabetes	Diabetic foot due to neuropathy due to diabetes mellitus type 2
Diabetes	Diabetic neuropathy due to diabetes mellitus type 2, without diabetic foot or amputation
Diabetes	Diabetic neuropathy and amputation with treatment due to diabetes mellitus type 2
Diabetes	Diabetic neuropathy and amputation without treatment due to diabetes mellitus type 2
Down syndrome	Borderline intellectual disability due to Down syndrome
Down syndrome	Mild intellectual disability due to Down syndrome
Down syndrome	Moderate intellectual disability due to Down syndrome
Down syndrome	Severe intellectual disability due to Down syndrome
Down syndrome	Profound intellectual disability due to Down syndrome
Down syndrome	Borderline intellectual disability with congenital heart disease due to Down syndrome
Down syndrome	Mild intellectual disability with congenital heart disease due to Down syndrome
Down syndrome	Moderate intellectual disability with congenital heart disease due to Down syndrome

Down syndrome	Severe intellectual disability with congenital heart disease due to Down syndrome
Down syndrome	Profound intellectual disability with congenital heart disease due to Down syndrome
Down syndrome	Mild dementia due to Down syndrome
Down syndrome	Moderate dementia due to Down syndrome
Down syndrome	Severe dementia due to Down syndrome
Down syndrome	Congenital heart disease and mild dementia due to Down syndrome
Down syndrome	Congenital heart disease and moderate dementia due to Down syndrome
Down syndrome	Congenital heart disease and severe dementia due to Down syndrome
Down syndrome	Borderline intellectual disability, mild dementia, and congenital heart disease due to Down syndrome
Down syndrome	Mild intellectual disability, mild dementia, and congenital heart disease due to Down syndrome
Down syndrome	Moderate intellectual disability, mild dementia, and congenital heart disease due to Down syndrome
Down syndrome	Severe intellectual disability, mild dementia, and congenital heart disease due to Down syndrome
Down syndrome	Profound intellectual disability, mild dementia, and congenital heart disease due to Down syndrome
Down syndrome	Borderline intellectual disability, moderate dementia, and congenital heart disease due to Down syndrome
Down syndrome	Mild intellectual disability, moderate dementia, and congenital heart disease due to Down syndrome
Down syndrome	Moderate intellectual disability, moderate dementia, and congenital heart disease due to Down syndrome
Down syndrome	Severe intellectual disability, moderate dementia, and congenital heart disease due to Down syndrome
Down syndrome	Profound intellectual disability, moderate dementia, and congenital heart disease due to Down syndrome
Down syndrome	Borderline intellectual disability, severe dementia, and congenital heart disease due to Down syndrome
Down syndrome	Mild intellectual disability, severe dementia, and congenital heart disease due to Down syndrome
Down syndrome	Moderate intellectual disability, severe dementia, and congenital heart disease due to Down syndrome
Down syndrome	Severe intellectual disability, severe dementia, and congenital heart disease due to Down syndrome
Down syndrome	Profound intellectual disability, severe dementia, and congenital heart disease due to Down syndrome
Down syndrome	Profound intellectual disability and mild dementia due to Down syndrome
Down syndrome	Severe intellectual disability and mild dementia due to Down syndrome
Down syndrome	Moderate intellectual disability and mild dementia due to Down syndrome
Down syndrome	Mild intellectual disability and mild dementia due to Down syndrome
Down syndrome	Borderline intellectual disability and mild dementia due to Down syndrome
Down syndrome	Profound intellectual disability and moderate dementia due to Down syndrome
Down syndrome	Severe intellectual disability and moderate dementia due to Down syndrome
Down syndrome	Moderate intellectual disability and moderate dementia due to Down syndrome

Down syndrome	Mild intellectual disability and moderate dementia due to Down syndrome
Down syndrome	Borderline intellectual disability and moderate dementia due to Down syndrome
Down syndrome	Profound intellectual disability and severe dementia due to Down syndrome
Down syndrome	Severe intellectual disability and severe dementia due to Down syndrome
Down syndrome	Moderate intellectual disability and severe dementia due to Down syndrome
Down syndrome	Mild intellectual disability and severe dementia due to Down syndrome
Down syndrome	Borderline intellectual disability and severe dementia due to Down syndrome
Echinococcosis	Epilepsy due to echinococcosis
Encephalitis	Mild behavioural problems due to encephalitis
Encephalitis	Mild motor impairment due to encephalitis
Encephalitis	Mild motor plus cognitive impairments due to encephalitis
Encephalitis	Borderline intellectual disability due to encephalitis
Encephalitis	Monocular distance vision loss due to encephalitis
Encephalitis	Mild intellectual disability due to encephalitis
Encephalitis	Epilepsy due to encephalitis
Encephalitis	Blindness due to encephalitis
Encephalitis	Moderate vision impairment due to encephalitis
Encephalitis	Severe vision impairment due to encephalitis
Encephalitis	Moderate motor impairment due to encephalitis
Encephalitis	Severe motor impairment due to encephalitis
Encephalitis	Moderate motor plus cognitive impairments due to encephalitis
Encephalitis	Severe motor plus cognitive impairments due to encephalitis
Epilepsy	Idiopathic, seizure-free, treated epilepsy
Epilepsy	Idiopathic, less severe epilepsy
Epilepsy	Idiopathic, severe epilepsy
Fetal alcohol syndrome	Mild fetal alcohol syndrome
Fetal alcohol syndrome	Moderate fetal alcohol syndrome
Fetal alcohol syndrome	Severe fetal alcohol syndrome
Guillain-Barré syndrome	Guillain-Barré syndrome due to upper respiratory infections
Guillain-Barré syndrome	Guillain-Barré syndrome due to other infectious diseases
Guillain-Barré syndrome	Guillain-Barré syndrome due to other neurological disorders

Guillain-Barré syndrome	Guillain-Barré syndrome due to diarrhoeal diseases
Guillain-Barré syndrome	Guillain-Barré syndrome due to lower respiratory infections
Guillain-Barré syndrome	Guillain-Barré syndrome due to Zika infection
Idiopathic developmental intellectual disability	Borderline idiopathic developmental intellectual disability
Idiopathic developmental intellectual disability	Mild idiopathic developmental intellectual disability
Idiopathic developmental intellectual disability	Moderate idiopathic developmental intellectual disability
Idiopathic developmental intellectual disability	Severe idiopathic developmental intellectual disability
Idiopathic developmental intellectual disability	Profound idiopathic developmental intellectual disability
Intracerebral haemorrhage	Acute intracerebral haemorrhage severity level 1
Intracerebral haemorrhage	Acute intracerebral haemorrhage severity level 2, without heart failure
Intracerebral haemorrhage	Acute intracerebral haemorrhage severity level 4, without heart failure
Intracerebral haemorrhage	Acute intracerebral haemorrhage severity level 3, without heart failure
Intracerebral haemorrhage	Acute intracerebral haemorrhage severity level 5, without heart failure
Intracerebral haemorrhage	Chronic intracerebral haemorrhage severity level 1
Intracerebral haemorrhage	Asymptomatic chronic intracerebral haemorrhage
Intracerebral haemorrhage	Acute intracerebral haemorrhage severity level 2, with asymptomatic heart failure
Intracerebral haemorrhage	Acute intracerebral haemorrhage severity level 2, with mild heart failure
Intracerebral haemorrhage	Acute intracerebral haemorrhage severity level 2, with moderate heart failure
Intracerebral haemorrhage	Acute intracerebral haemorrhage severity level 2, with severe heart failure
Intracerebral haemorrhage	Acute intracerebral haemorrhage severity level 3, with asymptomatic heart failure
Intracerebral haemorrhage	Acute intracerebral haemorrhage severity level 3, with mild heart failure
Intracerebral haemorrhage	Acute intracerebral haemorrhage severity level 3, with moderate heart failure
Intracerebral haemorrhage	Acute intracerebral haemorrhage severity level 3, with severe heart failure
Intracerebral haemorrhage	Acute intracerebral haemorrhage severity level 4, with asymptomatic heart failure
Intracerebral haemorrhage	Acute intracerebral haemorrhage severity level 4, with mild heart failure
Intracerebral haemorrhage	Acute intracerebral haemorrhage severity level 4, with moderate heart failure
Intracerebral haemorrhage	Acute intracerebral haemorrhage severity level 4, with severe heart failure
Intracerebral haemorrhage	Acute intracerebral haemorrhage severity level 5, with asymptomatic heart failure

Intracerebral haemorrhage	Acute intracerebral haemorrhage severity level 5, with mild heart failure
Intracerebral haemorrhage	Acute intracerebral haemorrhage severity level 5, with moderate heart failure
Intracerebral haemorrhage	Acute intracerebral haemorrhage severity level 5, with severe heart failure
Intracerebral haemorrhage	Chronic intracerebral haemorrhage severity level 2, with asymptomatic heart failure
Intracerebral haemorrhage	Chronic intracerebral haemorrhage severity level 2, with mild heart failure
Intracerebral haemorrhage	Chronic intracerebral haemorrhage severity level 2, with moderate heart failure
Intracerebral haemorrhage	Chronic intracerebral haemorrhage severity level 2, with severe heart failure
Intracerebral haemorrhage	Chronic intracerebral haemorrhage severity level 2, without heart failure
Intracerebral haemorrhage	Chronic intracerebral haemorrhage severity level 3, with asymptomatic heart failure, no dementia
Intracerebral haemorrhage	Chronic intracerebral haemorrhage severity level 3, with mild heart failure, no dementia
Intracerebral haemorrhage	Chronic intracerebral haemorrhage severity level 3, with moderate heart failure, no dementia
Intracerebral haemorrhage	Chronic intracerebral haemorrhage severity level 3, with severe heart failure, no dementia
Intracerebral haemorrhage	Chronic intracerebral haemorrhage severity level 3, without heart failure, no dementia
Intracerebral haemorrhage	Chronic intracerebral haemorrhage severity level 4, with asymptomatic heart failure
Intracerebral haemorrhage	Chronic intracerebral haemorrhage severity level 4, with mild heart failure
Intracerebral haemorrhage	Chronic intracerebral haemorrhage severity level 4, with moderate heart failure
Intracerebral haemorrhage	Chronic intracerebral haemorrhage severity level 4, with severe heart failure
Intracerebral haemorrhage	Chronic intracerebral haemorrhage severity level 4, without heart failure
Intracerebral haemorrhage	Chronic intracerebral haemorrhage severity level 5, with asymptomatic heart failure, no dementia
Intracerebral haemorrhage	Chronic intracerebral haemorrhage severity level 5, with mild heart failure, no dementia
Intracerebral haemorrhage	Chronic intracerebral haemorrhage severity level 5, with moderate heart failure, no dementia
Intracerebral haemorrhage	Chronic intracerebral haemorrhage severity level 5, with severe heart failure, no dementia
Intracerebral haemorrhage	Chronic intracerebral haemorrhage severity level 5, without heart failure, no dementia
Intracerebral haemorrhage	Chronic intracerebral haemorrhage severity level 3, with asymptomatic heart failure, with mild dementia
Intracerebral haemorrhage	Chronic intracerebral haemorrhage severity level 3, with mild heart failure, with mild dementia
Intracerebral haemorrhage	Chronic intracerebral haemorrhage severity level 3, with moderate heart failure, with mild dementia
Intracerebral haemorrhage	Chronic intracerebral haemorrhage severity level 3, with severe heart failure, with mild dementia
Intracerebral haemorrhage	Chronic intracerebral haemorrhage severity level 3, without heart failure, with mild dementia
Intracerebral haemorrhage	Chronic intracerebral haemorrhage severity level 5, with asymptomatic heart failure, with mild dementia
Intracerebral haemorrhage	Chronic intracerebral haemorrhage severity level 5, with mild heart failure, with mild dementia
Intracerebral haemorrhage	Chronic intracerebral haemorrhage severity level 5, with moderate heart failure, with mild dementia

Intracerebral haemorrhage	Chronic intracerebral haemorrhage severity level 5, with severe heart failure, with mild dementia
Intracerebral haemorrhage	Chronic intracerebral haemorrhage severity level 5, without heart failure, with mild dementia
Intracerebral haemorrhage	Chronic intracerebral haemorrhage severity level 3, with asymptomatic heart failure, with moderate dementia
Intracerebral haemorrhage	Chronic intracerebral haemorrhage severity level 3, with mild heart failure, with moderate dementia
Intracerebral haemorrhage	Chronic intracerebral haemorrhage severity level 3, with moderate heart failure, with moderate dementia
Intracerebral haemorrhage	Chronic intracerebral haemorrhage severity level 3, with severe heart failure, with moderate dementia
Intracerebral haemorrhage	Chronic intracerebral haemorrhage severity level 3, without heart failure, with moderate dementia
Intracerebral haemorrhage	Chronic intracerebral haemorrhage severity level 5, with asymptomatic heart failure, with moderate dementia
Intracerebral haemorrhage	Chronic intracerebral haemorrhage severity level 5, with mild heart failure, with moderate dementia
Intracerebral haemorrhage	Chronic intracerebral haemorrhage severity level 5, with moderate heart failure, with moderate dementia
Intracerebral haemorrhage	Chronic intracerebral haemorrhage severity level 5, with severe heart failure, with moderate dementia
Intracerebral haemorrhage	Chronic intracerebral haemorrhage severity level 5, without heart failure, with moderate dementia
Intracerebral haemorrhage	Chronic intracerebral haemorrhage severity level 3, with asymptomatic heart failure, with severe dementia
Intracerebral haemorrhage	Chronic intracerebral haemorrhage severity level 3, with mild heart failure, with severe dementia
Intracerebral haemorrhage	Chronic intracerebral haemorrhage severity level 3, with moderate heart failure, with severe dementia
Intracerebral haemorrhage	Chronic intracerebral haemorrhage severity level 3, with severe heart failure, with severe dementia
Intracerebral haemorrhage	Chronic intracerebral haemorrhage severity level 3, without heart failure, with severe dementia
Intracerebral haemorrhage	Chronic intracerebral haemorrhage severity level 5, with asymptomatic heart failure, with severe dementia
Intracerebral haemorrhage	Chronic intracerebral haemorrhage severity level 5, with mild heart failure, with severe dementia
Intracerebral haemorrhage	Chronic intracerebral haemorrhage severity level 5, with moderate heart failure, with severe dementia
Intracerebral haemorrhage	Chronic intracerebral haemorrhage severity level 5, with severe heart failure, with severe dementia
Intracerebral haemorrhage	Chronic intracerebral haemorrhage severity level 5, without heart failure, with severe dementia
Ischaemic stroke	Acute ischemic stroke severity level 1
Ischaemic stroke	Acute ischemic stroke severity level 2, without heart failure
Ischaemic stroke	Acute ischemic stroke severity level 4, without heart failure
Ischaemic stroke	Acute ischemic stroke severity level 3, without heart failure
Ischaemic stroke	Acute ischemic stroke severity level 5, without heart failure
Ischaemic stroke	Chronic ischemic stroke severity level 1
Ischaemic stroke	Asymptomatic chronic ischemic stroke
Ischaemic stroke	Acute ischaemic stroke severity level 2, with asymptomatic heart failure
Ischaemic stroke	Acute ischaemic stroke severity level 2, with mild heart failure

Ischaemic stroke	Acute ischaemic stroke severity level 2, with moderate heart failure
Ischaemic stroke	Acute ischaemic stroke severity level 2, with severe heart failure
Ischaemic stroke	Acute ischaemic stroke severity level 3, with asymptomatic heart failure
Ischaemic stroke	Acute ischaemic stroke severity level 3, with mild heart failure
Ischaemic stroke	Acute ischaemic stroke severity level 3, with moderate heart failure
Ischaemic stroke	Acute ischaemic stroke severity level 3, with severe heart failure
Ischaemic stroke	Acute ischaemic stroke severity level 4, with asymptomatic heart failure
Ischaemic stroke	Acute ischaemic stroke severity level 4, with mild heart failure
Ischaemic stroke	Acute ischaemic stroke severity level 4, with moderate heart failure
Ischaemic stroke	Acute ischaemic stroke severity level 4, with severe heart failure
Ischaemic stroke	Acute ischaemic stroke severity level 5, with asymptomatic heart failure
Ischaemic stroke	Acute ischaemic stroke severity level 5, with mild heart failure
Ischaemic stroke	Acute ischaemic stroke severity level 5, with moderate heart failure
Ischaemic stroke	Acute ischaemic stroke severity level 5, with severe heart failure
Ischaemic stroke	Chronic ischaemic stroke severity level 2, with asymptomatic heart failure
Ischaemic stroke	Chronic ischaemic stroke severity level 2, with mild heart failure
Ischaemic stroke	Chronic ischaemic stroke severity level 2, with moderate heart failure
Ischaemic stroke	Chronic ischaemic stroke severity level 2, with severe heart failure
Ischaemic stroke	Chronic ischaemic stroke severity level 2, without heart failure
Ischaemic stroke	Chronic ischaemic stroke severity level 3, with asymptomatic heart failure, no dementia
Ischaemic stroke	Chronic ischaemic stroke severity level 3, with mild heart failure, no dementia
Ischaemic stroke	Chronic ischaemic stroke severity level 3, with moderate heart failure, no dementia
Ischaemic stroke	Chronic ischaemic stroke severity level 3, with severe heart failure, no dementia
Ischaemic stroke	Chronic ischaemic stroke severity level 3, without heart failure, no dementia
Ischaemic stroke	Chronic ischaemic stroke severity level 4, with asymptomatic heart failure
Ischaemic stroke	Chronic ischaemic stroke severity level 4, with mild heart failure
Ischaemic stroke	Chronic ischaemic stroke severity level 4, with moderate heart failure
Ischaemic stroke	Chronic ischaemic stroke severity level 4, with severe heart failure
Ischaemic stroke	Chronic ischaemic stroke severity level 4, without heart failure
Ischaemic stroke	Chronic ischaemic stroke severity level 5, without heart failure, no dementia
Ischaemic stroke	Chronic ischaemic stroke severity level 5, with asymptomatic heart failure, no dementia

Ischaemic stroke	Chronic ischaemic stroke severity level 5, with mild heart failure, no dementia
Ischaemic stroke	Chronic ischaemic stroke severity level 5, with moderate heart failure, no dementia
Ischaemic stroke	Chronic ischaemic stroke severity level 5, with severe heart failure, no dementia
Ischaemic stroke	Chronic ischaemic stroke severity level 3, with asymptomatic heart failure, with mild dementia
Ischaemic stroke	Chronic ischaemic stroke severity level 3, with mild heart failure, with mild dementia
Ischaemic stroke	Chronic ischaemic stroke severity level 3, with moderate heart failure, with mild dementia
Ischaemic stroke	Chronic ischaemic stroke severity level 3, with severe heart failure, with mild dementia
Ischaemic stroke	Chronic ischaemic stroke severity level 3, without heart failure, with mild dementia
Ischaemic stroke	Chronic ischaemic stroke severity level 5, without heart failure, with mild dementia
Ischaemic stroke	Chronic ischaemic stroke severity level 5, with asymptomatic heart failure, with mild dementia
Ischaemic stroke	Chronic ischaemic stroke severity level 5, with mild heart failure, with mild dementia
Ischaemic stroke	Chronic ischaemic stroke severity level 5, with moderate heart failure, with mild dementia
Ischaemic stroke	Chronic ischaemic stroke severity level 5, with severe heart failure, with mild dementia
Ischaemic stroke	Chronic ischaemic stroke severity level 3, with asymptomatic heart failure, with moderate dementia
Ischaemic stroke	Chronic ischaemic stroke severity level 3, with mild heart failure, with moderate dementia
Ischaemic stroke	Chronic ischaemic stroke severity level 3, with moderate heart failure, with moderate dementia
Ischaemic stroke	Chronic ischaemic stroke severity level 3, with severe heart failure, with moderate dementia
Ischaemic stroke	Chronic ischaemic stroke severity level 3, without heart failure, with moderate dementia
Ischaemic stroke	Chronic ischaemic stroke severity level 5, without heart failure, with moderate dementia
Ischaemic stroke	Chronic ischaemic stroke severity level 5, with asymptomatic heart failure, with moderate dementia
Ischaemic stroke	Chronic ischaemic stroke severity level 5, with mild heart failure, with moderate dementia
Ischaemic stroke	Chronic ischaemic stroke severity level 5, with moderate heart failure, with moderate dementia
Ischaemic stroke	Chronic ischaemic stroke severity level 5, with severe heart failure, with moderate dementia
Ischaemic stroke	Chronic ischaemic stroke severity level 3, with asymptomatic heart failure, with severe dementia
Ischaemic stroke	Chronic ischaemic stroke severity level 3, with mild heart failure, with severe dementia
Ischaemic stroke	Chronic ischaemic stroke severity level 3, with moderate heart failure, with severe dementia
Ischaemic stroke	Chronic ischaemic stroke severity level 3, with severe heart failure, with severe dementia
Ischaemic stroke	Chronic ischaemic stroke severity level 3, without heart failure, with severe dementia
Ischaemic stroke	Chronic ischaemic stroke severity level 5, without heart failure, with severe dementia
Ischaemic stroke	Chronic ischaemic stroke severity level 5, with asymptomatic heart failure, with severe dementia
Ischaemic stroke	Chronic ischaemic stroke severity level 5, with mild heart failure, with severe dementia

Ischaemic stroke	Chronic ischaemic stroke severity level 5, with moderate heart failure, with severe dementia
Ischaemic stroke	Chronic ischaemic stroke severity level 5, with severe heart failure, with severe dementia
Klinefelter syndrome	Borderline intellectual disability due to Klinefelter syndrome
Klinefelter syndrome	Mild intellectual disability due to Klinefelter syndrome
Klinefelter syndrome	Mild intellectual disability with infertility due to Klinefelter syndrome
Klinefelter syndrome	Borderline intellectual disability with infertility due to Klinefelter syndrome
Malaria	Moderate motor impairment due to malaria
Malaria	Moderate motor impairment with blindness due to malaria
Malaria	Moderate motor impairment with epilepsy due to malaria
Malaria	Moderate motor impairment with blindness and epilepsy due to malaria
Malaria	Moderate motor plus cognitive impairment with blindness due to malaria
Malaria	Moderate motor plus cognitive impairment with epilepsy due to malaria
Malaria	Moderate motor plus cognitive impairment with blindness and epilepsy due to malaria
Malaria	Severe motor impairment due to malaria
Malaria	Severe motor impairment with blindness due to malaria
Malaria	Severe motor impairment with epilepsy due to malaria
Malaria	Severe motor impairment with blindness and epilepsy due to malaria
Malaria	Severe motor plus cognitive impairment with blindness due to malaria
Malaria	Severe motor plus cognitive impairment with epilepsy due to malaria
Malaria	Severe motor plus cognitive impairment with blindness and epilepsy due to malaria
Meningitis	Epilepsy due to meningitis
Meningitis	Mild motor plus cognitive impairments due to meningitis
Meningitis	Moderately severe hearing loss due to meningitis
Meningitis	Profound hearing loss with ringing due to meningitis
Meningitis	Moderate motor impairment due to meningitis
Meningitis	Blindness due to meningitis
Meningitis	Moderate hearing loss with ringing due to meningitis
Meningitis	Profound hearing loss due to meningitis
Meningitis	Mild intellectual disability due to meningitis
Meningitis	Moderate hearing loss due to meningitis
Meningitis	Severe hearing loss with ringing due to meningitis

Meningitis	Borderline intellectual disability due to meningitis
Meningitis	Monocular distance vision loss due to meningitis
Meningitis	Severe motor plus cognitive impairments due to meningitis
Meningitis	Mild hearing loss with ringing due to meningitis
Meningitis	Mild motor impairment due to long term due to meningitis
Meningitis	Mild behavioural problems due to meningitis
Meningitis	Severe hearing loss due to meningitis
Meningitis	Complete hearing loss with ringing due to meningitis
Meningitis	Moderate motor plus cognitive impairments due to meningitis
Meningitis	Mild hearing loss due to meningitis
Meningitis	Moderately severe hearing loss with ringing due to meningitis
Meningitis	Complete hearing loss due to meningitis
Meningitis	Severe motor impairment due to meningitis
Meningitis	Moderate vision impairment due to meningitis
Meningitis	Severe vision impairment due to meningitis
Migraine	Asymptomatic medication overuse headache due to migraine
Migraine	Symptomatic medication overuse headache due to migraine
Migraine	Symptomatic probable migraine
Migraine	Asymptomatic probable migraine
Migraine	Symptomatic definite migraine
Migraine	Asymptomatic definite migraine
Motor neuron disease	Mild motor impairment, mild respiratory problems, and speech problems due to motor neuron disease
Motor neuron disease	Mild motor impairment, moderate respiratory problems, and speech problems due to motor neuron disease
Motor neuron disease	Mild motor impairment, severe respiratory problems, and speech problems due to motor neuron disease
Motor neuron disease	Mild motor impairment and speech problems due to motor neuron disease
Motor neuron disease	Moderate motor impairment, mild respiratory problems, and speech problems due to motor neuron disease
Motor neuron disease	Moderate motor impairment, moderate respiratory problems, and speech problems due to motor neuron disease
Motor neuron disease	Moderate motor impairment, severe respiratory problems, and speech problems due to motor neuron disease
Motor neuron disease	Severe motor impairment, mild respiratory problems, and speech problems due to motor neuron disease
Motor neuron disease	Severe motor impairment, moderate respiratory problems, and speech problems due to motor neuron disease
Motor neuron disease	Severe motor impairment, severe respiratory problems, and speech problems due to motor neuron disease

Motor neuron disease	Mild motor impairment and mild respiratory problems due to motor neuron disease
Motor neuron disease	Mild motor impairment and severe respiratory problems due to motor neuron disease
Motor neuron disease	Moderate motor impairment and mild respiratory problems due to motor neuron disease
Motor neuron disease	Moderate motor impairment and moderate respiratory problems due to motor neuron disease
Motor neuron disease	Moderate motor impairment and severe respiratory problems due to motor neuron disease
Motor neuron disease	Severe motor impairment and mild respiratory problems due to motor neuron disease
Motor neuron disease	Severe motor impairment and moderate respiratory problems due to motor neuron disease
Motor neuron disease	Severe motor impairment and severe respiratory problems due to motor neuron disease
Motor neuron disease	Moderate motor impairment and speech problems due to motor neuron disease
Motor neuron disease	Severe motor impairment and speech problems due to motor neuron disease
Motor neuron disease	Mild motor impairment due to motor neuron disease
Motor neuron disease	Moderate motor impairment due to motor neuron disease
Motor neuron disease	Severe motor impairment due to motor neuron disease
Motor neuron disease	Mild respiratory problems and speech problems due to motor neuron disease
Motor neuron disease	Moderate respiratory problems and speech problems due to motor neuron disease
Motor neuron disease	Severe respiratory problems and speech problems due to motor neuron disease
Motor neuron disease	Mild respiratory problems due to motor neuron disease
Motor neuron disease	Moderate respiratory problems due to motor neuron disease
Motor neuron disease	Severe respiratory problems due to motor neuron disease
Motor neuron disease	Speech problems due to motor neuron disease
Motor neuron disease	Diagnosis of motor neuron disease
Motor neuron disease	Mild motor impairment and moderate respiratory problems due to motor neuron disease
Multiple sclerosis	Mild multiple sclerosis
Multiple sclerosis	Moderate multiple sclerosis
Multiple sclerosis	Severe multiple sclerosis
Multiple sclerosis	Asymptomatic multiple sclerosis
Neonatal encephalopathy	Mild motor plus cognitive impairments due to neonatal encephalopathy due to birth asphyxia and trauma
Neonatal encephalopathy	Mild motor impairment due to neonatal encephalopathy due to birth asphyxia and trauma
Neonatal encephalopathy	Moderate motor plus cognitive impairment with blindness due to neonatal encephalopathy due to birth asphyxia and trauma
Neonatal encephalopathy	Moderate motor plus cognitive impairment with blindness and epilepsy due to neonatal encephalopathy due to birth asphyxia and trauma

Neonatal encephalopathy	Moderate motor plus cognitive impairment with epilepsy due to neonatal encephalopathy due to birth asphyxia and trauma
Neonatal encephalopathy	Severe motor plus cognitive impairment with blindness due to neonatal encephalopathy due to birth asphyxia and trauma
Neonatal encephalopathy	Severe motor plus cognitive impairment with blindness and epilepsy due to neonatal encephalopathy due to birth asphyxia and trauma
Neonatal encephalopathy	Severe motor plus cognitive impairment with epilepsy due to neonatal encephalopathy due to birth asphyxia and trauma
Neonatal encephalopathy	Moderate motor impairment due to neonatal encephalopathy due to birth asphyxia and trauma
Neonatal encephalopathy	Moderate motor impairment with blindness due to neonatal encephalopathy due to birth asphyxia and trauma
Neonatal encephalopathy	Moderate motor impairment with blindness and epilepsy due to neonatal encephalopathy due to birth asphyxia and trauma
Neonatal encephalopathy	Moderate motor impairment with epilepsy due to neonatal encephalopathy due to birth asphyxia and trauma
Neonatal encephalopathy	Severe motor impairment due to neonatal encephalopathy due to birth asphyxia and trauma
Neonatal encephalopathy	Severe motor impairment with blindness due to neonatal encephalopathy due to birth asphyxia and trauma
Neonatal encephalopathy	Severe motor impairment with blindness and epilepsy due to neonatal encephalopathy due to birth asphyxia and trauma
Neonatal encephalopathy	Severe motor impairment with epilepsy due to neonatal encephalopathy due to birth asphyxia and trauma
Neonatal jaundice	Moderate motor plus cognitive impairment with blindness due to haemolytic disease and other neonatal jaundice
Neonatal jaundice	Moderate motor plus cognitive impairment with blindness and epilepsy due to haemolytic disease and other neonatal jaundice
Neonatal jaundice	Moderate motor plus cognitive impairment with epilepsy due to haemolytic disease and other neonatal jaundice
Neonatal jaundice	Severe motor plus cognitive impairment with blindness due to haemolytic disease and other neonatal jaundice
Neonatal jaundice	Severe motor plus cognitive impairment with blindness and epilepsy due to haemolytic disease and other neonatal jaundice
Neonatal jaundice	Severe motor plus cognitive impairment with epilepsy due to haemolytic disease and other neonatal jaundice
Neonatal jaundice	Moderate motor impairment due to haemolytic disease and other neonatal jaundice
Neonatal jaundice	Moderate motor impairment with blindness due to haemolytic disease and other neonatal jaundice
Neonatal jaundice	Moderate motor impairment with blindness and epilepsy due to haemolytic disease and other neonatal jaundice
Neonatal jaundice	Moderate motor impairment with epilepsy due to haemolytic disease and other neonatal jaundice
Neonatal jaundice	Severe motor impairment severe due to haemolytic disease and other neonatal jaundice
Neonatal jaundice	Severe motor impairment with blindness due to haemolytic disease and other neonatal jaundice
Neonatal jaundice	Severe motor impairment with blindness and epilepsy due to haemolytic disease and other neonatal jaundice
Neonatal jaundice	Severe motor impairment with epilepsy due to haemolytic disease and other neonatal jaundice
Neonatal preterm birth	Moderate motor plus cognitive impairment with blindness due to neonatal preterm birth complications 28–32 wks
Neonatal preterm birth	Moderate motor plus cognitive impairment with blindness due to neonatal preterm birth complications <28 wks
Neonatal preterm birth	Moderate motor plus cognitive impairment with blindness due to neonatal preterm birth complications 32–36 wks
Neonatal preterm birth	Moderate motor plus cognitive impairment with blindness and epilepsy due to neonatal preterm birth complications 32–36 wks
Neonatal preterm birth	Moderate motor plus cognitive impairment with blindness and epilepsy due to neonatal preterm birth complications <28 wks

Neonatal preterm birth	Moderate motor plus cognitive impairment with blindness and epilepsy due to neonatal preterm birth complications 28–32 wks
Neonatal preterm birth	Moderate motor plus cognitive impairment with epilepsy due to neonatal preterm birth complications 32–36 wks
Neonatal preterm birth	Moderate motor plus cognitive impairment with epilepsy due to neonatal preterm birth complications <28 wks
Neonatal preterm birth	Moderate motor plus cognitive impairment with epilepsy due to neonatal preterm birth complications 28–32 wks
Neonatal preterm birth	Severe motor plus cognitive impairment with blindness due to neonatal preterm birth complications 28–32 wks
Neonatal preterm birth	Severe motor plus cognitive impairment with blindness due to neonatal preterm birth complications 32–36 wks
Neonatal preterm birth	Severe motor plus cognitive impairment with blindness due to neonatal preterm birth complications <28 wks
Neonatal preterm birth	Severe motor plus cognitive impairment with blindness and epilepsy due to neonatal preterm birth complications 28–32 wks
Neonatal preterm birth	Severe motor plus cognitive impairment with blindness and epilepsy due to neonatal preterm birth complications <28 wks
Neonatal preterm birth	Severe motor plus cognitive impairment with blindness and epilepsy due to neonatal preterm birth complications 32–36 wks
Neonatal preterm birth	Severe motor plus cognitive impairment with epilepsy due to neonatal preterm birth complications <28 wks
Neonatal preterm birth	Severe motor plus cognitive impairment with epilepsy due to neonatal preterm birth complications 32–36 wks
Neonatal preterm birth	Severe motor plus cognitive impairment with epilepsy due to neonatal preterm birth complications 28–32 wks
Neonatal preterm birth	Moderate motor impairment due to neonatal preterm birth complications 28–32 wks
Neonatal preterm birth	Moderate motor impairment due to neonatal preterm birth complications <28 wks
Neonatal preterm birth	Moderate motor impairment due to neonatal preterm birth complications 32–36 wks
Neonatal preterm birth	Moderate motor impairment with blindness due to neonatal preterm birth complications <28 wks
Neonatal preterm birth	Moderate motor impairment with blindness due to neonatal preterm birth complications 28–32 wks
Neonatal preterm birth	Moderate motor impairment with blindness due to neonatal preterm birth complications 32–36 wks
Neonatal preterm birth	Moderate motor impairment with blindness and epilepsy due to neonatal preterm birth complications 32–36 wks
Neonatal preterm birth	Moderate motor impairment with blindness and epilepsy due to neonatal preterm birth complications 28–32 wks
Neonatal preterm birth	Moderate motor impairment with blindness and epilepsy due to neonatal preterm birth complications <28 wks
Neonatal preterm birth	Moderate motor impairment with epilepsy due to neonatal preterm birth complications 32–36 wks
Neonatal preterm birth	Moderate motor impairment with epilepsy due to neonatal preterm birth complications 28–32 wks
Neonatal preterm birth	Moderate motor impairment with epilepsy due to neonatal preterm birth complications <28 wks
Neonatal preterm birth	Severe motor impairment due to neonatal preterm birth complications <28 wks
Neonatal preterm birth	Severe motor impairment due to neonatal preterm birth complications 32–36 wks
Neonatal preterm birth	Severe motor impairment due to neonatal preterm birth complications 28–32 wks
Neonatal preterm birth	Severe motor impairment with blindness due to neonatal preterm birth complications 28–32 wks
Neonatal preterm birth	Severe motor impairment with blindness due to neonatal preterm birth complications <28 wks
Neonatal preterm birth	Severe motor impairment with blindness due to neonatal preterm birth complications 32–36 wks

Neonatal preterm birth	Severe motor impairment with blindness and epilepsy due to neonatal preterm birth complications <28 wks
Neonatal preterm birth	Severe motor impairment with blindness and epilepsy due to neonatal preterm birth complications 28–32 wks
Neonatal preterm birth	Severe motor impairment with blindness and epilepsy due to neonatal preterm birth complications 32–36 wks
Neonatal preterm birth	Severe motor impairment with epilepsy due to neonatal preterm birth complications 32–36 wks
Neonatal preterm birth	Severe motor impairment with epilepsy due to neonatal preterm birth complications <28 wks
Neonatal preterm birth	Severe motor impairment with epilepsy due to neonatal preterm birth complications 28–32 wks
Neonatal preterm birth	Blindness due to retinopathy of prematurity
Neonatal preterm birth	Mild motor impairment due to neonatal preterm birth complications <28 wks
Neonatal preterm birth	Mild motor impairment due to neonatal preterm birth complications 28–32 wks
Neonatal preterm birth	Mild motor impairment due to neonatal preterm birth complications 32–36 wks
Neonatal preterm birth	Mild motor plus cognitive impairments due to neonatal preterm birth complications <28 wks
Neonatal preterm birth	Mild motor plus cognitive impairments due to neonatal preterm birth complications 28–32 wks
Neonatal preterm birth	Mild motor plus cognitive impairments due to neonatal preterm birth complications 32–36 wks
Neonatal preterm birth	Moderate vision impairment due to retinopathy of prematurity
Neonatal preterm birth	Severe vision impairment due to retinopathy of prematurity
Neonatal sepsis	Moderate motor impairment with epilepsy due to neonatal sepsis and other neonatal infections
Neonatal sepsis	Moderate motor impairment with blindness and epilepsy due to neonatal sepsis and other neonatal infections
Neonatal sepsis	Moderate motor plus cognitive impairment with epilepsy due to neonatal sepsis and other neonatal infections
Neonatal sepsis	Moderate motor plus cognitive impairment with blindness and epilepsy due to neonatal sepsis and other neonatal infections
Neonatal sepsis	Severe motor impairment with epilepsy due to neonatal sepsis and other neonatal infections
Neonatal sepsis	Severe motor impairment with blindness and epilepsy due to neonatal sepsis and other neonatal infections
Neonatal sepsis	Severe motor plus cognitive impairment with epilepsy due to neonatal sepsis and other neonatal infections
Neonatal sepsis	Severe motor plus cognitive impairment with blindness and epilepsy due to neonatal sepsis and other neonatal infections
Neonatal sepsis	Moderate motor impairment due to neonatal sepsis and other neonatal infections
Neonatal sepsis	Moderate motor impairment with blindness due to neonatal sepsis and other neonatal infections
Neonatal sepsis	Moderate motor plus cognitive impairment with blindness due to neonatal sepsis and other neonatal infections
Neonatal sepsis	Severe motor impairment due to neonatal sepsis and other neonatal infections
Neonatal sepsis	Severe motor impairment with blindness due to neonatal sepsis and other neonatal infections
Neonatal sepsis	Severe motor plus cognitive impairment with blindness due to neonatal sepsis and other neonatal infections
Neural tube defects	Severe motor and cognitive impairment due to anencephaly
Neural tube defects	Asymptomatic encephalocele following treatment

Neural tube defects	Borderline intellectual disability due to encephalocele
Neural tube defects	Mild intellectual disability due to encephalocele
Neural tube defects	Moderate intellectual disability due to encephalocele
Neural tube defects	Severe intellectual disability due to encephalocele
Neural tube defects	Profound intellectual disability due to encephalocele
Neural tube defects	Incontinence due to encephalocele
Neural tube defects	Borderline intellectual disability and incontinence due to encephalocele
Neural tube defects	Mild intellectual disability and incontinence due to encephalocele
Neural tube defects	Moderate intellectual disability and incontinence due to encephalocele
Neural tube defects	Severe intellectual disability and incontinence due to encephalocele
Neural tube defects	Profound intellectual disability and incontinence due to encephalocele
Neural tube defects	Mild motor impairment due to spina bifida
Neural tube defects	Moderate motor impairment due to spina bifida
Neural tube defects	Severe motor impairment due to spina bifida
Neural tube defects	Mild motor impairment and borderline intellectual disability due to spina bifida
Neural tube defects	Moderate motor impairment and borderline intellectual disability due to spina bifida
Neural tube defects	Severe motor impairment and borderline intellectual disability due to spina bifida
Neural tube defects	Mild motor impairment and mild intellectual disability due to spina bifida
Neural tube defects	Moderate motor impairment and mild intellectual disability due to spina bifida
Neural tube defects	Severe motor impairment and mild intellectual disability due to spina bifida
Neural tube defects	Mild motor impairment and moderate intellectual disability due to spina bifida
Neural tube defects	Moderate motor impairment and moderate intellectual disability due to spina bifida
Neural tube defects	Severe motor impairment and moderate intellectual disability due to spina bifida
Neural tube defects	Mild motor impairment and severe intellectual disability due to spina bifida
Neural tube defects	Moderate motor impairment and severe intellectual disability due to spina bifida
Neural tube defects	Severe motor impairment and severe intellectual disability due to spina bifida
Neural tube defects	Mild motor impairment, borderline intellectual disability, and incontinence due to spina bifida
Neural tube defects	Moderate motor impairment, borderline intellectual disability, and incontinence due to spina bifida
Neural tube defects	Severe motor impairment, borderline intellectual disability, and incontinence due to spina bifida
Neural tube defects	Mild motor impairment, mild intellectual disability, and incontinence due to spina bifida
Neural tube defects	Moderate motor impairment, mild intellectual disability, and incontinence due to spina bifida

Neural tube defects	Severe motor impairment, mild intellectual disability, and incontinence due to spina bifida
Neural tube defects	Mild motor impairment, moderate intellectual disability, and incontinence due to spina bifida
Neural tube defects	Moderate motor impairment, moderate intellectual disability, and incontinence due to spina bifida
Neural tube defects	Severe motor impairment, moderate intellectual disability, and incontinence due to spina bifida
Neural tube defects	Mild motor impairment, severe intellectual disability, and incontinence due to spina bifida
Neural tube defects	Moderate motor impairment, severe intellectual disability, and incontinence due to spina bifida
Neural tube defects	Severe motor impairment, severe intellectual disability, and incontinence due to spina bifida
Neural tube defects	Mild motor impairment and incontinence due to spina bifida
Neural tube defects	Moderate motor impairment and incontinence due to spina bifida
Neural tube defects	Severe motor impairment and incontinence due to spina bifida
Neural tube defects	Mild motor impairment and profound intellectual disability due to spina bifida
Neural tube defects	Moderate motor impairment and profound intellectual disability due to spina bifida
Neural tube defects	Severe motor impairment and profound intellectual disability due to spina bifida
Neural tube defects	Mild motor impairment, profound intellectual disability, and incontinence due to spina bifida
Neural tube defects	Moderate motor impairment, profound intellectual disability, and incontinence due to spina bifida
Neural tube defects	Severe motor impairment, profound intellectual disability, and incontinence due to spina bifida
Neural tube defects	Mild motor impairment due to encephalocele
Neural tube defects	Moderate motor impairment due to encephalocele
Neural tube defects	Severe motor impairment due to encephalocele
Neural tube defects	Mild motor impairment and incontinence due to encephalocele
Neural tube defects	Moderate motor impairment and incontinence due to encephalocele
Neural tube defects	Severe motor impairment and incontinence due to encephalocele
Neural tube defects	Mild motor impairment and borderline intellectual disability due to encephalocele
Neural tube defects	Moderate motor impairment and borderline intellectual disability due to encephalocele
Neural tube defects	Severe motor impairment and borderline intellectual disability due to encephalocele
Neural tube defects	Mild motor impairment and mild intellectual disability due to encephalocele
Neural tube defects	Moderate motor impairment and mild intellectual disability due to encephalocele
Neural tube defects	Severe motor impairment and mild intellectual disability due to encephalocele
Neural tube defects	Mild motor impairment and moderate intellectual disability due to encephalocele
Neural tube defects	Moderate motor impairment and moderate intellectual disability due to encephalocele
Neural tube defects	Severe motor impairment and moderate intellectual disability due to encephalocele

Neural tube defects	Mild motor impairment and severe intellectual disability due to encephalocele
Neural tube defects	Moderate motor impairment and severe intellectual disability due to encephalocele
Neural tube defects	Severe motor impairment and severe intellectual disability due to encephalocele
Neural tube defects	Mild motor impairment and profound intellectual disability due to encephalocele
Neural tube defects	Moderate motor impairment and profound intellectual disability due to encephalocele
Neural tube defects	Severe motor impairment and profound intellectual disability due to encephalocele
Neural tube defects	Mild motor impairment, borderline intellectual disability, and incontinence due to encephalocele
Neural tube defects	Moderate motor impairment, borderline intellectual disability, and incontinence due to encephalocele
Neural tube defects	Severe motor impairment, borderline intellectual disability, and incontinence due to encephalocele
Neural tube defects	Mild motor impairment, mild intellectual disability, and incontinence due to encephalocele
Neural tube defects	Moderate motor impairment, mild intellectual disability, and incontinence due to encephalocele
Neural tube defects	Severe motor impairment, mild intellectual disability, and incontinence due to encephalocele
Neural tube defects	Mild motor impairment, moderate intellectual disability, and incontinence due to encephalocele
Neural tube defects	Moderate motor impairment, moderate intellectual disability, and incontinence due to encephalocele
Neural tube defects	Severe motor impairment, moderate intellectual disability, and incontinence due to encephalocele
Neural tube defects	Mild motor impairment, severe intellectual disability, and incontinence due to encephalocele
Neural tube defects	Moderate motor impairment, severe intellectual disability, and incontinence due to encephalocele
Neural tube defects	Severe motor impairment, severe intellectual disability, and incontinence due to encephalocele
Neural tube defects	Mild motor impairment, profound intellectual disability, and incontinence due to encephalocele
Neural tube defects	Moderate motor impairment, profound intellectual disability, and incontinence due to encephalocele
Neural tube defects	Severe motor impairment, profound intellectual disability, and incontinence due to encephalocele
Neuroblastoma and other peripheral nervous cell tumours	Diagnosis and primary therapy phase of neuroblastoma and other peripheral nervous cell tumours
Neuroblastoma and other peripheral nervous cell tumours	Controlled phase of neuroblastoma and other peripheral nervous cell tumours
Neuroblastoma and other peripheral nervous cell tumours	Metastatic phase of neuroblastoma and other peripheral nervous cell tumours
Neuroblastoma and other peripheral nervous cell tumours	Terminal phase of neuroblastoma and other peripheral nervous cell tumours
Neurocysticercosis	Neurocysticercosis with epilepsy
Other chromosomal abnormalities	Borderline intellectual disability due to other chromosomal abnormalities
Other chromosomal abnormalities	Mild intellectual disability due to other chromosomal abnormalities
Other chromosomal abnormalities	Moderate intellectual disability due to other chromosomal abnormalities

Other chromosomal abnormalities	Severe intellectual disability due to other chromosomal abnormalities
Other chromosomal abnormalities	Profound intellectual disability due to other chromosomal abnormalities
Other chromosomal abnormalities	Borderline intellectual disability with congenital heart disease due to other chromosomal abnormalities
Other chromosomal abnormalities	Mild intellectual disability with congenital heart disease due to other chromosomal abnormalities
Other chromosomal abnormalities	Moderate intellectual disability with congenital heart disease due to other chromosomal abnormalities
Other chromosomal abnormalities	Severe intellectual disability with congenital heart disease due to other chromosomal abnormalities
Other chromosomal abnormalities	Profound intellectual disability with congenital heart disease due to other chromosomal abnormalities
Other chromosomal abnormalities	Mild dementia due to other chromosomal abnormalities
Other chromosomal abnormalities	Moderate dementia due to other chromosomal abnormalities
Other chromosomal abnormalities	Severe dementia due to other chromosomal abnormalities
Other chromosomal abnormalities	Congenital heart disease and mild dementia due to other chromosomal abnormalities
Other chromosomal abnormalities	Congenital heart disease and moderate dementia due to other chromosomal abnormalities
Other chromosomal abnormalities	Congenital heart disease and severe dementia due to other chromosomal abnormalities
Other chromosomal abnormalities	Borderline intellectual disability, mild dementia, and congenital heart disease due to other chromosomal abnormalities
Other chromosomal abnormalities	Mild intellectual disability, mild dementia, and congenital heart disease due to other chromosomal abnormalities
Other chromosomal abnormalities	Moderate intellectual disability, mild dementia, and congenital heart disease due to other chromosomal abnormalities
Other chromosomal abnormalities	Severe intellectual disability, mild dementia, and congenital heart disease due to other chromosomal abnormalities
Other chromosomal abnormalities	Profound intellectual disability, mild dementia, and congenital heart disease due to other chromosomal abnormalities
Other chromosomal abnormalities	Borderline intellectual disability, moderate dementia, and congenital heart disease due to other chromosomal abnormalities
Other chromosomal abnormalities	Mild intellectual disability, moderate dementia, and congenital heart disease due to other chromosomal abnormalities
Other chromosomal abnormalities	Moderate intellectual disability, moderate dementia, and congenital heart disease due to other chromosomal abnormalities
Other chromosomal abnormalities	Severe intellectual disability, moderate dementia, and congenital heart disease due to other chromosomal abnormalities
Other chromosomal abnormalities	Profound intellectual disability, moderate dementia, and congenital heart disease due to other chromosomal abnormalities
Other chromosomal abnormalities	Borderline intellectual disability, severe dementia, and congenital heart disease due to other chromosomal abnormalities
Other chromosomal abnormalities	Mild intellectual disability, severe dementia, and congenital heart disease due to other chromosomal abnormalities
Other chromosomal abnormalities	Moderate intellectual disability, severe dementia, and congenital heart disease due to other chromosomal abnormalities
Other chromosomal abnormalities	Severe intellectual disability, severe dementia, and congenital heart disease due to other chromosomal abnormalities
Other chromosomal abnormalities	Profound intellectual disability, severe dementia, and congenital heart disease due to other chromosomal abnormalities
Other chromosomal abnormalities	Profound intellectual disability and mild dementia due to other chromosomal abnormalities
Other chromosomal abnormalities	Severe intellectual disability and mild dementia due to other chromosomal abnormalities
Other chromosomal abnormalities	Moderate intellectual disability and mild dementia due to other chromosomal abnormalities

Other chromosomal abnormalities	Mild intellectual disability and mild dementia due to other chromosomal abnormalities
Other chromosomal abnormalities	Borderline intellectual disability and mild dementia due to other chromosomal abnormalities
Other chromosomal abnormalities	Profound intellectual disability and moderate dementia due to other chromosomal abnormalities
Other chromosomal abnormalities	Severe intellectual disability and moderate dementia due to other chromosomal abnormalities
Other chromosomal abnormalities	Moderate intellectual disability and moderate dementia due to other chromosomal abnormalities
Other chromosomal abnormalities	Mild intellectual disability and moderate dementia due to other chromosomal abnormalities
Other chromosomal abnormalities	Borderline intellectual disability and moderate dementia due to other chromosomal abnormalities
Other chromosomal abnormalities	Profound intellectual disability and severe dementia due to other chromosomal abnormalities
Other chromosomal abnormalities	Severe intellectual disability and severe dementia due to other chromosomal abnormalities
Other chromosomal abnormalities	Moderate intellectual disability and severe dementia due to other chromosomal abnormalities
Other chromosomal abnormalities	Mild intellectual disability and severe dementia due to other chromosomal abnormalities
Other chromosomal abnormalities	Borderline intellectual disability and severe dementia due to other chromosomal abnormalities
Other neurological disorders	Other neurological disorders
Parkinson’s disease	Mild Parkinson’s disease
Parkinson’s disease	Moderate Parkinson’s disease
Parkinson’s disease	Severe Parkinson’s disease
Rabies	Rabies
Spinal cord injury	Spinal cord lesion at neck level
Spinal cord injury	Spinal cord lesion below neck level
Subarachnoid haemorrhage	Acute subarachnoid haemorrhage severity level 1
Subarachnoid haemorrhage	Acute subarachnoid haemorrhage severity level 2, without heart failure
Subarachnoid haemorrhage	Acute subarachnoid haemorrhage severity level 3, without heart failure
Subarachnoid haemorrhage	Acute subarachnoid haemorrhage severity level 4, without heart failure
Subarachnoid haemorrhage	Acute subarachnoid haemorrhage severity level 5, without heart failure
Subarachnoid haemorrhage	Asymptomatic chronic subarachnoid haemorrhage
Subarachnoid haemorrhage	Chronic subarachnoid haemorrhage severity level 1
Subarachnoid haemorrhage	Acute subarachnoid haemorrhage severity level 2, with asymptomatic heart failure
Subarachnoid haemorrhage	Acute subarachnoid haemorrhage severity level 2, with mild heart failure
Subarachnoid haemorrhage	Acute subarachnoid haemorrhage severity level 2, with moderate heart failure
Subarachnoid haemorrhage	Acute subarachnoid haemorrhage severity level 2, with severe heart failure
Subarachnoid haemorrhage	Acute subarachnoid haemorrhage severity level 3, with asymptomatic heart failure

Subarachnoid haemorrhage	Acute subarachnoid haemorrhage severity level 3, with mild heart failure
Subarachnoid haemorrhage	Acute subarachnoid haemorrhage severity level 3, with moderate heart failure
Subarachnoid haemorrhage	Acute subarachnoid haemorrhage severity level 3, with severe heart failure
Subarachnoid haemorrhage	Acute subarachnoid haemorrhage severity level 4, with asymptomatic heart failure
Subarachnoid haemorrhage	Acute subarachnoid haemorrhage severity level 4, with mild heart failure
Subarachnoid haemorrhage	Acute subarachnoid haemorrhage severity level 4, with moderate heart failure
Subarachnoid haemorrhage	Acute subarachnoid haemorrhage severity level 4, with severe heart failure
Subarachnoid haemorrhage	Acute subarachnoid haemorrhage severity level 5, with asymptomatic heart failure
Subarachnoid haemorrhage	Acute subarachnoid haemorrhage severity level 5, with mild heart failure
Subarachnoid haemorrhage	Acute subarachnoid haemorrhage severity level 5, with moderate heart failure
Subarachnoid haemorrhage	Acute subarachnoid haemorrhage severity level 5, with severe heart failure
Subarachnoid haemorrhage	Chronic subarachnoid haemorrhage severity level 2, with asymptomatic heart failure
Subarachnoid haemorrhage	Chronic subarachnoid haemorrhage severity level 2, with mild heart failure
Subarachnoid haemorrhage	Chronic subarachnoid haemorrhage severity level 2, with moderate heart failure
Subarachnoid haemorrhage	Chronic subarachnoid haemorrhage severity level 2, with severe heart failure
Subarachnoid haemorrhage	Chronic subarachnoid haemorrhage severity level 2, without heart failure
Subarachnoid haemorrhage	Chronic subarachnoid haemorrhage severity level 3, with asymptomatic heart failure, no dementia
Subarachnoid haemorrhage	Chronic subarachnoid haemorrhage severity level 3, with mild heart failure, no dementia
Subarachnoid haemorrhage	Chronic subarachnoid haemorrhage severity level 3, with moderate heart failure, no dementia
Subarachnoid haemorrhage	Chronic subarachnoid haemorrhage severity level 3, with severe heart failure, no dementia
Subarachnoid haemorrhage	Chronic subarachnoid haemorrhage severity level 3, without heart failure, no dementia
Subarachnoid haemorrhage	Chronic subarachnoid haemorrhage severity level 4, with asymptomatic heart failure
Subarachnoid haemorrhage	Chronic subarachnoid haemorrhage severity level 4, with mild heart failure
Subarachnoid haemorrhage	Chronic subarachnoid haemorrhage severity level 4, with moderate heart failure
Subarachnoid haemorrhage	Chronic subarachnoid haemorrhage severity level 4, with severe heart failure
Subarachnoid haemorrhage	Chronic subarachnoid haemorrhage severity level 4, without heart failure
Subarachnoid haemorrhage	Chronic subarachnoid haemorrhage severity level 5, with asymptomatic heart failure, no dementia
Subarachnoid haemorrhage	Chronic subarachnoid haemorrhage severity level 5, with mild heart failure, no dementia
Subarachnoid haemorrhage	Chronic subarachnoid haemorrhage severity level 5, with moderate heart failure, no dementia
Subarachnoid haemorrhage	Chronic subarachnoid haemorrhage severity level 5, with severe heart failure, no dementia
Subarachnoid haemorrhage	Chronic subarachnoid haemorrhage severity level 5, without heart failure, no dementia

Subarachnoid haemorrhage	Chronic subarachnoid haemorrhage severity level 3, with asymptomatic heart failure, with mild dementia
Subarachnoid haemorrhage	Chronic subarachnoid haemorrhage severity level 3, with mild heart failure, with mild dementia
Subarachnoid haemorrhage	Chronic subarachnoid haemorrhage severity level 3, with moderate heart failure, with mild dementia
Subarachnoid haemorrhage	Chronic subarachnoid haemorrhage severity level 3, with severe heart failure, with mild dementia
Subarachnoid haemorrhage	Chronic subarachnoid haemorrhage severity level 3, without heart failure, with mild dementia
Subarachnoid haemorrhage	Chronic subarachnoid haemorrhage severity level 5, with asymptomatic heart failure, with mild dementia
Subarachnoid haemorrhage	Chronic subarachnoid haemorrhage severity level 5, with mild heart failure, with mild dementia
Subarachnoid haemorrhage	Chronic subarachnoid haemorrhage severity level 5, with moderate heart failure, with mild dementia
Subarachnoid haemorrhage	Chronic subarachnoid haemorrhage severity level 5, with severe heart failure, with mild dementia
Subarachnoid haemorrhage	Chronic subarachnoid haemorrhage severity level 5, without heart failure, with mild dementia
Subarachnoid haemorrhage	Chronic subarachnoid haemorrhage severity level 3, with asymptomatic heart failure, with moderate dementia
Subarachnoid haemorrhage	Chronic subarachnoid haemorrhage severity level 3, with mild heart failure, with moderate dementia
Subarachnoid haemorrhage	Chronic subarachnoid haemorrhage severity level 3, with moderate heart failure, with moderate dementia
Subarachnoid haemorrhage	Chronic subarachnoid haemorrhage severity level 3, with severe heart failure, with moderate dementia
Subarachnoid haemorrhage	Chronic subarachnoid haemorrhage severity level 3, without heart failure, with moderate dementia
Subarachnoid haemorrhage	Chronic subarachnoid haemorrhage severity level 5, with asymptomatic heart failure, with moderate dementia
Subarachnoid haemorrhage	Chronic subarachnoid haemorrhage severity level 5, with mild heart failure, with moderate dementia
Subarachnoid haemorrhage	Chronic subarachnoid haemorrhage severity level 5, with moderate heart failure, with moderate dementia
Subarachnoid haemorrhage	Chronic subarachnoid haemorrhage severity level 5, with severe heart failure, with moderate dementia
Subarachnoid haemorrhage	Chronic subarachnoid haemorrhage severity level 5, without heart failure, with moderate dementia
Subarachnoid haemorrhage	Chronic subarachnoid haemorrhage severity level 3, with asymptomatic heart failure, with severe dementia
Subarachnoid haemorrhage	Chronic subarachnoid haemorrhage severity level 3, with mild heart failure, with severe dementia
Subarachnoid haemorrhage	Chronic subarachnoid haemorrhage severity level 3, with moderate heart failure, with severe dementia
Subarachnoid haemorrhage	Chronic subarachnoid haemorrhage severity level 3, with severe heart failure, with severe dementia
Subarachnoid haemorrhage	Chronic subarachnoid haemorrhage severity level 3, without heart failure, with severe dementia
Subarachnoid haemorrhage	Chronic subarachnoid haemorrhage severity level 5, with asymptomatic heart failure, with severe dementia
Subarachnoid haemorrhage	Chronic subarachnoid haemorrhage severity level 5, with mild heart failure, with severe dementia
Subarachnoid haemorrhage	Chronic subarachnoid haemorrhage severity level 5, with moderate heart failure, with severe dementia
Subarachnoid haemorrhage	Chronic subarachnoid haemorrhage severity level 5, with severe heart failure, with severe dementia
Subarachnoid haemorrhage	Chronic subarachnoid haemorrhage severity level 5, without heart failure, with severe dementia
Syphilis	Neurological problems and cardiovascular complications due to adult tertiary syphilis

Syphilis	Severe disfigurement and neurological problems due to adult tertiary syphilis
Syphilis	Severe disfigurement, neurological problems, and cardiovascular complications due to adult tertiary syphilis
Syphilis	Neurological problems due to adult tertiary syphilis
Syphilis	Late symptomatic congenital syphilis, neurosyphilis
Syphilis	Late symptomatic congenital syphilis, unilateral hearing loss
Tension-type headache	Asymptomatic medication overuse headache due to tension-type headache
Tension-type headache	Symptomatic medication overuse headache due to tension-type headache
Tension-type headache	Symptomatic probable tension-type headache
Tension-type headache	Asymptomatic probable tension-type headache
Tension-type headache	Symptomatic definite tension-type headache
Tension-type headache	Asymptomatic definite tension-type headache
Tetanus	Moderate motor plus cognitive impairment with blindness due to neonatal tetanus
Tetanus	Moderate motor plus cognitive impairment with blindness and epilepsy due to neonatal tetanus
Tetanus	Moderate motor plus cognitive impairment with epilepsy due to neonatal tetanus
Tetanus	Severe motor plus cognitive impairment with blindness due to neonatal tetanus
Tetanus	Severe motor plus cognitive impairment with blindness and epilepsy due to neonatal tetanus
Tetanus	Severe motor plus cognitive impairment with epilepsy due to neonatal tetanus
Tetanus	Moderate motor impairment due to neonatal tetanus
Tetanus	Moderate motor impairment with blindness due to neonatal tetanus
Tetanus	Moderate motor impairment with blindness and epilepsy due to neonatal tetanus
Tetanus	Moderate motor impairment with epilepsy due to neonatal tetanus
Tetanus	Severe motor impairment due to neonatal tetanus
Tetanus	Severe motor impairment with blindness due to neonatal tetanus
Tetanus	Severe motor impairment with blindness and epilepsy due to neonatal tetanus
Tetanus	Severe motor impairment with epilepsy due to neonatal tetanus
Tetanus	Mild motor impairment due to neonatal tetanus
Tetanus	Mild motor plus cognitive impairments due to neonatal tetanus
Tetanus	Severe tetanus
Traumatic brain injury	Minor TBI
Traumatic brain injury	Moderate/severe TBI
Zika virus disease	Congenital Zika syndrome

Neurological outcomes of HIV

We could not directly isolate neurological outcomes of HIV, and therefore do not include HIV as one of the conditions explicitly quantified in this analysis. Outcomes such as polyneuropathy (ICD10 G62.9) or HIV-associated neurocognitive disorder (e.g. encephalopathy; ICD10 B22) are either not captured or fall under an HIV residual category in vital registration data mapping and thus cannot be included. Dementia due to HIV is captured in dementia, and thus is included in this analysis. However, opportunistic infections secondary to HIV, such as cryptococcal meningitis, are categorized under HIV, and not explicitly quantified as part of HIV (or TB) and therefore could not be added in the aggregations presented in this paper.

Supplemental methods table 10. Disability weights used to calculate YLDs

**Health state**	**Used for which condition(s)?**	**Lay description**	**Disability weight (95% UI)**
ADHD	ADHD	This person is hyperactive and has difficulty concentrating, remembering things, and completing tasks.	0.045 (0.028–0.066)
Asperger's syndrome and other ASDs	Autism spectrum disorder	This person has difficulty interacting with other people and is slow to understand or respond to questions. The person is often preoccupied with one thing and has some difficulty with basic daily activities.	0.104 (0.071–0.147)
Autism		This person has severe problems interacting with others and difficulty understanding simple questions or directions. The person has great difficulty with basic daily activities and becomes distressed by any change in routine.	0.262 (0.176–0.365)
Asymptomatic, but worry	Motor neuron disease	This person has a disease diagnosis that causes some worry but minimal interference with daily activities.	0.012 (0.006–0.023)
Behaviour problems, mild	Encephalitis, meningitis	This person is hyperactive and has difficulty concentrating, remembering things, and completing tasks.	0.045 (0.028–0.066)
Cancer, diagnosis and primary therapy	Nervous system cancer	This person has pain, nausea, fatigue, weight loss, and high anxiety.	0.288 (0.193–0.399)
Cancer, controlled phase		This person has chronic disease that requires medication every day and causes some worry but minimal interference with daily activities.	0.049 (0.031–0.072)
Cancer, metastatic		This person has severe pain, extreme fatigue, weight loss, and high anxiety.	0.451 (0.307–0.600)

Cancer, terminal phase, with medication		This person has lost a lot of weight and regularly uses strong medication to avoid constant pain. The person has no appetite, feels nauseous, and needs to spend most of the day in bed.	0.540 (0.377–0.687)
COVID-19, mild cognitive impairment (same as mild dementia)	COVID-19	This person has some trouble remembering recent events and finds it hard to concentrate and make decisions and plans. They may have slight to moderate difficulty engaging in community affairs, complicated hobbies, and intellectual interests.	0.069 (0.046–0.099)
COVID-19, severe cognitive impairment (same as severe dementia)	COVID-19	This person has complete memory loss, no longer recognises close family members, and requires help with all daily activities, including personal care.	0.449 (0.304–0.595)
Dementia, mild	Alzheimer's disease and other dementias, Down syndrome, other chromosomal abnormalities, stroke	This person has some trouble remembering recent events and finds it hard to concentrate and make decisions and plans. They may have slight to moderate difficulty engaging in community affairs, complicated hobbies, and intellectual interests.	0.069 (0.046–0.099)
Dementia, moderate		This person retains highly learned material, but has severe memory problems, is disoriented with respect to time and sometimes place. They are severely impaired in their ability to handle problems and make social judgements. They require assistance with daily activities, and only retain simple chores and hobbies.	0.377 (0.252–0.508)
Dementia, severe		The person has complete memory loss, no longer recognises close family members, and requires help with all daily activities, including personal care.	0.449 (0.304–0.595)
Diabetic neuropathy	Diabetes (neuropathy)	Has pain, tingling, and numbness in the arms, legs, hands, and feet. The person sometimes gets cramps and muscle weakness.	0.133 (0.089–0.187)
Diabetic neuropathy with diabetic foot		Has a sore foot that is swollen and causes some difficulty in walking.	0.150 (0.103–0.208)*
Diabetic neuropathy with treated amputation		Has lost part of one leg, leaving pain and tingling in the stump. The person has an artificial leg that helps in moving around.	0.167 (0.114–0.229)*

Diabetic neuropathy with untreated amputation		Has lost part of one leg, leaving pain and tingling in the stump. The person does not have an artificial leg, has frequent sores, and uses crutches.	0.282 (0.198–0.379)*
Encephalitis, acute	Encephalitis	This person has a high fever and pain, and feels very weak, which causes great difficulty with daily activities.	0.133 (0.088–0.19)
Epilepsy, less severe	Epilepsy, echinococcosis, encephalitis, malaria, meningitis, congenital Zika	This person has sudden seizures two to five times a year, with violent muscle contractions and stiffness, loss of consciousness, and loss of urine or bowel control.	0.263 (0.173–0.367)
Epilepsy, severe		This person has sudden seizures one or more times each month, with violent muscle contractions and stiffness, loss of consciousness, and loss of urine or bowel control. Between seizures, the person has memory loss and difficulty concentrating.	0.552 (0.375–0.71)
Epilepsy, treated		This person has a chronic disease that requires medication every day and causes some worry but minimal interference with daily activities.	0.049 (0.031–0.072)
Fetal alcohol syndrome, mild	Fetal alcohol syndrome	This person is a little slow in developing physically and mentally, which causes some difficulty in learning but no other difficulties in daily activities.	0.016 (0.008–0.03)
Fetal alcohol syndrome, moderate		This person is slow in developing physically and mentally, which causes some difficulty in daily activities.	0.056 (0.035–0.083)
Fetal alcohol syndrome, severe		This person is very slow in developing physically and mentally, which causes great difficulty in daily activities.	0.179 (0.119–0.257)
Headache, symptomatic, medication overuse, due to migraine or tension-type	Migraine, tension-type headache	This person has daily headaches, felt as dull pain and often lasting all day, with poor sleep, nausea, and fatigue. The person takes medicine for the headaches, which provides little relief but is needed to avoid having worse symptoms.	0.223 (0.146–0.313)
Headache, symptomatic, tension-type		This person has a moderate headache that also affects the neck, which causes difficulty in daily activities.	0.037 (0.022–0.057)

Headache, symptomatic, migraine		This person has severe, throbbing head pain and nausea that cause great difficulty in daily activities and sometimes confine the person to bed. Moving around, light, and noise make it worse.	0.441 (0.294–0.588)
Hearing loss, mild	Meningitis	This person has great difficulty hearing and understanding another person talking in a noisy place (for example, on an urban street).	0.01 (0.004–0.019)
Hearing loss, mild, with ringing	Meningitis	This person has great difficulty hearing and understanding another person talking in a noisy place (for example, on an urban street), and sometimes has annoying ringing in the ears.	0.021 (0.012–0.036)
Hearing loss, moderate	Meningitis	This person is unable to hear and understand another person talking in a noisy place (for example, on an urban street), and has difficulty hearing another person talking even in a quiet place or on the phone.	0.027 (0.015–0.042)
Hearing loss, moderate, with ringing	Meningitis	This person is unable to hear and understand another person talking in a noisy place (for example, on an urban street), and has difficulty hearing another person talking even in a quiet place or on the phone, and has annoying ringing in the ears for more than 5 minutes at a time, almost every day.	0.074 (0.048–0.107)
Hearing loss, moderately severe	Meningitis	(custom DW from hearing loss impairment envelope)	
Hearing loss, moderately severe, with ringing	Meningitis	(custom DW from hearing loss impairment envelope)	
Hearing loss, severe	Meningitis	This person is unable to hear and understand another person talking, even in a quiet place, and unable to take part in a phone conversation. Difficulties with communicating and relating to others cause emotional impact at times (for example worry or depression).	0.158 (0.105–0.227)
Hearing loss, severe, with ringing	Meningitis	This person is unable to hear and understand another person talking, even in a quiet place, is unable to take part in a phone conversation, and has annoying ringing in the ears for more than 5 minutes at a time, almost every day. Difficulties with communicating and relating to others cause emotional impact at times (for example worry or depression).	0.261 (0.175–0.36)
Hearing loss, profound	Meningitis	This person is unable to hear and understand another person talking, even in a quiet place, is unable to take part in a phone conversation, and has great difficulty hearing anything in any other situation. Difficulties with	0.204 (0.134–0.288)

		communicating and relating to others often cause worry, depression, or loneliness.	
Hearing loss, profound, with ringing	Meningitis	This person is unable to hear and understand another person, even in a quiet place, is unable to take part in a phone conversation, has great difficulty hearing anything in any other situation, and has annoying ringing in the ears for more than 5 minutes at a time, several times a day. Difficulties with communicating and relating to others often cause worry, depression, or loneliness.	0.277 (0.182–0.387)
Hearing loss, complete	Meningitis	This person cannot hear at all in any situation, including even the loudest sounds, and cannot communicate verbally or use a phone. Difficulties with communicating and relating to others often cause worry, depression, or loneliness.	0.215 (0.144–0.307)
Hearing loss, complete, with ringing	Meningitis	This person cannot hear at all in any situation, including even the loudest sounds, and cannot communicate verbally or use a phone, and has very annoying ringing in the ears for more than half of the day. Difficulties with communicating and relating to others often cause worry, depression, or loneliness.	0.316 (0.212–0.435)
Infectious disease, acute episode, severe	Rabies	This person has a high fever and pain and feels very weak, which causes great difficulty with daily activities.	0.133 (0.088–0.190)
Intellectual disability, borderline	Idiopathic intellectual disability, neural tube defects, Down syndrome, other chromosomal abnormalities, congenital birth defects, encephalitis, meningitis, Klinefelter syndrome	This person is slow in learning at school. As an adult, the person has some difficulty doing complex or unfamiliar tasks but otherwise functions independently.	0.011 (0.005–0.020)
Intellectual disability, mild	Idiopathic intellectual disability, autism spectrum disorder, neural tube defects, Down syndrome, other chromosomal	This person has low intelligence and is slow in learning at school. As an adult, the person can live independently, but often needs help to raise children and can only work at simple supervised jobs.	0.043 (0.026–0.064)

	abnormalities, congenital birth defects, encephalitis, meningitis, Klinefelter syndrome		
Intellectual disability, moderate	Idiopathic intellectual disability, autism spectrum disorder, neural tube defects, Down syndrome, other chromosomal abnormalities, congenital birth defects, encephalitis, meningitis	This person has low intelligence and is slow in learning to speak and to do even simple tasks. As an adult, the person requires a lot of support to live independently and raise children. The person can only work at the simplest supervised jobs.	0.100 (0.066–0.142)
Intellectual disability, severe	Idiopathic intellectual disability, autism spectrum disorder, neural tube defects, Down syndrome, other chromosomal abnormalities, congenital birth defects, encephalitis, meningitis	This person has very low intelligence and cannot speak more than a few words, needs constant supervision and help with most daily activities, and can do only the simplest tasks.	0.160 (0.107–0.226)
Intellectual disability, profound	Idiopathic intellectual disability, autism spectrum disorder, neural tube defects, Down syndrome, other chromosomal abnormalities, congenital birth defects, encephalitis, meningitis	This person has very low intelligence, has almost no language, and does not understand even the most basic requests or instructions. The person requires constant supervision and help for all activities.	0.200 (0.133–0.283)
Motor impairment, mild, long-term	Encephalitis, meningitis, motor neuron disease, preterm birth, neonatal	This person has some difficulty in moving around but is able to walk without help.	0.01 (0.005–0.020)

	encephalopathy, neonatal jaundice, neonatal sepsis, neural tube defects		
Motor impairment, moderate	Encephalitis, malaria, meningitis, motor neuron disease, preterm birth, neonatal encephalopathy, neonatal jaundice, neonatal sepsis, neural tube defects	This person has some difficulty in moving around, and difficulty in lifting and holding objects, dressing, and sitting upright, but is able to walk without help.	0.061 (0.040–0.089)
Motor impairment, severe	Encephalitis, malaria, meningitis, motor neuron disease, preterm birth, neonatal encephalopathy, neonatal jaundice, neonatal sepsis, neural tube defects	This person is unable to move around without help, and is not able to lift or hold objects, get dressed, or sit upright.	0.40 (0.27–0.55)
Motor plus cognitive impairment, mild	Encephalitis, meningitis, preterm birth, neonatal encephalopathy, neonatal jaundice, neonatal sepsis	This person has some difficulty in moving around but is able to walk without help. The person is slow in learning at school. As an adult, the person has some difficulty doing complex or unfamiliar tasks but otherwise functions independently.	0.031 (0.018–0.050)
Motor plus cognitive impairment, moderate	Encephalitis, malaria, meningitis, preterm birth, neonatal encephalopathy, neonatal jaundice, neonatal sepsis	This person has some difficulty in moving around, holding objects, dressing, and sitting upright, but can walk without help. This person has low intelligence and is slow in learning to speak and to do simple tasks.	0.20 (0.13–0.29)
Motor plus cognitive impairment, severe	Encephalitis, meningitis, preterm birth, neonatal encephalopathy, neonatal jaundice, neonatal sepsis, congenital Zika	This person cannot move around without help, and cannot lift or hold objects, get dressed, or sit upright. The person also has very low intelligence, speaks few words, and needs constant supervision and help with all daily activities.	0.54 (0.37–0.70)
Multiple sclerosis, mild	Multiple sclerosis	This person has mild loss of feeling in one hand, is a little unsteady while walking, has slight loss of vision in one eye, and often needs to urinate urgently.	0.183 (0.124–0.253)

Multiple sclerosis, moderate	Multiple sclerosis	This person needs help walking, has difficulty with writing and arm coordination, has loss of vision in one eye, and cannot control urinating.	0.463 (0.313–0.613)
Multiple sclerosis, severe	Multiple sclerosis	This person has slurred speech and difficulty swallowing. The person has weak arms and hands, very limited and stiff leg movement, has loss of vision in both eyes, and cannot control urinating.	0.719 (0.534–0.858)
Parkinson's disease, mild	Parkinson's disease	This person has mild tremors and moves a little slowly, which causes some difficulty in walking and daily activities. The person has some trouble swallowing, talking, sleeping, and remembering things.	0.01 (0.005–0.019)
Parkinson's disease, moderate	Parkinson's disease	This person has moderate tremors and moves slowly, which causes some difficulty in walking and daily activities. The person has some trouble swallowing, talking, sleeping, and remembering things.	0.267 (0.181–0.372)
Parkinson's disease, severe	Parkinson's disease	This person has severe tremors and moves very slowly, which causes great difficulty in walking and daily activities. The person falls easily and has a lot of difficulty talking, swallowing, sleeping, and remembering things	0.575 (0.396–0.730)
Respiratory problems, mild	Motor neuron disease	This person has cough and shortness of breath after heavy physical activity but is able to walk long distances and climb stairs.	0.019 (0.011–0.033)
Respiratory problems, moderate	Motor neuron disease	This person has cough, wheezing, and shortness of breath, even after light physical activity. The person feels tired and can walk only short distances or climb only a few stairs.	0.225 (0.153–0.31)
Respiratory problems, severe	Motor neuron disease	This person has cough, wheezing, and shortness of breath all the time. The person has great difficulty walking even short distances or climbing any stairs, feels tired when at rest, and is anxious.	0.408 (0.273–0.556)
Speech problems	Motor neuron disease	This person has difficulty speaking, and others find it difficult to understand.	0.051 (0.032–0.078)
Spinal cord lesion, at neck level, treated	Spinal cord injury	This person is paralyzed from the neck down, with no feeling or control over any part of the body below the neck, and no urine or bowel control.	0.589 (0.415–0.748)
Spinal cord lesion, at neck level, untreated	Spinal cord injury	This person is paralyzed from the neck down, with no feeling or control over any part of the body below the neck, and no urine or bowel control. Arms and legs are in fixed, bent positions, and the person gets frequent infections and pressure sores.	0.732 (0.544–0.871)

Spinal cord lesion, below neck level, treated	Guillain-Barré syndrome, spinal cord injury	This person is paralyzed from the waist down, cannot feel or move the legs, and has difficulties with urine and bowel control. This person uses a wheelchair to move around.	0.296 (0.198–0.414)
Spinal cord lesion, below neck level, untreated	Spinal cord injury	This person is paralyzed from the waist down, cannot feel or move the legs, and has difficulties with urine and bowel control. Legs are in fixed, bent positions, and the person gets frequent infections and pressure sores.	0.623 (0.434–0.777)
Stroke, mild	Stroke	This person has some difficulty in moving around and some weakness in one hand but is able to walk without help.	0.019 (0.010–0.032)
Stroke, moderate, with no heart failure	Stroke	This person has some difficulty in moving around and in using the hands for lifting and holding things, dressing, and grooming.	0.070 (0.046–0.099)
Stroke, acute, moderate, with controlled, medically managed heart failure	Stroke	This person has some difficulty in moving around and in using the hands for lifting and holding things, dressing, and grooming. Has been diagnosed with clinical heart failure, a chronic disease that requires medication every day and causes some worry but minimal interference with daily activities.	0.116 (0.076–0.164)
Stroke, chronic, moderate, with controlled, medically managed heart failure	Stroke	This person has some difficulty in moving around and in using the hands for lifting and holding things, dressing, and grooming. Has been diagnosed with clinical heart failure, a chronic disease that requires medication every day and causes some worry but minimal interference with daily activities.	0.082 (0.053–0.118)
Stroke, acute, moderate, with mild heart failure	Stroke	This person has some difficulty in moving around and in using the hands for lifting and holding things, dressing, and grooming. Is short of breath and easily tires with moderate physical activity, such as walking uphill or more than a quarter mile on level ground. The person feels comfortable at rest or during activities requiring less effort.	0.108 (0.074–0.154)
Stroke, moderate, with moderate heart failure	Stroke	This person has some difficulty in moving around, and in using the hands for lifting and holding things, dressing, and grooming. Is short of breath and easily tires with minimal physical activity, such as walking only a short distance. The person feels comfortable at rest but avoids moderate activity.	0.137 (0.091–0.191)

Stroke, moderate, with severe heart failure	Stroke	This person has some difficulty in moving around and in using the hands for lifting and holding things, dressing, and grooming. Is short of breath and feels tired when at rest. The person avoids any physical activity, for fear of worsening the breathing problems.	0.236 (0.165–0.319)
Stroke, moderate plus cognition, with no heart failure	Stroke	This person has some difficulty in moving around, in using the hands for lifting and holding things, dressing, and grooming, and in speaking. The person is often forgetful and confused.	0.316 (0.206–0.437)
Stroke, acute, moderate plus cognition problems, with controlled, medically managed heart failure	Stroke	This person has some difficulty in moving around, in using the hands for lifting and holding things, dressing, and grooming, and in speaking. The person is often forgetful and confused. Has been diagnosed with clinical heart failure, a chronic disease that requires medication every day and causes some worry but minimal interference with daily activities.	0.349 (0.241–0.470)
Stroke, chronic, moderate plus cognition problems, with controlled, medically managed heart failure	Stroke	This person has some difficulty in moving around, in using the hands for lifting and holding things, dressing, and grooming, and in speaking. The person is often forgetful and confused. Has been diagnosed with clinical heart failure, a chronic disease that requires medication every day and causes some worry but minimal interference with daily activities.	0.325 (0.219–0.443)
Stroke, moderate plus cognition problems, with mild heart failure	Stroke	This person has some difficulty in moving around, in using the hands for lifting and holding things, dressing, and grooming, and in speaking. The person is often forgetful and confused. Is short of breath and easily tires with moderate physical activity, such as walking uphill or more than a quarter mile on level ground. The person feels comfortable at rest or during activities requiring less effort.	0.344 (0.237–0.464)
Stroke, moderate plus cognition problems, with moderate heart	Stroke	This person has some difficulty in moving around, in using the hands for lifting and holding things, dressing, and grooming, and in speaking. The person is often forgetful and confused. Is short of breath and easily tires with minimal	0.365 (0.253–0.487)

failure, no dementia		physical activity, such as walking only a short distance. The person feels comfortable at rest but avoids moderate activity.	
Stroke, moderate plus cognition problems, with severe heart failure	Stroke	This person has some difficulty in moving around, in using the hands for lifting and holding things, dressing, and grooming, and in speaking. The person is often forgetful and confused. Is short of breath and feels tired when at rest. The person avoids any physical activity, for fear of worsening the breathing problems.	0.437 (0.308–0.575)
Stroke, severe, with no heart failure	Stroke	Is confined to bed or a wheelchair, has difficulty speaking, and depends on others for feeding, toileting, and dressing.	0.552 (0.377–0.707)
Stroke, acute, severe, with controlled, medically managed heart failure	Stroke	This person is confined to bed or a wheelchair, has difficulty speaking, and depends on others for feeding, toileting, and dressing. Has been diagnosed with clinical heart failure, a chronic disease that requires medication every day and causes some worry but minimal interference with daily activities.	0.573 (0.408–0.720)
Stroke, chronic, severe, with asymptomatic heart failure	Stroke	This person is confined to bed or a wheelchair, has difficulty speaking, and depends on others for feeding, toileting, and dressing. Has been diagnosed with clinical heart failure, a chronic disease that requires medication every day and causes some worry but minimal interference with daily activities.	0.558 (0.389–0.711)
Stroke, severe, with mild heart failure	Stroke	This person is confined to bed or a wheelchair, has difficulty speaking, and depends on others for feeding, toileting, and dressing. Is short of breath and easily tires with moderate physical activity, such as walking uphill or more than a quarter mile on level ground. The person feels comfortable at rest or during activities requiring less effort.	0.570 (0.404–0.720)
Stroke, severe, with moderate heart failure	Stroke	This person is confined to bed or a wheelchair, has difficulty speaking, and depends on others for feeding, toileting, and dressing. Is short of breath and easily tires with minimal physical activity, such as walking only a short distance. The person feels comfortable at rest but avoids moderate activity.	0.584 (0.417–0.732)
Stroke, severe, with severe heart failure	Stroke	This person is confined to bed or a wheelchair, has difficulty speaking, and depends on others for feeding, toileting, and dressing. Is short of breath and feels tired when at rest. The person avoids any physical activity, for fear of worsening the breathing problems.	0.630 (0.458–0.777)

Stroke, severe plus cognition problems, no heart failure	Stroke	This person is confined to bed or a wheelchair, depends on others for feeding, toileting, and dressing, and has difficulty speaking, thinking clearly, and remembering things.	0.593 (0.421–0.747)
Stroke, acute, severe plus cognition problems, controlled, medically managed heart failure	Stroke	This person is confined to bed or a wheelchair, depends on others for feeding, toileting, and dressing, and has difficulty speaking, thinking clearly, and remembering things. Has been diagnosed with clinical heart failure, a chronic disease that requires medication every day and causes some worry but minimal interference with daily activities.	0.608 (0.438–0.759)
Stroke, chronic, severe plus cognition problems, asymptomatic heart failure	Stroke	This person is confined to bed or a wheelchair, depends on others for feeding, toileting, and dressing, and has difficulty speaking, thinking clearly, and remembering things. Has been diagnosed with clinical heart failure, a chronic disease that requires medication every day and causes some worry but minimal interference with daily activities.	0.593 (0.421–0.747)
Stroke, severe plus cognition problems, mild heart failure	Stroke	This person is confined to bed or a wheelchair, depends on others for feeding, toileting, and dressing, and has difficulty speaking, thinking clearly, and remembering things. Is short of breath and easily tires with moderate physical activity, such as walking uphill or more than a quarter-mile on level ground. The person feels comfortable at rest or during activities requiring less effort.	0.605 (0.436–0.758)
Stroke, severe plus cognition problems, moderate heart failure	Stroke	This person is confined to bed or a wheelchair, depends on others for feeding, toileting, and dressing, and has difficulty speaking, thinking clearly, and remembering things. Is short of breath and easily tires with minimal physical activity, such as walking only a short distance. The person feels comfortable at rest but avoids moderate activity.	0.617 (0.448–0.768)
Stroke, severe plus cognition problems, severe heart failure	Stroke	This person is confined to bed or a wheelchair, depends on others for feeding, toileting, and dressing, and has difficulty speaking, thinking clearly, and remembering things. Is short of breath and feels tired when at rest. The person avoids any physical activity, for fear of worsening the breathing problems.	0.659 (0.488–0.808)

Syphilis, adult tertiary, neurological problems	Syphilis	This person has some difficulty in moving around, holding objects, dressing, and sitting upright, but can walk without help. The person has low intelligence and is slow in learning to speak and to do simple tasks.	0.203 (0.134–0.290)
Syphilis, congenital, neurosyphilis	Syphilis	This person has some difficulty in moving around, holding objects, dressing, and sitting upright, but can walk without help. The person has low intelligence and is slow in learning to speak and to do simple tasks.	0.203 (0.134–0.290)
Syphilis, congenital, unilateral hearing loss	Syphilis	This person can hear well with one ear but has hearing loss in the other ear, resulting in some trouble following a conversation in a noisy environment.	0.008 (0.003–0.020)
Tetanus	Tetanus	This person has a high fever and pain, and feels very weak, which causes great difficult with daily activities.	0.133 (0.088–0.19)
Traumatic brain injury, mild, long-term	Traumatic brain injury	Combined disability weight with components of headaches, dizziness, nausea, difficulty concentrating, anxiety/moodiness, dependencies on others for feeding, toileting, dressing, walking.	0.132 (0.090–0.182)
Traumatic brain injury, moderate or severe, long-term	Traumatic brain injury	Combined disability weight with components of headaches, dizziness, nausea, difficulty concentrating, anxiety/moodiness, dependencies on others for feeding, toileting, dressing, walking.	0.164 (0.112–0.226)
Urinary incontinence	Neural tube defects	This person cannot control urinating.	0.139 (0.094–0.198)
Vision loss, moderate	Encephalitis, meningitis	This person has vision problems that make it difficult to recognise faces or objects across a room.	0.031 (0.019–0.049)
Vision loss, severe	Encephalitis, meningitis	This person has severe vision loss, which causes difficulty in daily activities, some emotional impact (for example worry), and some difficulty going outside the home without assistance.	0.18 (0.13–0.26)
Vision loss, blindness	Encephalitis, malaria, meningitis	This person is completely blind, which causes great difficulty in some daily activities, worry and anxiety, and great difficulty going outside the home without assistance.	0.19 (0.12–0.26)
Vision loss, monocular	Encephalitis, meningitis	This person is blind in one eye and has difficulty judging distances.	0.017 (0.009–0.029)

Asymptomatic health states without worry are not included because the disability weight is 0. Custom disability weights for combined health states are not displayed here.

*Disability weights for these diabetes sequela are produced from a combination of two health states: neuropathy and diabetic foot/amputation.

Analysis of Guillain-Barré Syndrome due to COVID-19

Data demonstrating an increase in GBS occurrence due to COVID-19 come from the United States PRA health services medical record database (https://www.iconplc.com/). The occurrence of GBS following COVID-19 was quantified as the difference in the incidence of GBS in COVID-19 cases and controls in the PRA database, matched by age, sex, race, month of COVID diagnosis or outpatient visit, and previous diagnosis of cancer, diabetes, heart failure, and/or stroke. This analysis produced over one million matched pairs, a large sample size that was beneficial given GBS has a low incidence rate. Data were tabulated based on COVID-19 case ascertainment as either community cases, hospitalized cases, or ICU cases. Risk of GBS increased with severity of COVID infection. The main limitations of this approach include: (1) the risk analysis used data solely from the U.S. but was applied globally; (2) cases were from the first wave of the pandemic but the risk was applied to the whole pandemic regardless of COVID variant or vaccination history; and (3) the analysis did not control for other recent infection that could lead to GBS such as diarrheal disease or influenza.

Data types and modelling methods to estimate deaths

For most conditions, fatal data consisted primarily of vital registration data, along with available verbal autopsy, registry, mortuary, hospital, police, and census data from 1980 onward. Cause of death data categorised by ICD or other country-specific classification systems were mapped to individual diseases within the GBD. Broad category codes (eg, "disorder of central nervous system, unspecified") or codes for diseases not considered underlying causes of death (eg, headache) were redistributed as previously described for GBD.[8] A misdiagnosis correction was applied to account for under-coding of certain diseases including atrial fibrillation, dementia, and Parkinson's disease, and noise reduction was used to smooth stochastic temporal variation due to small sample size (Section 2.6 and 2.7 of reference 8, Appendix 1).[13]

An ensemble modelling approach was used to estimate deaths for each condition.[16] Input data and predictive covariates were used to create component models. The predictive covariates included in each model are summarised in supplemental methods table 12. Model performance was tested with 30% holdouts of input data, and models with the highest out-of-sample predictive validity were retained and ultimately combined into a weighted ensemble used to calculate deaths by age, year, sex, and location.

Supplemental methods table 11. Source counts in fatal models

**Condition**	**Vital registration and other death data**	**Number of countries**
Alzheimer's disease and other dementias†	476	54
Encephalitis	3719	147
Idiopathic epilepsy	3560	148
Meningitis	4090	163
Motor neuron disease	3398	124
Multiple sclerosis	3634	125
Nervous system cancers*	6347	160
Neural tube defects	3161	151
Neurocysticercosis	3354	122

Other neurological disorders	2823	127
Parkinson's disease	3437	129
Rabies	3700	147
Stroke (any)	4017	152
Tetanus	4075	160

*Nervous system cancers refers to the combination of "Brain and central nervous system cancer" and "Neuroblastoma and other peripheral nervous cell tumours"; †data come from non-fatal modelling, which is used to inform fatal estimates.

Supplemental methods table 12. ICD-9 and ICD-10 fatal coding for each condition

**Condition**	**ICD-9**	**ICD-10**
Alzheimer's disease and other dementias	290, 291.2, 291.8, 294, 331	F00, F01, F02, F03, G30, G31
Encephalitis	062, 064.9, 139.0, 323	A83-A86.4, B94.1, F07.1, G04-G05.8, G21.3
Epilepsy	345	G40, G41
Meningitis	036, 047, 049.9, 320.0-320.3, 320.5-320.89, 321-322.9	A39, A87, D86.81, G00, G03
Motor neuron disease	335	G12
Multiple sclerosis	340	G35
Neonatal encephalopathy	761.7-761.9, 762, 763, 767, 768, 768.2-768.7, 768.9, 770.1, 779.0-779.2	P01.7, P02, P03, P10, P11, P12, P13, P14, P15, P20, P21, P24, P91
Nervous system cancers*	191, 191.0, 191.1, 191.2, 191.3, 191.4, 191.5, 191.6, 191.7, 191.8, 191.9, 192, 192.0, 192.1, 192.2 192.3, 192.8, 192.9, 194.3, 194.4, 194‡, 194.0‡, and 194.9‡	C47, C47.0, C47.1, C47.10. C47.11, C47.12, C47.2, C47.20, C47.21, C47.22, C47.3, C47.4, C47.5, C47.6, C47.8, C47.9, C47.90, C70, C70.0, C70.1, C70.5, C70.6, C70.9, C71, C71.0, C71.1, C71.2, C71.3, C71.4, C71.5, C71.6, C71.7, C71.8, C71.9, C72, C72.0, C72.1, C72.2, C72.20, C72.21, C72.22, C72.3, C72.30, C72.31, C72.32, C72.4, C72.40, C72.41, C72.42, C72.5, C72.50, C72.59, C72.8, C72.9, C74‡, C75.1-C75.3
Neural tube defects	740, 741, 742.0	Q00.0-Q00.2; Q01, Q05
Other neurological disorders	330, 331.8, 331.9, 333, 334, 335.3 336, 337, 341, 349, 349.2, 349.3, 349.8, 353.8, 353.9, 356, 357.0, 357.1, 357.3, 357.4, 357.7, 358, 359, 775.2	F02.2, G10, G11, G12, G12.0, G12.1, G13, G23, G24, G25, G26, G26.0, G36, G37, G61, G70, G71, G73, G90, G95
Parkinson's disease	332, 332.0	F02.3, G20
Rabies	071	A82
Stroke (ischaemic)	433, 434, 435, 437	G45, G46, I63, I65, I66, I67.2-I67.6, I67.5-I67.6, I69.3
Stroke (intracerebral haemorrhage)	431, 432, 437.2	I61, 162, I68.1-I68.2, I69.1-I69.2

Stroke (subarachnoid haemorrhage)	430	I60, I62.0, I67.0-I67.1, I69.0
Tetanus	037-037.9, 771.3, V03.7	A33, A34, A35

*Nervous system cancers refers to the combination of “Brain and central nervous system cancer” and “Neuroblastoma and other peripheral nervous cell tumours”; ‡ Cases and deaths are redistributed by age group between the two GBD causes “neuroblastoma and other peripheral nervous cell tumours” and “other malignant neoplasms.”

Common ICD-10 vital registration codes for the residual “other neurological disorders” category globally in 2021

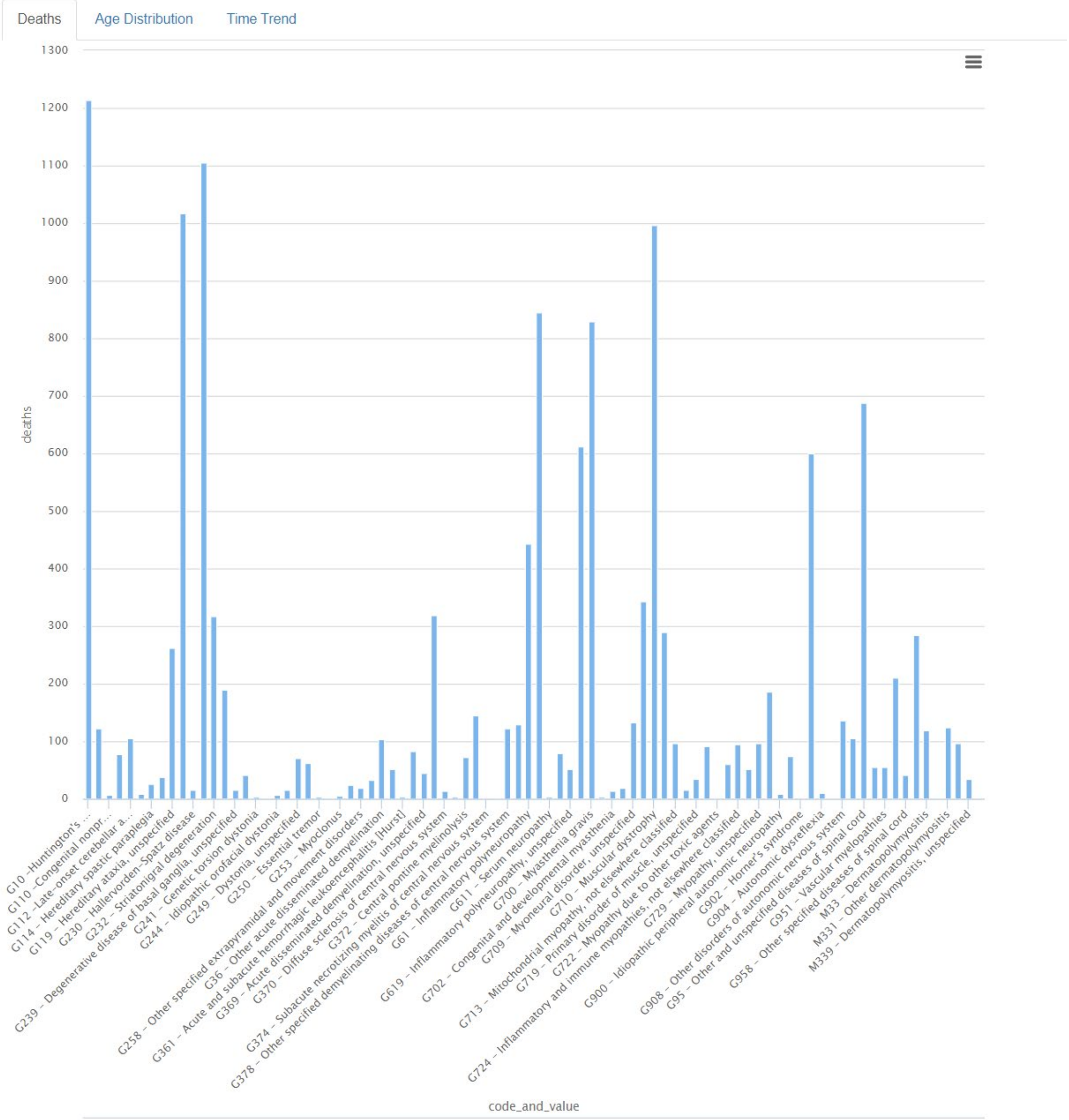

Supplemental methods table 13. Predictive covariates included in fatal models

**Condition**	
Alzheimer's disease and other dementias†	Education (years per capita), smoking prevalence
Encephalitis	Fraction of children born in a given country-year who have received three doses of DTP3, squared percentage of women giving birth in a health facility, lag-distributed income (per capita), sanitation (proportion with access), improved water source (proportion with access), maternal care and immunisation, maternal education (years per capita), Socio-demographic Index, Japanese encephalitis endemic area, Healthcare Access and Quality Index, SEV for child underweight
Idiopathic epilepsy	Cumulative cigarettes (5-year, 10-year), education (years per capita), lag-distributed income (per capita), mean body-mass index, mean cholesterol, mean systolic blood pressure (mmHg), pigs (per capita), SEV for idiopathic epilepsy, Socio-demographic Index, Healthcare Access and Quality Index
Meningitis	Lag-distributed income (per capita), sanitation (proportion with access), improved water source (proportion with access), maternal care and immunisation, maternal education (years per capita), proportion of the population in the meningitis belt, Socio-demographic Index, proportion of population covered by MenAfriVac initiative (meningitis meningococcal type A vaccine), Healthcare Access and Quality Index, SEV for child underweight, proportion of population with PCV3 vaccine (5-year lagged for stockout discontinuities), Hib3 vaccine coverage
Motor neuron disease	Average absolute latitude, diabetes fasting plasma glucose (mmol/L), education (years per capita), lag-distributed income (per capita), mean body-mass index, mean cholesterol, mean temperature, sanitation (proportion with access), improved water source (proportion with access), Socio-demographic Index, Healthcare Access and Quality Index, SEV for diet high in fruit
Multiple sclerosis	Average absolute latitude, cumulative cigarettes (5-year, 10-year), education (years per capita), lag-distributed income (per capita), mean cholesterol, smoking prevalence, Socio-demographic Index, Healthcare Access and Quality Index
Neural tube defects	Abortion legality, ANC1 coverage proportion, ANC4 coverage prop, IFD coverage proportion, maternal educational, Socio-demographic Index, maternal alcohol during pregnancy, Healthcare Access and Quality Index, folic acid µg, fortification folic acid, SEV for age-standardised air hap, SEV for smoking, SEV for diet high in fruit, SEV for diet high in vegetables, SEV for fasting plasma glucose, alcohol litres per capita
Nervous system cancers*	Cumulative cigarettes (10 years), education (years per capita), lag-distributed income (per capita), cholesterol (total, mean per capita), smoking prevalence, Socio-demographic Index, Healthcare Access and Quality Index, summary exposure value (SEV) for diet low in fruit, SEV for diet low in vegetables, SEV for high red meat, litres of alcohol consumed per capita, systolic blood pressure (mmHg), universal health coverage, health worker density, maternal care and immunisation

Other neurological disorders	Cumulative cigarettes (5 years, 10 years), education (years per capita), lag-distributed income (per capita), mean body-mass index, mean cholesterol, mean systolic blood pressure, pigs per capita, population density over 1000 per km^2 (percent), smoking prevalence, Socio-demographic Index, Healthcare Access and Quality Index, SEV for underweight, SEV for low fruit, SEV for diet high in red meat
Parkinson's disease	Average absolute latitude, cumulative cigarettes (10 years), education (years per capita), lag-distributed income (per capita), mean cholesterol, sanitation (proportion with access), improved water source (proportion with access), Socio-demographic Index, Healthcare Access and Quality Index, SEV for diet high in fruit
Rabies	ANC6 coverage proportion, IFD coverage proportion, population density between 500 and 1000 people per km^2 (percent), population density under 150 people per square kilometre (percent), skilled birth attendant coverage proportion, maternal care and immunisation, Socio-demographic Index, Healthcare Access and Quality Index
Stroke (ischaemic)	Diabetes fasting plasma glucose (mmol/L), lag-distributed income, mean body-mass index, mean cholesterol, mean systolic blood pressure, indoor air pollution (household prevalence of cooking with coal or biomass), outdoor air pollution ($PM_{2.5}$, micrograms per cubic meter), proportion over population living above 1500 m elevation, smoking prevalence, SEV for ischaemic stroke, pulses and legumes (grams per day), Healthcare Access and Quality Index, diet high in trans fatty acid, SEV for diet low in fruit, SEV for diet low in vegetables, SEV for diet low in nuts and seeds, SEV for diet low in seafood omega-3 fatty acids, SEV for diet high in polyunsaturated fatty acids, alcohol litres per capita
Stroke (intracerebral haemorrhage)	Diabetes fasting plasma glucose (mmol/L), lag-distributed income, mean body-mass index, mean cholesterol, mean systolic blood pressure, indoor air pollution (household prevalence of cooking with coal or biomass), outdoor air pollution ($PM_{2.5}$, micrograms per cubic meter), proportion over population living above 1500m elevation, smoking prevalence, SEV for intracerebral haemorrhage, pulses and legumes (grams per day), Healthcare Access and Quality Index, diet high in trans fatty acid, SEV for diet low in fruit, SEV for diet low in vegetables, SEV for diet low in nuts and seeds, SEV for diet low in seafood omega-3 fatty acids, SEV for diet high in polyunsaturated fatty acids, alcohol litres per capita
Stroke (subarachnoid haemorrhage)	Lag-distributed income (per capita), mean systolic blood pressure (mmHg), smoking prevalence, Healthcare Access and Quality Index, SEV for subarachnoid stroke, alcohol litres per capita
Tetanus	Education (years per capita), lag-distributed income (per capita), sanitation (proportion with access), Socio-demographic Index, Healthcare Access and Quality Index, fraction of children born in a given country-year who have received three doses of DTP3 (lagged five years for stockout discontinuities)

*Nervous system cancers refers to the combination of "Brain and central nervous system cancer" and "Neuroblastoma and other peripheral nervous cell tumours"; †predictive covariates for dementia come from non-fatal modelling, which informs mortality estimates.

Supplemental results

Supplemental results figure 1. Proportion of total DALYs attributable to different categories of conditions.

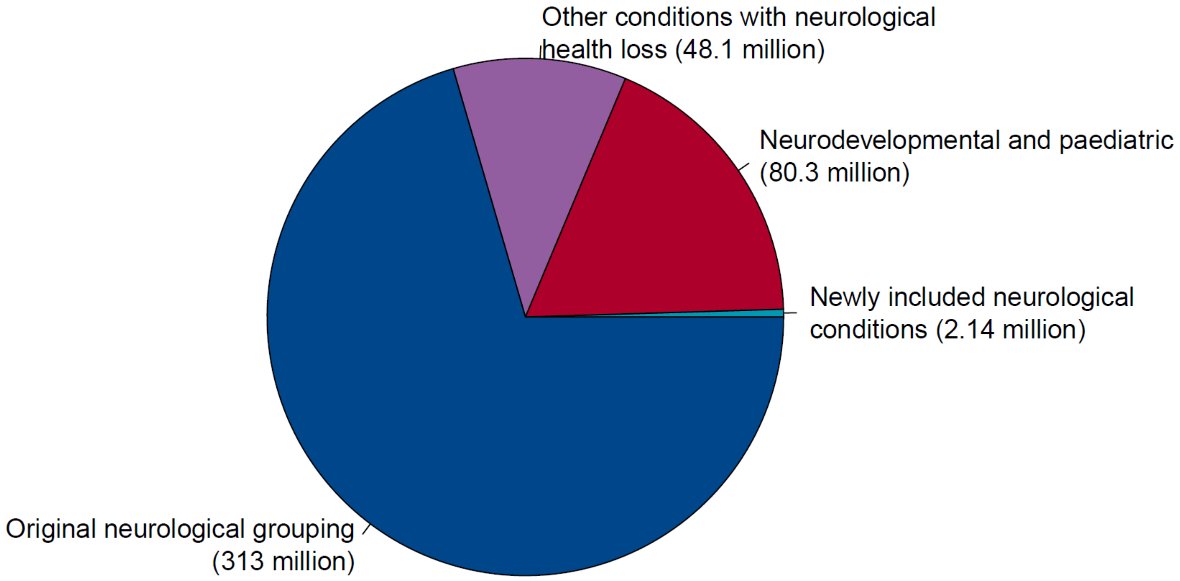

Supplemental results figure 1. Original neurological grouping = 15 categories included in Feigin et al. 2016 analysis, consisting of brain and central nervous system cancer, dementia, encephalitis, epilepsy, meningitis, migraine, motor neuron disease, multiple sclerosis, Parkinson's disease, other neurological disorders, spinal cord injury, stroke, tension-type headache, tetanus, and traumatic brain injury. Neurodevelopmental and paediatric grouping = AD/HD, autism spectrum disorder, fetal alcohol syndrome, idiopathic intellectual disability, neonatal encephalopathy, and neural tube defects. Newly included neurological conditions = Guillain-Barre syndrome, neurocysticercosis, neuroblastoma and other peripheral nervous cell tumours, and rabies. Other conditions with neurological health loss = neurological health loss from cerebral malaria, congenital birth defects, congenital Zika, COVID-19, cystic echinococcosis, diabetic neuropathy, Down syndrome, Klinefelter, neonatal jaundice, neonatal sepsis, neurosyphilis, other chromosomal abnormalities, and preterm birth.

Supplemental results figure 2. Age and sex trends in YLDs in 2021 due to neurological conditions

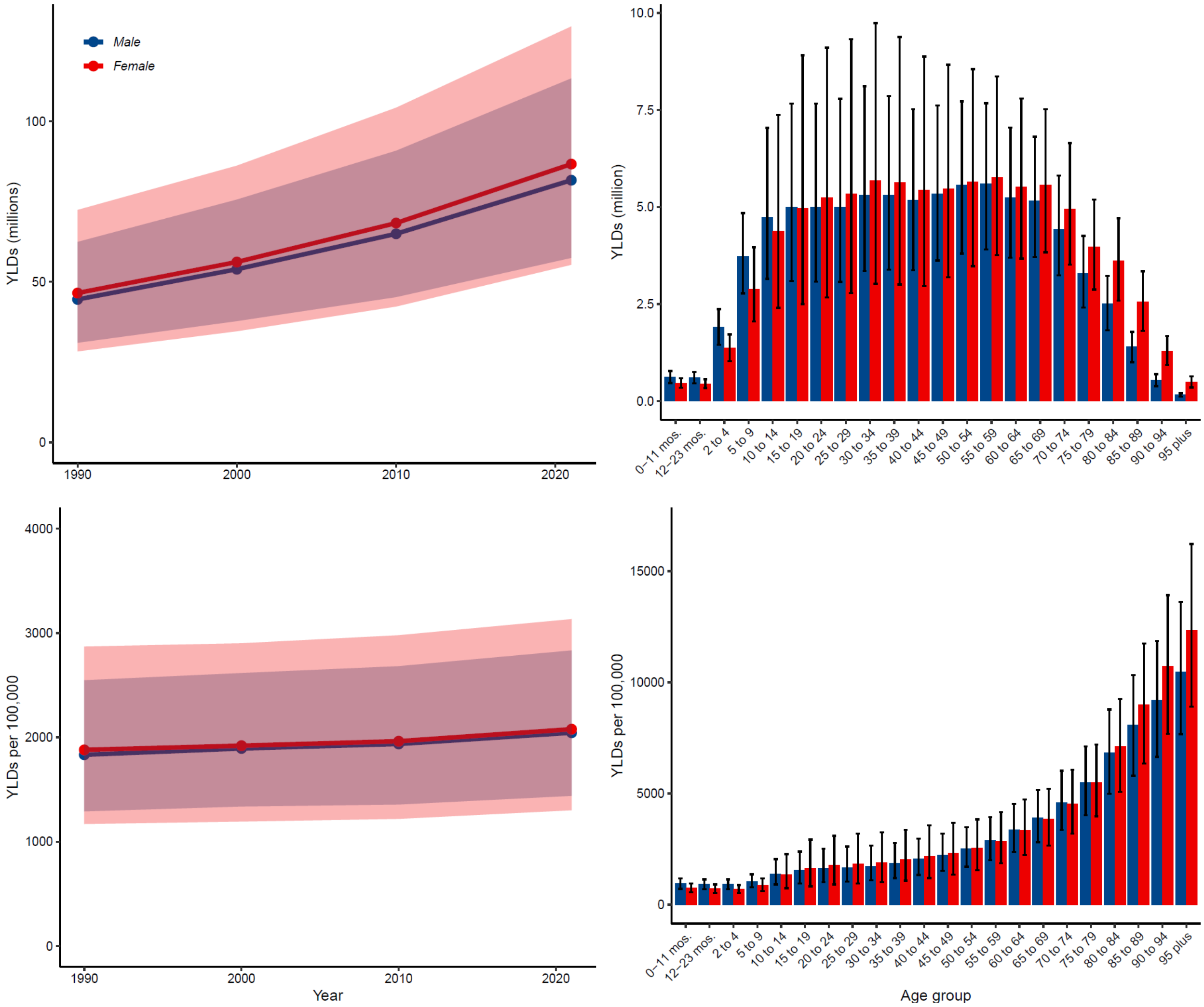

Supplemental results figure 2. Age and sex trends in YLDs in 2021 due to neurological conditions. Left panels show change in counts (top) and age-standardised rates (bottom) over time; right panels show age patterns in counts (top) and rates (bottom). 95% uncertainty intervals are depicted as shading on the left, and black bars on the right.

Supplemental results figure 3. Age and sex trends in YLLs in 2021 due to neurological conditions

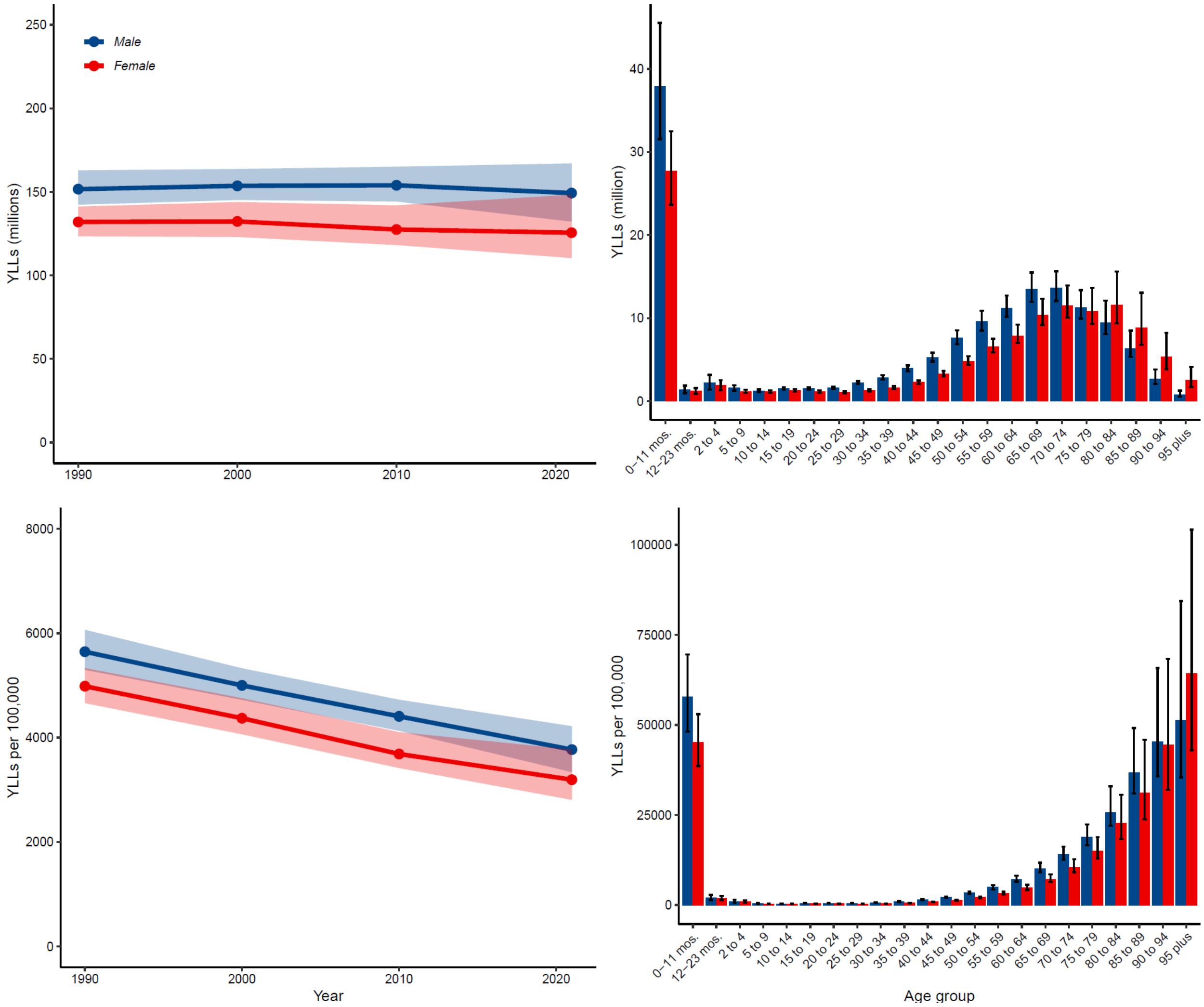

Supplemental results figure 3. Age and sex trends in YLLs in 2021 due to neurological conditions. Left panels show change in counts (top) and age-standardised rates (bottom) over time; right panels show age patterns in counts (top) and rates (bottom). 95% uncertainty intervals are depicted as shading on the left, and black bars on the right.

Supplemental results table 1. Global, regional, and national age-standardised prevalence, years lived with disability, deaths, years of life lost, and disability-adjusted life-years (per 100 000) for all neurological conditions

Location	Prevalence	Years lived with disability	Deaths	Years of life lost	Disability-adjusted life-years
Global	41204·1 (38654·3–43869·9)	2064·1 (1390–2983·1)	139·0 (121·3–173·3)	3573·3 (3190·9–4134·3)	5637·6 (4829·7–6587·9)
Central Europe, eastern Europe, and central Asia	44909·3 (42058·8–48072·2)	2102·9 (1434·5–3033·1)	147·8 (134·0–176·3)	2927·8 (2694·8–3323·8)	5030·9 (4295·3–5899·7)
Central Asia	44400·6 (41269·7–47774·5)	2101·6 (1441·2–3007·1)	162·4 (143·6–190·6)	3536·5 (3170·4–3995·0)	5638·2 (4864·2–6630·4)
Armenia	44550·8 (41412·1–47932·0)	2091·6 (1406·4–2985·1)	99·7 (83·5–131·0)	1869·0 (1622·2–2279·2)	3960·8 (3210·9–4924·0)
Azerbaijan	44150·9 (41038·4–47475·4)	2024·2 (1343·9–2965·8)	144·0 (118·5–177·4)	3425·6 (2882·3–4061·9)	5449·8 (4599·0–6565·2)
Georgia	44663·7 (41410·7–48010·8)	2174·3 (1448·3–3131·6)	195·6 (175·9–223·0)	3668·5 (3286·5–4136·8)	5843·0 (5085·4–6824·9)
Kazakhstan	44726·0 (41528·2–48183·0)	2208·9 (1500·7–3187·7)	196·2 (172·3–226·2)	3576·4 (3116·7–4081·2)	5785·5 (4921·7–6865·1)
Kyrgyzstan	44120·0 (40930·5–47633·3)	1972·4 (1309·1–2872·7)	128·0 (106·1–160·9)	3006·3 (2603·1–3484·8)	4979·0 (4141·0–5911·5)
Mongolia	44073·1 (40783·3–47310·6)	2057·7 (1386·3–2991·4)	174·0 (143·3–210·2)	4037·7 (3400·6–4711·2)	6095·4 (5201·6–7136·4)
Tajikistan	44224·8 (41051·0–47680·0)	2033·4 (1341·9–2972·5)	178·4 (146·0–214·5)	4176·8 (3528·2–4860·7)	6210·2 (5221·1–7267·7)
Turkmenistan	43772·2 (40500·1–47138·6)	1996·1 (1329·0–2881·6)	203·6 (163·4–254·7)	5222·9 (4273·0–6232·7)	7219·2 (6156·0–8578·6)
Uzbekistan	44388·5 (41217·8–47770·1)	2103·4 (1433·7–3030·1)	142·0 (120·0–175·4)	3248·6 (2800·3–3807·1)	5352·2 (4550·4–6445·1)
Central Europe	44859·7 (42020·4–48157·3)	2143·3 (1472·2–3108·3)	126·0 (111·5–151·2)	2245·8 (2025·8–2572·7)	4389·3 (3676·9–5314·6)
Albania	43924·5 (40791·5–47446·8)	2071·5 (1402·6–2993·2)	190·2 (157·3–228·4)	3169·1 (2626·8–3791·1)	5240·5 (4310·4–6250·0)
Bosnia and Herzegovina	45366·2 (42245·2–48907·3)	2450·8 (1700·7–3408·9)	147·0 (119·1–178)	2627·5 (2134·2–3120·9)	5078·2 (4192·1–6039·4)
Bulgaria	44715·4 (41504·2–48267·3)	2276·4 (1552·9–3210·0)	232·0 (204·1–261·4)	4133·6 (3588·4–4733·8)	6410·2 (5546·0–7534·0)
Croatia	44350·4 (41327·1–48034·4)	2154·7 (1457·8–3124·2)	103·3 (87·6–132·8)	1792·7 (1532·3–2164·6)	3947·5 (3200·7–4888·5)
Czechia	44545·2 (41490·0–48142·1)	2190·4 (1474·1–3144·6)	74·9 (60·3–102·2)	1313·1 (1091·3–1685·8)	3503·6 (2766·4–4472·0)
Hungary	44555·6 (41504·8–48087·3)	2172·5 (1466·2–3087·8)	88·3 (72·5–116·1)	1632·6 (1372·6–2026·5)	3805·3 (3018·3–4713·3)
Montenegro	44881·4 (41698·1–48388·4)	2239·1 (1530·2–3234·4)	264·0 (228·9–302·6)	4169·3 (3579·8–4871·5)	6408·5 (5524·0–7474·5)
North Macedonia	45167·2 (42105·4–48696·4)	2384·6 (1626·6–3335·7)	315·5 (267·7–363·8)	4850·1 (4038·7–5675·9)	7234·6 (6188·9–8445·7)
Poland	45589·2 (42683·8–48573·3)	2040·8 (1389·7–2960·3)	94·9 (79·4–123·4)	1722·8 (1505·3–2087·0)	3763·9 (3062·0–4682·1)
Romania	44193·9 (41132·9–47689·7)	2147·2 (1464·6–3127·9)	163·4 (142·6–191·1)	2914·0 (2558·4–3323·0)	5061·3 (4319·5–6048·0)

Serbia	44694·1 (41553·2–48211·1)	2223·8 (1517·6–3146·6)	197·1 (168·4–232·1)	3321·9 (2838·7–3873·0)	5545·7 (4651·2–6568·5)
Slovakia	44354·4 (41397·8–47857·1)	2188·2 (1500·7–3116·8)	99·9 (81·8–128·1)	1828·6 (1514·2–2243·6)	4016·8 (3289·3–4957·5)
Slovenia	43885·8 (40772·8–47405·3)	2027·1 (1378·9–3005·5)	66·8 (52·2–94·1)	1077·5 (875·5–1422·1)	3104·8 (2405·1–3982·7)
Eastern Europe	45126 (42390·3–48210·1)	2066·8 (1395·6–2987·3)	155·4 (138·7–184·4)	2950·8 (2681–3351·9)	5017·8 (4278·7–5913)
Belarus	43405·7 (40126–46923·6)	1906·7 (1290·1–2770·4)	133·6 (110·2–168·7)	2574·8 (2125·5–3129·7)	4481·7 (3728·6–5500)
Estonia	43608·5 (40362·6–47072·5)	1978·1 (1344·6–2838·7)	74·2 (57·8–104·6)	1385·4 (1144·6–1766·7)	3363·7 (2665–4270·8)
Latvia	43754·3 (40495·3–47324·8)	2061 (1394·1–3004·3)	145 (127·4–171·6)	2604·1 (2294·1–2983·2)	4665·2 (3927·2–5642·9)
Lithuania	44350·6 (41031·1–48002·6)	1966·1 (1324·8–2829·5)	106·8 (89·3–138·2)	1991·3 (1731–2411·5)	3957·6 (3236·2–4862·3)
Moldova	43984·3 (40674·7–47459·4)	2023·5 (1375–2908·3)	118·8 (102–147·7)	2604·4 (2279·1–3068·3)	4628·1 (3896·4–5570·7)
Russia	45245·5 (42553·3–48323·8)	2082 (1400·7–3020·2)	166·6 (150·4–194·2)	3105·8 (2829–3493·5)	5188 (4451·5–6119)
Ukraine	45359·9 (42617·7–48362·9)	2066·6 (1409–2932·6)	138·5 (108·1–181·2)	2778·6 (2201·9–3413·6)	4845·3 (3895–5874)
High income	47134·2 (44134·9–50135·3)	1987·8 (1300·4–2953·1)	69·7 (52·8–102·6)	1238·2 (1031·5–1624·3)	3226·4 (2491·6–4185·1)
Australasia	42441·3 (39561–45581·2)	1783·5 (1209·5–2601·2)	61·8 (46·8–91·5)	1098·9 (910·3–1456·4)	2882·6 (2253·6–3717·3)
Australia	42268·6 (39280·3–45483·6)	1772·3 (1199·4–2591·5)	60·6 (45·6–90)	1082·6 (892·9–1436·7)	2855 (2234·7–3693·4)
New Zealand	43307 (40534·1–46225·5)	1841·4 (1224·7–2708)	68·8 (53·1–102)	1189·5 (994·5–1578·6)	3031·2 (2369·9–3887·8)
High-income Asia Pacific	42535·4 (39908·1–45604·3)	1878·6 (1332·1–2673·5)	65·4 (49·1–96)	1105·3 (913·1–1470·4)	2984·6 (2359·4–3768·2)
Brunei	43389·5 (40095·5–47130·3)	2370·6 (1651·1–3250·4)	107·7 (87·7–139·4)	2075·5 (1765·9–2517·3)	4446·6 (3680·3–5298·8)
Japan	42048·3 (39387·9–44958·9)	1808·3 (1286·4–2578·5)	63·8 (47·8–94·3)	1092·1 (900·9–1459·2)	2901 (2286·8–3647·4)
South Korea	43915·6 (40715–47350)	2061·7 (1409·3–2918·3)	79 (60·8–112)	1260·1 (1041·5–1653·8)	3322·4 (2645–4200·1)
Singapore	40516·6 (37304·2–44021·6)	1795·1 (1276·2–2506·3)	38·9 (26·9–62·1)	684·8 (536·9–957·2)	2480·6 (1907·6–3195·1)
High-income North America	49791·6 (46771·4–52802·4)	2165·8 (1436·4–3175·7)	73·7 (55·3–111·2)	1331·2 (1098·5–1778·2)	3497·5 (2697·7–4522·9)
Canada	48071·7 (44639·7–51557·8)	2012·9 (1305–3024·3)	57·6 (43·9–85·4)	1079·6 (909·4–1391·4)	3092·9 (2363·8–4082·4)
Greenland	47587·9 (44350·1–51099·2)	1993·8 (1274·8–2982·6)	112·5 (91·5–148·4)	2176·3 (1860·2–2654·8)	4170·3 (3339·1–5208·9)
USA	49980·6 (47021·3–52986·0)	2183·4 (1448·5–3183·7)	75·8 (56·7–114·7)	1362·9 (1120·3–1829·5)	3546·9 (2737·0–4584·8)
Southern Latin America	40078·8 (37083·5–43287·7)	1867·8 (1255·1–2674·3)	73·4 (59·9–102)	1457·8 (1273·8–1805·9)	3326 (2711·4–4134)
Argentina	39904·2 (36946·4–43170·4)	1827·5 (1236·2–2621)	73 (59·1–102·4)	1489·7 (1297·4–1848·3)	3317·7 (2707·1–4139·5)
Chile	40474·1 (37510·8–43638·4)	1959·9 (1306·7–2800·2)	69·6 (57–95·5)	1315 (1146·7–1640·9)	3275·3 (2602·6–4125·3)

Uruguay	40010·7 (37056·9–43192·3)	1847·6 (1249–2648·8)	91·8 (78–119·9)	1774·4 (1577·5–2140·7)	3622·3 (2986·3–4400·6)
Western Europe	48055·5 (44933–51202·5)	1918·6 (1194·5–2974·6)	68·6 (51·8–101·1)	1194·7 (998·3–1573·3)	3113·5 (2337–4166·5)
Andorra	47164·7 (43847·3–50648·8)	1857·8 (1154·1–2856·6)	59·1 (38·3–95·7)	1024·5 (692·1–1498·6)	2882·5 (2080·5–3946·2)
Austria	46695·6 (43388·6–50094·9)	1943·9 (1244·2–2918)	59·6 (43·3–92·7)	1019 (820·2–1411·6)	2963·3 (2242·4–3956·2)
Belgium	49879·1 (46527·4–53315·2)	2084·6 (1222·1–3267·1)	69·9 (53–103·7)	1298·4 (1083·6–1688·8)	3383·2 (2461·3–4602·3)
Cyprus	47393·6 (44075·5–51001)	1859 (1121–2874·6)	86·1 (66·3–125·4)	1247·9 (1000·5–1725·4)	3107 (2332·8–4182·6)
Denmark	46413·8 (42972·2–50013)	1624·2 (984·7–2529·7)	71·2 (55·3–103·3)	1233·3 (1037·6–1605·3)	2857·7 (2173·1–3746)
Finland	47782·1 (44405·5–51347·7)	1953·4 (1219·9–2998·7)	73·5 (56·8–106·5)	1282·4 (1075·3–1676·9)	3236 (2466·3–4281·5)
France	46142·3 (42801·8–49652·2)	1752·2 (1079·9–2743·6)	59·5 (45·4–88·2)	1090·3 (912·2–1419·7)	2842·6 (2139·8–3816·1)
Germany	48738·4 (45479·6–52080·8)	2049·2 (1261·1–3235·7)	71·7 (52·6–107·3)	1250 (1022·5–1683·9)	3299·4 (2451·4–4467·2)
Greece	48224·6 (44765·4–51691·8)	1986·8 (1247–3040·4)	93·9 (78·9–124·3)	1617·9 (1427–1988·2)	3604·9 (2827·8–4669·4)
Iceland	47421·4 (44029·6–51117·2)	1879·4 (1177·7–2901·4)	64·2 (48–96·2)	1121·1 (912·4–1514·9)	3000·7 (2251·7–4022)
Ireland	47436·3 (44064·9–50961·4)	1835·3 (1137·7–2849·6)	61·8 (46–93·2)	1079·1 (882·3–1445·5)	2914·6 (2173·3–3955·5)
Israel	47431·3 (44162·9–50946·3)	1866·5 (1160·7–2905·2)	57·9 (41·7–89·6)	979·7 (774·9–1377)	2846·4 (2086·4–3907·9)
Italy	48919·6 (46003–51806·3)	1916·3 (1162·6–3014·1)	74·6 (56·2–110·9)	1205·3 (986·1–1629·6)	3121·8 (2305·3–4208·6)
Luxembourg	46537·5 (43246–50102·8)	1794·5 (1116·7–2763·1)	59 (46·8–82·4)	1019·5 (855·5–1321·2)	2814·1 (2120·4–3778·5)
Malta	47420·2 (44169·6–51028·7)	1890·7 (1151·7–2917·5)	62·7 (46·9–93·3)	1159·3 (943·1–1545·8)	3050·1 (2283–4049·3)
Monaco	47082·1 (43709·7–50606·7)	1804 (1099·8–2798·5)	85·5 (65·3–120·9)	1463·5 (1158·8–1916·7)	3267·6 (2490–4251·9)
Netherlands	48845 (44994·9–52482·7)	1799·2 (1113·6–2796·1)	75·7 (58·1–111·3)	1287·7 (1075·5–1717·9)	3087·1 (2321·4–4091·3)
Norway	49331·1 (46431·6–52332·2)	1887·1 (1164·7–2892·1)	64·8 (48·9–97·7)	1140·7 (946·2–1551·8)	3028·1 (2248·8–4087·4)
Portugal	47516·7 (44196·1–51136·4)	1841 (1110·3–2886)	83·6 (68·5–114·6)	1436·5 (1246·9–1803·3)	3277·7 (2536·7–4319·6)
San Marino	47299·2 (43988·8–50827·7)	1810·9 (1105·9–2830·1)	52 (33·4–81·9)	908·9 (606·4–1358·5)	2719·9 (1899·6–3713·1)
Spain	48872·4 (45419·9–52316·2)	2073 (1309·7–3183·7)	63·4 (47·5–94·3)	1100·1 (903·4–1468·6)	3173·4 (2380·5–4283·2)
Sweden	48947·1 (45940·7–51805·7)	1896 (1178·7–2922·7)	61·9 (46·2–93·7)	1048·2 (853·5–1428·6)	2944·5 (2188·4–3981·1)
Switzerland	44913·2 (41629·6–48213·3)	1882·3 (1195·4–2802·5)	57 (41·1–88)	1016·1 (817·3–1378·9)	2898·6 (2178·9–3860·5)
UK	47980·5 (45095·5–50990·4)	1848·2 (1135·3–2840·9)	66·6 (51·9–97·2)	1210·2 (1033·5–1582·8)	3058·6 (2313·1–4069·3)
Latin America and Caribbean	43290·2 (40704·3–46199·7)	2188 (1441·1–3197·1)	83·9 (69–112·4)	1973·7 (1717·9–2358·5)	4162 (3339·9–5145)

Andean Latin America	35634·5 (32927·1–38584·8)	1806·2 (1224·9–2551·6)	69·1 (54·8–90·5)	1734 (1416–2116·7)	3540·3 (2880·7–4338·2)
Bolivia	35682·5 (33021·9–38737·6)	1832·9 (1227–2636·4)	103·5 (78·3–137·2)	2521·6 (1961·1–3236·1)	4354·5 (3552·2–5308·6)
Ecuador	36310 (33769·7–39225·5)	2112·4 (1397·4–2943·5)	70·1 (54–94·3)	1577·5 (1270·6–1962·2)	3690·1 (2908·8–4656·9)
Peru	35334·6 (32343·6–38308·6)	1648·6 (1098·3–2338·9)	61·1 (45·8–83·6)	1592·7 (1256–2020·5)	3241·5 (2615·5–4003·8)
Caribbean	42779·2 (39969·7–45993·8)	2327·5 (1571·2–3295·6)	112·1 (95·1–136·7)	3203·5 (2731·8–3729)	5531·1 (4660·7–6598·2)
Antigua and Barbuda	43116·8 (40272–46386·2)	2445·4 (1610·6–3434·8)	103·6 (92·6–128·3)	2035·9 (1879·1–2366·8)	4481·4 (3627·7–5508·7)
The Bahamas	42863·4 (40019·3–46001·5)	2385 (1565·6–3395·1)	87·6 (69·7–116·2)	1949·9 (1556·4–2448)	4335·1 (3422·5–5479)
Barbados	42833·8 (40031·9–46093·9)	2347·7 (1553·4–3377·2)	106·1 (83·4–134·1)	2217·1 (1727·9–2787·8)	4565 (3648·6–5664·7)
Belize	42547·5 (39752·1–45750)	2322·6 (1551·4–3331·2)	84·6 (70·6–109·2)	1890·5 (1630·2–2204·8)	4213·4 (3419·6–5174·3)
Bermuda	41354 (38453·3–44511·8)	1984 (1270·2–2881·2)	58·1 (44·6–83·7)	1103·6 (881·7–1443·7)	3087·8 (2366·3–4064·8)
Cuba	41042 (38181·5–44266·4)	1887 (1204·1–2795·3)	83·7 (69·6–110·4)	1673·5 (1429·3–2042·9)	3560·7 (2831·8–4543·3)
Dominica	43374·2 (40542·4–46578·3)	2599·8 (1707·2–3647·1)	136·4 (116·5–166)	3391·3 (2768·5–4174·7)	5991 (4984·7–7213)
Dominican Republic	42831·3 (39991·8–46018·1)	2387·6 (1592·7–3387·9)	105·5 (83–138·4)	2643·8 (2136·1–3265·3)	5031·3 (4062·9–6188·5)
Grenada	43440·6 (40585·9–46728·9)	2543·5 (1708·4–3589·6)	122·8 (104·8–149·5)	2600·5 (2277·7–2995·1)	5144·2 (4240·2–6222·1)
Guyana	45340·5 (42491·7–48737·4)	2959·3 (1995·3–4044·7)	171 (136·7–214)	3938·4 (3073·2–4994·4)	6897·8 (5678·2–8394·8)
Haiti	44068·5 (41198·1–47450·5)	2588·7 (1754·7–3670·3)	238·2 (181·9–300·9)	6474·1 (5214·2–7976·6)	9062·3 (7583·5–10882·2)
Jamaica	42531·4 (39769·4–45685)	2308·9 (1519·3–3319·6)	118·5 (92–149·4)	2520·9 (1945·7–3234·1)	4830·1 (3895·8–5963·8)
Puerto Rico	43391·3 (40497·1–46659·8)	2447·4 (1628·2–3448·7)	45·6 (33·1–68·6)	926·5 (729·4–1234·3)	3374 (2530·6–4396)
Saint Kitts and Nevis	42996·4 (40249·6–46179·6)	2443·8 (1610·8–3467·7)	160·5 (136·9–185·8)	3450·8 (2892·3–4052·8)	5894·9 (4981·9–6936·1)
Saint Lucia	44451·7 (41663·9–47753·9)	2759·8 (1889·3–3881·4)	121·7 (99·9–149·4)	2593 (2101·9–3216·1)	5353·1 (4353·5–6499·3)
Saint Vincent and the Grenadines	43710·6 (40991·6–46990·3)	2610·3 (1711·3–3665·2)	120·3 (103·8–145·5)	2519·8 (2162·3–2942·9)	5130·3 (4217·6–6151·2)
Suriname	43834·8 (41038·2–47153·4)	2638·5 (1772·2–3686·6)	130·1 (101–164·2)	3381·6 (2665·2–4121·5)	6020 (4968·7–7336·6)
Trinidad and Tobago	44840·8 (41919·7–48078·9)	2935·2 (1969·1–4129·3)	100·1 (77·4–129·8)	2208·4 (1711·1–2842·2)	5143·8 (4149·5–6480·3)
Virgin Islands	44267·6 (41348·2–47536·6)	2672·4 (1841·6–3827·9)	62·4 (47·7–89)	1292·2 (1017·4–1675·7)	3964·7 (3079·1–5072·7)
Central Latin America	41600·5 (39005·6–44454·7)	2260·8 (1514·9–3222)	68·6 (55–94·2)	1602·7 (1355·3–1956·2)	3864 (3064·5–4804·3)
Colombia	40612·6 (37738·3–43864·1)	2064·9 (1339·3–2993·4)	60·6 (45·1–86·9)	1358 (1070·2–1747·8)	3423·4 (2654·5–4371·2)
Costa Rica	40728·5 (37937·3–43999·8)	2094·5 (1358·5–2998·3)	60·1 (46·2–85·3)	1303·6 (1085·2–1641·5)	3398·6 (2659·5–4280)

El Salvador	40887·6 (38072·3–44132·4)	2133·3 (1384·3–3093·9)	63 (46–90·7)	1389·9 (1072·3–1795·6)	3523·5 (2675·1–4511·1)
Guatemala	41863·5 (39101·5–45011)	2414·5 (1610·7–3376·6)	74·8 (58·8–103·3)	1756·6 (1430·6–2183·6)	4171·5 (3322·8–5155·3)
Honduras	41587·5 (38729·7–44785·3)	2351·5 (1558·1–3323·1)	161·6 (132·7–201·2)	3412·9 (2816·4–4141·4)	5764·6 (4806·8–6967·2)
Mexico	42158·6 (39643·3–44909·2)	2354·7 (1559·8–3315·3)	62·3 (50·5–86·1)	1472·9 (1272·6–1810·3)	3828·1 (3014·8–4763·3)
Nicaragua	41308·7 (38477·9–44520·4)	2255·7 (1535·1–3193·6)	63 (47·9–89·5)	1474·1 (1206·3–1877)	3730·1 (2948·2–4704)
Panama	40542·2 (37743·6–43685·2)	2114·7 (1388–3047·9)	73·9 (56·1–98·1)	1568·4 (1248·8–1953·1)	3683·5 (2881·8–4605·8)
Venezuela	41382 (38483·4–44692·2)	2180·1 (1403·3–3186·7)	95 (68·6–126·8)	2238·5 (1654·5–2887·6)	4419 (3498·1–5460·9)
Tropical Latin America	47458·2 (44643·2–50612·5)	2194·8 (1382·9–3275·5)	96·2 (79–131·8)	2134·7 (1885·5–2585·3)	4329·6 (3462–5497·8)
Brazil	47536·7 (44751·2–50715·7)	2194 (1382·9–3273·7)	95·9 (78·7–131·5)	2130·5 (1884·7–2586·4)	4324·6 (3458·3–5497·6)
Paraguay	45182 (42046·3–48493·1)	2216 (1388·1–3321·2)	109·5 (81·7–145·5)	2325·7 (1778·1–2986·1)	4541·7 (3553·6–5764)
North Africa and Middle East	42526·3 (39912·2–45505·7)	2322·6 (1558·3–3346·4)	144·7 (122·5–180·2)	2946 (2566·4–3483·7)	5268·6 (4388–6356·3)
North Africa and Middle East	42526·3 (39912·2–45505·7)	2322·6 (1558·3–3346·4)	144·7 (122·5–180·2)	2946 (2566·4–3483·7)	5268·6 (4388–6356·3)
Afghanistan	44463 (41658·3–47780)	2884 (1959·8–4060·8)	256·3 (199·5–312·8)	6694·6 (5412·2–7969·2)	9578·1 (7919·5–11313·3)
Algeria	41322·3 (38422·4–44425·8)	2205·8 (1453·2–3147·3)	146·8 (116·7–187·2)	2452·3 (1974·9–3009·9)	4658 (3764–5703·7)
Bahrain	42118·8 (39176·5–45548)	2523·7 (1676·6–3562)	121·2 (97·4–163·5)	1977 (1627·5–2511·3)	4500·9 (3606·8–5649·6)
Egypt	44233·5 (41513·5–47215·2)	2356·2 (1552·4–3393·4)	215 (175·6–262·9)	3936·9 (3206·6–4738·6)	6293·1 (5252·6–7532·1)
Iran	45467·4 (42892·7–48175·6)	2215·3 (1451·6–3249)	101 (84·5–135·4)	1803 (1580·4–2252)	4018·4 (3207·9–5031·9)
Iraq	43529·2 (40716·6–46759·3)	2770·8 (1872·3–3963·6)	214·9 (174·3–257·3)	4256·2 (3420·1–5062·1)	7026·9 (5826·7–8291·9)
Jordan	42171·7 (39355·9–45572·5)	2401·1 (1607·9–3428)	103·1 (80·2–136·7)	1874·7 (1493·5–2384·3)	4275·9 (3372·2–5422·3)
Kuwait	42267·3 (39402–45611·8)	2500·7 (1645·3–3532)	66·3 (47·3–99·1)	1156·8 (880·4–1581·1)	3657·6 (2772·8–4735·1)
Lebanon	42784·3 (39966–46110·9)	2529·4 (1683·1–3595·4)	79·3 (61·2–115·2)	1414·1 (1148·1–1909·2)	3943·6 (3066·1–5014·1)
Libya	41848·1 (39024·5–45171)	2325·8 (1539·2–3339·9)	123·5 (90·8–170·9)	2632·3 (1977·6–3506·2)	4958 (3928·1–6110·3)
Morocco	42631·5 (39763·5–45881·4)	2459·8 (1643·8–3454·1)	165·1 (127·1–206·3)	3109·9 (2433·7–3780·1)	5569·6 (4483·9–6856·7)
Oman	40632·1 (37795–43827·4)	2187·9 (1452–3119·4)	112·1 (88·6–152·2)	2023·2 (1644·8–2559·5)	4211·2 (3320·8–5188·5)
Palestine	42362 (39469·7–45707·4)	2405·6 (1601·4–3450·9)	156 (132·3–197·6)	2775·5 (2380–3312·6)	5181 (4218·7–6268·4)
Qatar	41883·5 (38892·5–45162·6)	2565·6 (1698–3613·6)	93·2 (68·8–134·8)	1465 (1099·6–2004·5)	4030·8 (3114·2–5212·3)
Saudi Arabia	39380 (36700·9–42502·3)	2225·2 (1479·3–3248·9)	140·2 (113·6–182·1)	2613·6 (2124·5–3275·3)	4838·6 (3965·9–5931·2)

Sudan	41070 (38249·9–44230·5)	2048·2 (1326·7–3001·2)	159·8 (123·1–207·8)	3510·9 (2684·5–4492·3)	5558·8 (4446·6–6874·6)
Syria	42373·6 (39616·7–45566·3)	2400·3 (1604–3399·7)	148·4 (114·1–196)	2936·5 (2287–3756·5)	5336·7 (4248·9–6640·5)
Tunisia	41612·3 (38712–44827·3)	2215·3 (1469·6–3238·5)	117·1 (83·7–161·5)	2066·8 (1483·7–2763·7)	4282·2 (3372·9–5449·5)
Türkiye	39770·4 (37278–42750·7)	2166·4 (1433·9–3140·9)	115·6 (91·3–155·3)	2136·5 (1751·9–2688·5)	4303 (3438·9–5335·7)
United Arab Emirates	39970·3 (37071–43171·3)	2233·6 (1474·8–3223)	126·8 (100·9–164·9)	2122·6 (1718·4–2638·9)	4356·3 (3511·2–5381·8)
Yemen	41851·4 (38949·9–44924·2)	2174·8 (1433·5–3126·3)	208·6 (155·1–275·1)	4410·3 (3371·6–5689)	6584·8 (5164·6–8118·9)
South Asia	43445·1 (40894·5–46204·5)	2207 (1469·5–3182·2)	129·3 (113·4–156)	3758 (3360·1–4278·7)	5965·1 (5111·2–6944·1)
South Asia	43445·1 (40894·5–46204·5)	2207 (1469·5–3182·2)	129·3 (113·4–156)	3758 (3360·1–4278·7)	5965·1 (5111·2–6944·1)
Bangladesh	41147·6 (38211·8–44408·2)	2012·8 (1305·2–2957·2)	189·2 (154·6–231·9)	4528·6 (3627·5–5638·8)	6541·3 (5349·4–7962)
Bhutan	40147·6 (37147·5–43437·7)	1836·3 (1167·9–2720·4)	120·2 (94–158·4)	3298·2 (2549·5–4164)	5134·3 (4152·4–6326·7)
India	43746·4 (41241·4–46479·4)	2240·5 (1490·9–3225·7)	113·2 (97·4–141·1)	3042·1 (2666·6–3609·2)	5282·6 (4388·5–6321·5)
Nepal	42184·5 (39308·3–45472)	2154·6 (1391·2–3104·2)	142·5 (117–177·4)	4118·2 (3445·2–4924·1)	6272·5 (5292–7463·8)
Pakistan	43927·7 (41347·3–46644·1)	2216·5 (1472·4–3179·4)	175·3 (146·7–215·6)	6255·4 (5305·1–7273·3)	8471·7 (7193·1–9808·6)
Southeast Asia, east Asia, and Oceania	36566 (34302·6–38893·7)	1832·9 (1207·5–2665·8)	185·1 (159·5–225·9)	3650·9 (3218·3–4214·2)	5483·7 (4591·9–6407)
East Asia	33675·4 (31485·7–35984·2)	1698·6 (1133·9–2446)	179·5 (149·2–223·2)	3256·7 (2753·8–3862·9)	4955·2 (4093·3–5859·4)
China	33683·1 (31485·2–35988·2)	1693·9 (1129·5–2439·2)	181·8 (150·9–225·9)	3275 (2756·7–3875·2)	4968·8 (4099·9–5888·4)
North Korea	33041·8 (30508–35752·8)	1763·1 (1181·3–2569·7)	228·1 (182·2–282·1)	4935·2 (3937·3–6099)	6698 (5517·5–8070·2)
Taiwan (province of China)	33965 (31409·2–36642·1)	1899·1 (1285·1–2765·2)	63·2 (48·1–93·2)	1176·8 (977·8–1555·9)	3076 (2402·1–3930·4)
Oceania	40010·3 (37092·7–42882·9)	2276·1 (1541·3–3264·5)	196·8 (163·3–239·4)	4641·7 (3851·4–5506·5)	6917·7 (5822·1–8166·8)
American Samoa	42024·3 (38961–45206·5)	2801·6 (1867·5–3880·6)	146·6 (121·6–181·6)	3166·2 (2624·9–3858·6)	5967·7 (4950·1–7212·4)
Cook Islands	41621·6 (38665·9–44812·1)	2654·4 (1729·1–3698·2)	91·8 (71·9–127·6)	1753·3 (1388·9–2245·1)	4407·8 (3531–5479·7)
Fiji	41160·1 (38139·3–44291·8)	2623·6 (1776·8–3708·3)	167 (131·9–209·6)	3623·2 (2830·9–4610·1)	6246·8 (5081·9–7584·6)
Guam	38820·3 (35975·8–41794·3)	2084·8 (1364·6–3022·3)	62·9 (49·9–84·8)	1614·2 (1364·5–1937·3)	3699·2 (2938·5–4604·7)
Kiribati	41176·8 (38363·5–44297·2)	2582·4 (1761·5–3566·3)	240 (196·8–297·6)	6151·8 (5081·9–7516·8)	8733·9 (7434·5–10355)
Marshall Islands	42181·2 (39232·9–45363·4)	2815·4 (1946·1–3879·1)	243·1 (191·4–301·3)	5636·6 (4401–7148·6)	8451·6 (6844·7–10185·6)
Federated States of Micronesia	40680·5 (37845·9–43733)	2502·9 (1719·3–3566·9)	236·1 (188·8–297·8)	5387·1 (4280·3–6783·4)	7889·7 (6452·6–9479·2)
Nauru	41209·1 (38422·5–44277·6)	2670 (1814·8–3716·3)	281·8 (227·8–350·5)	6832·7 (5484·3–8585·3)	9502·6 (7832·2–11390·4)

Niue	41275·4 (38268·6–44413·5)	2642·9 (1777·7–3679·6)	178·3 (146·4–218·2)	4325·3 (3593·3–5156·9)	6967·7 (5812·9–8260)
Northern Mariana Islands	39107·6 (36242·2–42003·1)	2169·2 (1436·8–3164·1)	133·3 (112·9–169)	2638·3 (2265·3–3134·7)	4807·5 (4010·3–5799)
Palau	40853 (38017·3–43943·3)	2643·8 (1788·7–3663·6)	175·7 (143·5–222·7)	3998·5 (3291·2–4882·2)	6642·3 (5559·7–8077·3)
Papua New Guinea	39788·9 (36810·4–42640·6)	2204·1 (1482·2–3189·4)	210·1 (165·3–263)	4898·9 (3897·7–5995·7)	7102·8 (5819·1–8573·2)
Samoa	40536 (37711·6–43615·9)	2492·6 (1670·5–3569·2)	189·3 (158·9–235·6)	4072·7 (3423·4–4975·2)	6565·2 (5503·4–7795·6)
Solomon Islands	39610·3 (36776·8–42613·8)	2260·7 (1514·7–3228·2)	265·3 (218·3–333·1)	5849·3 (4777·8–7314)	8109·8 (6820·1–9717·4)
Tokelau	40976·7 (38078·6–44070·3)	2530·1 (1692·2–3612·2)	176·4 (141·9–222·6)	4704·2 (3980–5514·7)	7234 (6089·2–8397)
Tonga	39921·8 (36970·6–43050·5)	2256·2 (1480·6–3256·9)	115·6 (89·1–154)	2331·8 (1820·2–2974·1)	4588·1 (3685·1–5626·8)
Tuvalu	39574·8 (36721·6–42562·1)	2242·5 (1501·3–3220·1)	213·9 (176·2–263·1)	4822·2 (3992·7–5799·5)	7064·5 (5948·8–8493·8)
Vanuatu	39891·5 (37063·6–42904·8)	2309·8 (1536·9–3337·5)	219·5 (183·1–264·9)	5090·3 (4308·4–6044·6)	7400 (6228·4–8692)
Southeast Asia	42198·1 (39641·1–44804)	2102·4 (1348·4–3075·8)	194·8 (171·9–225·4)	4366·2 (3860–4839·5)	6468·7 (5612·9–7534·1)
Cambodia	41549 (38579·5–44616·7)	2076·3 (1325·6–2990·8)	230·9 (185·1–282·5)	4960·6 (3876·2–6147·4)	7036·6 (5666·7–8481)
Indonesia	42280·4 (39740·2–44741·7)	2030·3 (1268·3–3015·6)	260·6 (218–298·6)	5672·2 (4830–6427·5)	7702·4 (6627·1–8898·2)
Laos	41470·1 (38608·2–44533·3)	2106·8 (1353·2–3093·8)	229·6 (181·1–283)	5623·3 (4363·2–7049·2)	7729·7 (6266·3–9467·9)
Malaysia	40443·9 (37424·3–43457·2)	2146·6 (1406·7–3094·1)	131·3 (112·9–167·3)	2608 (2344·8–3091)	4754·7 (3954·6–5832·1)
Maldives	40667 (37725·5–43640·8)	2054·9 (1313·5–3009·4)	111·2 (90·5–139·7)	2187·8 (1809·8–2622·6)	4242·7 (3405·2–5290·8)
Mauritius	43492·4 (40523–46667·5)	2656·6 (1745·1–3861·8)	104 (90·5–134·8)	2346·8 (2108·9–2727·7)	5003·5 (4114·3–6148·7)
Myanmar	42412·1 (39489·8–45491·2)	2301·6 (1510·1–3307·5)	223·7 (184·6–281·1)	5374·5 (4416·4–6587·8)	7675·8 (6357·7–9217·2)
Philippines	42491·3 (39953·6–44984·8)	2048·3 (1298·6–3055)	151·5 (128·1–185·9)	3635·5 (3167·2–4205·4)	5683·8 (4812·4–6733·9)
Seychelles	42834·4 (39753·2–46011·3)	2474·1 (1650·4–3545·4)	117·5 (99·7–151·3)	2465·1 (2136·2–2910·8)	4939·3 (4033·6–6006·4)
Sri Lanka	43934·4 (41087·5–46994·5)	2734·4 (1855·1–3900·6)	143·7 (101·5–188·2)	2513·8 (1769·8–3371·1)	5248·4 (4029·1–6516·6)
Thailand	43285·3 (40285·5–46303·1)	2188·9 (1378·8–3246·4)	99·3 (75·9–131·9)	2141 (1672·9–2690·3)	4329·9 (3399·5–5426·4)
Timor-Leste	41883·7 (39046·9–44973·5)	2248·1 (1450·6–3241·3)	216·4 (168–267·7)	5070·8 (4088·2–6168·4)	7318·6 (6017–8809·6)
Viet Nam	40975·9 (38012·4–44047·2)	1989·1 (1265·5–2967·3)	235·8 (193·4–277·5)	4528·9 (3660·7–5297·1)	6518 (5456·3–7803·5)
Sub-Saharan Africa	37428·6 (34937–40134·4)	1981·5 (1368·5–2774·8)	196·8 (169·3–237·9)	5801·2 (4994·1–6668·5)	7782·9 (6782·9–8916·4)
Central sub-Saharan Africa	38582·4 (35831·6–41757·4)	2126·8 (1459·7–2953)	215·5 (168·3–282·2)	5841 (4824·7–7138·5)	7967·5 (6665·8–9546·6)
Angola	38953·3 (36179·5–42134·3)	2318·2 (1582·7–3243·4)	211·4 (165·7–274·2)	5748 (4574·6–7107·7)	8065·9 (6732·4–9818·7)

Central African Republic	39367·1 (36651·7–42472·8)	2225·6 (1555·4–3113·9)	290 (214·7–373)	9018·8 (7024·5–11246·2)	11243·4 (9115·4–13528·3)
Congo (Brazzaville)	38724·6 (35978·9–41966·1)	2263·2 (1527·9–3129·8)	213 (170·8–276·6)	5188·2 (4207–6342)	7451·1 (6168·9–8786·3)
DR Congo	38390·3 (35584·6–41595·5)	2038·7 (1373·3–2848·5)	213·9 (161·4–288·9)	5751·9 (4675·2–7142·1)	7789·7 (6467·1–9386·7)
Equatorial Guinea	38647·7 (36010·3–41790·4)	2260·1 (1526·2–3149·5)	168·8 (119·8–247·7)	4201·4 (3049·8–5919·5)	6461·4 (5016·7–8220·8)
Gabon	39207·6 (36516·2–42297·6)	2404 (1606·6–3347·6)	183 (140·5–252)	4404·5 (3394·2–5766·7)	6808·6 (5489·3–8288·1)
Eastern sub-Saharan Africa	32204·6 (29956·1–34691·7)	1823 (1289·5–2494·4)	197·9 (168·5–238·5)	5650·4 (4885·2–6475·3)	7473·2 (6585–8482·2)
Burundi	32756·9 (30341·9–35480·3)	1904·7 (1331–2625·2)	213·5 (169·5–266·1)	5932 (4870·6–7039·5)	7835·9 (6598·3–9221·5)
Comoros	33454·2 (31013·4–36368·5)	2000·4 (1429·5–2775·5)	189·5 (151·9–242·7)	5403·4 (4397·5–6486·5)	7403·3 (6223·5–8570·7)
Djibouti	32391·3 (30085·8–35112·4)	1827·8 (1280–2498·9)	188 (142·4–254·1)	4747·3 (3645·2–6208·6)	6575 (5367·9–8094·1)
Eritrea	33288·8 (30778·8–36186·4)	2048 (1446·4–2802·3)	237·1 (182·7–313·3)	6271·1 (4800·2–7969)	8318·6 (6708·1–10120·5)
Ethiopia	29334·5 (27155·7–31729·4)	1738·5 (1219·5–2388)	166 (138·7–206·6)	5292·2 (4559·7–6191·1)	7030·5 (6099·4–8149·2)
Kenya	33809·2 (31586–36249·4)	1850·8 (1268–2555·2)	171 (138·4–214·7)	4361 (3792·2–5122·4)	6211·7 (5313–7223·4)
Madagascar	33292·8 (30918·8–35954)	1893·2 (1305·8–2589·4)	258·4 (198·5–319·5)	6483·3 (5183·5–7900·9)	8375·9 (6957·4–9920·7)
Malawi	32492·9 (29957·1–35397·5)	1724·5 (1193·5–2364·4)	234·4 (194·3–289)	6373·5 (5283·4–7608·1)	8097·7 (6860·5–9602·9)
Mozambique	33471 (31002·4–36374·6)	1942·3 (1362–2703·2)	301·5 (236·9–374·6)	8099·3 (6394·1–9846·9)	10041·3 (8139–12030)
Rwanda	32313·7 (29951·9–35069·4)	1847 (1302·6–2529)	198·3 (152·1–253)	5067·3 (4021·6–6231·1)	6913·8 (5740·3–8311·5)
Somalia	33840·6 (31421·8–36537·4)	1808·7 (1269·7–2518·6)	269 (202·1–344·9)	8568·1 (6537·7–11205·8)	10374·2 (8281·8–12996·1)
South Sudan	32570·7 (30056·2–35419·8)	1692·6 (1197·7–2371·9)	231 (174·5–305·2)	8073·4 (6055·7–10362·5)	9764·8 (7693·4–12235·7)
Uganda	32553·6 (30126·4–35298·5)	1823·3 (1283·1–2516·3)	171·9 (135·8–223·6)	4943·8 (4053·8–6063·6)	6766·8 (5724·1–8076·6)
Tanzania	32568·6 (30028·7–35422·2)	1791·8 (1232·1–2454·2)	171·8 (134·5–223)	4368·2 (3454·2–5451·9)	6159·8 (5058·2–7414·1)
Zambia	35345·1 (32486·7–38202)	2065·5 (1410·2–2873·4)	246·3 (192·5–311·2)	6514·2 (4984·6–8386·7)	8579·1 (6987·9–10471·1)
Southern sub-Saharan Africa	39697·4 (37215·3–42317·6)	2162·3 (1493·2–3013·7)	162 (143·6–193·9)	3909·5 (3494·7–4478·9)	6072 (5258·6–7015·7)
Botswana	38770 (35968·2–41982·9)	2244·4 (1502·8–3090)	154·6 (127·2–198·1)	3803·8 (3110·5–4607·8)	6048 (5046·6–7285·7)
Eswatini	38605·6 (35742·4–41813·1)	2159·1 (1479·3–3057·6)	214·1 (157·2–289·1)	5163·1 (3715·6–6988·5)	7322 (5683·5–9128·4)
Lesotho	38302·9 (35505·4–41530·1)	2018·7 (1402·8–2845·8)	275·3 (217–346·4)	7043·9 (5532·2–8744·1)	9062·5 (7512·8–10965·6)
Namibia	38177·1 (35365·5–41290·5)	2065·3 (1372·1–2906·4)	204·1 (158·1–256·4)	4556·2 (3510·6–5788·4)	6621·4 (5401·3–8180)
South Africa	40110·9 (37663·3–42642·6)	2181·9 (1514·2–3058·1)	149·3 (131·7–181·6)	3525·6 (3171–4018·3)	5707·6 (4906·4–6636·2)

Zimbabwe	38280·6 (35457–41496·9)	2048·4 (1385·6–2867·3)	218·5 (179·1–266·5)	5431·1 (4389·3–6649·4)	7479·1 (6255·1–8892·3)
Western sub-Saharan Africa	40967·8 (38251·6–43720·2)	2026·6 (1342·5–2930·3)	198·4 (169–239·9)	6163·8 (5131–7314·6)	8190·6 (6986–9548·9)
Benin	40832·5 (38019·7–43962·5)	2164·7 (1441·8–3085·2)	217·1 (182·7–261)	6349·9 (5262·4–7731·8)	8514 (7228·6–10143·3)
Burkina Faso	40088·6 (37092·1–43144·1)	1905·4 (1252·8–2790·1)	175·8 (146·1–217·4)	5288·5 (4367·7–6277·6)	7193·4 (6031·2–8507)
Cabo Verde	40952·2 (38118–44063)	2294 (1546·6–3288·4)	144·8 (116·7–179·7)	3228·6 (2556·3–3953·4)	5522·6 (4529·8–6706)
Cameroon	40201·2 (37247·5–43237)	2025·5 (1329·5–2974·3)	211·6 (166·1–279·9)	5887·6 (4606·4–7470·9)	7912·9 (6490–9537·9)
Chad	40240·5 (37315·7–43170·6)	1956·8 (1292–2863·7)	246·3 (195·9–305·6)	7971·5 (6386·4–9874·3)	9927·5 (8255–11898·7)
Côte d'Ivoire	40577·9 (37675·1–43668·7)	2151 (1439·3–3092)	207·9 (165·8–259·5)	6152·2 (4872·5–7549·4)	8302·8 (6833·5–9902·3)
The Gambia	41156·7 (38193·4–44268·8)	2173·3 (1472·3–3099·9)	238·7 (188·7–295·2)	6505·4 (5303·8–7904·3)	8678·6 (7272·8–10370·6)
Ghana	40693·1 (37744·4–43770·3)	2149·5 (1423·2–3066·2)	251·3 (200·6–309·3)	6749·6 (5381·6–8204·4)	8898·6 (7377·5–10648·4)
Guinea	40479·9 (37648·2–43374)	1990·6 (1328·2–2946·1)	230·7 (182·7–290·6)	6873·9 (5311·6–8751·9)	8863·3 (7183–10901·3)
Guinea-Bissau	41046·8 (38200·7–44128·3)	2175·5 (1423·9–3140·9)	301·1 (243·3–361·8)	8459·1 (6877·9–10207·2)	10633·9 (8939·6–12766·4)
Liberia	40884·9 (38045–44122)	2135 (1444·6–3041·9)	210·1 (164·1–270·4)	5995·1 (4550·7–7956·7)	8129·6 (6500·2–10124)
Mali	41449·2 (38491·2–44488·9)	2192·2 (1445·1–3121·2)	196·8 (158·6–242·4)	6345·8 (5205·7–7547·1)	8537·5 (7167·4–10133·8)
Mauritania	40990·2 (38065·5–44174)	2138·1 (1416·1–3035·9)	183·5 (143·6–233·9)	4687·2 (3813·7–5695·1)	6825·3 (5726·8–8117·5)
Niger	40535·3 (37581·6–43739·2)	1930·2 (1254·9–2818·1)	203·7 (158–253·8)	5830·5 (4531·7–7342·6)	7759·4 (6273·3–9359·5)
Nigeria	41350·5 (38752·7–44021·6)	1967·6 (1281–2877·9)	176·7 (149·4–220·3)	5975·6 (4843–7188·3)	7943·1 (6687·3–9358·8)
São Tomé and Príncipe	40830·2 (37965·2–43874·5)	2263·5 (1523·7–3258·6)	173·9 (149·9–208·7)	3936·8 (3294·4–4790·1)	6200·2 (5243·5–7350·7)
Senegal	41264·2 (38395·2–44345·8)	2272·5 (1509–3285)	211·9 (168·9–267·4)	5597·1 (4540·4–6713·5)	7869·4 (6574·3–9297·8)
Sierra Leone	40367·8 (37471·1–43440·1)	1980·3 (1347·3–2873)	224·5 (180·4–283·7)	6871·2 (5352·9–8681·9)	8851·1 (7133·5–11095·5)
Togo	40280·7 (37263·9–43444·6)	2027 (1384·7–2946)	228·7 (178·2–293·7)	6267·4 (4795–8062·8)	8294 (6685·7–10237·3)

Estimates are provided as mean estimates per 100 000 with 95% uncertainty intervals. Locations are organized hierarchically – regions are listed below their respective super region, and countries are listed below their respective region.

Supplemental results figure 4. Age-standardised rate of YLDs and YLLs for all neurological health loss by region in 2021.

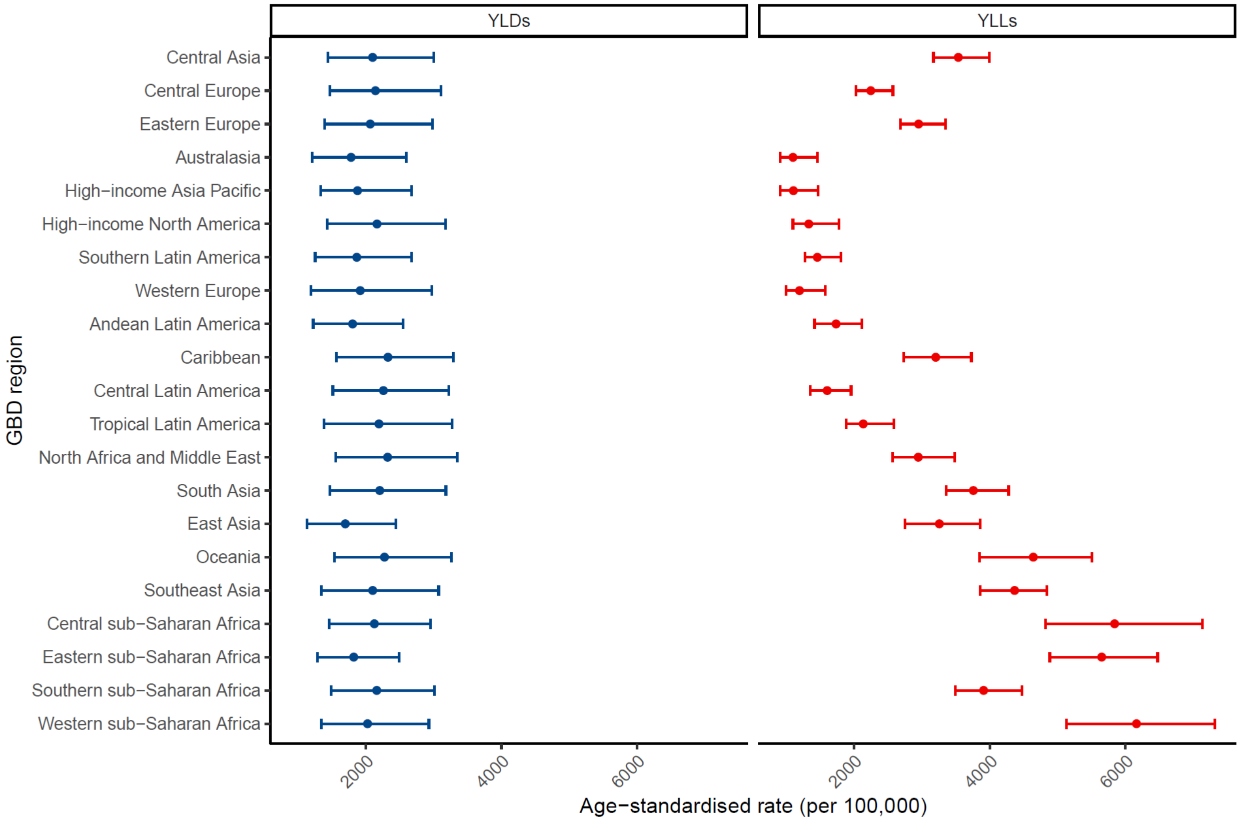

Supplemental results table 2. Global, all-age population attributable fractions for risk and neurological outcome pairs

	Population-attributable fraction (percentage with 95% uncertainty intervals)							
	Stroke	Dementia	Encephalitis	Idiopathic epilepsy	Idiopathic intellectual disability	Meningitis	Multiple sclerosis	Parkinson's disease
Ambient particulate matter pollution	16·7 (11·6-21·0)		0·1 (0·1-0·2)			0·5 (0·3-0·7)		
Household air pollution (solid fuels)	11·3 (6·5-19·5)		0·4 (0·3-0·5)			1·7 (1·4-2·2)		
Lead exposure	7·5 (-1·0-16·7)				63·1 (33·3-81·1)			
Diet low in fibre	2·5 (-0·6-5·2)							
Diet low in fruits	6·0 (0·4-10·6)							
Diet low in vegetables	1·6 (0·4-2·6)							
Diet low in whole grains	1·9 (-2·0-5·3)							
Diet high in red meat	-3·2 (-13·4-4·7)							
Diet high in sodium	10·9 (2·9-23·2)							
High alcohol use	5·3 (1·3-9·9)			7·4 (5·3-9·6)				
High body-mass index	4·8 (0·4-9·9)	7·1 (-1·8-20·0)						
High fasting plasma glucose	10·3 (8·1-12·5)	14·6 (1·2-29·4)						
High LDL cholesterol	13·1 (4·6-21·3)							
High systolic blood pressure	57·3 (42·7-68·4)							
Kidney dysfunction	9·4 (6·9-11·8)							
Low physical activity	2·1 (0·6-3·9)							
Smoking	14·1 (2·6-26·5)	4·3 (1·0-8·0)					11·4 (8·4-14·8)	-9·5 (-15·8--2·8)
Second-hand smoke	4·5 (1·0-8·0)							
Short gestation			0·7 (0·5-0·8)			2·4 (2·0-3·0)		
Low birth weight			1·4 (1·2-1·6)			5·3 (4·5-6·5		

References

1 Sanchez JD, https://www.facebook.com/pahowho. PAHO/WHO | Zika Resources: Case Definitions. Pan American Health Organization / World Health Organization. 2015; published online July 20. https://www3.paho.org/hq/index.php?option=com_content&view=article&id=11117:zika-resources-case-definitions&Itemid=41532&lang=en#gsc.tab=0 (accessed May 11, 2023).

2 Global Burden of Disease Long COVID Collaborators. Estimated Global Proportions of Individuals With Persistent Fatigue, Cognitive, and Respiratory Symptom Clusters Following Symptomatic COVID-19 in 2020 and 2021. *JAMA* 2022; **328**: 1604–15.

3 Battle KE, Lucas TCD, Nguyen M, *et al.* Mapping the global endemicity and clinical burden of Plasmodium vivax, 2000–17: a spatial and temporal modelling study. *The Lancet* 2019; **394**: 332–43.

4 Weiss DJ, Lucas TCD, Nguyen M, *et al.* Mapping the global prevalence, incidence, and mortality of Plasmodium falciparum, 2000–17: a spatial and temporal modelling study. *The Lancet* 2019; **394**: 322–31.

Global Burden of Disease 2021 Nervous System Disorders Collaborators' contributions

Managing the overall research enterprise

Hailey Hagins, Christopher J L Murray, Kanyin Liane Ong, Valery L Feigin, Theo Vos, and Tarun Dua.

Writing the first draft of the manuscript

Jaimie D Steinmetz, Katrin Maria Seeher, Nicoline Schiess, Bochen Cao, Chiara Servili, Vanessa Cavallera, and Max L Mehlman.

Primary responsibility for applying analytical methods to produce estimates

Jaimie D Steinmetz, Emma Nichols, and Madeline Moberg.

Primary responsibility for seeking, cataloguing, extracting, or cleaning data; designing or coding figures and tables

Jaimie D Steinmetz, Katrin Maria Seeher, Emma Nichols, and Ewerton Cousin.

Providing data or critical feedback on data sources

Yohannes Habtegiorgis Abate, Jaffar Abbas, Madineh Akram Abbasi, Mohammadreza Abbasian, Hedayat Abbastabar, Mozhan Abdollahi, Mohammad-Amin Abdollahifar, Rami Abd-Rabu, Auwal Abdullahi, Aidin Abedi, Hassan Abidi, Richard Gyan Aboagye, Hassan Abolhassani, Ahmed Abualhasan, Oladimeji M Adebayo, Victor Adekanmbi, Tayo Alex Adekiya, Qorinah Estiningtyas Sakilah Adnani, Aanuoluwapo Adeyimika Afolabi, Saira Afzal, Bright Opoku Ahinkorah, Sajjad Ahmad, Ali Ahmed, Ayman Ahmed, Haroon Ahmed, Jivan Qasim Ahmed, Muktar Beshir Ahmed, Budi Aji, Hossein Akbarialiabad, Fares

Alahdab, Mohammed Albashtawy, Mohammad T. AlBataineh, Hediyeh Alemi, Abdelazeem M. Algammal, Abid Ali, Liaqat Ali, Mohammed Usman Ali, Rafat Ali, Syed Shujait Shujait Ali, Syed Mohamed Aljunid, Joseph Uy Almazan, Sami Almustanyir, Ahmed Yaseen Alqutaibi, Zaid Altaany, Nelson Alvis-Guzman, Karem H Alzoubi, Alireza Amindarolzarbi, Hubert Amu, Dickson A Amugsi, Deanna Anderlini, Sofia Androudi, Dhanalakshmi Angappan, Hany Ariffin, Johan Ärnlöv, Tahira Ashraf, Seyyed Shamsadin Athari, Bantalem Tilaye Tilaye Atinafu, Prince Atorkey, Maha Moh'd Wahbi Atout, Alok Atreya, Beatriz Paulina Ayala Quintanilla, Zahra Azizi, Ahmed Y Azzam, Ashish D Badiye, Sara Bagherieh, Atif Amin Baig, Senthilkumar Balakrishnan, Ovidiu Constantin Baltatu, Maciej Banach, Soham Bandyopadhyay, Indrajit Banerjee, Mehmet Firat Baran, Miguel A Barboza, Mainak Bardhan, Till Winfried Bärnighausen, Amadou Barrow, Sanjay Basu, Abdul-Monim Mohammad Batiha, Bernhard T Baune, Ahmet Begde, Alehegn Bekele, Melaku Ashagrie Belete, Uzma Iqbal Belgaumi, Derrick A. Bennett, Abhishek Bhadra, Kayleigh Bhangdia, Ajay Nagesh Bhat, Gurjit Kaur Bhatti, Jasvinder Singh Bhatti, Rajbir Bhatti, Mariah Malak Bilalaga, Aadam Olalekan Bodunrin, Archith Boloor, Dejana Braithwaite, Carol Brayne, Danilo Buonsenso, Katrin Burkart, Muhammad Hammad Butt, Nadeem Shafique Butt, Daniela Calina, Ismael R Campos-Nonato, Chao Cao, Andre F Carvalho, Carlos A Castañeda-Orjuela, Ferrán Catalá-López, Pamela R Uscamaita Chacón-Uscamaita, Gashaw Sisay Chanie, Vijay Kumar Chattu, Jianqi Chen, Ritesh Chimoriya, Abdulaal Chitheer, Rajiv Chowdhury, Dinh-Toi Chu, Isaac Sunday Chukwu, Eric Chung, Alyssa Columbus, Josielli Comachio, Paolo Angelo Cortesi, Rosa A S Couto, Michael H Criqui, Natália Cruz-Martins, Sriharsha Dadana, Xiaochen Dai, Zhaoli Dai, Lalit Dandona, Rakhi Dandona, Saswati Das, Alejandro de la Torre-Luque, Hiwot Dejene, Andreas K Demetriades, Hardik Dineshbhai Desai, Samath Dhamminda Dharmaratne, Meghnath Dhimal, Diana Dias da Silva, Daniel Diaz, Mahmoud Dibas, M Ashworth Dirac, Thanh Chi Do, Thao Huynh Phuong Do, Khanh Duy Khanh Doan, Milad Dodangeh, Mohamed Fahmy Doheim, Haneil Larson Dsouza, Senbagam Duraisamy, Oyewole Christopher Durojaiye, Michael Ekholuenetale, Temitope Cyrus Ekundayo, Noha Mousaad Elemam, Frank J Elgar, Islam Y Elgendy, Legesse Tesfaye Elilo, Sharareh Eskandarieh, Abdolmajid Fadaei, Adeniyi Francis Fagbamigbe, Ayesha Fahim, Jawad Fares, Farshad Farzadfar, Ali Fatehizadeh, Syeda Anum Fatima Fatima, Alireza Feizkhah, Seyed-Mohammad Fereshtehnejad, Alize J Ferrari, Takeshi Fukumoto, Muktar A. Gadanya, Yaseen Galali, William M. Gardner, Shuo-Yan Gau, Lemma Getacher, Bardiya Ghaderi Yazdi, Fariborz Ghaffarpasand, Reza Ghanbari, Ramy Mohamed Ghazy, Elena Ghotbi, Sherief Ghozy, Ali Golchin, Mahaveer Golechha, Pouya Goleij, Alessandra C Goulart, Habtamu Alganeh Guadie, Bhawna Gupta, Sapna Gupta, Veer Bala Gupta, Vivek Kumar Gupta, Nils Haep, Teklehaimanot Gereziher Haile, Arvin Haj-Mirzaian, Brian J Hall, Rabih Halwani, Mohammad Hamiduzzaman, Hannah Han, Harapan Harapan, Arief Hargono, Netanja I. Harlianto, Josep Maria Haro, Nicholas Nathaniel Hartman, Faizul Hasan, Soheil Hassanipour, Hadi Hassankhani, Mohammed Bheser Hassen, Johannes Haubold, Nobuyuki Horita, Mehdi Hosseinzadeh, Chengxi Hu, Nawfal R Hussein, Hong-Han Huynh, Segun Emmanuel Ibitoye, Farideh Iravanpour, Sheikh Mohammed Shariful Islam, Gaetano Isola, Chidozie C D Iwu, Mahalaxmi Iyer, Mahboobeh Jafari, Morteza Jafarinia, Haitham Jahrami, Abhishek Jaiswal, Mihajlo Jakovljevic, Roland Dominic G. Jamora, Saad Javeed, Sathish Kumar Jayapal, Shubha Jayaram, Catherine Owens Johnson, Walter D Johnson, Jost B. Jonas, Abel Joseph, Charity Ehimwenma Joshua, Mikk Jürisson, Zubair Kabir, Vidya Kadashetti, Rizwan Kalani, Feroze Kaliyadan, Himal Kandel, Mehrdad Karajizadeh, André Karch, Nicholas J Kassebaum, Harkiran Kaur, Gbenga A. Kayode, Foad Kazemi, Cathleen Keller, Yousef Saleh Khader, Himanshu Khajuria, Mohammad Khammarnia, Maseer Khan, Moien AB Khan, Yusra H Khan, Mahammed Ziauddin Khan suheb, Shaghayegh Khanmohammadi, Khaled Khatab, Sorour Khateri, Mahalaqua Nazli Khatib, Grace Kim, Jihee Kim, Yun Jin Kim, Adnan Kisa, Sezer Kisa, Soewarta Kosen, Kewal Krishan, Vijay

Krishnamoorthy, Barthelemy Kuate Defo, G Anil Kumar, Manasi Kumar, Dian Kusuma, Hmwe Hmwe Kyu, Muhammad Awwal Ladan, Tri Laksono, Dharmesh Kumar Lal, Judit Lám, Savita Lasrado, Kamaluddin Latief, Kaveh Latifinaibin, Kathryn Mei-Ming Lau, Long Khanh Dao Le, Thao Thi Thu Le, Caterina Ledda, Munjae Lee, Sang-woong Lee, Seung Won Lee, Yo Han Lee, Wei Li, Jue Liu, Wei Liu, László Lorenzovici, Stefan Lorkowski, Jailos Lubinda, Zheng Feei Ma, Mohammed Magdy Abd El Razek, Azzam A Maghazachi, Azeem Majeed, Jeadran N. Malagón-Rojas, Kashish Malhotra, Tauqeer Hussain Mallhi, Deborah Carvalho Malta, Aseer Manilal, Mohammad Ali Mansournia, Seyed Farzad Maroufi, Francisco Rogerlândio Martins-Melo, Winfried März, Roy Rillera Marzo, Andrea Maugeri, John J. McGrath, Jitendra Kumar Meena, Walter Mendoza, Oliver Mendoza-Cano, Ritesh G Menezes, Atte Meretoja, Irmina Maria Michalek, Le Huu Nhat Minh, Erkin M Mirrakhimov, Awoke Misganaw, Prasanna Mithra, Biru Abdissa Mizana, Esmaeil Mohammadi, Mustapha Mohammed, Salahuddin Mohammed, Shafiu Mohammed, Syam Mohan, Nagabhishek Moka, Ali H Mokdad, Holger Möller, Lorenzo Monasta, Maryam Moradi, Yousef Moradi, Sumaira Mubarik, Faraz Mughal, George Duke Mukoro, Admir Mulita, Francesk Mulita, Christopher J L Murray, Ahamarshan Jayaraman Nagarajan, Ganesh R Naik, Hastyar Hama Rashid Najmuldeen, Hossein Namdar Areshtanab, Shumaila Nargus, Bruno Ramos Nascimento, Hadis Nasoori, Zuhair S Natto, Biswa Prakash Nayak, Ionut Negoi, Ruxandra Irina Negoi, Charles Richard James Newton, Dang H Nguyen, Hau Thi Hien Nguyen, Hien Quang Nguyen, Phat Tuan Nguyen, Van Thanh Nguyen, Robina Khan Niazi, Taxiarchis Konstantinos Nikolouzakis, Bo Norrving, Jean Jacques Noubiap, Dieta Nurrika, Bogdan Oancea, Ismail A. Odetokun, Osaretin Christabel Okonji, Patrick Godwin Okwute, Andrew T Olagunju, Bolajoko Olubukunola Olusanya, Sokking Ong, Obinna E Onwujekwe, Aislyn U Orji, Uchechukwu Levi Osuagwu, Stanislav S Otstavnov, Amel Ouyahia, Mayowa O Owolabi, Mahesh Padukudru P A, Kevin Pacheco-Barrios, Jagadish Rao Padubidri, Raffaele Palladino, Demosthenes Panagiotakos, Songhomitra Panda-Jonas, Anamika Pandey, Ashok Pandey, Jeyaraj Durai Pandian, Helena Ullyartha Pangaribuan, Shahina Pardhan, Romil R Parikh, Jay Patel, Shankargouda Patil, Shrikant Pawar, David M. Pereira, Jeevan Pereira, Mario F P Peres, Simone Perna, Hoang Tran Pham, Gabriel D. Pinilla-Monsalve, Ramesh Poluru, Ali Mohammad Pourbagher-Shahri, Naeimeh Pourtaheri, Elton Junio Sady Prates, Natalie Pritchett, Nameer Hashim Qasim, Fakher Rahim, Amir Masoud Rahmani, Masoud Rahmati, Ali Rajabpour-Sanati, Shakthi Kumaran Ramasamy, Sheena Ramazanu, Nemanja Rancic, Sowmya J Rao, Ahmed Mustafa Rashid, Salman Rawaf, Christian Razo, Nima Rezaei, Taeho Gregory Rhee, Stephen R Robinson, Mónica Rodrigues, Jefferson Antonio Buendia Rodriguez, Leonardo Roever, Emma L B Rogowski, Michele Romoli, Luca Ronfani, Priyanka Roy, Enrico Rubagotti, Aly M A Saad, Korosh Saber, Basema Saddik, Umar Saeed, Mahdi Safdarian, Rajesh Sagar, Mohammad Ali Sahraian, Marwa Rashad Salem, Sara Samadzadeh, Vijaya Paul Samuel, Abdallah M Samy, Nima Sanadgol, Damian Francesco Santomauro, Milena M. Santric-Milicevic, Aswini Saravanan, Arash Sarveazad, Maheswar Satpathy, Mete Saylan, Mehdi Sayyah, Markus P Schlaich, David C Schwebel, Ashenafi Kibret Sendekie, Mihretu Tagesse Sergindo, Yashendra Sethi, Allen Seylani, Moyad jamal Shahwan, Masood Ali Shaikh, Sunder Sham, Muhammad Aaqib Shamim, Mohammad Anas Shamsi, Mohd Shanawaz, Amin Sharifan, Rajesh Sharma, Maryam Shayan, Jiabin Shen, Suchitra M Shenoy, Pavanchand H Shetty, Aminu Shittu, K M Shivakumar, Sunil Shrestha, Inga Dora Sigfusdottir, Garima Singh, Paramdeep Singh, Sarah Brooke Sirota, Shravan Sivakumar, Yerukneh Solomon, Yonatan Solomon, Michael Spartalis, Benjamin A Stark, Timothy J Steiner, Lars Jacob Stovner, Muhammad Suleman, Rizwan Suliankatchi Abdulkader, Jing Sun, David Sunkersing, Chandan Kumar Swain, Rafael Tabarés-Seisdedos, Shima Tabatabai, Mircea Tampa, Ker-Kan Tan, Yibekal Manaye Tefera, Pugazhenthan Thangaraju, Nikhil Kenny Thomas, Tala Tillawi, Marcos Roberto Tovani-Palone, Phu Van Tran, Samuel Joseph Tromans, Guesh Mebrahtom Tsegay,

Abdul Rohim Tualeka, Srikanth Umakanthan, Bhaskaran Unnikrishnan, Alireza Vakilian, Sahel Valadan Tahbaz, Jef Van den Eynde, Shoban Babu Varthya, Tommi Juhani Vasankari, Balachandar Vellingiri, Narayanaswamy Venketasubramanian, Georgios-Ioannis Verras, Vasily Vlassov, Simona Ruxandra Volovat, Theo Vos, Loc Tri Vu, Yasir Waheed, Shu Wang, Kosala Gayan Weerakoon, Abrha Hailay Weldemariam, Ronny Westerman, Dakshitha Praneeth Wickramasinghe, Lauren B Wilner, Charles D A Wolfe, Sarah Wulf Hanson, Suowen Xu, Ali Yadollahpour, Kazumasa Yamagishi, Yuichiro Yano, Alex Yeshaneh, Siyan Yi, Naohiro Yonemoto, Mustafa Z Younis, Chuanhua Yu, Siddhesh Zadey, Mikhail Sergeevich Zastrozhin, Francis Zeukeng, Yunquan Zhang, Zhi-Jiang Zhang, Abzal Zhumagaliuly, Magdalena Zielińska, Yossef Teshome Zikarg, and Mohammad Zoladl.

Developing methods or computational machinery

Madineh Akram Abbasi, Mozhan Abdollahi, Qorinah Estiningtyas Sakilah Adnani, Saira Afzal, Syed Anees Ahmed, Tareq Mohammed Ali AL-Ahdal, Mohammed Albashtawy, Hediyeh Alemi, Abdelazeem M. Algammal, Hubert Amu, Aleksandr Y Aravkin, Ahmed Y Azzam, Kavita Batra, Rose G Bender, Aadam Olalekan Bodunrin, Muhammad Hammad Butt, Gashaw Sisay Chanie, Hitesh Chopra, Kaleb Coberly, Rosa A S Couto, Xiaochen Dai, Frances E Dean, Hiwot Dejene, Hardik Dineshbhai Desai, M Ashworth Dirac, Thanh Chi Do, Khanh Duy Khanh Doan, Iman El Sayed, Mohammad Amin Elahi Najafi, Mehdi Emamverdi, Ayesha Fahim, Ali Fatehizadeh, Lisa M Force, Quan Gan, William M. Gardner, Haftay Gebremedhin Gebreslassie, Sherief Ghozy, Ali Golchin, Teklehaimanot Gereziher Haile, Mohammad Hamiduzzaman, Nicholas Nathaniel Hartman, Mohammad Hasanian, Simon I Hay, Mohammad Heidari, Mehdi Hosseinzadeh, Md. Nazmul Huda, Hong-Han Huynh, Farideh Iravanpour, Morteza Jafarinia, Somnath Jana, Sathish Kumar Jayapal, Farnaz Kalantar, Mohammad Khammarnia, Sorour Khateri, Mahalaqua Nazli Khatib, Adnan Kisa, Chandrakant Lahariya, Kaveh Latifinaibin, Kathryn Mei-Ming Lau, Thao Thi Thu Le, Sang-woong Lee, Mohammed Magdy Abd El Razek, Le Huu Nhat Minh, Ali H Mokdad, Yousef Moradi, Maziar Moradi-Lakeh, Jonathan F. Mosser, George Duke Mukoro, Admir Mulita, Francesk Mulita, Christopher J L Murray, Hastyar Hama Rashid Najmuldeen, Abdallah Y Naser, Dang H Nguyen, Hien Quang Nguyen, Phat Tuan Nguyen, Van Thanh Nguyen, Emma Nichols, Andrew T Olagunju, Michal Ordak, Aislyn U Orji, Hoang Tran Pham, Nameer Hashim Qasim, Amir Masoud Rahmani, Ali Rajabpour-Sanati, Nemanja Rancic, Ahmed Mustafa Rashid, Christian Razo, Mónica Rodrigues, Emma L B Rogowski, Koushik Roy Pramanik, Enrico Rubagotti, Korosh Saber, Umar Saeed, Sara Samadzadeh, Abdallah M Samy, Damian Francesco Santomauro, Maheswar Satpathy, Mehdi Sayyah, Sarah Brooke Sirota, Jaimie D Steinmetz, Chandan Kumar Swain, Yibekal Manaye Tefera, Tala Tillawi, Theo Vos, Ronny Westerman, Lauren B Wilner, Sarah Wulf Hanson, Alireza Zali, Ghazal G Z Zandieh, Mohammed G M Zeariya, and Peng Zheng.

Providing critical feedback on methods or results

Yohannes Habtegiorgis Abate, Jaffar Abbas, Madineh Akram Abbasi, Mohammadreza Abbasian, Hedayat Abbastabar, Michael Abdelmasseh, Mohammad Abdollahi, Mozhan Abdollahi, Rami Abd-Rabu, Deldar Morad Abdulah, Auwal Abdullahi, Aidin Abedi, Roberto Ariel Abeldaño Zuñiga, Hassan Abidi, Richard Gyan Aboagye, Hassan Abolhassani, Woldu Aberhe Abrha, Ahmed Abualhasan, Eman Abu-Gharbieh, Salahdein Aburuz, Lawan Hassan Adamu, Isaac Yeboah Addo, Oladimeji M Adebayo, Victor Adekanmbi, Tayo Alex Adekiya, Wirawan Adikusuma, Qorinah Estiningtyas Sakilah Adnani, Saryia Adra, Tsion

Afework Afework, Aanuoluwapo Adeyimika Afolabi, Ali Afraz, Saira Afzal, Shahin Aghamiri, Antonella Agodi, Williams Agyemang-Duah, Bright Opoku Ahinkorah, Aqeel Ahmad, Danish Ahmad, Sajjad Ahmad, Amir Mahmoud Ahmadzade, Ali Ahmed, Ayman Ahmed, Haroon Ahmed, Jivan Qasim Ahmed, Luai A. Ahmed, Muktar Beshir Ahmed, Syed Anees Ahmed, Budi Aji, Olufemi Ajumobi, Seyed Esma'il Akade, Hossein Akbarialiabad, Karolina Akinosoglou, Rufus Olusola Akinyemi, Maxwell Akonde, Syed Mahfuz Al Hasan, Fares Alahdab, Tareq Mohammed Ali AL-Ahdal, Rasmieh Mustafa Al-amer, Mohammed Albashtawy, Mohammad T. AlBataineh, Hediyeh Alemi, Abdelazeem M. Algammal, Adel Ali Saeed Al-Gheethi, Robert Kaba Alhassan, Abid Ali, Endale Alemayehu Ali, Liaqat Ali, Mohammed Usman Ali, Musa Mohammed Ali, Rafat Ali, Shahid Ali, Syed Shujait Shujait Ali, Zahid Ali, Sheikh Mohammad Alif, Yousef Alimohamadi, Ahmednur Adem Aliyi, Mohamad Aljofan, Syed Mohamed Aljunid, Suvarna Alladi, Joseph Uy Almazan, Sami Almustanyir, Basem Al-Omari, Jaber S Alqahtani, Ibrahim Alqasmi, Rustam Al-Shahi Salman, Zaid Altaany, Jaffar A. Al-Tawfiq, Khalid A Altirkawi, Nelson Alvis-Guzman, Yaser Mohammed Al-Worafi, Hany Aly, Safwat Aly, Karem H Alzoubi, Reza Amani, Alireza Amindarolzarbi, Sohrab Amiri, Mohammad Hosein Amirzade-Iranaq, Hubert Amu, Dickson A Amugsi, Ganiyu Adeniyi Amusa, Jimoh Amzat, Robert Ancuceanu, Deanna Anderlini, David B Anderson, Catalina Liliana Andrei, Dhanalakshmi Angappan, Teklit W Angesom, Alireza Ansari-Moghaddam, Razique Anwer, Mosab Arafat, Demelash Areda, Hany Ariffin, Hidayat Arifin, Mesay Arkew, Mahwish Arooj, Anton A Artamonov, Kurnia Dwi Artanti, Raphael Taiwo Aruleba, Ali A Asadi-Pooya, Tilahun Ferede Asena, Mohammad Asghari-Jafarabadi, Muhammad Ashraf, Tahira Ashraf, Kendalem Asmare Atalell, Seyyed Shamsadin Athari, Bantalem Tilaye Tilaye Atinafu, Prince Atorkey, Maha Moh'd Wahbi Atout, Alok Atreya, Avinash Aujayeb, Abolfazl Avan, Beatriz Paulina Ayala Quintanilla, Haleh Ayatollahi, Olatunde O Ayinde, Seyed Mohammad Ayyoubzadeh, Sina Azadnajafabad, Zahra Azizi, Ahmed Y Azzam, Mahsa Babaei, Muhammad Badar, Ashish D Badiye, Soroush Baghdadi, Sara Bagherieh, Ruhai Bai, Atif Amin Baig, Senthilkumar Balakrishnan, Shivanthi Balalla, Ovidiu Constantin Baltatu, Maciej Banach, Soham Bandyopadhyay, Indrajit Banerjee, Mehmet Firat Baran, Miguel A Barboza, Martina Barchitta, Mainak Bardhan, Suzanne Lyn Barker-Collo, Till Winfried Bärnighausen, Amadou Barrow, Hamideh Bashiri, Hameed Akande Bashiru, Afisu Basiru, João Diogo Basso, Sanjay Basu, Abdul-Monim Mohammad Batiha, Kavita Batra, Bernhard T Baune, Neeraj Bedi, Ahmet Begde, Tahmina Begum, Babak Behnam, Amir Hossein Behnoush, Yannick Béjot, Alehegn Bekele, Melaku Ashagrie Belete, Uzma Iqbal Belgaumi, Rose G Bender, Bright Benfor, Derrick A. Bennett, Isabela M Bensenor, Betyna N Berice, Paulo J G Bettencourt, Abhishek Bhadra, Devidas S. Bhagat, Nikha Bhardwaj, Pankaj Bhardwaj, Ashish Bhargava, Ajay Nagesh Bhat, Vivek Bhat, Gurjit Kaur Bhatti, Jasvinder Singh Bhatti, Rajbir Bhatti, Ali Bijani, Boris Bikbov, Mariah Malak Bilalaga, Atanu Biswas, Veera R Bitra, Virginia Bodolica, Aadam Olalekan Bodunrin, Archith Boloor, Dejana Braithwaite, Hermann Brenner, Maria L. Bringas Vega, Julie Brown, Danilo Buonsenso, Katrin Burkart, Richard A Burns, Yasser Bustanji, Muhammad Hammad Butt, Nadeem Shafique Butt, Zahid A Butt, Lucas Scotta Cabral, Florentino Luciano Caetano dos Santos, Ismael R Campos-Nonato, Chao Cao, Rosario Cárdenas, Giulia Carreras, Andre F Carvalho, Carlos A Castañeda-Orjuela, Adriano Casulli, Ferrán Catalá-López, Alberico L. Catapano, Arthur Caye, Ester Cerin, Pamela R Uscamaita Chacón-Uscamaita, Gashaw Sisay Chanie, Jaykaran Charan, Vijay Kumar Chattu, Endeshaw Chekol Abebe, Hui Chen, Jianqi Chen, Saravana Babu Chidambaram, Ritesh Chimoriya, Abdulaal Chitheer, Yuen Yu Chong, Hitesh Chopra, Sonali Gajanan Choudhari, Enayet Karim Chowdhury, Rajiv Chowdhury, Hanne Christensen, Dinh-Toi Chu, Isaac Sunday Chukwu, Eric Chung, Alyssa Columbus, Josielli Comachio, Joao Conde, Paolo Angelo Cortesi, Vera Marisa Costa, Ewerton Cousin, Rosa A S Couto, Michael H Criqui, Natália Cruz-Martins, Mohammad Amin Dabbagh Ohadi, Sriharsha Dadana,

Omid Dadras, Xiaochen Dai, Zhaoli Dai, Emanuele D'Amico, Hadi A Danawi, Lalit Dandona, Rakhi Dandona, Amira Hamed Darwish, Saswati Das, Subasish Das, Ana Maria Dascalu, Nihar Ranjan Dash, Mohsen Dashti, Fernando Pio De la Hoz, Alejandro de la Torre-Luque, Diego De Leo, Amin Dehghan, Azizallah Dehghan, Hiwot Dejene, Daniel Demant, Andreas K Demetriades, Solomon Demissie, Xinlei Deng, Hardik Dineshbhai Desai, Vinoth Gnana Chellaiyan Devanbu, Kuldeep Dhama, Samath Dhamminda Dharmaratne, Meghnath Dhimal, Diana Dias da Silva, Daniel Diaz, Mahmoud Dibas, Delaney D Ding, M Ashworth Dirac, Mengistie Diress, Thanh Chi Do, Thao Huynh Phuong Do, Khanh Duy Khanh Doan, Milad Dodangeh, Mohamed Fahmy Doheim, Deepa Dongarwar, Haneil Larson Dsouza, Senbagam Duraisamy, Oyewole Christopher Durojaiye, Sulagna Dutta, Arkadiusz Marian Dziedzic, Hisham Atan Edinur, Michael Ekholuenetale, Temitope Cyrus Ekundayo, Iman El Sayed, Mohammad Amin Elahi Najafi, Noha Mousaad Elemam, Frank J Elgar, Islam Y Elgendy, Hala Rashad Elhabashy, Muhammed Elhadi, Legesse Tesfaye Elilo, Richard G Ellenbogen, Omar Abdelsadek Abdou Elmeligy, Mohamed A Elmonem, Mohammed Elshaer, Ibrahim Elsohaby, Mehdi Emamverdi, Theophilus I Emeto, Sharareh Eskandarieh, Adeniyi Francis Fagbamigbe, Ayesha Fahim, Jawad Fares, Mohsen Farjoud Kouhanjani, Andre Faro, Farshad Farzadfar, Ali Fatehizadeh, Mobina Fathi, Saeid Fathi, Syeda Anum Fatima Fatima, Valery L Feigin, Alireza Feizkhah, Seyed-Mohammad Fereshtehnejad, Alize J Ferrari, Nuno Ferreira, Getahun Fetensa, Neda Firouraghi, Florian Fischer, Ana Catarina Fonseca, Lisa M Force, Takeshi Fukumoto, Muktar A. Gadanya, Abhay Motiramji Gaidhane, Yaseen Galali, Nasrin Galehdar, Quan Gan, Aravind P Gandhi, Balasankar Ganesan, William M. Gardner, Naval Garg, Shuo-Yan Gau, Rupesh K. Gautam, Teshome Gebre, Mesfin Gebrehiwot, Haftay Gebremedhin Gebreslassie, Lemma Getacher, Bardiya Ghaderi Yazdi, Fariborz Ghaffarpasand, Reza Ghanbari, Sailaja Ghimire, Ali Gholami, Elena Ghotbi, Sherief Ghozy, Paramjit Singh Gill, Logan M. Glasstetter, Elena V. Gnedovskaya, Ali Golchin, Mahaveer Golechha, Davide Golinelli, Mansueto Gomes-Neto, Alessandra C Goulart, Anmol Goyal, Richard J Gray, Michal Grivna, Habtamu Alganeh Guadie, Bin Guan, Stefano Guicciardi, Damitha Asanga Gunawardane, Hanbing Guo, Bhawna Gupta, Rajeev Gupta, Sapna Gupta, Veer Bala Gupta, Vivek Kumar Gupta, Reyna Alma Gutiérrez, Farrokh Habibzadeh, Vladimir Hachinski, Rasool Haddadi, Mostafa Hadei, Nils Haep, Teklehaimanot Gereziher Haile, Brian J Hall, Rabih Halwani, Mohammad Hamiduzzaman, Hannah Han, Junwei Hao, Harapan Harapan, Habtamu Endashaw Hareru, Netanja I. Harlianto, Josep Maria Haro, Nicholas Nathaniel Hartman, Ahmed I. Hasaballah, Faizul Hasan, Hamidreza Hasani, Shoaib Hassan, Soheil Hassanipour, Hadi Hassankhani, Mohammed Bheser Hassen, Johannes Haubold, Simon I Hay, Khezar Hayat, Mohamed I Hegazy, Golnaz Heidari, Mohammad Heidari, Reza Heidari-Soureshjani, Hamed Hesami, Kamal Hezam, Yuta Hiraike, Howard J. Hoffman, Ramesh Holla, Kathleen Pillsbury Hopf, Nobuyuki Horita, Md Mahbub Hossain, Md. Belal Hossain, Sahadat Hossain, Hassan Hosseinzadeh, Mehdi Hosseinzadeh, Chengxi Hu, Md. Nazmul Huda, Javid Hussain, Nawfal R Hussein, Hong-Han Huynh, Bing-Fang Hwang, Segun Emmanuel Ibitoye, Mehran Ilaghi, Olayinka Stephen Ilesanmi, Irena M. Ilic, Milena D. Ilic, Mustapha Immurana, Farideh Iravanpour, Sheikh Mohammed Shariful Islam, Faisal Ismail, Gaetano Isola, Masao Iwagami, Chidozie C D Iwu, Mahalaxmi Iyer, Ali Jaan, Louis Jacob, Farhad Jadidi-Niaragh, Mahboobeh Jafari, Morteza Jafarinia, Kasra Jahankhani, Nader Jahanmehr, Haitham Jahrami, Abhishek Jaiswal, Mihajlo Jakovljevic, Roland Dominic G. Jamora, Somnath Jana, Nilofer Javadi, Saad Javeed, Sathish Kumar Jayapal, Shubha Jayaram, Heng Jiang, Catherine Owens Johnson, Mohammad Jokar, Jost B. Jonas, Abel Joseph, Nitin Joseph, Charity Ehimwenma Joshua, Mikk Jürisson, Ali Kabir, Zubair Kabir, Gebisa Guyasa Kabito, Vidya Kadashetti, Fatemeh Kafi, Rizwan Kalani, Farnaz Kalantar, Feroze Kaliyadan, Ashwin Kamath, Sagarika Kamath, Tanuj Kanchan, Amit Kandel, Himal Kandel, Kehinde Kazeem Kanmodi, Mehrdad Karajizadeh, Jafar Karami, Ibraheem M Karaye, André Karch, Hanie Karimi, Arman

Karimi Behnagh, Nicholas J Kassebaum, Joonas H Kauppila, Harkiran Kaur, Navjot Kaur, Gbenga A. Kayode, Foad Kazemi, Cathleen Keller, Mohammad Keykhaei, Mohammad Amin Khadembashiri, Yousef Saleh Khader, Morteza Abdullatif Khafaie, Himanshu Khajuria, Amirmohammad Khalaji, Faham Khamesipour, Mohammad Khammarnia, Maseer Khan, Moien AB Khan, Yusra H Khan, Mahammed Ziauddin Khan suheb, Shaghayegh Khanmohammadi, Tripti Khanna, Khaled Khatab, Haitham Khatatbeh, Moawiah Mohammad Khatatbeh, Sorour Khateri, Mahalaqua Nazli Khatib, Mohammad Saeid Khonji, Fatemeh khorashadizadeh, Moein Khormali, Jagdish Khubchandani, Saeid Kian, Grace Kim, Jihee Kim, Min Seo Kim, Yun Jin Kim, Ruth W Kimokoti, Adnan Kisa, Sezer Kisa, Mika Kivimäki, Sonali Kochhar, Ali-Asghar Kolahi, Kamrun Nahar Koly, Farzad Kompani, Walter J Koroshetz, Masoumeh Kourosh Arami, Ai Koyanagi, Michael A Kravchenko, Kewal Krishan, Vijay Krishnamoorthy, Barthelemy Kuate Defo, Md Abdul Kuddus, Ashish Kumar, G Anil Kumar, Manasi Kumar, Nithin Kumar, Netsanet Bogale Kumsa, Satyajit Kundu, Maria Dyah Kurniasari, Dian Kusuma, Ambily Kuttikkattu, Hmwe Hmwe Kyu, Carlo La Vecchia, Muhammad Awwal Ladan, Chandrakant Lahariya, Tri Laksono, Dharmesh Kumar Lal, Tea Lallukka, Judit Lám, Savita Lasrado, Kamaluddin Latief, Kaveh Latifinaibin, Basira Kankia Lawal, Long Khanh Dao Le, Thao Thi Thu Le, Caterina Ledda, Munjae Lee, Sang-woong Lee, Seung Won Lee, Yo Han Lee, Matilde Leonardi, Temesgen L. Lerango, Ming-Chieh Li, Wei Li, Virendra S Ligade, Stephen S Lim, Chaojie Liu, Jue Liu, Wei Liu, Chun-Han Lo, Stany W Lobo, Giancarlo Logroscino, Graciliana Lopes, László Lorenzovici, Stefan Lorkowski, Joana A Loureiro, Jailos Lubinda, Giancarlo Lucchetti, Ricardo Lutzky Saute, Zheng Feei Ma, Mahmoud Mabrok, Monika Machoy, Farzan Madadizadeh, Azzam A Maghazachi, Nastaran Maghbouli, Soleiman Mahjoub, Morteza Mahmoudi, Azeem Majeed, Jeadran N. Malagón-Rojas, Elaheh Malakan Rad, Kashish Malhotra, Ahmad Azam Malik, Iram Malik, Tauqeer Hussain Mallhi, Deborah Carvalho Malta, Aseer Manilal, Vahid Mansouri, Mohammad Ali Mansournia, Bishnu P. Marasini, Hamid Reza Marateb, Seyed Farzad Maroufi, Jose Martinez-Raga, Santi Martini, Francisco Rogerlândio Martins-Melo, Miquel Martorell, Winfried März, Roy Rillera Marzo, João Massano, Yasith Mathangasinghe, Richard James Maude, Andrea Maugeri, Pallab K Maulik, Mahsa Mayeli, Colm McAlinden, John J. McGrath, Jitendra Kumar Meena, Max Alberto Mendez Mendez-Lopez, Walter Mendoza, Ritesh G Menezes, Mohsen Merati, Atte Meretoja, Alexander Merkin, Abera M Mersha, Tomislav Mestrovic, Tomasz Miazgowski, Irmina Maria Michalek, Ephrem Tesfaye Mihretie, Le Huu Nhat Minh, Reza Mirfakhraie, Andreea Mirica, Erkin M Mirrakhimov, Mehdi Mirzaei, Awoke Misganaw, Sanjeev Misra, Prasanna Mithra, Biru Abdissa Mizana, Madeline E Moberg, Ashraf Mohamadkhani, Nouh Saad Mohamed, Esmaeil Mohammadi, Hiwa Mohammadi, Shadieh Mohammadi, Soheil Mohammadi, Marita Mohammadshahi, Mustapha Mohammed, Salahuddin Mohammed, Shafiu Mohammed, Syam Mohan, Hoda Mojiri-forushani, Nagabhishek Moka, Ali H Mokdad, Sabrina Molinaro, Holger Möller, Lorenzo Monasta, Md Moniruzzaman, Fateme Montazeri, Maryam Moradi, Yousef Moradi, Maziar Moradi-Lakeh, Negar Morovatdar, Abbas Mosapour, Jonathan F. Mosser, Elias Mossialos, Majid Motaghinejad, Seyed Ehsan Mousavi, Sumaira Mubarik, Lorenzo Muccioli, Faraz Mughal, George Duke Mukoro, Admir Mulita, Francesk Mulita, Christopher J L Murray, Fungai Musaigwa, Ahmad Mustafa, Ghulam Mustafa, Sathish Muthu, Ahamarshan Jayaraman Nagarajan, Pirouz Naghavi, Ganesh R Naik, Firzan Nainu, Tapas Sadasivan Nair, Hastyar Hama Rashid Najmuldeen, Noureddin Nakhostin Ansari, Gopal Nambi, Hossein Namdar Areshtanab, Shumaila Nargus, Bruno Ramos Nascimento, Abdallah Y Naser, Abdulqadir J J Nashwan, Hadis Nasoori, Zuhair S Natto, Javaid Nauman, Biswa Prakash Nayak, Athare Nazri-Panjaki, Mohammad Negaresh, Hadush Negash, Ionut Negoi, Ruxandra Irina Negoi, Seyed Aria Nejadghaderi, Charles Richard James Newton, Dang H Nguyen, Hau Thi Hien Nguyen, Hien Quang Nguyen, Ngoc-Trinh Thi Nguyen, Phat Tuan Nguyen, Van Thanh Nguyen, Robina Khan Niazi, Emma

Nichols, Taxiarchis Konstantinos Nikolouzakis, Vikram Niranjan, Lawrence Achilles Nnyanzi, Efaq Ali Noman, Nafise Noroozi, Bo Norrving, Jean Jacques Noubiap, Chisom Adaobi Nri-Ezedi, Dieta Nurrika, Bogdan Oancea, Ismail A. Odetokun, Martin James O'Donnell, Ropo Ebenezer Ogunsakin, James Odhiambo Oguta, Hassan Okati-Aliabad, Akinkunmi Paul Okekunle, Osaretin Christabel Okonji, Patrick Godwin Okwute, Andrew T Olagunju, Muideen Tunbosun Olaiya, Matifan Dereje Olana, Matthew Idowu Olatubi, Isaac Iyinoluwa Olufadewa, Bolajoko Olubukunola Olusanya, Ahmed Omar Bali, Kanyin Liane Ong, Obinna E Onwujekwe, Michal Ordak, Aislyn U Orji, Doris V. Ortega-Altamirano, Uchechukwu Levi Osuagwu, Nikita Otstavnov, Stanislav S Otstavnov, Amel Ouyahia, Mayowa O Owolabi, Mahesh Padukudru P A, Kevin Pacheco-Barrios, Jagadish Rao Padubidri, Pramod Kumar Pal, Padmavali Nanaji Palange, Claudia Palladino, Raul Felipe Palma-Alvarez, Feng Pan, Demosthenes Panagiotakos, Songhomitra Panda-Jonas, Anamika Pandey, Ashok Pandey, Jeyaraj Durai Pandian, Ioannis Pantazopoulos, Shahina Pardhan, Pragyan Paramita Parija, Romil R Parikh, Seoyeon Park, Ashwaghosha Parthasarathi, Ava Pashaei, Jay Patel, Shankargouda Patil, Dimitrios Patoulias, Shrikant Pawar, Paolo Pedersini, Umberto Pensato, Jeevan Pereira, Maria Odete Pereira, Ionela-Roxana Petcu, Fanny Emily Petermann-Rocha, Hoang Tran Pham, Michael R Phillips, Gabriel D. Pinilla-Monsalve, Michael A Piradov, Evgenii Plotnikov, Ramesh Poluru, Constance Dimity Pond, Govinda Raj Poudel, Alireza Pouramini, Ali Mohammad Pourbagher-Shahri, Naeimeh Pourtaheri, Peralam Yegneswaran Prakash, Sanjay Prakash, V Prakash, Elton Junio Sady Prates, Natalie Pritchett, Hery Purnobasuki, Nameer Hashim Qasim, Ibrahim Qattea, Gangzhen Qian, Venkatraman Radhakrishnan, Pourya Raee, Hadi Raeisi Shahraki, Ibrar Rafique, Alberto Raggi, Pankaja Raghav Raghav, Meghdad M Rahati, Fakher Rahim, Zahra Rahimi, Mahban Rahimifard, Md Obaidur Rahman, Mosiur Rahman, Muhammad Aziz Rahman, Amir Masoud Rahmani, Shayan Rahmani, Masoud Rahmati, Ali Rajabpour-Sanati, Shakthi Kumaran Ramasamy, Sheena Ramazanu, Nemanja Rancic, Indu Ramachandra Rao, Sowmya J Rao, Deepthi Rapaka, Vahid Rashedi, Ahmed Mustafa Rashid, Mohammad-Mahdi Rashidi, Mehran Rashidi Alavijeh, Salman Rawaf, Christian Razo, Elrashdy Moustafa Mohamed Redwan, Nazila Rezaei, Negar Rezaei, Nima Rezaei, Mohsen Rezaeian, Taeho Gregory Rhee, Abanoub Riad, Stephen R Robinson, Mónica Rodrigues, Jefferson Antonio Buendia Rodriguez, Leonardo Roever, Emma L B Rogowski, Priyanka Roy, Koushik Roy Pramanik, Enrico Rubagotti, Milagros A Ruiz, Katharina S Sunnerhagen, Aly M A Saad, Zahra Saadatian, Korosh Saber, Morteza SaberiKamarposhti, Simona Sacco, Basema Saddik, Erfan Sadeghi, Saeid Sadeghian, Umar Saeed, Usman Saeed, Sher Zaman Safi, Rajesh Sagar, Fatemeh Saheb Sharif-Askari, Narjes Saheb Sharif-Askari, Soumya Swaroop Sahoo, Mohammad Ali Sahraian, Seyed Aidin Sajedi, Joseph W Sakshaug, Mohamed A. Saleh, Marwa Rashad Salem, Hossein Samadi Kafil, Sara Samadzadeh, Yoseph Leonardo Samodra, Vijaya Paul Samuel, Abdallah M Samy, Nima Sanadgol, Rama Krishna Sanjeev, Francesco Sanmarchi, Damian Francesco Santomauro, Ichtiarini Nurullita Santri, Milena M. Santric-Milicevic, Arash Sarveazad, Maheswar Satpathy, Mete Saylan, Mehdi Sayyah, Nikolaos Scarmeas, Nicoline Schiess, Markus P Schlaich, Art Schuermans, Michaël Schwarzinger, David C Schwebel, Katrin Maria Seeher, Ashenafi Kibret Sendekie, Pallav Sengupta, Subramanian Senthilkumaran, Dragos Serban, Mihretu Tagesse Sergindo, Yashendra Sethi, SeyedAhmad SeyedAlinaghi, Mahan Shafie, Saeed Shahabi, Ataollah Shahbandi, Samiah Shahid, Fariba Shahraki-Sanavi, Hamid R Shahsavari, Moyad Jamal Shahwan, Masood Ali Shaikh, Adisu Tafari T Shama, Muhammad Aaqib Shamim, Mehran Shams-Beyranvand, Mohammad Anas Shamsi, Mohd Shanawaz, Medha Sharath, Sadaf Sharfaei, Amin Sharifan, Rajesh Sharma, Bereket Beyene Shashamo, Maryam Shayan, Rahim Ali Sheikhi, Shashank Shekhar, Jiabin Shen, Suchitra M Shenoy, Pavanchand H Shetty, Desalegn Shiferaw Shiferaw, Rahman Shiri, Aminu Shittu, K M Shivakumar, Fereshteh Shokri, Sina Shool, Seyed Afshin Shorofi, Sunil Shrestha, Akhenaten Benjamin

Siankam Tankwanchi, Emmanuel Edwar Siddig, Inga Dora Sigfusdottir, João Pedro Silva, Luís Manuel Lopes Rodrigues Silva, Ehsan Sinaei, Balbir Bagicha Singh, Garima Singh, Paramdeep Singh, Sarah Brooke Sirota, Shravan Sivakumar, Abdullah Al Mamun Sohag, Hamidreza Soleimani, Solikhah Solikhah, Yerukneh Solomon, Yonatan Solomon, Suhang Song, Yimeng Song, Houman Sotoudeh, Michael Spartalis, Benjamin A Stark, Joseph R Starnes, Antonina V Starodubova, Timothy J Steiner, Muhammad Suleman, Rizwan Suliankatchi Abdulkader, Abida Sultana, Jing Sun, Angel Sunny, Hani Susianti, Chandan Kumar Swain, Mindy D Szeto, Rafael Tabarés-Seisdedos, Seyyed Mohammad Tabatabaei, Shima Tabatabai, Mohammad Tabish, Majid Taheri, Mircea Tampa, Jacques JL Lukenze Tamuzi, Ker-Kan Tan, Haosu Tang, Minale Tareke, Ingan Ukur Tarigan, Razieh Tavakoli Oliaee, Seyed Mohammad Tavangar, Yibekal Manaye Tefera, Worku Animaw Temesgen, Mohamad-Hani Temsah, Masayuki Teramoto, Amensisa Hailu Tesfaye, Edosa Geta Tesfaye, Ocean Thakali, Pugazhenthan Thangaraju, Rajshree Thapa, Rekha Thapar, Nikhil Kenny Thomas, Amanda G Thrift, Jansje Henny Vera Ticoalu, Tala Tillawi, Razie Toghroli, Marcello Tonelli, Marcos Roberto Tovani-Palone, Eugenio Traini, Nghia Minh Tran, Ngoc-Ha Tran, Phu Van Tran, Samuel Joseph Tromans, Thien Tan Tri Tai Truyen, Guesh Mebrahtom Tsegay, Evangelia Eirini Tsermpini, Derara Girma Tufa, Aniefiok John Udoakang, Inam Ulhaq, Muhammad Umair, Srikanth Umakanthan, Krishna Kishore Umapathi, Bhaskaran Unnikrishnan, Alireza Vakilian, Sahel Valadan Tahbaz, Rohollah Valizadeh, Jef Van den Eynde, Priya Vart, Shoban Babu Varthya, Siavash Vaziri, Balachandar Vellingiri, Narayanaswamy Venketasubramanian, Georgios-Ioannis Verras, Dominique Vervoort, Jorge Hugo Villafañe, Sergey Konstantinovitch Vladimirov, Simona Ruxandra Volovat, Theo Vos, Loc Tri Vu, Isidora S Vujcic, Yasir Waheed, Waseem Wahood, Mandaras Tariku Walde, Fang Wang, Shu Wang, Yanzhong Wang, Yuan-Pang Wang, Muhammad Waqas, Abdul Waris, Kosala Gayan Weerakoon, Abrha Hailay Weldemariam, Ronny Westerman, Dakshitha Praneeth Wickramasinghe, Nuwan Darshana Wickramasinghe, Barbara Willekens, Andrea Sylvia Winkler, Charles D A Wolfe, Ai-Min Wu, Sarah Wulf Hanson, Suowen Xu, Xiaoyue Xu, Ali Yadollahpour, Galal Yahya, Kazumasa Yamagishi, Lin Yang, Yao Yao, Sisay Shewasinad Yehualashet, Alex Yeshaneh, Metin Yesiltepe, Siyan Yi, Arzu Yiğit, Vahit Yiğit, Dong Keon Yon, Naohiro Yonemoto, Yuyi You, Mustafa Z Younis, Chuanhua Yu, Hadiza Yusuf, Siddhesh Zadey, Mohammad Zahedi, Fathiah Zakham, Nazar Zaki, Alireza Zali, Giulia Zamagni, Ramin Zand, Ghazal G Z Zandieh, Moein Zangiabadian, Amin Zarghami, Mikhail Sergeevich Zastrozhin, Mohammed G M Zeariya, Francis Zeukeng, Chen Zhang, Haijun Zhang, Yunquan Zhang, Hanqing Zhao, Yang Zhao, Hengxing Zhou, Bin Zhu, Magdalena Zielińska, Yossef Teshome Zikarg, and Mohammad Zoladl.

Drafting the work or revising it critically for important intellectual content

Yohannes Habtegiorgis Abate, Jaffar Abbas, Madineh Akram Abbasi, Hedayat Abbastabar, Michael Abdelmasseh, Mohammad Abdollahi, Mozhan Abdollahi, Rami Abd-Rabu, Auwal Abdullahi, Aidin Abedi, Vida Abedi, Roberto Ariel Abeldaño Zuñiga, Hassan Abidi, Olumide Abiodun, Hassan Abolhassani, Victor Aboyans, Ahmed Abualhasan, Eman Abu-Gharbieh, Salahdein Aburuz, Lawan Hassan Adamu, Isaac Yeboah Addo, Oladimeji M Adebayo, Victor Adekanmbi, Qorinah Estiningtyas Sakilah Adnani, Saryia Adra, Aanuoluwapo Adeyimika Afolabi, Ali Afraz, Saira Afzal, Antonella Agodi, Bright Opoku Ahinkorah, Danish Ahmad, Ayman Ahmed, Haroon Ahmed, Jivan Qasim Ahmed, Luai A. Ahmed, Muktar Beshir Ahmed, Marjan Ajami, Olufemi Ajumobi, Seyed Esma'il Akade, Morteza Akbari, Hossein Akbarialiabad, Shiva Akhlaghi, Karolina Akinosoglou, Rufus Olusola Akinyemi, Fares Alahdab, Tareq Mohammed Ali AL-Ahdal, Rasmieh Mustafa Al-amer, Mohammed Albashtawy, Mohammad T. AlBataineh, Khalifah A Aldawsari, Hediyeh Alemi, Sharifullah Alemi, Abdelazeem M. Algammal, Fadwa Alhalaiqa Naji Alhalaiqa, Robert Kaba Alhassan, Abid Ali, Liaqat Ali, Mohammed Usman Ali, Rafat Ali, Shahid Ali, Syed Shujait Shujait Ali, Zahid Ali, Sheikh Mohammad Alif, Ahmednur Adem Aliyi, Mohamad Aljofan, Suvarna Alladi,

Sami Almustanyir, Basem Al-Omari, Jaber S Alqahtani, Rustam Al-Shahi Salman, Jaffar A. Al-Tawfiq, Nelson Alvis-Guzman, Yaser Mohammed Al-Worafi, Hany Aly, Safwat Aly, Karem H Alzoubi, Reza Amani, Alireza Amindarolzarbi, Sohrab Amiri, Mohammad Hosein Amirzade-Iranaq, Hubert Amu, Dickson A Amugsi, Ganiyu Adeniyi Amusa, Jimoh Amzat, Robert Ancuceanu, Deanna Anderlini, David B Anderson, Catalina Liliana Andrei, Teklit W Angesom, Abhishek Anil, Razique Anwer, Demelash Areda, Hany Ariffin, Hidayat Arifin, Mesay Arkew, Johan Ärnlöv, Mahwish Arooj, Kurnia Dwi Artanti, Raphael Taiwo Aruleba, Ali A Asadi-Pooya, Muhammad Ashraf, Kendalem Asmare Atalell, Seyyed Shamsadin Athari, Prince Atorkey, Maha Moh'd Wahbi Atout, Alok Atreya, Avinash Aujayeb, Abolfazl Avan, Beatriz Paulina Ayala Quintanilla, Sina Azadnajafabad, Zahra Azizi, Khalil Azizian, Ahmed Y Azzam, Mahsa Babaei, Muhammad Badar, Ashish D Badiye, Soroush Baghdadi, Sara Bagherieh, Ruhai Bai, Atif Amin Baig, Ovidiu Constantin Baltatu, Maciej Banach, Soham Bandyopadhyay, Mehmet Firat Baran, Martina Barchitta, Mainak Bardhan, Suzanne Lyn Barker-Collo, Till Winfried Bärnighausen, Amadou Barrow, Davood Bashash, Hamideh Bashiri, Hameed Akande Bashiru, Afisu Basiru, João Diogo Basso, Sanjay Basu, Abdul-Monim Mohammad Batiha, Bernhard T Baune, Neeraj Bedi, Ahmet Begde, Babak Behnam, Amir Hossein Behnoush, Maryam Beiranvand, Yannick Béjot, Alehegn Bekele, Melaku Ashagrie Belete, Uzma Iqbal Belgaumi, Maryam Bemanalizadeh, Bright Benfor, Derrick A. Bennett, Isabela M Bensenor, Kebede A Beyene, Abhishek Bhadra, Devidas S. Bhagat, Sonu Bhaskar, Ajay Nagesh Bhat, Vivek Bhat, Gurjit Kaur Bhatti, Jasvinder Singh Bhatti, Rajbir Bhatti, Boris Bikbov, Mariah Malak Bilalaga, Atanu Biswas, Saeid Bitaraf, Veera R Bitra, Tone Bjørge, Virginia Bodolica, Dejana Braithwaite, Hermann Brenner, Andrey Briko, Maria L. Bringas Vega, Julie Brown, Christine M. Budke, Danilo Buonsenso, Richard A Burns, Yasser Bustanji, Muhammad Hammad Butt, Lucas Scotta Cabral, Florentino Luciano Caetano dos Santos, Chao Cao, Hélène Carabin, Andre F Carvalho, Carlos A Castañeda-Orjuela, Ferrán Catalá-López, Vanessa Cavallera, Arthur Caye, Luca Cegolon, Muthia Cenderadewi, Ester Cerin, Pamela R Uscamaita Chacón-Uscamaita, Jeffrey Shi Kai Chan, Gashaw Sisay Chanie, Vijay Kumar Chattu, Hui Chen, Jianqi Chen, Gerald Chi, Fatemeh Chichagi, Saravana Babu Chidambaram, Patrick R Ching, Yuen Yu Chong, Hitesh Chopra, Rajiv Chowdhury, Dinh-Toi Chu, Eric Chung, Alyssa Columbus, Joao Conde, Paolo Angelo Cortesi, Vera Marisa Costa, Ewerton Cousin, Rosa A S Couto, Michael H Criqui, Natália Cruz-Martins, Sriharsha Dadana, Zhaoli Dai, Emanuele D'Amico, Hadi A Danawi, Ana Maria Dascalu, Nihar Ranjan Dash, Mohsen Dashti, Alejandro de la Torre-Luque, Amin Dehghan, Hiwot Dejene, Andreas K Demetriades, Hardik Dineshbhai Desai, Vinoth Gnana Chellaiyan Devanbu, Samath Dhamminda Dharmaratne, Meghnath Dhimal, Diana Dias da Silva, Daniel Diaz, Mahmoud Dibas, Delaney D Ding, Monica Dinu, Thanh Chi Do, Khanh Duy Khanh Doan, Milad Dodangeh, Mohamed Fahmy Doheim, Klara Georgieva Dokova, Deepa Dongarwar, Haneil Larson Dsouza, John Dube, Oyewole Christopher Durojaiye, Sulagna Dutta, Arkadiusz Marian Dziedzic, Negin Eissazade, Nevine El Nahas, Iman El Sayed, Mohammad Amin Elahi Najafi, Iffat Elbarazi, Noha Mousaad Elemam, Frank J Elgar, Islam Y Elgendy, Muhammed Elhadi, Richard G Ellenbogen, Omar Abdelsadek Abdou Elmeligy, Mohamed A Elmonem, Mohammed Elshaer, Ibrahim Elsohaby, Mehdi Emamverdi, Theophilus I Emeto, Matthias Endres, Christopher Imokhuede Esezobor, Sharareh Eskandarieh, Adeniyi Francis Fagbamigbe, Ayesha Fahim, Ali Faramarzi, Jawad Fares, Mohsen Farjoud Kouhanjani, Andre Faro, Ali Fatehizadeh, Mobina Fathi, Valery L Feigin, Seyed-Mohammad Fereshtehnejad, Alize J Ferrari, Nuno Ferreira, Getahun Fetensa, Neda Firouraghi, Florian Fischer, Ana Catarina Fonseca, Lisa M Force, Arianna Fornari, Behzad Foroutan, Takeshi Fukumoto, Muktar A. Gadanya, Yaseen Galali, Nasrin Galehdar, Aravind P Gandhi, Balasankar Ganesan, Rupesh K. Gautam, Teshome Gebre, Mesfin Gebrehiwot, Gebreamlak Gebremedhn Gebremeskel, Haftay Gebremedhin Gebreslassie, Lemma Getacher, Fataneh Ghadirian, Fariborz Ghaffarpasand, MohammadReza Ghasemi, Sailaja Ghimire, Ali Gholami, Ali Gholamrezanezhad, Elena Ghotbi, Sherief Ghozy, Alessandro Gialluisi, Paramjit Singh Gill, Logan M. Glasstetter, Ali Golchin, Davide Golinelli, Mansueto Gomes-Neto, Alessandra C Goulart, Anmol Goyal, Richard J Gray, Michal Grivna, Giovanni Guarducci, Damitha Asanga Gunawardane, Bhawna Gupta, Rajeev Gupta, Sapna Gupta, Veer Bala Gupta, Vivek Kumar Gupta, Reyna

Alma Gutiérrez, Farrokh Habibzadeh, Vladimir Hachinski, Rasool Haddadi, Mostafa Hadei, Najah R Hadi, Nils Haep, Teklehaimanot Gereziher Haile, Arvin Haj-Mirzaian, Brian J Hall, Rabih Halwani, Sajid Hameed, Mohammad Hamiduzzaman, Ahmad Hammoud, Hannah Han, Nasrin Hanifi, Graeme J Hankey, Md. Abdul Hannan, Harapan Harapan, Netanja I. Harlianto, Josep Maria Haro, Nicholas Nathaniel Hartman, Ahmed I. Hasaballah, Faizul Hasan, Hamidreza Hasani, Amr Hassan, Shoaib Hassan, Johannes Haubold, Simon I Hay, Khezar Hayat, Golnaz Heidari, Reza Heidari-Soureshjani, Hamed Hesami, Kamal Hezam, Yuta Hiraike, Howard J. Hoffman, Ramesh Holla, Nobuyuki Horita, Md Mahbub Hossain, Sahadat Hossain, Sorin Hostiuc, Junjie Huang, Md. Nazmul Huda, Javid Hussain, Nawfal R Hussein, Hong-Han Huynh, Segun Emmanuel Ibitoye, Mehran Ilaghi, Olayinka Stephen Ilesanmi, Irena M. Ilic, Milena D. Ilic, Mustapha Immurana, Farideh Iravanpour, Sheikh Mohammed Shariful Islam, Faisal Ismail, Hiroyasu Iso, Gaetano Isola, Chidozie C D Iwu, Mahalaxmi Iyer, Ali Jaan, Louis Jacob, Morteza Jafarinia, Abdollah Jafarzadeh, Kasra Jahankhani, Nader Jahanmehr, Haitham Jahrami, Mihajlo Jakovljevic, Roland Dominic G. Jamora, Somnath Jana, Nilofer Javadi, Saad Javed, Saad Javeed, Sathish Kumar Jayapal, Shubha Jayaram, Catherine Owens Johnson, Walter D Johnson, Jost B. Jonas, Abel Joseph, Nitin Joseph, Mikk Jürisson, Ali Kabir, Gebisa Guyasa Kabito, Vidya Kadashetti, Fatemeh Kafi, Farnaz Kalantar, Feroze Kaliyadan, Ashwin Kamath, Himal Kandel, Mehrdad Karajizadeh, Shama D Karanth, André Karch, Aliasghar Karimi, Hanie Karimi, Hengameh Kasraei, Nicholas J Kassebaum, Joonas H Kauppila, Navjot Kaur, Gbenga A. Kayode, Foad Kazemi, Leila Keikavoosi-Arani, Himanshu Khajuria, Amirmohammad Khalaji, Faham Khamesipour, Mohammad Khammarnia, Maseer Khan, Moien AB Khan, Yusra H Khan, Mahammed Ziauddin Khan suheb, Khaled Khatab, Haitham Khatatbeh, Moawiah Mohammad Khatatbeh, Sorour Khateri, Mahalaqua Nazli Khatib, Hamid Reza Khayat Kashani, Saeid Kian, Grace Kim, Jihee Kim, Min Seo Kim, Yun Jin Kim, Adnan Kisa, Sezer Kisa, Mika Kivimäki, Sonali Kochhar, Farzad Kompani, Ai Koyanagi, Michael A Kravchenko, Kewal Krishan, Barthelemy Kuate Defo, Md Abdul Kuddus, Satyajit Kundu, Maria Dyah Kurniasari, Dian Kusuma, Carlo La Vecchia, Muhammad Awwal Ladan, Chandrakant Lahariya, Tea Lallukka, Faris Hasan Lami, Iván Landires, Berthold Langguth, Savita Lasrado, Kamaluddin Latief, Kaveh Latifinaibin, Matthew B Laurens, Basira Kankia Lawal, Long Khanh Dao Le, Thao Thi Thu Le, Caterina Ledda, Munjae Lee, Wei-Chen Lee, Matilde Leonardi, Christine Linehan, Chaojie Liu, Wei Liu, Chun-Han Lo, Warren David Lo, Platon D Lopukhov, László Lorenzovici, Stefan Lorkowski, Joana A Loureiro, Giancarlo Lucchetti, Ricardo Lutzky Saute, Zheng Feei Ma, Mahmoud Mabrok, Monika Machoy, Farzan Madadizadeh, Mohammed Magdy Abd El Razek, Nastaran Maghbouli, Soleiman Mahjoub, Elaheh Malakan Rad, Ahmad Azam Malik, Tauqeer Hussain Mallhi, Deborah Carvalho Malta, Aseer Manilal, Vahid Mansouri, Bishnu P. Marasini, Hamid Reza Marateb, Francisco Rogerlândio Martins-Melo, Miquel Martorell, Winfried März, Roy Rillera Marzo, João Massano, Yasith Mathangasinghe, Elezebeth Mathews, Andrea Maugeri, Mahsa Mayeli, Maryam Mazaheri, Colm McAlinden, John J. McGrath, Jitendra Kumar Meena, Max L Mehlman, Man Mohan Mehndiratta, Max Alberto Mendez Mendez-Lopez, Walter Mendoza, Oliver Mendoza-Cano, Ritesh G Menezes, Mohsen Merati, Atte Meretoja, Tomislav Mestrovic, Tianyue Mi, Tomasz Miazgowski, Irmina Maria Michalek, Ephrem Tesfaye Mihretie, Le Huu Nhat Minh, Reza Mirfakhraie, Mehdi Mirzaei, Awoke Misganaw, Biru Abdissa Mizana, Esmaeil Mohammadi, Hiwa Mohammadi, Soheil Mohammadi, Marita Mohammadshahi, Mustapha Mohammed, Salahuddin Mohammed, Shafiu Mohammed, Hoda Mojiri-forushani, Nagabhishek Moka, Ali H Mokdad, Sabrina Molinaro, Lorenzo Monasta, Fateme Montazeri, Maryam Moradi, Yousef Moradi, Maziar Moradi-Lakeh, Paula Moraga, Shane Douglas Morrison, Abbas Mosapour, Majid Motaghinejad, Parsa Mousavi, Seyed Ehsan Mousavi, Faraz Mughal, George Duke Mukoro, Admir Mulita, Francesk Mulita, Christopher J L Murray, Fungai Musaigwa, Ahmad Mustafa, Ghulam Mustafa, Sathish Muthu, Ahamarshan Jayaraman Nagarajan, Firzan Nainu, Hastyar Hama Rashid Najmuldeen, Noureddin Nakhostin Ansari, Gopal Nambi, Shumaila Nargus, Bruno Ramos Nascimento, Abdallah Y Naser, Hadis Nasoori, Ahmed Nasreldein, Zuhair S Natto, Javaid Nauman, Biswa Prakash Nayak, Mohammad Negaresh, Hadush Negash, Ionut Negoi, Ruxandra Irina Negoi, Serban Mircea Negru,

Seyed Aria Nejadghaderi, Mohammad Hadi Nematollahi, Charles Richard James Newton, Dang H Nguyen, Hien Quang Nguyen, Phat Tuan Nguyen, Van Thanh Nguyen, Robina Khan Niazi, Emma Nichols, Taxiarchis Konstantinos Nikolouzakis, Vikram Niranjan, Lawrence Achilles Nnyanzi, Bo Norrving, Chisom Adaobi Nri-Ezedi, George Ntaios, Virginia Nuñez-Samudio, Dieta Nurrika, Bogdan Oancea, Ismail A. Odetokun, In-Hwan Oh, Sylvester Reuben Okeke, Akinkunmi Paul Okekunle, Osaretin Christabel Okonji, Patrick Godwin Okwute, Andrew T Olagunju, Matifan Dereje Olana, Matthew Idowu Olatubi, Gláucia Maria Moraes Oliveira, Bolajoko Olubukunola Olusanya, Kanyin Liane Ong, Obinna E Onwujekwe, Michal Ordak, Doris V. Ortega-Altamirano, Uchechukwu Levi Osuagwu, Nikita Otstavnov, Mayowa O Owolabi, Mahesh Padukudru P A, Kevin Pacheco-Barrios, Jagadish Rao Padubidri, Pramod Kumar Pal, Claudia Palladino, Raffaele Palladino, Feng Pan, Songhomitra Panda-Jonas, Ashok Pandey, Jeyaraj Durai Pandian, Helena Ullyartha Pangaribuan, Ioannis Pantazopoulos, Shahina Pardhan, Pragyan Paramita Parija, Romil R Parikh, Ashwaghosha Parthasarathi, Ava Pashaei, Jay Patel, Shankargouda Patil, Dimitrios Patoulias, Shrikant Pawar, Paolo Pedersini, Umberto Pensato, David M. Pereira, Jeevan Pereira, Maria Odete Pereira, Mario F P Peres, Norberto Perico, Ionela-Roxana Petcu, Fanny Emily Petermann-Rocha, Hoang Tran Pham, Michael R Phillips, Gabriel D. Pinilla-Monsalve, Dimitri Poddighe, Burcu Polat, Constance Dimity Pond, Govinda Raj Poudel, Alireza Pouramini, Mohammad Pourfridoni, Peralam Yegneswaran Prakash, Sanjay Prakash, Elton Junio Sady Prates, Natalie Pritchett, Nameer Hashim Qasim, Ibrahim Qattea, Venkatraman Radhakrishnan, Hadi Raeisi Shahraki, Ibrar Rafique, Alberto Raggi, Pankaja Raghav Raghav, Fakher Rahim, Mahban Rahimifard, Mohammad Hifz Ur Rahman, Shayan Rahmani, Hamed Rahmani Youshanlouei, Masoud Rahmati, Sheetal Raj Moolambally, Ali Rajabpour-Sanati, Hazem Ramadan, Premkumar Ramasubramani, Sheena Ramazanu, Nemanja Rancic, Indu Ramachandra Rao, Sowmya J Rao, Deepthi Rapaka, Vahid Rashedi, Ahmed Mustafa Rashid, Mehran Rashidi Alavijeh, Ashkan Rasouli-Saravani, Salman Rawaf, Elrashdy Moustafa Mohamed Redwan, Atefe Rekabi Bana, Giuseppe Remuzzi, Nazila Rezaei, Nima Rezaei, Taeho Gregory Rhee, Abanoub Riad, Stephen R Robinson, Mónica Rodrigues, Jefferson Antonio Buendia Rodriguez, Leonardo Roever, Michele Romoli, Luca Ronfani, Priyanka Roy, Koushik Roy Pramanik, Enrico Rubagotti, Milagros A Ruiz, Tom C Russ, Katharina S Sunnerhagen, Aly M A Saad, Korosh Saber, Morteza SaberiKamarposhti, Basema Saddik, Umar Saeed, Usman Saeed, Mahdi Safdarian, Rajesh Sagar, Dominic Sagoe, Fatemeh Saheb Sharif-Askari, Narjes Saheb Sharif-Askari, Amirhossein Sahebkar, Soumya Swaroop Sahoo, Mohammad Ali Sahraian, Hossein Salehi omran, Marwa Rashad Salem, Sohrab Salimi, Hossein Samadi Kafil, Sara Samadzadeh, Saad Samargandy, Yoseph Leonardo Samodra, Vijaya Paul Samuel, Abdallah M Samy, Francesco Sanmarchi, Damian Francesco Santomauro, Milena M. Santric-Milicevic, Aswini Saravanan, Arash Sarveazad, Maheswar Satpathy, Mete Saylan, Mehdi Sayyah, Nikolaos Scarmeas, Nicoline Schiess, Art Schuermans, David C Schwebel, Katrin Maria Seeher, Siddharthan Selvaraj, Pallav Sengupta, Dragos Serban, Yashendra Sethi, Allen Seylani, Mohammad Shabani, Maryam Shabany, Mahan Shafie, Saeed Shahabi, Samiah Shahid, Moyad jamal Shahwan, KS Shaji, Adisu Tafari T Shama, Muhammad Aaqib Shamim, Mehran Shams-Beyranvand, Mohammad Anas Shamsi, Mohd Shanawaz, Medha Sharath, Amin Sharifan, Manoj Sharma, Rajesh Sharma, Bereket Beyene Shashamo, Shashank Shekhar, Jiabin Shen, Mika Shigematsu, Aminu Shittu, K M Shivakumar, Fereshteh Shokri, Sina Shool, Seyed Afshin Shorofi, Sunil Shrestha, Akhenaten Benjamin Siankam Tankwanchi, Emmanuel Edwar Siddig, Inga Dora Sigfusdottir, João Pedro Silva, Luís Manuel Lopes Rodrigues Silva, Paramdeep Singh, Surjit Singh, Shravan Sivakumar, Abdullah Al Mamun Sohag, Ranjan Solanki, Hamidreza Soleimani, Solikhah Solikhah, Yerukneh Solomon, Yonatan Solomon, Suhang Song, Joseph R Starnes, Antonina V Starodubova, Dan J Stein, Timothy J Steiner, Jaimie D Steinmetz, Muhammad Suleman, Abida Sultana, David Sunkersing, Chandan Kumar Swain, Mindy D Szeto, Shima Tabatabai, Mohammad Tabish, Majid Taheri, Azin Tahvildari, Ardeshir Tajbakhsh, Mircea Tampa, Jacques JL Lukenze Tamuzi, Ker-Kan Tan, Haosu Tang, Nathan Y. Tat, Vivian Y Tat, Razieh Tavakoli Oliaee, Seyed Mohammad Tavangar, Arian Tavasol, Yibekal Manaye Tefera, Arash Tehrani-Banihashemi, Worku Animaw Temesgen, Mohamad-Hani Temsah, Masayuki Teramoto, Edosa

Geta Tesfaye, Riki Tesler, Ocean Thakali, Pugazhenthan Thangaraju, Amanda G Thrift, Jansje Henny Vera Ticoalu, Tala Tillawi, Marcello Tonelli, Marcos Roberto Tovani-Palone, Nghia Minh Tran, Ngoc-Ha Tran, Phu Van Tran, Samuel Joseph Tromans, Thomas Clement Truelsen, Thien Tan Tri Tai Truyen, Aristidis Tsatsakis, Evangelia Eirini Tsermpini, Chukwudi S. Ubah, Aniefiok John Udoakang, Inam Ulhaq, Muhammad Umair, Srikanth Umakanthan, Krishna Kishore Umapathi, Brigid Unim, Bhaskaran Unnikrishnan, Asokan Govindaraj Vaithinathan, Sahel Valadan Tahbaz, Jef Van den Eynde, Priya Vart, Shoban Babu Varthya, Tommi Juhani Vasankari, Balachandar Vellingiri, Narayanaswamy Venketasubramanian, Georgios-Ioannis Verras, Dominique Vervoort, Jorge Hugo Villafañe, Leonardo Villani, Andres Fernando Vinueza Veloz, Maria Viskadourou, Vasily Vlassov, Simona Ruxandra Volovat, Theo Vos, Loc Tri Vu, Birhanu Wagaye, Waseem Wahood, Fang Wang, Shu Wang, Yanzhong Wang, Yuan-Pang Wang, Kosala Gayan Weerakoon, Robert G Weintraub, Ronny Westerman, Joanna L Whisnant, Dakshitha Praneeth Wickramasinghe, Nuwan Darshana Wickramasinghe, Barbara Willekens, Andrea Sylvia Winkler, Charles D A Wolfe, Ai-Min Wu, Sajad Yaghoubi, Galal Yahya, Kazumasa Yamagishi, Lin Yang, Yao Yao, Sisay Shewasinad Yehualashet, Alex Yeshaneh, Metin Yesiltepe, Arzu Yiğit, Vahit Yiğit, Dong Keon Yon, Naohiro Yonemoto, Hadiza Yusuf, Mohammad Zahedi, Fathiah Zakham, Ramin Zand, Mikhail Sergeevich Zastrozhin, Zelalem Banjaw Zegeye, Francis Zeukeng, chunxia Zhai, Haijun Zhang, Hanqing Zhao, Yang Zhao, Magdalena Zielińska, Yossef Teshome Zikarg, and Mohammad Zoladl.

Managing the estimation or publications process

Auwal Abdullahi, Saira Afzal, Mohammed Albashtawy, Hediyeh Alemi, Liaqat Ali, Joseph Uy Almazan, Hany Aly, Ahmed Y Azzam, Muhammad Hammad Butt, Rosa A S Couto, Hiwot Dejene, M Ashworth Dirac, Thanh Chi Do, Khanh Duy Khanh Doan, Ali Fatehizadeh, Hailey Hagins, Teklehaimanot Gereziher Haile, Simon I Hay, Hong-Han Huynh, Farideh Iravanpour, Gaetano Isola, Morteza Jafarinia, Sathish Kumar Jayapal, Aliasghar Karimi, Mohammad Khammarnia, Mahalaqua Nazli Khatib, Chandrakant Lahariya, Thao Thi Thu Le, Mohammed Magdy Abd El Razek, Le Huu Nhat Minh, Mustapha Mohammed, Ali H Mokdad, Christopher J L Murray, Hastyar Hama Rashid Najmuldeen, Olivia D Nesbit, Hien Quang Nguyen, Phat Tuan Nguyen, Van Thanh Nguyen, Bolajoko Olubukunola Olusanya, Kanyin Liane Ong, Mahesh Padukudru P A, Hoang Tran Pham, Nameer Hashim Qasim, Nemanja Rancic, Aly M A Saad, Korosh Saber, Abdallah M Samy, Maheswar Satpathy, Mehdi Sayyah, Sunil Shrestha, Jaimie D Steinmetz, Yibekal Manaye Tefera, Theo Vos, and Mikhail Sergeevich Zastrozhin.
